# Discovery of Pyranoquinolone Diterpenoids Biosynthesized through Oxidative Indole Rearrangement and Atypical Terpene Cyclization

**DOI:** 10.1021/jacs.6c07454

**Published:** 2026-06-10

**Authors:** Yuya Kakumu, Eric J. N. Helfrich

**Affiliations:** † 9173 Institute of Molecular Biosciences, Goethe University Frankfurt, Max-von-Laue Strasse 9, Frankfurt am Main 60438, Germany; ‡ Senckenberg Research Institute and Natural History Museum Frankfurt, Senckenberganlage 25, Frankfurt am Main 60325, Germany

## Abstract

Pyr4-like transmembrane terpene cyclases (TCs) are key enzymes in the biosynthesis of diverse fungal meroterpenoids. However, their roles in bacterial meroterpenoid biosynthesis remain largely unexplored. Here, we charted the biosynthetic space of bacterial Pyr4-like TCs and discovered zigralone A, a quinolone diterpenoid. Functional characterization of the gene cluster established a multienzymatic assembly of its 6/6/6/5/6/6/6-heptacyclic scaffold. Key transformations include an oxidative indole rearrangement catalyzed by a thiamine pyrophosphate-dependent enzyme to form a 4-quinolone. Subsequent cytochrome P450-mediated epoxidation and hydroxylation direct carbo/oxacyclizations catalyzed by a Pyr4-like TC, generating a bilobed architecture of a pyranoquinolone and a 6/6/6-tricyclic ring system. Further P450-mediated oxidations and nonenzymatic hemiketalization furnish a spiro-fused tetrahydrofuran, completing the heptacyclic framework. Notably, zigralone A congeners exhibit potent antimicrobial activity against *Staphylococcus aureus*. This work provides insights into bacterial Pyr4-like TC-associated pathways, expanding the biosynthetic logic underlying the assembly of complex bioactive meroterpenoids.

## Graphical abstract

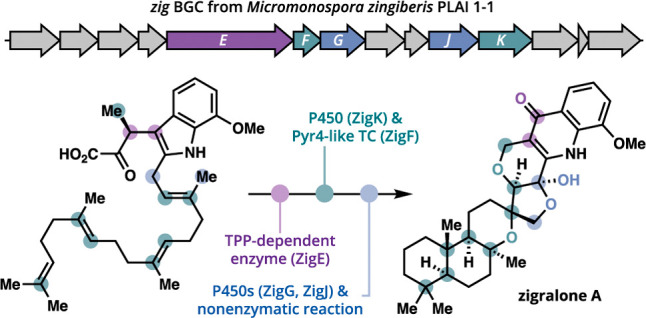

Stereoselective cyclization of prenyl chains represents a critical process in terpenoid biosynthesis. These reactions are catalyzed by terpene cyclases (TCs), which convert linear polyene precursors into topologically complex carbocyclic skeletons with multiple stereocenters through carbocation-mediated reactions.^1^ Canonical TCs are classified into two types based on their modes of carbocation generation.^2^ Type I TCs activate oligoprenyl pyrophosphates and generate allylic cations, whereas type II TCs protonate alkenes or epoxides to form tertiary carbocations. In addition to canonical TCs, several other enzymes have been identified to catalyze TC-like reactions through similar carbocation-based or distinct mechanisms, such as Cope rearrangements.^3^ Following TC-catalyzed scaffold formation, various tailoring enzymes further modify the core structures, giving rise to densely functionalized terpenoids.^4^

Pyr4-like transmembrane TCs are a family of noncanonical TCs characterized by compact architectures.^5^ Their cyclization mechanism resembles that of type II TCs, with an acidic residue acting as a Brønsted acid to initiate cyclization.^6^ Despite this functional similarity, Pyr4-like TCs show no sequence homology to type II TCs, highlighting their distinct evolutionary origin. Since the first characterization of Pyr4 in the pyripyropene A biosynthetic pathway,^6a^ more than 60 fungal homologues have been associated with the biosynthesis of diverse meroterpenoids^7^ and triterpenoids^8^ (Figure 1). These (mero)­terpenoids frequently exhibit potent bioactivities and have garnered attention as leads for pharmaceutical and agrochemical development, as exemplified by afidopyropen, a pyripyropene-derived insecticide.^9^ In contrast to the structural diversity of fungal Pyr4-like TC-derived (mero)­terpenoids and the well-characterized pathways underlying their assembly,^10^ bacterial meroterpenoids produced through analogous pathways remain largely unexplored, with only a few examples identified to date.^11^ Here, we report the genome mining-driven discovery of zigralones, a new family of pyranoquinolone-based meroditerpenoids, and we elucidate their biosynthesis through heterologous pathway reconstruction and *in vitro* assays.

**Figure 1:**
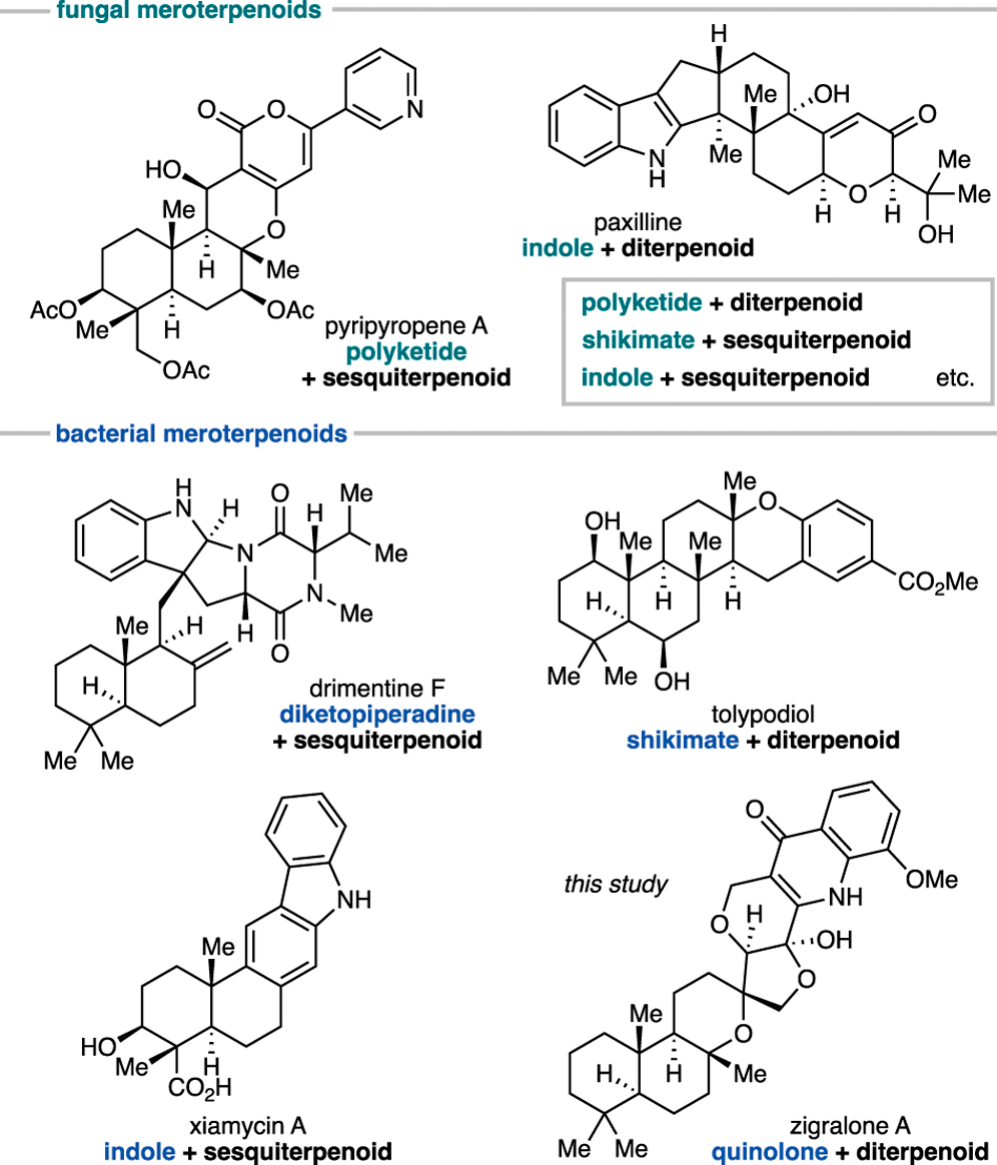
Examples of fungal and bacterial meroterpenoids biosynthesized by Pyr4-like TCs.

We began by exploring the global sequence space of bacterial Pyr4-like TCs. To this end, we constructed a custom profile hidden Markov model (pHMM) based on 62 previously identified Pyr4-like TCs including six bacterial homologues, two of which remain uncharacterized (Table S1). Searches of the NCBI nonredundant database restricted to bacterial taxa using the custom pHMM yielded 1107 candidate sequences at a 95% identity threshold. Candidates were subsequently filtered based on the presence of genes associated with terpenoid biosynthesis in the genomic neighborhood (see Supporting Information). This filtration process refined the dataset into 264 sequences predominantly originating from Actinomycetota (69%) (Table S2).

We next classified the filtered proteins into putative isofunctional groups using a sequence similarity network (SSN)^12^ (Figure 2A). The two largest clusters in the SSN contained known Pyr4-like TCs, XiaH (xiamycin A) and TylF (tolypodiol). The latter cluster also contained GroF, a TC involved in the biosynthesis of the guanidine triterpenoid gromomycins (Figure S1).^13^ We turned our attention to a smaller cluster of 10 sequences predominantly derived from rare actinobacteria. These Pyr4-like TCs form a distinct subclade in a maximum-likelihood phylogenetic tree (Figure S2). Moreover, their genes are consistently colocalized with those encoding an atypical thiamine pyrophosphate (TPP)-dependent enzyme composed of two acetolactate synthase subunits and at least two cytochrome P450 monooxygenases (P450s) (Figure S3). We selected a BGC (*zig*) identified in the genome of *Micromonospora zingiberis* PLAI 1-1 for further investigations. The *zig* BGC encodes an aminotransferase, a polyprenyl synthase, a UbiA-like prenyltransferase (PT), two methyltranserases (MTs), a TPP-dependent enzyme, a Pyr4-like TC, five P450s, a protein containing a domain of unknown function 6875 (DUF6875), and a ferredoxin (Fdx) (Figure 2B, Table S3).

**Figure 2:**
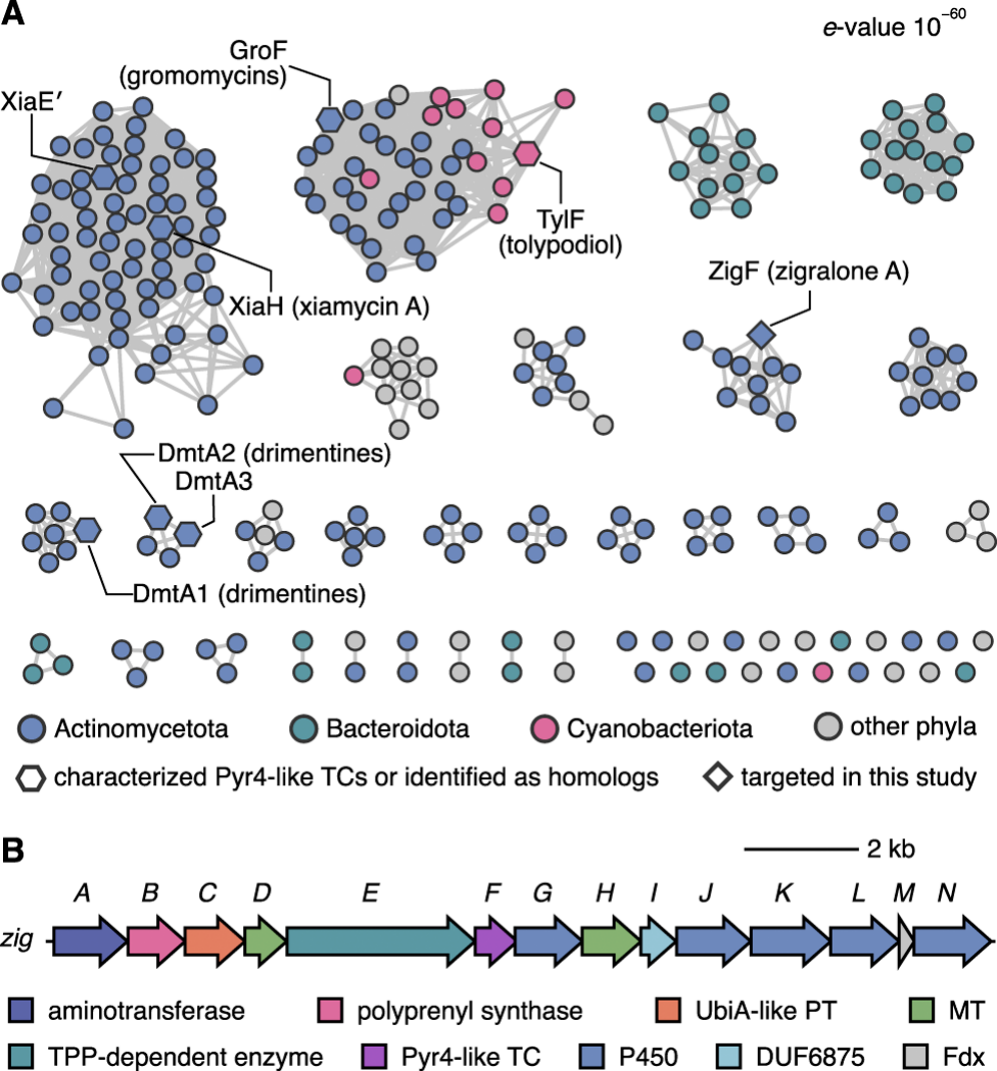
Genome mining of bacterial Pyr4-like TCs. (A) A SSN of 264 bacterial Pyr4-like TCs. (B) The *zig* BGC from *M. zingiberis*.

To characterize products derived from the *zig* BGC, the gene cluster was amplified and cloned into pSET152 under the control of the constitutive SP44 promoter^14^ and heterologously expressed in *Streptomyces albus* J1074, *Streptomyces lividans* TK64, and *Streptomyces coelicolor* M1154 (Tables S4–S6). LC-MS analysis of extracts from *S. albus*::*zig* cultures revealed the production of seven metabolites (**1**–**7**) (Figure 3). These compounds, designated zigralones A–G, were also produced in *S. lividans*::*zig* and *S. coelicolor*::*zig* (Figure S4). Additionally, *zig*-derived metabolites were detected in *M. zingiberis* culture extracts in trace amounts (Figure S5). Large-scale fermentation of *S. albus*::*zig* followed by chromatographic separations enabled the isolation of **1**–**5** for structure elucidation, whereas **6** and **7** could not be readily purified and were instead obtained during subsequent biosynthetic investigations.

**Figure 3:**
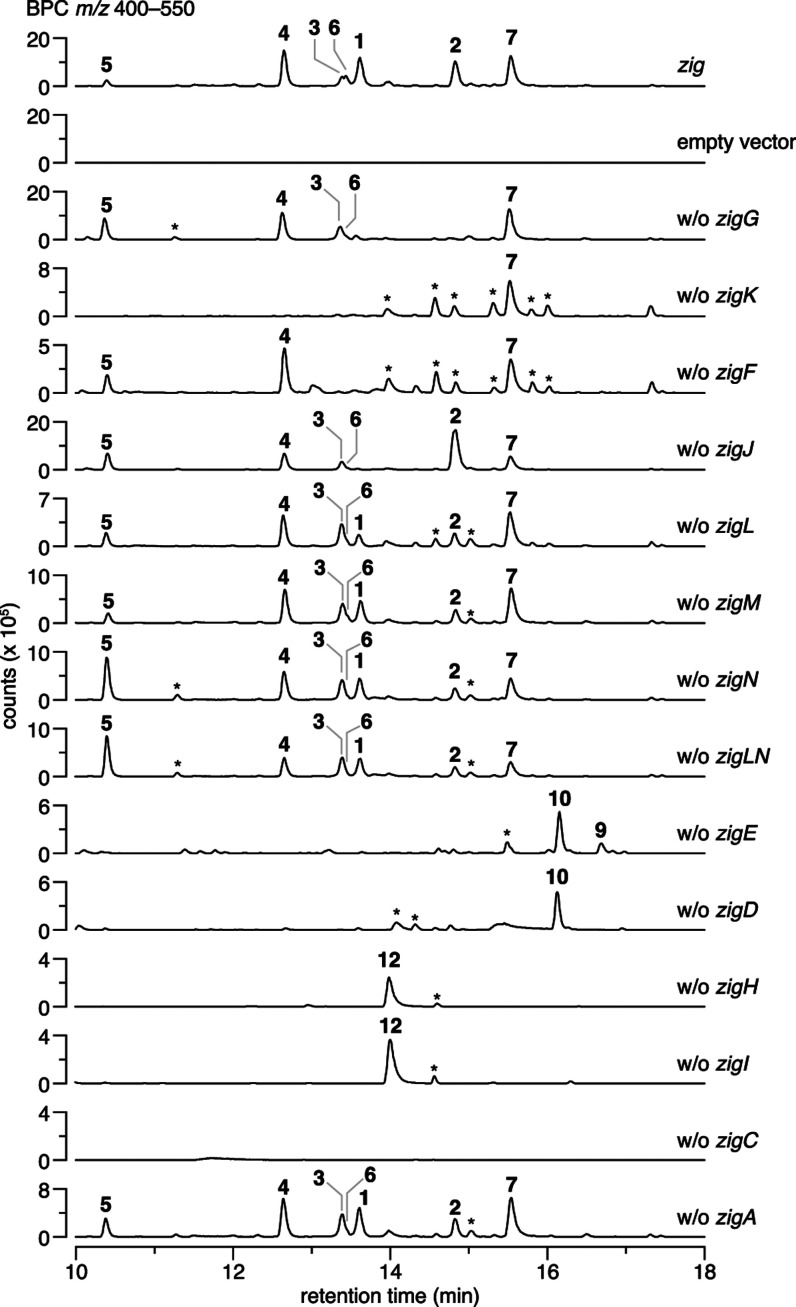
LC-MS analysis of *S. albus* expressing different combinations of *zig* genes. Asterisks denote peaks of unrelated compounds or products whose structures were not determined due to low yield.

The molecular formula of **1** was established as C_31_H_41_NO_6_ based on HRMS data (*m*/*z* 524.3007, [M + H]^+^). Analysis of 1D and 2D NMR data, together with characteristic UV absorptions (λ_max_ 237, 325, 338 nm), revealed the presence of an 8-methoxy-4-quinolone fused to a 3,6-dihydropyran at C-2′/C-3′ (Table S7). The terpenoid portion was assigned as a manoyl oxide-type skeleton spiro-fused to a tetrahydrofuran that is annulated to the pyranoquinolone moiety. The planar structure of **1** was thus established as a 6/6/6/5/6/6/6-heptacyclic framework (Scheme 1). HRMS and NMR analyses of **2** indicated that it is likely an intermediate *en route* to **1**, lacking the central tetrahydrofuran and instead bearing a methyl and ketone at C-13 and C-15, respectively (Table S8). The relative and absolute configurations of **1** and **2** were determined by NOESY/ROESY correlations, single-crystal X-ray diffraction analysis of **2**, and comparison of experimental and time-dependent DFT-calculated ECD spectra (Scheme 1, Figures S6–S7, Tables S7–S8). NMR and ECD analyses of **3**–**5** identified **3** as the 15-deoxo-2′,3′-diol derivative of **2**, **4** as a 3-hydroxymethyl-4-quinolone substituted by a (2*R*,3*R*)-epoxygeranylgeranyl group at C-2, and **5** as a pyranoquinolone substituted by a farnesyl group at C-4′ (Figures S8–S9, Tables S9–S11).

**Scheme 1:**
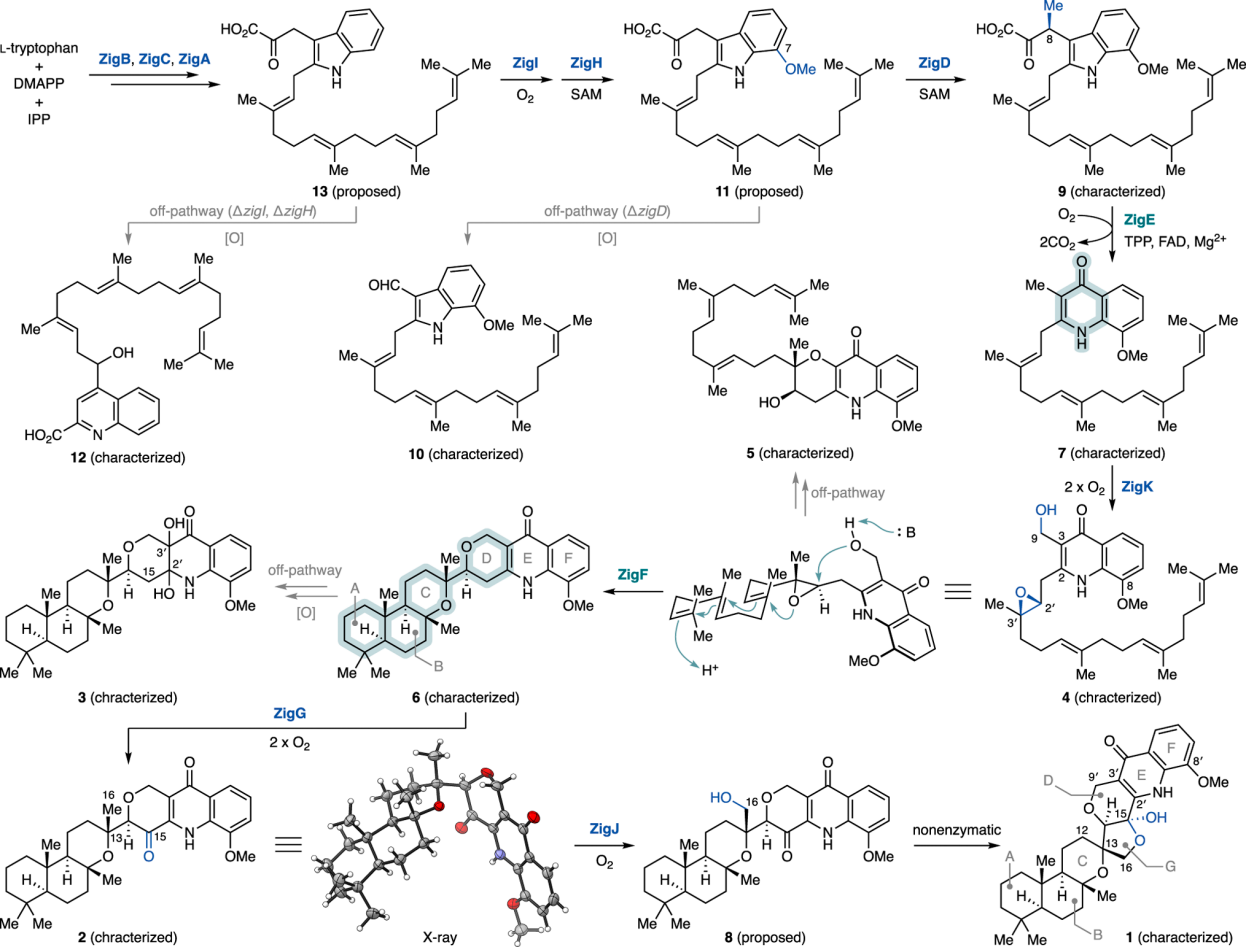
Proposed Biosynthetic Pathway of Zigralone A

Zigralones A–C (**1**–**3**) represent the first pyranoquinolone-based meroditerpenoids identified in nature. Structurally, **1** features an unprecedented heptacyclic architecture comprising a dihydropyranoquinolone chromophore fused to a dioxaspiro[4.5]­decane system, whereas **2** and **3** feature a bilobed architecture in which a pyranoquinolone moiety is connected to a manoyl oxide-like skeleton via a C­(sp^3^)–C­(sp^3^) bond. Antibacterial assays using ESKAPE pathogens^15^ revealed that **2** and **3** exhibit potent activity against *Staphylococcus aureus* DSM 2569 with minimum inhibitory concentrations of 2 μg/mL (Table S12).

We next sought to assign the roles of individual Zig enzymes. For this purpose, we constructed a series of single-gene deletion variants in the heterologous expression system, analyzed their metabolic profiles, and purified accumulating products for structure elucidation. Based on the structures of **1**–**5**, the biosynthetic steps from **4** to **1** were proposed to proceed via Pyr4-like TC-catalyzed cyclizations and P450-mediated oxidations, followed by hemiketalization (Scheme 1). We therefore first focused on the late-stage steps of zigralone biosynthesis.

Deletion of *zigG*, encoding a P450, abolished production of **1** and **2** while retaining production of **3**–**7** (Figure 3). Compound **6** was isolated and identified as a 15-deoxo analog of **2** (Table S13), indicating that ZigG catalyzes C-15 oxidation of **6** to form **2**, whereas **3** likely arises as an off-pathway product via oxidation at C-2′/C-3′. Deletion of *zigK*, encoding a P450, led to accumulation of **7**, an analog of **4** lacking the epoxide and hydroxy group (Table S14), suggesting that ZigK converts **7** into **4** via (*R*,*R*)-epoxidation at C-2′/C-3′ and hydroxylation at C-9. Additionally, deletion of *zigF*, encoding a Pyr4-like TC, abolished production of the cyclic compounds (**1**–**3**, and **6**), indicating that ZigF is essential for polyene cyclization. To further validate the function of ZigF, we expressed *zigF* in *Escherichia coli* Rosetta­(DE3)­pLysS and prepared cell-free extracts for *in vitro* assays. Conversion of **4** into **6** was observed only in reactions using the cell-free extract containing ZigF (Figure S10), supporting the role of ZigF in catalyzing the cyclization of **4** to yield **6**.

Epoxidation of prenyl chains that prime TC-mediated scaffold formation is typically catalyzed by flavin-dependent monooxygenases (FMOs),^16^ with a single exception, the P450 AscE in ascochlorin/ascofulanone biosynthesis.^17^ In contrast to previously characterized meroterpenoid epoxidases that introduce epoxides at terminal or distal olefins of oligoprenyl chains,^16^ ZigK installs the epoxide proximal to the nonterpenoid core, which is essential for subsequent cyclization. Moreover, ZigF catalyzes the formation of both a *trans*-decalin (fused ring) and a 2,2′-bihydropyran (attached-ring). The latter bis-oxacyclic motif is rarely generated through TC-catalyzed cyclizations and instead resembles structural features formed by epoxide hydrolase-mediated transformations in polyether polyketide biosynthesis.^18^ Mechanistically, the ZigF-mediated tandem carbo/oxacyclizations are proposed to be initiated by protonation of the terminal alkene in **4**, generating transient oxonium species,^19^ followed by 6-*exo*-tet cyclization to yield **6** (Figure S11).

Deletion of *zigJ*, encoding a P450, abolished production of **1** and led to the accumulation of **2** (Figure 3). We propose that ZigJ catalyzes C-16 hydroxylation of **2** to yield **8**, which undergoes nonenzymatic hemiketalization to form **1** (Scheme 1). DFT calculations support that the *Re*-face intramolecular addition of OH-16 to the C-15 ketone in **8** proceeds under ambient conditions (Figure S12). In contrast, deletion of genes encoding the remaining P450s (ZigL, ZigN) and Fdx (ZigM) caused no detectable changes in metabolite profiles, either in single- or double-gene deletion mutants (Figure 3), indicating that these enzymes are not required for the biosynthesis of **1**.

We next investigated the early stage pathway leading to the formation of the quinolone **7**. Known quinolone/quinoline biosynthetic routes typically involve thiotemplate-dependent enzymes or FMOs (Figure S13),^20^ neither of which is encoded in the *zig* BGC. We therefore focused on the functionally unassigned TPP-dependent enzyme ZigE, comprising two subunits with low homology to the α-hydroxy-β-keto acid synthase CsmA^21^ and the pyruvate oxidase PoxB,^22^ respectively (Table S3). Deletion of *zigE* abolished the production of **1**–**7** and led to the accumulation of an (*S*)-β-methyl-indole-3-pyruvate derivative (**9**) and an indole-3-carbaxaldehyde derivative (**10**) (Figures 3, S14, Tables S15–S16). Additionally, the Δ*zigD* (MT gene) mutant resulted in the accumulation of **10**. These results indicate that ZigD catalyzes (*S*)-methylation of the indole-3-pyruvate derivative **11** to form **9**, which is subsequently converted to **7** by ZigE (Scheme 1). Compound **10** is likely a shunt product arising from oxidative cleavage of the α-keto acid of **11**. Feeding of **9** to *S. albus* expressing *zigE* resulted in its biotransformation to **7** (Figure S15), confirming **9** as a substrate of ZigE. Notably, ZigE catalyzes an unconventional indole ring expansion that entails C-2/C-3 double bond cleavage, C–C bond formation at C-2/C-8, and tandem decarboxylation of the α-keto acid. We speculate that ZigE catalyzes a Witkop–Winterfeldt-like oxidation^23^ that proceeds via oxidative indole ring cleavage and intramolecular aldol condensation (Figure S16). The mechanism of this transformation is currently under investigation.

Deletion of *zigH* (MT gene) or *zigI* (DUF6875 gene) resulted in the accumulation of the demethoxylated quinaldic acid **12** (Figure 3, Table S17). DUF6875-containing proteins were recently identified as heme-binding proteins capable of catalyzing l-tryptophan oxidations.^24^ Multiple sequence alignments revealed that ZigI has the conserved heme-binding motif found in l-tryptophan C-7 hydroxylases (Figure S17). We expected that ZigI catalyzes C-7 hydroxylation of **13**, followed by ZigH-mediated methoxylation to generate **9** (Scheme 1). In the absence of *zigH* or *zigI*, **13** likely undergoes oxidative rearrangement leading to **12**.

The remaining enzymes ZigA (aminotransferase), ZigB (polyprenyl synthase), and ZigC (PT) are proposed to assemble the indole intermediate **13** from l-tryptophan, dimethylallyl pyrophosphate, and three molecules of isopentenyl pyrophosphate (Scheme 1). Deletion of *zigC* abolished the production of *zig*-associated metabolites, whereas the Δ*zigA* mutant still produced **1**–**7** (Figure 3), indicating that the ZigA-catalyzed transamination can be complemented by endogenous transaminases.^25^

With the zigralone biosynthetic model, we envisioned that an 8-demethoxy analog of **4** may serve as a precursor to the recently identified goondolinone A whose biosynthetic gene cluster remains elusive.^26^ Its distinct *trans*-bisoxepane system could arise from an alternative epoxide-opening pathway during cyclization (Figure S18). We identified a *zig*-like BGC in the genome of *Actinomadura yumaensis* (Figure S3), a bacterium closely related to the goondolinone A producer.^26^ The ZigF homologue may catalyze an analogous cyclization cascade, although the mechanistic determinants governing pathway branching remain unclear.

In summary, we discovered a family of pyranoquinolone diterpenoids and elucidated the genetic basis of their biosynthesis. The complex carbo/heterocyclic framework arises from the unparalleled sequence of an oxidative indole rearrangement catalyzed by a TPP-dependent enzyme, Pyr4-like TC-catalyzed atypical tandem carbo/oxacyclizations, P450-mediated oxidations, and putative nonenzymatic hemiketalization. These findings expand the characterized functions of Pyr4-like TCs and highlight bacterial meroterpenoid pathways as a source of previously unexpected chemical complexity and enzymatic repertoires.

## Supplementary Material



## Notes

Materials and methods, experimental procedures, structure characterization details of isolated compounds, bioinformatic data, LC-MS chromatograms, X-ray structure data, copies of NMR spectra, Cartesian coordinates of DFT-optimized structures (PDF)
