# Supplementary material for: Discovery of Pyranoquinolone Diterpenoids Biosynthesized through Oxidative Indole Rearrangement and Atypical Terpene Cyclization
Source: J Am Chem Soc. 2026 Jun 10;148(24):24603–8. doi: 10.1021/jacs.6c07454 (PMC13307353; doi:10.1021/jacs.6c07454)
Supplement: Supplementary file 1 [file ja6c07454_si_001.pdf]

Supporting Information

Discovery of Pyranoquinolone Diterpenoids Biosynthesized through Oxidative Indole Rearrangement and Atypical Terpene Cyclization

Yuya Kakumu† and Eric J. N. Helfrich*,†,‡

†Institute of Molecular Biosciences, Goethe University Frankfurt, Max-von-Laue Strasse 9, Frankfurt am Main 60438, Germany.

‡Senckenberg Society for Nature Research, Senckenberganlage 25, Frankfurt am Main 60325, Germany.

*To whom correspondence should be addressed: eric.helfrich@bio.uni-frankfurt.de

Table of Contents

1. Supplementary Figures S2
2. Supplementary Tables S19
3. Experimental Procedures S36
4. References S51
5. Single-Crystal X-ray Diffraction Data S54
6. Copies of NMR Spectroscopic Data S55
7. Cartesian Coordinates of DFT-Optimized Structures S121

1. Supplementary Figures

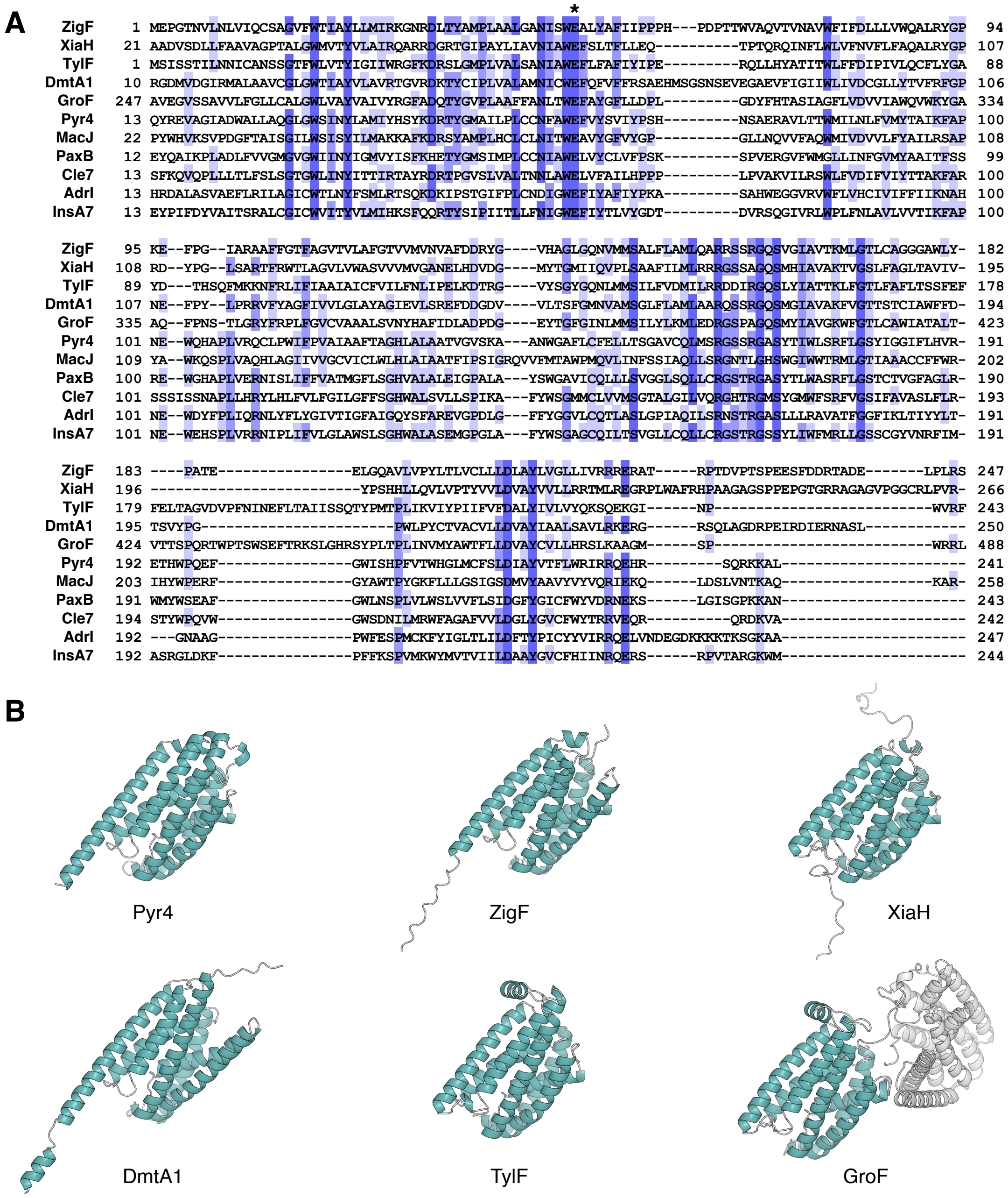

Figure S1. Bioinformatic analysis of bacterial Pyr4-like TCs. (A) Multiple sequence alignment of selected known Pyr4-like TCs from bacterial (ZigF, XiaH, TylF, DmtA1, GroF) and fungal origins (Pyr4, MacJ, PaxB, Cle7, AdrI, InaA7). Residues are colored according to percent identity in each column, with darker blue indicating higher conservation. Conserved glutamic acid residues proposed to function as the catalytic acid are marked with an asterisk. In AdrI, Asp59 acts as the Brønsted acid instead of the conserved glutamic acid. (B) Predicted structures of bacterial Pyr4-like TCs compared with that of Pyr4. In GroF, an additional domain is shown in gray. Consistent with Pyr4, all predicted bacterial Pyr4-like TC structures comprise seven α-helices arranged in a similar architecture.

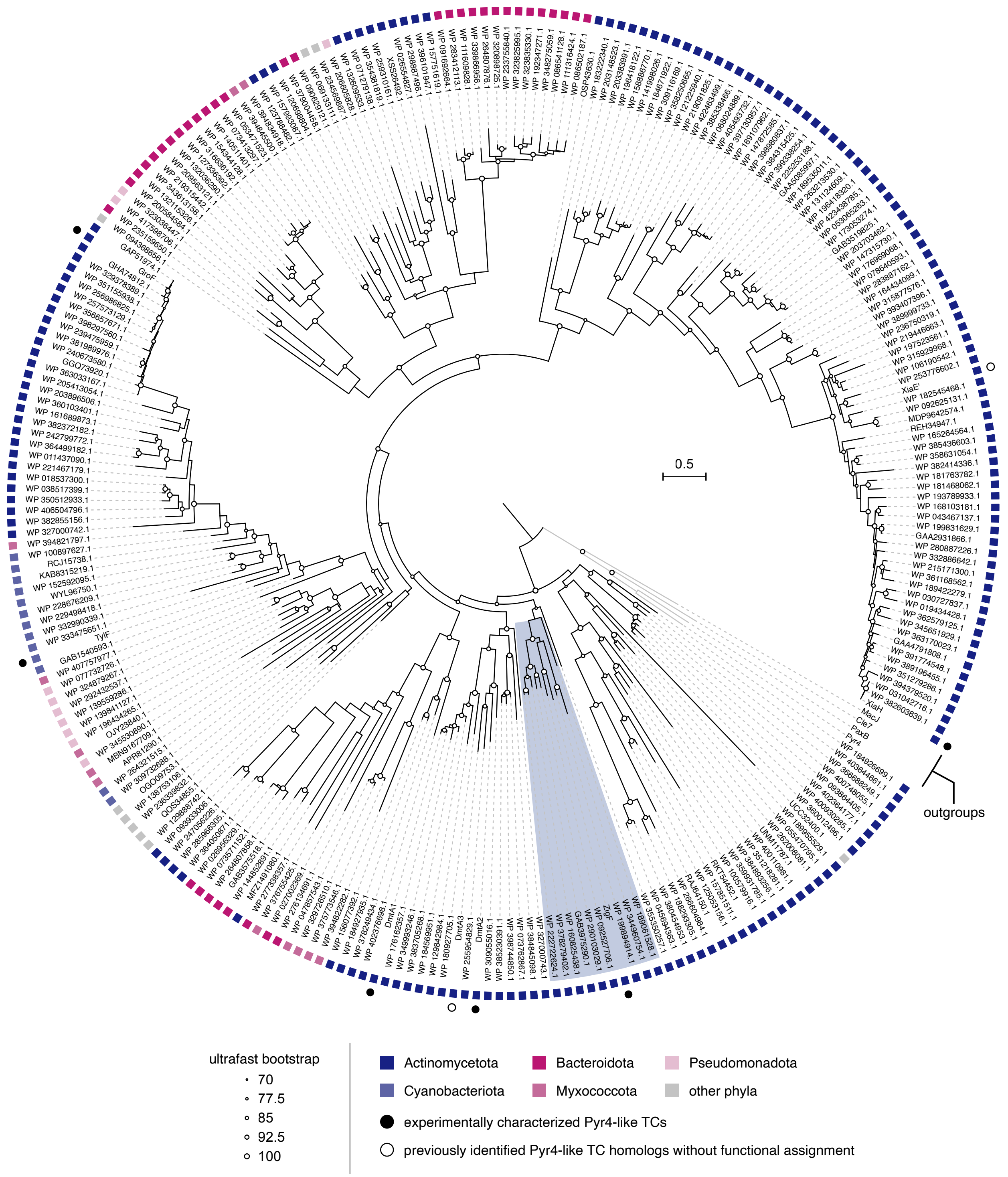

Figure S2. Maximum likelihood phylogenetic tree of 264 bacterial Pyr4-like TCs. Four known fungal Pyr4-like TCs (Pyr4, PaxB, MacJ, Cle7) were used as outgroups and the corresponding subclade is shown in gray. Branch support was assessed using ultrafast bootstrap analysis with 10000 replicates. Nodes with bootstrap values ≥ 70 are indicated by circles, with circle size corresponding to the bootstrap values. The subclade highlighted in blue contains ZigF and its homologs, which clustered together in the SSN at an *e*-value of 10^{-60}.

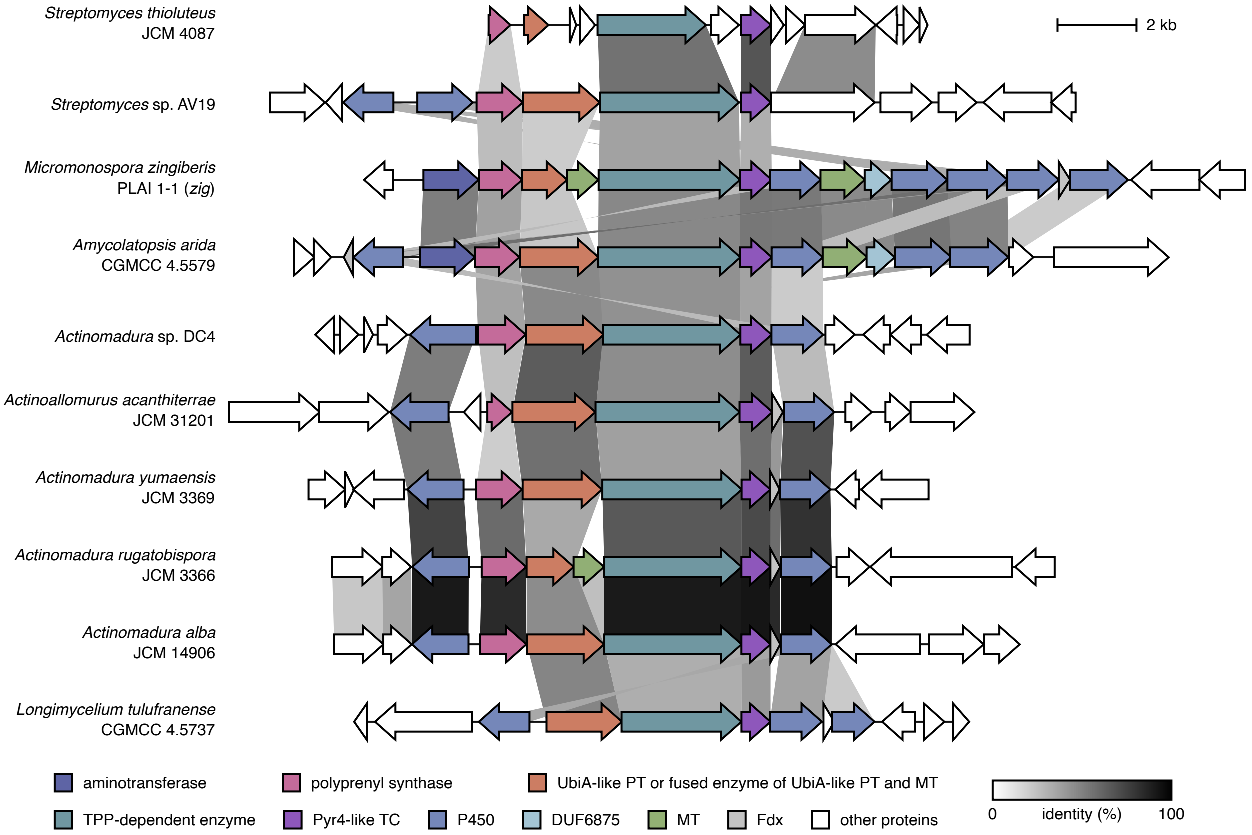

Figure S3. Synteny analysis of the *zig* BGC and homologous BGCs. Genes encoding proteins with more than 30% sequence identity are connected.

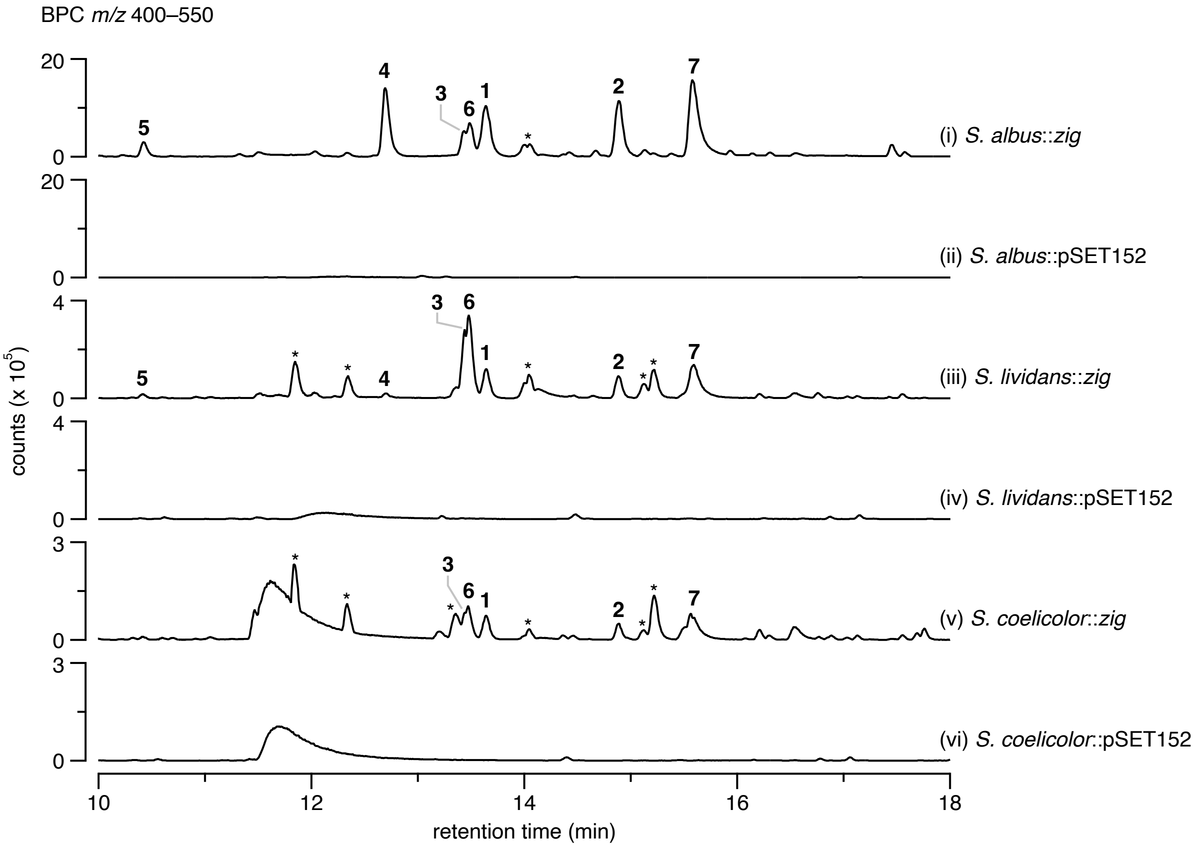

Figure S4. LC-MS metabolic profiles of *Streptomyces* strains expressing the *zig* BGC. (i) *S. albus* harboring the *zig* BGC; (ii) *S. albus* harboring the empty pSET152-SP44-SR41 vector; (iii) *S. lividans* harboring the *zig* BGC; (iv) *S. lividans* harboring the empty pSET152-SP44-SR41 vector; (v) *S. coelicolor* harboring the *zig* BGC; and (vi) *S. coelicolor* harboring the empty pSET152-SP44-SR41 vector. Base peak chromatograms (BPCs) were traced at *m/z* 400–550. Asterisks denote peaks of unidentified products.

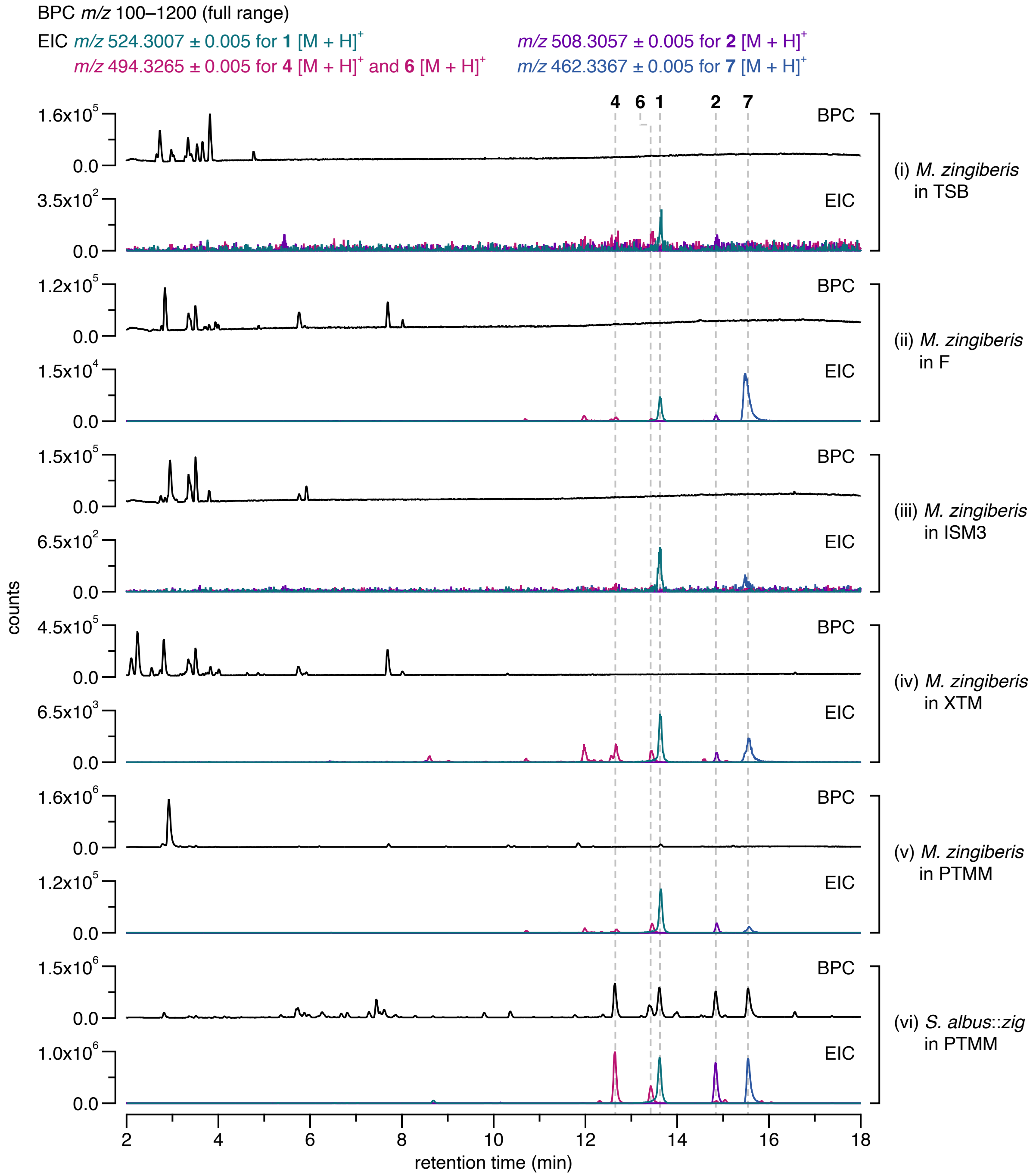

Figure S5. LC-MS metabolic profiles of *M. zingiberis* cultured in different media. Cultures were grown in TSB (i), F (ii), ISM3 (iii), XTM (iv), and PTMM (v). The culture extract of *S. albus* expressing the *zig* BGC grown in PTMM is shown as a reference (vi). BPCs were monitored over a mass range from *m/z* 100–1200. Extracted ion chromatograms (EICs) were traced at *m/z* 524.3007 (±5 mmu) for **1** $[M + H]^+$, *m/z* 508.3057 (±5 mmu) for **2** $[M + H]^+$, *m/z* 494.3265 (±5 mmu) for **4** $[M + H]^+$ and **6** $[M + H]^+$, and *m/z* 462.3367 (±5 mmu) for **7** $[M + H]^+$. Metabolites derived from the *zig* BGC were detected in culture extracts of the native producer, albeit at substantially lower titers than in extracts of *S. albus*::*zig*.

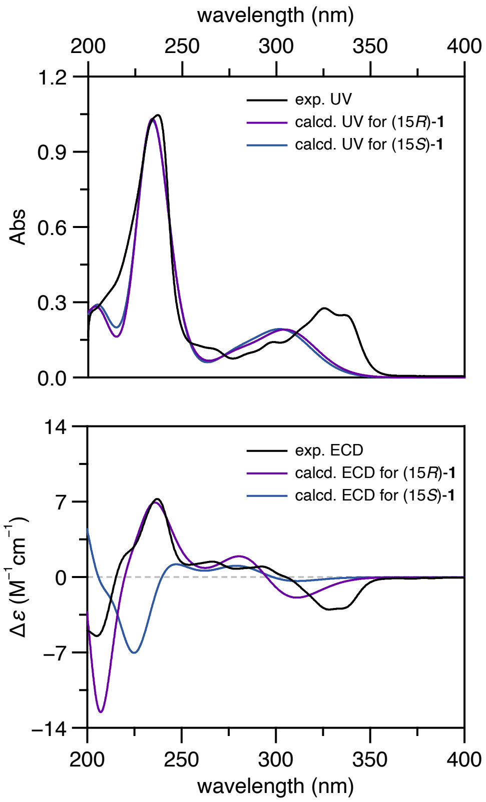

Figure S6. Comparison of experimental and calculated UV and ECD spectra for **1**. Experimental UV and ECD spectra of **1** were recorded in MeOH. Boltzmann-weighted UV and ECD spectra for (5*S*,8*R*,9*R*,10*S*,13*S*,14*R*,15*R*)-**1** and (5*S*,8*R*,9*R*,10*S*,13*S*,14*R*,15*S*)-**1** were calculated at TD-ωB97X-D/aug-cc-pVTZ/SMD(MeOH)//M06-2X/def2-TZVP/SMD(MeOH).

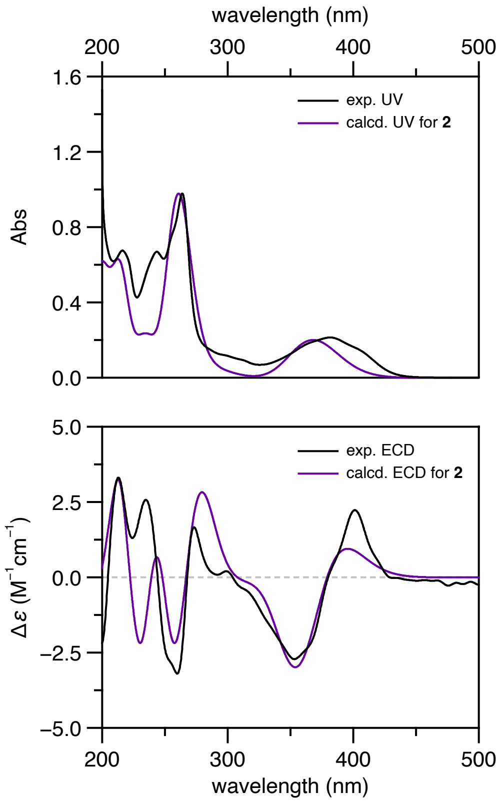

Figure S7. Comparison of experimental and calculated UV and ECD spectra for **2**. Experimental UV and ECD spectra of **2** were recorded in MeOH. Boltzmann-weighted UV and ECD spectra for (5*S*,8*R*,9*R*,10*S*,13*S*,14*R*)-**2** were calculated at TD-*ω*B97X-D/aug-cc-pVTZ/CPCM(MeOH)//PBE0/def2-TZVP/CPCM(MeOH).

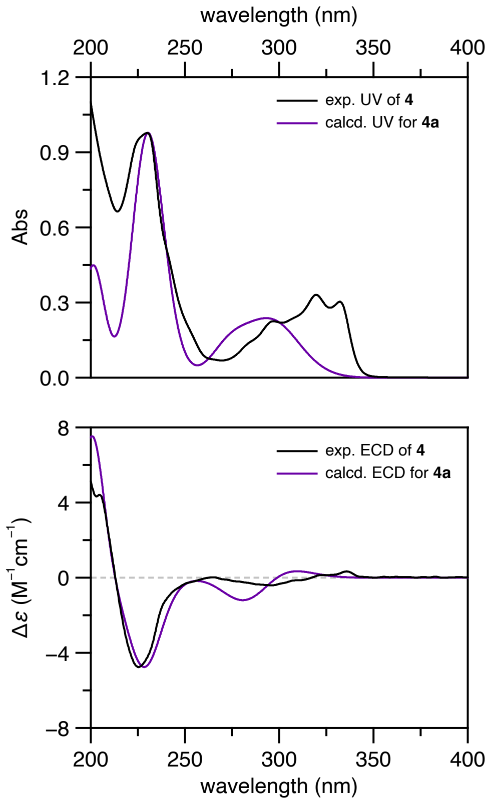

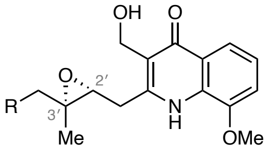

4 R = farnesyl
4a R = Me

Figure S8. Comparison of experimental and calculated UV and ECD spectra for **4**. Experimental UV and ECD spectra of **4** were recorded in MeCN. Boltzmann-weighted UV and ECD spectra for (2′*R*,3′*R*)-**4a** were calculated at TD-ωB97X-D/def2-TZVPP/SMD(MeCN)//M06-2X/def2-SVP.

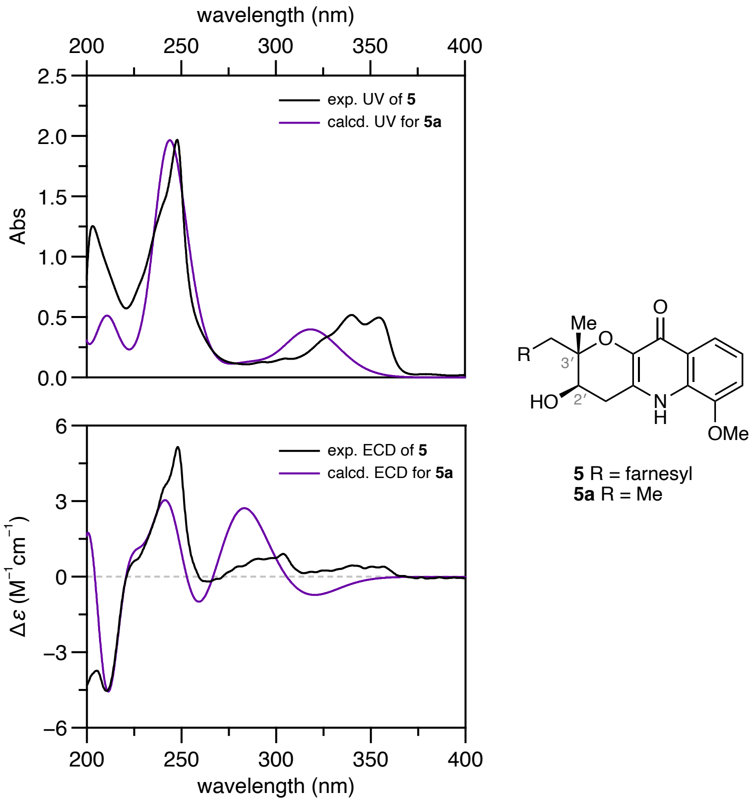

Figure S9. Comparison of experimental and calculated UV and ECD spectra for **5**. Experimental UV and ECD spectra of **5** were recorded in MeOH. Boltzmann-weighted UV and ECD spectra for (2′*R*,3′*S*)-**4a** were calculated at TD-*ω*B97X-D/def2-TZVPP/SMD(MeOH)//M06-2X/def2-TZVP/SMD(MeOH).

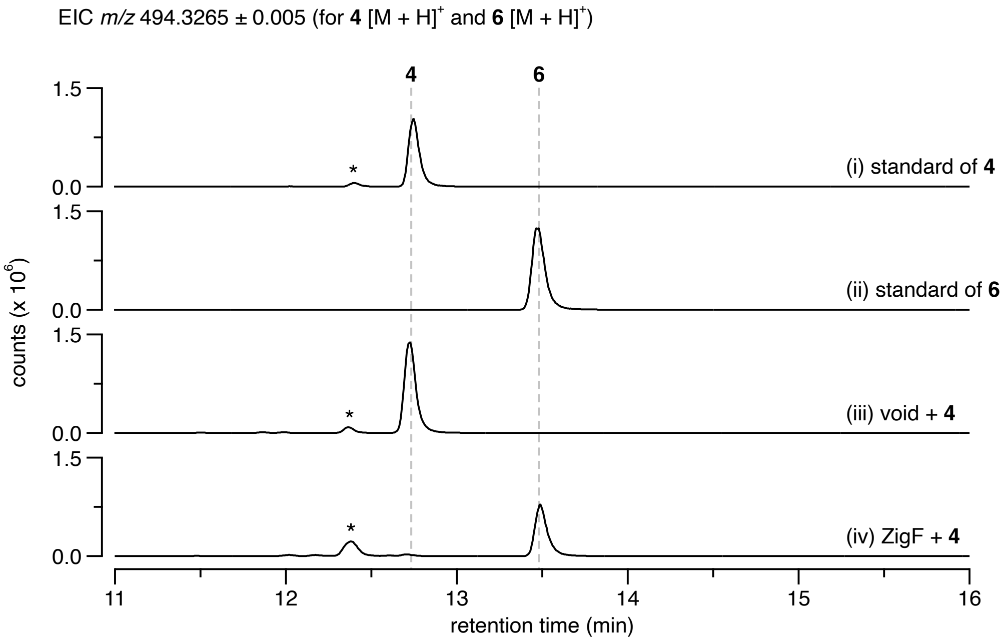

Figure S10. LC-MS analysis of the *in vitro* reaction of ZigF with **4**. (i) Standard of **4**; (ii) standard of **6**; (iii) cell-free extract of *E. coli* Rosetta(DE3)pLysS harboring an empty vector incubated with **4**; and (iv) cell-free extract of *E. coli* Rosetta(DE3)pLysS expressing ZigF incubated with **4**. EICs were traced at *m/z* 494.3265 (±5 mmu) for **4** $[M + H]^+$ and **6** $[M + H]^+$. Asterisks denote peaks corresponding to a putative isomer of **4** that was not structurally characterized.

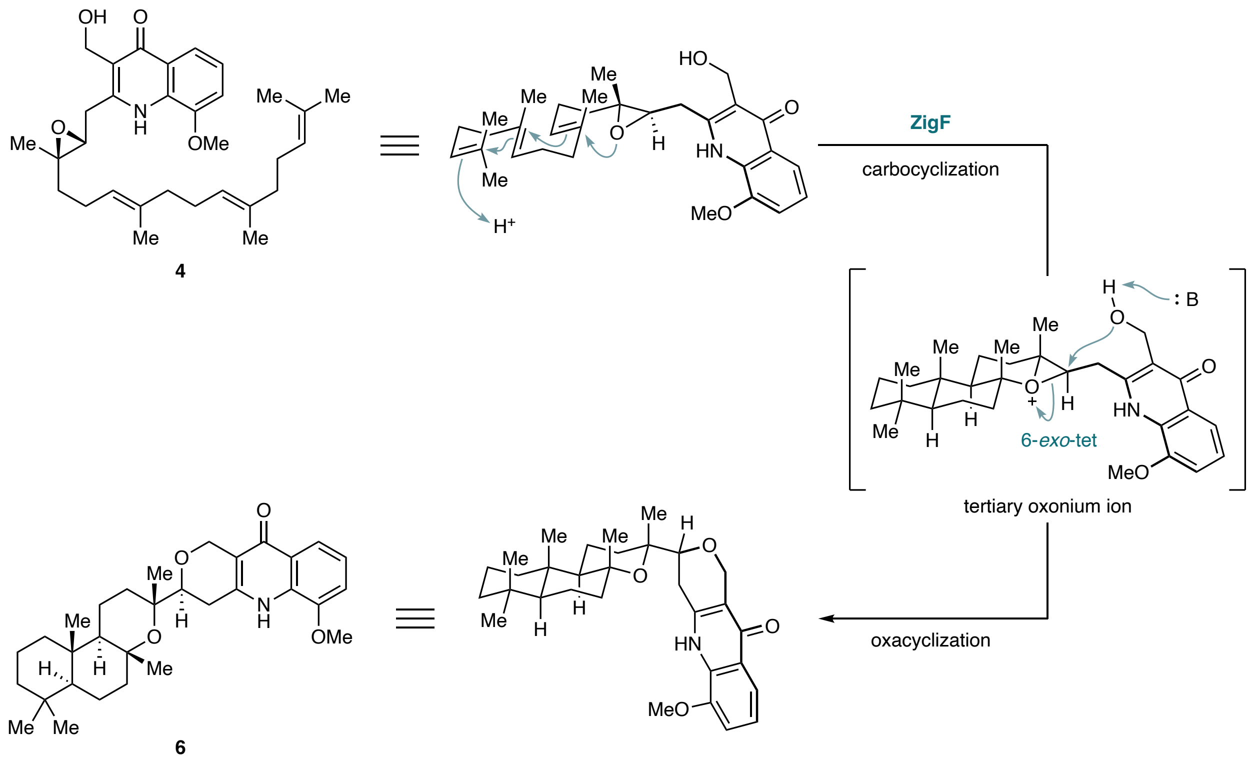

Figure S11. Proposed mechanism of tandem carbo- and oxacyclizations catalyzed by the Pyr4-like TC ZigF.

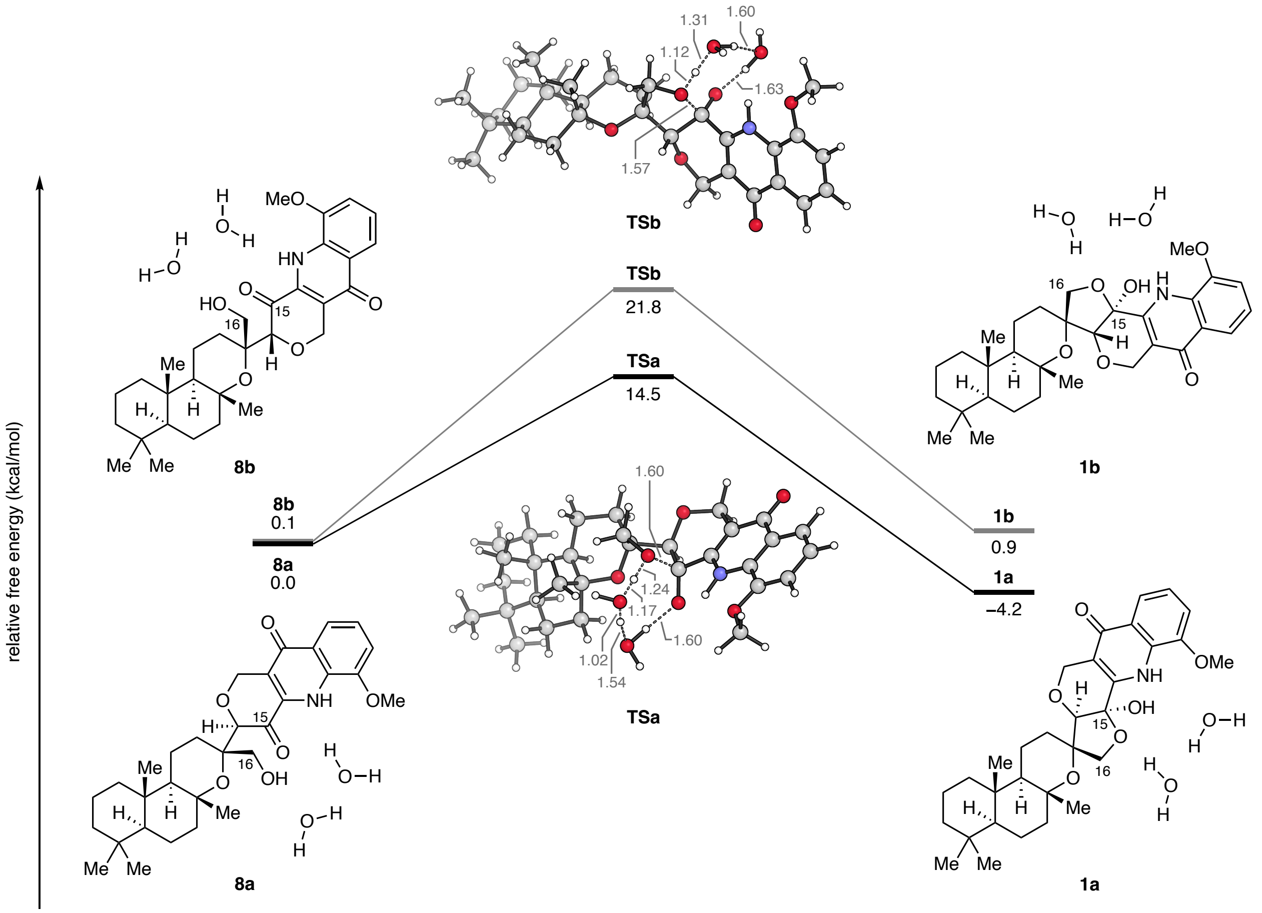

Figure S12. Computed energy diagram for the putative nonenzymatic hemiketalization of **8** to form **1**. Models incorporating two bridging water molecules to assist proton transfer were constructed for nucleophilic addition of OH-16 to the C-15 ketone via the *Re*-face (**8a**–**TSa**–**1a**) or *Si*-face (**8b**–**TSb**–**1b**). In the optimized structures of transition states (**TSa** and **TSb**), key bond distances associated with proton shuttling and bond formation are shown in Å. Calculations was performed at the *ω*B97X-D/def2-TZVPP/SMD(H_2O)//*ω*B97X-D/def2-SVP/SMD(H_2O) level of theory under standard state (298.15 K, 1 atm, 1 M). The conversion of **8a** to **1a** is exergonic, and the activation barrier ($\Delta G^{\ddagger}$ = 14.5 kcal/mol) indicates that a reaction can proceed under ambient conditions. The large substantial difference in activation energies ($\Delta\Delta G^{\ddagger}$ = 7.3 kcal/mol) accounts for the dominant stereoselectivity of the addition reaction, as the (15*S*)-**1** isomer was not detected in the culture extract of *S. albus*::*zig*.

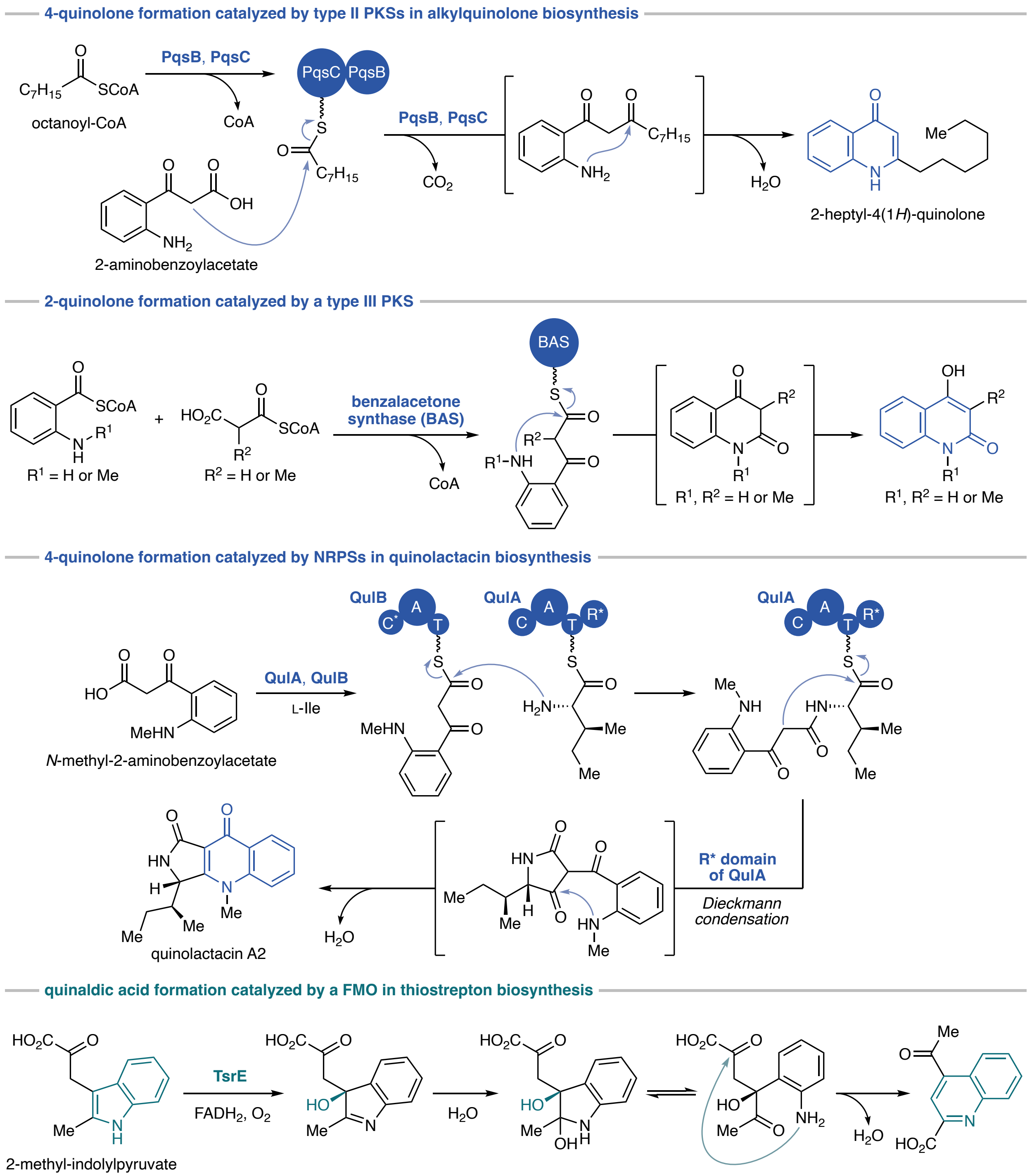

Figure S13. Examples of known quinolone and quinoline biosynthetic pathways involving type II polyketide synthases (PKSs), type III PKSs, nonribosomal peptide synthases (NRPSs), and flavin-dependent monooxygenases (FMOs). Abbreviations: A, adenylation domain; C, condensation domain; C*, truncated condensation domain; R*, a domain responsible for Dieckmann condensation; T, thiolation domain.

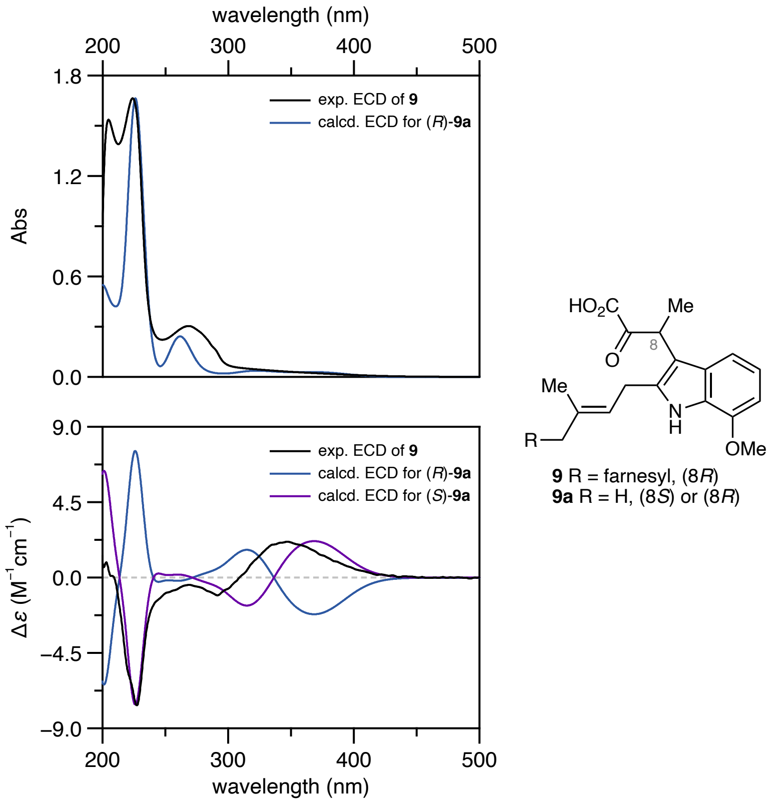

Figure S14. Comparison of experimental and calculated UV and ECD spectra for **9**. Experimental UV and ECD spectra of **9** were recorded in MeOH. Boltzmann-weighted UV and ECD spectra for (*R*)-**9a** were calculated at TD-*ω*B97X-D/def2-TZVPP/SMD(MeOH)//M06-2X/def2-TZVP/SMD(MeOH).

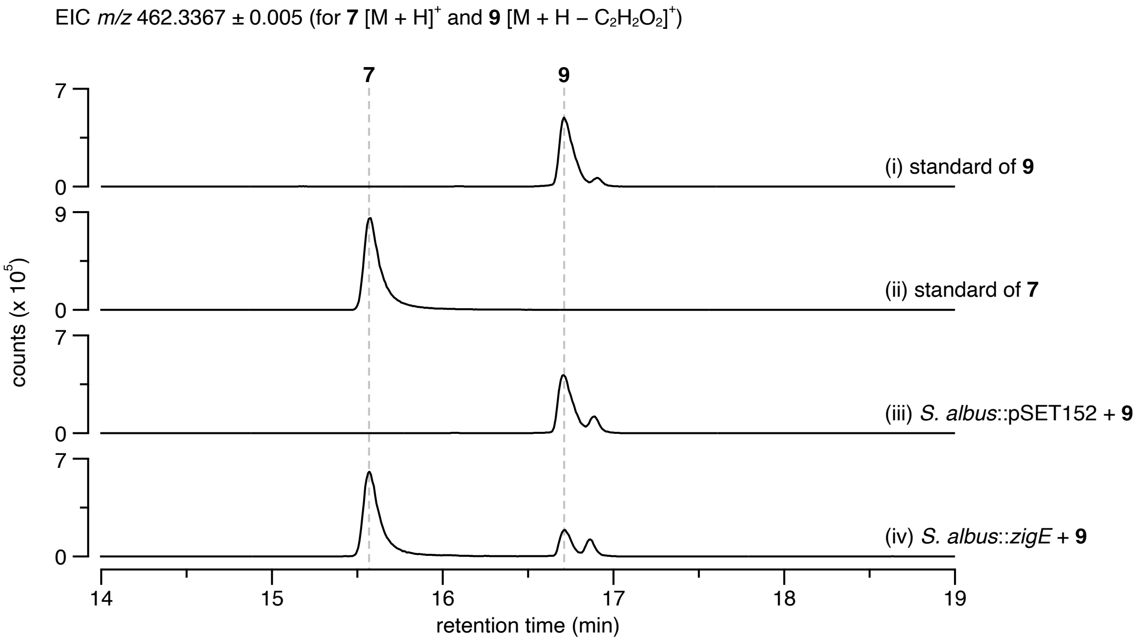

Figure S15. LC-MS analysis of the biotransformation of **9** in *S. albus* expressing *zigE*. (i) Standard of **9**; (ii) standard of **7**; (iii) *S. albus* harboring an empty vector supplemented with **9** for 24 h; and (iv) *S. albus* expressing *zigE* supplemented with **9** for 24 h. EICs were traced at *m/z* 462.3367 (±5 mmu) for **7** $[M + H]^+$ and **9** $[M + H - C_2H_2O_2]^+$. For **9**, the in-source fragment ion $[M + H - C_2H_2O_2]^+$ was used for EICs as it was the most intense ion among the observed ion species in positive ion mode.

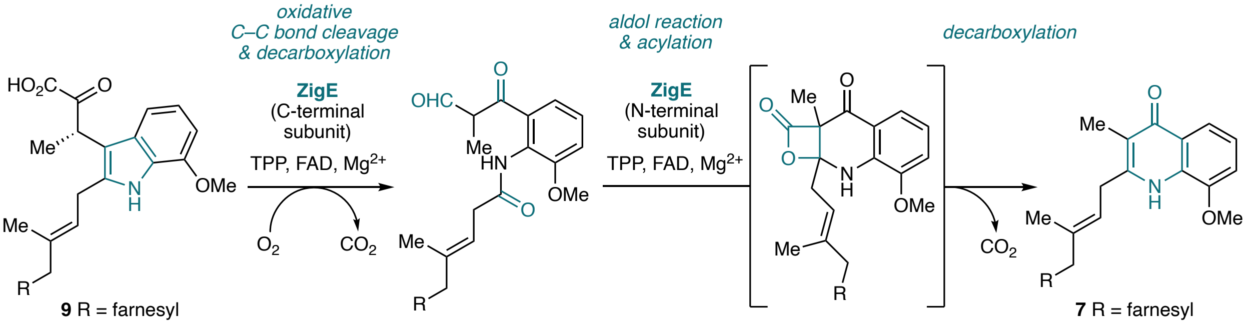

Figure S16. Proposed Witkop–Winterfeldt-like oxidation catalyzed by ZigE. The origin of the carbonyl oxygen in the quinolone moiety remains unresolved. Molecular oxygen is proposed as the source during the initial oxidative C–C bond cleavage and decarboxylation, but this hypothesis requires further experimental validation.

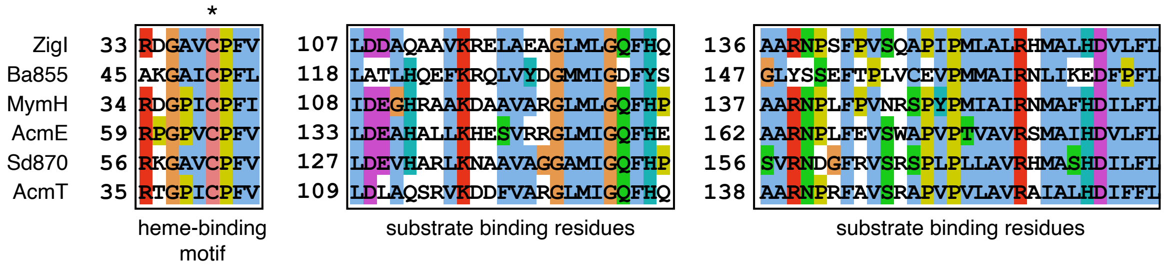

Figure S17. Multiple sequence alignment of ZigI and biochemically characterized DUF6875-containing tryptophan 7-hydroxylases, Ba855 (WP_061573388.1, *Bacillus atrophaeus*), MymH (AVR52611.1, *Streptomyces olivaceus*), AcmE (ADG27360.1, *Streptomyces anulatus*), Sd870 (WP_015660585.1, *Streptomyces davaonensis*), and AcmT (ADG27353.1, *Streptomyces anulatus*). The conserved heme-binding motif and substrate-binding residues are shown. The cysteine residue predicted to bind a heme iron was indicated by an asterisk.

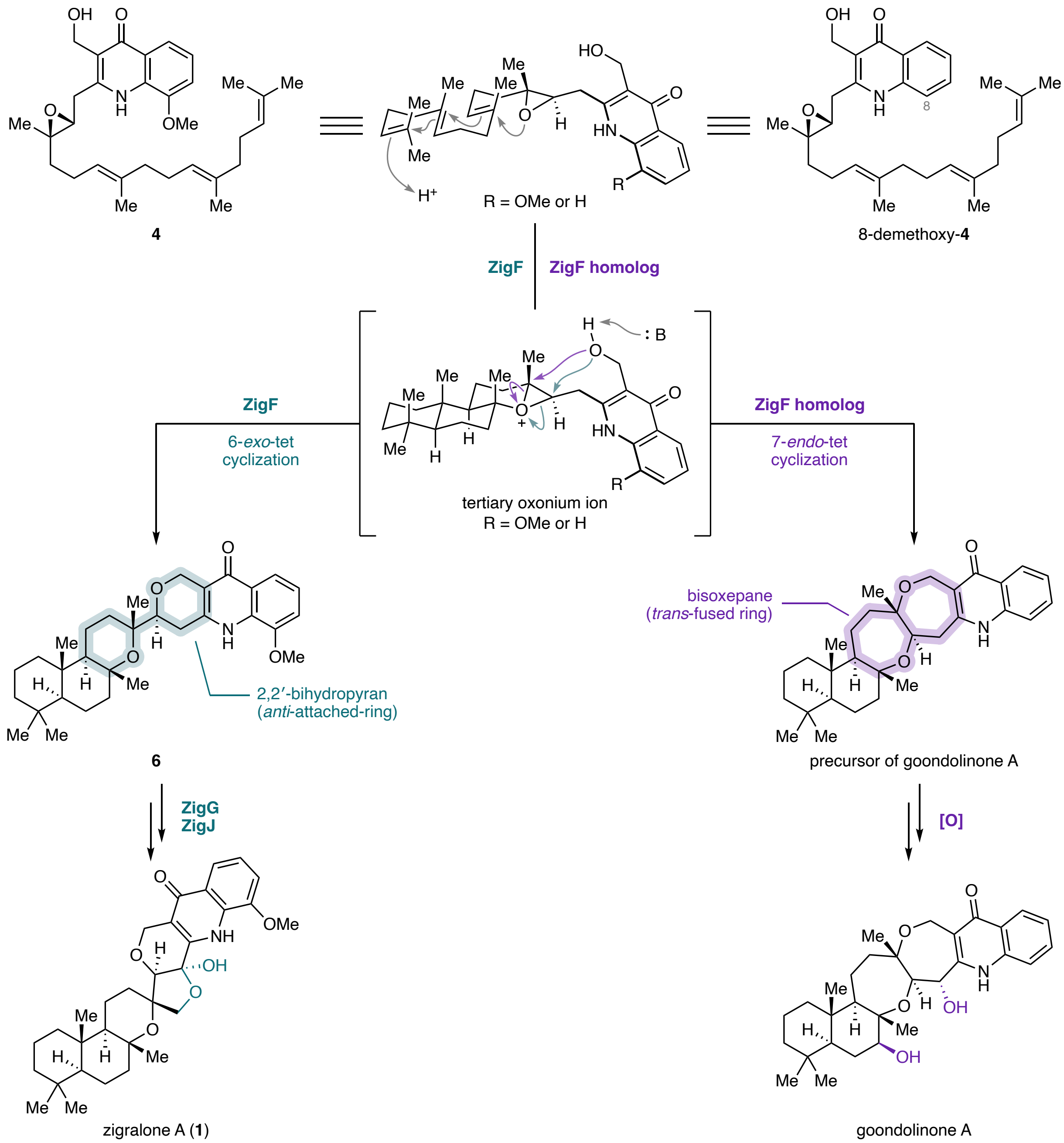

Figure S18. Proposed pathway branching in the biosynthesis of zigralone A and goondolinone A via terpene cyclization catalyzed by ZigF and its homolog in the goondolinone pathway. A tertiary oxonium ion formed during cyclization is proposed to serve as a branching point that undergoes either 6-*exo*-tet cyclization (zigranone pathway) or 7-*endo*-tet cyclization (goondolinone A pathway), resulting in skeletally distinct oxacyclic ring systems.

2. Supplementary Tables

Table S1 Known Pyr4-like TCs used for pHMM construction in this study

name	origin	accession no.	name	origin	accession no.
Pyr4	*Aspergillus fumigatus* Af293	XP_751270.1	NodB	*Hypoxylon pulicicidum* MF5954	AUM60064.1
AusL	*Aspergillus nidulans* FGSC A4	EAA66324.1	AtmB	*Aspergillus flavus* NRRL 6541	CAP53939.1
Trt1	*Aspergillus terreus* NIH2624)	EAU29526.1	IdtB	*Claviceps paspali* RRC-1481	AFO85421.1
AdrI	*Penicillium rubens* Wisconsin 54-1255	B6HV37.2	LtmB	*Epichloe festucae* Fl1	AFO85410.1
AndB	*Emericella variecolor* NBRC 32302	BAP81856.1	TerB	*Tolypocladium album*	BAM84047.1
AdrI′	*Emericella variecolor* NBRC 32302	BAW27604.1	NomB	*Aspergillus nomiae* NRRL 13137	XP_015406232.1
PrhH	*Penicillium brasilianum* NBRC 6234	BAV69309.1	SpdB	*Cordana terrestris* FKA-25	BBD84644.1
AscF	*Acremonium egyptiacum* F-1392	BBF25318.1	AstH	*Penicillium arizonense* CBS 141311	OGE52342.1
NvfL	*Aspergillus novofumigatus* IBT 16806	XP_024677071.1	EsdpB	*Penicillium shearii* IFM 42152	BCP96886.1
CdmG	*Talaromyces verruculosus* TPU1311	BBG28486.1	EstB1	*Aspergillus striatus* ATCC 64988	WGJ63576.1
Cle7	*Aspergillus versicolor* 0312	BBG28477.1	EstB2	*Aspergillus striatus* ATCC 64988	WGJ63583.1
Sre3	*Aspergillus felis* 0260	BBG67005.1	HomoB	*Aspergillus homomorphus* CBS 101889	XP_025551548.1
SptB	*Aspergillus* sp. TJ23	BCD52380.1	FumiB	*Aspergillus fumigatus* A1163	EDP47980.1
FncH	*Aspergillus funiculosus* CBS 116.56	JGI_210966	AlliB	*Aspergillus alliaceus* CBS 536.65	XP_031902718.1
SetH	*Aspergillus duricaulis* CBS 481.65	JGI_270779	SpyD	*Aspergillus fumigatus* Af293	EAL84929.1
OlcD′	*Aspergillus felis* 0260	BDD69374.1	MfmH	*Annulohypoxylon moriforme* CBS 123579	KAI1452419.1
InsA7	*Aspergillus insuetus* CBS 107.25	KAL3443341.1	OcdTC	*Colletotrichum orchidophilum* IMI 309357	XP_022481694.1
InsB4	*Aspergillus insuetus* CBS 107.25	KAL3439813.1	MosB	*Neoarthrinium moseri* CBS 164.80	XP_049167926.1
BrvF	*Penicillium bialowiezense* CBS 227.28	BDO47156.1	DesB	*Aspergillus desertorum* CBS 653.73	DBA45434.1
PaxB	*Penicillium paxilli* PN2013	ADO29934.1	TlxF	*Talaromyces purpureogenus* CX11	BDC03468.1
MacJ	*Penicillium terrestre* LM2	QBC75443.1	ParJ	*Paraphaeosphaeria* sp. C-XB-J-1	XPR29134.1
AceB	*Aspergillus alliaceus* ATCC 20738	UOH28368.1	AtS5B1	*Aspergillus tubingensis* CBS 134.48	OJI82697.1
AtlC	*Penicillium chrysogenum* MT-40	UOK93803.1	AfB	*Aspergillus flavus* NRRL3357	KAF7622179.1
IdtB	*Epichloe* sp. LpTG-3 AR37	UUW39079.1	AtS2B	*Aspergillus tubingensis* CBS 134.48	OJI86757.1
DpfgB	*Fusarium graminearum* PH-1	P9WEY0.1	AtoK	*Aspergillus ochraceus* LZDX-32-15	XIT78377.1
DpchB	*Colletotrichum higginsianum* IMI 349063	OBR09784.1	XiaE	*Streptomyces* sp. HKI0576	CCH63731.1
DpmaB	*Metarhizium anisopliae* E6	KFG81920.1	XiaE′[a]	*Amycolatopsis nigrescens* CSC17Ta-90	WP_020673281.1
DpmpB	*Macrophomina phaseolina* MS6	EKG13729.1	TylF	*Nostocales cyanobacterium* HT-58-2	ARV57256.1
OlcD	*Penicillium canescens* ATCC 10419	P9WEQ3.1	DmtA1	*Streptomyces youssoufiensis* OUC6819	AVP32200.1
JanB	*Penicillium janthinellum* PN2408	AGZ20474.1	DmtA2	*Streptomyces* sp. NRRL F-5123	WP_052397357.1
PtmB	*Penicillium simplicissimum* AK-40	BAU61559.1	DmtA3[a]	*Streptomyces aidingensis* CGMCC 4.5739	SFD40844.1

[a]Homologs of XiaE or DmtA1/DmtA2, for which the associated natural products have not yet been characterized.

Table S2 Taxonomic distribution of bacterial Pyr4-like TCs identified in this study

phylum	no. of TCs	family	no. of TCs
Actinomycetota	183	Actinopolysporaceae	2
		Cellulomonadaceae	1
		Dermatophilaceae	1
		Frankiaceae	1
		Ilumatobacteraceae	1
		Jiangellaceae	1
		Microbacteriaceae	1
		Micrococcaceae	2
		Micromonosporaceae	22
		Nocardiaceae	4
		Nocardiopsidaceae	1
		Ornithinimicrobiaceae	1
		Promicromonosporaceae	1
		Pseudonocardiaceae	29
		Streptomycetaceae	109
		Streptosporangiaceae	1
		Thermomonosporaceae	5
Bacillota	4	Listeriaceae	1
		Paenibacillaceae	2
		Peptostreptococcaceae	1
Bacteroidota	37	Bernardetiaceae	1
		Chitinophagaceae	1
		Cyclobacteriaceae	17
		Flavobacteriaceae	10
		Hymenobacteraceae	2
		Sphingobacteriaceae	3
		Spirosomataceae	3
Balneolota	1	Balneolaceae	1
Chloroflexota	2	Ktedonosporobacteraceae	1
		unclassified	1
Cyanobacteriota	13	Chamaesiphonaceae	1
		Coleofasciculaceae	1
		Gloeotrichiaceae	1
		Nostocaceae	5
		Scytonemataceae	2
		Tolypothrichaceae	1
		unclassified	2
Ignavibacteriota	1	unclassified	1
Myxococcota	12	Archangiaceae	5
		Pendulisporaceae	4
		Polyangiaceae	1
		unclassified	2
Planctomycetota	1	unclassified	1
Pseudomonadota	10	Methylococcaceae	6
		Paracoccaceae	2
		Rhodoblastaceae	1
		Rhodocyclaceae	1

Table S3 Annotation of each gene in the *zig* cluster from the genome of *Micromonospora zingiberis* PLAI 1-1[a]

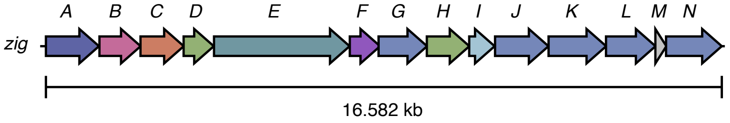

ORFs	size (aa)	protein homolog (origin, accession no.)	%ID/%SI[b]	proposed function
ZigA	433	aminotransferase PamX (*Streptomyces alboniger*)	33/52	aminotransferase
ZigB	336	geranylfarnesyl diphosphate synthase MrtP (*Streptomyces* sp. AJS-327)	41/51	polyprenyl synthase
ZigC	353	prenyltransferase AuaA (*Stigmatella aurantiaca* Sg a15)	27/41	prenyltransferase
ZigD	246	demethylmenaquinone methyltransferase MenG (*Thermus thermophilus* HB27)	42/55	methyltransferase
ZigE	1108	N-terminal subunit: α-hydroxy-β-keto acid synthase CsmA (*Chryseomicrobium* sp. PKU-MA01392)<br>C-termial subunit: pyruvate oxidase PoxB (*Escherichia coli* K-12)	N: 29/44[c]<br>C: 29/45[c]	thiamine pyrophosphate-dependent enzyme
ZigF	247	terpene cyclase DmtA1 (*Streptomyces youssoufiensis*)	39/54	terpene cyclase
ZigG	396	nocardicin C N-oxygenase (*Nocardia uniformis* subsp. *tsuyamanensis*)	39/56	cytochrome P450 monooxygenase
ZigH	347	O-methyltransferase SfmM3 (*Streptomyces lavendulae*)	45/59	methyltransferase
ZigI	207	tryptophan-7-hydroxylase MymH (*Streptomyces olivaceus*)	42/55	DUF6875-containing protein (cytochrome P422 monooxygenase)
ZigJ	436	cytochrome P450 SpiL (*Sorangium cellulosum*)	37/54	cytochrome P450 monooxygenase
ZigK	470	cytochrome P450 139A3 (*Mycobacterium marinum* M)	42/58	cytochrome P450 monooxygenase
ZigL	404	cytochrome P450 RapN (*Streptomyces rapamycinicus* NRRL 5491)	50/65	cytochrome P450 monooxygenase
ZigM	89	ferredoxin EncQ (*Streptomyces maritimus*)	48/59	ferredoxin
ZigN	455	cytochrome P450 Cac8 (*Streptantibioticus cattleyicolor*)	51/66	cytochrome P450 monooxygenase

[a]Gene bank accession, SJJR01000005.1; nucleotide positions, 54,716–38,135. [b]Sequence identity (%) and similarity (%). [c]Sequence identities and similarities of two subunits (N: N-terminal region, 21–455; C: C-terminal region, 522–1052).

Table S4 Primers used in this study

primer	sequence (5′ to 3′)
SP44SR41-F	CTAGAGGATCCTGTTCACATTCGAACCGTC
SP44SR41-R	CAGCCTACTCCTTACTTAGACTGTCGTATTCTCCTACACCAGACTTTACAAC
pSET152-SP44-F	GTCTAAGTAAGGAGTAGGCTGGCGGCCGCGCGCGATATC
pSET152-SP44-R	CGAATGTGAACAGGATCCTCTAGAGTCGACCTGCAGCC
zig-f1-F	CAGTCTAAGTAAGGAGTAGGCTGATGAGCCAGGTGCTGGCAACC
zig-f1-R	GACGGCGTCGATCTCCTGGAAGGACACG
zig-f2-F	CGTGTCCTTCCAGGAGATCGACGCCGTC
zig-f2-R	GCTATGACATGATTACGAATTCTCACCATGTGACCGGGAGCTGCTTCG
zig-f3-F	GAGGGCCCGAAGCAGCTCCCGGTCACATG
zig-f3-R	CCTGCGTCAGGGTCATCGCTTCTTCTCCTGC
zig-f4-F	CAGGAGAAGAAGCGATGACCCTGACGCAGG
zig-f4-R	GGAAACAGCTATGACATGATTACGTCAGTCGACCGGGCGGATGC
pSET152-inv-F	GGTGAGAATTCGTAATCATGTCATAGCTGTTTCC
pSET152-inv-R	CTGGCTCATCAGCCTACTCCTTACTTAGAC
Δ*zigA*-f1-F	GTAAGGAGTAGGCTGATGTCCACCCTCGTCCCGCCCACTC
Δ*zigC*-f1-R	CTGCTGGGCTACGACATGCTCCTCAGCCTCGCCTTTCTG
Δ*zigC*-f2-F	GAGGAGCATGTCGTAGCCCAGCAGAAAGGCCACCAC
Δ*zigD*-f1-R	CTCGCGCAGGTGTCGATCCGAGCCCATGCTGAGCAGTG
Δ*zigD*-f2-F	GCTCGGATCGACACCTGCGCGAGGAGATCAGCACCTAC
Δ*zigE*-f1-R	GATGGCGTCGATTTCGTGCCGGACGCCGATGAGTTGCAG
Δ*zigE*-f2-F	GTCCGGCACGAAATCGACGCCATCGTCGACCCGTTGGAAC
Δ*zigF*-f2-R	GCTATGACATGATTACGAATTCGATGAAGGCGTACAACGCCTCCCAGCTG
pSET152-Δ*zigF*-inv-F	CTTCATCGAATTCGTAATCATGTCATAGCTGTTTCC
Δ*zigF*-f3-F	GAGGCGTTGTACGCCTTCATCACCGAGGAACTCGGTCAAGCGGTC
Δ*zigG*-f2-R	GCTATGACATGATTACGAATTCGATCGACACCGGCAGTTGGACGAGACC
pSET152-Δ*zigG*-inv-F	CGATCGAATTCGTAATCATGTCATAGCTGTTTCC
Δ*zigG*-f3-F	GTCCAACTGCCGGTGTCGATCAACCAGCACCTCTCGTTCGGGATC
Δ*zigH*-f2-R	GCTATGACATGATTACGAATTCCACCACGTCGAACACGACAAGCGCGGTG
pSET152-Δ*zigH*-inv-F	GTGGTGGAATTCGTAATCATGTCATAGCTGTTTCC
Δ*zigH*-f3-F	CTTGTCGTGTTCGACGTGGTGATGGATTCCGTGGCGACCCTGC
Δ*zigI*-f3-R	GAACAGCACGTCCCGAATCTCCACCACGAGTGTCC
Δ*zigI*-f4-F	GGAGATTCGGGACGTGCTGTTCCTTGCCGACGACC
Δ*zigJ*-f3-R	ACAGCGCGAAGGTGATGTCCACCCGTACGCCGTAGG
Δ*zigJ*-f4-F	GTGGACATCACCTTCGCGCTGTACGAGATGCGCCTGACG
Δ*zigK*-f3-R	GCTCGAACAGCACGAAACAGACATCGCCGTACCGCTG
Δ*zigK*-f4-F	GTCTGTTTCGTGCTGTTCGAGCTGCGGCTCTGCGTACC
Δ*zigL*-f3-R	GAGCTCCATCCGCAACACCGTCCGGTTGTCGGCGTGGGTGAC
Δ*zigL*-f4-F	GGACGGTGTTGCGGATGGAGCTCTCGGTGGCACTGAC
Δ*zigM*-f3-R	GACGAGGAGAAGGTAACAGAACCCCGATCCGATGCAGACC
Δ*zigM*-f4-F	GGTTCTGTTACCTTCTCCTCGTCGCCGACGGCTGC
Δ*zigN*-f4-R	GGAAACAGCTATGACATGATTACGTCAGTCATCGGCGTGCTCCTGCAC
zigE-F	GTCTAAGTAAGGAGTAGGCTGGTGCGCGCCAACACCCACATCTAC
zigE-R	CAGCTATGACATGATTACGAATTCTTACCACCCCTGACGAGATCGTG
zigF-F	GTTTAACTTTAAGAAGGAGATATACCATGGAGCCCGGCACCAACGTGTTG
zigF-R	CAAGCTTGTCGACGGAGCTCGATCACGAGCGAAGGGGTAGCTCATC

Table S5 Plasmids used in this study

plasmid	description	source
pSET152	integrative vector in *Streptomyces* containing Apr^r, $int^{\varphi C31}$, and *oriT*	1
pET-28c(+)	protein expression vector in *E. coli* containing Kan^r, *lacI*, and *f1*	Novagen
pSET152-SP44-SR41	pSET152 containing a SP44 constitutive promoter and a SR41 ribosome binding site between *BamH*I and *Not*I	this study
pSET152-*zig*-f1-f2	pSET152-SP44-SR41 containing a part of the *zig* BGC	this study
pSET152-*zig*	pSET152-SP44-SR41 containing the *zig* BGC	this study
pSET152-*zig*Δ*zigA*-f1-f2	pSET152-SP44-SR41 containing a part of the *zig* BGC and used for the construction of pSET152-*zig*Δ*zigA*	this study
pSET152-*zig*Δ*zigA*	pSET152-SP44-SR41 containing the *zig* BGC lacking *zigA*	this study
pSET152-*zig*Δ*zigC*-f1-f2	pSET152-SP44-SR41 containing a part of the *zig* BGC and used for the construction of pSET152-*zig*Δ*zigC*	this study
pSET152-*zig*Δ*zigC*	pSET152-SP44-SR41 containing the *zig* BGC with in-flame deletion of *zigC*	this study
pSET152-*zig*Δ*zigD*-f1-f2	pSET152-SP44-SR41 containing a part of the *zig* BGC and used for the construction of pSET152-*zig*Δ*zigD*	this study
pSET152-*zig*Δ*zigD*	pSET152-SP44-SR41 containing the *zig* BGC with in-flame deletion of *zigD*	this study
pSET152-*zig*Δ*zigE*-f1-f2	pSET152-SP44-SR41 containing a part of the *zig* BGC and used for the construction of pSET152-*zig*Δ*zigE*	this study
pSET152-*zig*Δ*zigE*	pSET152-SP44-SR41 containing the *zig* BGC with in-flame deletion of *zigE*	this study
pSET152-*zig*Δ*zigF*-f1-f2	pSET152-SP44-SR41 containing a part of the *zig* BGC and used for the construction of pSET152-*zig*Δ*zigF*	this study
pSET152-*zig*Δ*zigF*	pSET152-SP44-SR41 containing the *zig* BGC with in-flame deletion of *zigF*	this study
pSET152-*zig*Δ*zigG*-f1-f2	pSET152-SP44-SR41 containing a part of the *zig* BGC and used for the construction of pSET152-*zig*Δ*zigG*	this study
pSET152-*zig*Δ*zigG*	pSET152-SP44-SR41 containing the *zig* BGC with in-flame deletion of *zigG*	this study
pSET152-*zig*Δ*zigH*-f1-f2	pSET152-SP44-SR41 containing a part of the *zig* BGC and used for the construction of pSET152-*zig*Δ*zigH*	this study
pSET152-*zig*Δ*zigH*	pSET152-SP44-SR41 containing the *zig* BGC with in-flame deletion of *zigH*	this study
pSET152-*zig*Δ*zigI*	pSET152-SP44-SR41 containing the *zig* BGC with in-flame deletion of *zigI*	this study
pSET152-*zig*Δ*zigJ*	pSET152-SP44-SR41 containing the *zig* BGC with in-flame deletion of *zigJ*	this study
pSET152-*zig*Δ*zigK*	pSET152-SP44-SR41 containing the *zig* BGC with in-flame deletion of *zigK*	this study
pSET152-*zig*Δ*zigL*	pSET152-SP44-SR41 containing the *zig* BGC with in-flame deletion of *zigL*	this study
pSET152-*zig*Δ*zigM*	pSET152-SP44-SR41 containing the *zig* BGC with in-flame deletion of *zigM*	this study
pSET152-*zig*Δ*zigN*	pSET152-SP44-SR41 containing the *zig* BGC lacking *zigN*	this study
pSET152-*zig*Δ*zigLN*	pSET152-SP44-SR41 containing the *zig* BGC lacking *zigLN*	this study
pSET152-*zigE*	pSET152-SP44-SR41 containing *zigE*	this study
pET28-*zigF*	pET-28c(+) containing *zigF*	this study

Table S6 Strains used in this study

strain	description	source
E. coli DH5α	host for general cloning	New England Biolabs
E. coli ET12567/pUZ8002	donor host for intergenic conjugation	2
E. coli Rosetta(DE3)pLysS	host for protein expression	Novagen
M. zingiberis PLAI 1-1	native strain harboring the *zig* BGC	TBRC
S. albus J1074	host for heterologous expression	3
S. lividans TK64	host for heterologous expression	4
S. coelicolor M1154	host for heterologous expression	5
S. albus::*zig*	*S. albus* harboring pSET152-*zig*	this study
S. albus::pSET152	*S. albus* harboring pSET152-SP44-SR41	this study
S. lividans::*zig*	*S. lividans* harboring pSET152-*zig*	this study
S. lividans::pSET152	*S. lividans* harboring pSET152-SP44-SR41	this study
S. coelicolor::*zig*	*S. coelicolor* harboring pSET152-*zig*	this study
S. coelicolor::pSET152	*S. coelicolor* harboring pSET152-SP44-SR41	this study
S. albus::*zig*Δ*zigA*	*S. albus* harboring pSET152-*zig*Δ*zigA*	this study
S. albus::*zig*Δ*zigC*	*S. albus* harboring pSET152-*zig*Δ*zigC*	this study
S. albus::*zig*Δ*zigD*	*S. albus* harboring pSET152-*zig*Δ*zigD*	this study
S. albus::*zig*Δ*zigE*	*S. albus* harboring pSET152-*zig*Δ*zigE*	this study
S. albus::*zig*Δ*zigF*	*S. albus* harboring pSET152-*zig*Δ*zigF*	this study
S. albus::*zig*Δ*zigG*	*S. albus* harboring pSET152-*zig*Δ*zigG*	this study
S. albus::*zig*Δ*zigH*	*S. albus* harboring pSET152-*zig*Δ*zigH*	this study
S. albus::*zig*Δ*zigI*	*S. albus* harboring pSET152-*zig*Δ*zigI*	this study
S. albus::*zig*Δ*zigJ*	*S. albus* harboring pSET152-*zig*Δ*zigJ*	this study
S. albus::*zig*Δ*zigK*	*S. albus* harboring pSET152-*zig*Δ*zigK*	this study
S. albus::*zig*Δ*zigL*	*S. albus* harboring pSET152-*zig*Δ*zigL*	this study
S. albus::*zig*Δ*zigM*	*S. albus* harboring pSET152-*zig*Δ*zigM*	this study
S. albus::*zig*Δ*zigN*	*S. albus* harboring pSET152-*zig*Δ*zigN*	this study
S. albus::*zig*Δ*zigLN*	*S. albus* harboring pSET152-*zig*Δ*zigLN*	this study
S. albus::*zigE*	*S. albus* harboring pSET152-*zigE*	this study
E. coli::*zigF*	*E. coli* Rosetta(DE3)pLysS harboring pET28-*zigF*	this study
E. coli::pET28c	*E. coli* Rosetta(DE3)pLysS harboring pET-28c(+)	this study

Table S7 ^{1}H (500.18 MHz) and ^{13}C{^{1}H} (125.78 MHz) NMR chemical shifts and 2D NMR data for **1** in $CDCl_3$ at 298K

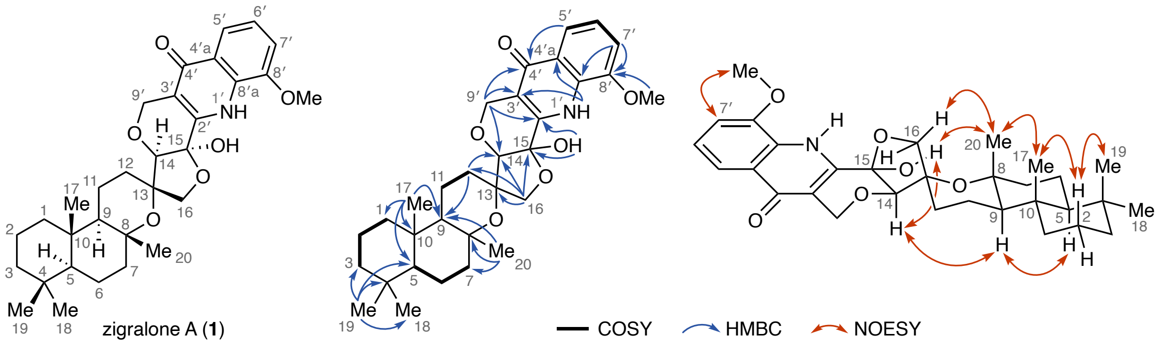

no.	δ_C, type	δ_H, mult. (J in Hz)	COSY	HMBC	NOESY
1	39.1, CH_2	a: 1.64, m	2b	5	1b
		b: 0.92, td (13.4, 3.7)	2a		1a, 3b
2	18.6, CH_2	a: 1.62, m	1a, 1b, 2b		
		b: 1.47, m[a]	2a, 3b	4	17, 19
3	42.1, CH_2	a: 1.40, m[b]	3b		3b, 18
		b: 1.16, td (13.5, 4.3)	2b, 3a	2, 4, 5, 19	1b, 3a, 5, 18
4	33.42, C				
5	56.6, CH	0.98, dd (12.3, 2.4)	6b	4, 6, 7, 9, 10, 17, 19	3b, 6a, 9
6	19.9, CH_2	a: 1.71, m	6b, 7a		5, 6b, 7a, 7b, 18
		b: 1.31, m	5, 6a	7	6a, 7a, 19
7	43.0, CH_2	a: 1.86, dt (12.0, 3.2)	6a, 7b	6, 9, 20	6a, 6b, 7b, OH-15
		b: 1.46, m[a]	7a	6	6a, 7a, OH-15
8	77.9, C				
9	56.4, CH	1.41, m[b]			5, 14
10	37.3, C				
11	15.5, CH_2	a: 1.77, m	11b, 12a, 12b	8, 13, 12	14
		b: 1.41, m[b]	11a, 12a, 12b		17
12	25.9, CH_2	a: 2.03, m	11a, 11b	13, 14, 16	14
		b: 1.98, m	11a, 11b	9, 11	16a, 16b
13	83.1, C				
14	86.3, CH	3.77, s		13, 15, 16, 9′	9, 11a, 12a, 9′b, OH-15
15	98.4, C				
16	78.0, CH_2	a: 4.48, d (9.3)	16b	13, 14, 15	12b, 20, OH-15
		b: 3.87, d (9.3)	16a	12, 13, 15	12b
17	15.6, CH_3	0.80, s		1, 5, 9, 10	2b, 11b, 20
18	33.43, CH_3	0.87, s		3, 4, 5, 19	3a, 3b, 6a
19	21.4, CH_3	0.81, s		3, 4, 5, 18	2b, 6b
20	24.8, CH_3	1.43, s		7, 8, 9	16a, 17, OH-15
OH-15		5.30, s		14, 15, 2′	7a, 7b, 14, 16a, 20, NH-1′
1′		9.14, br s		3′, 4′a	OH-15, OMe-8′
2′	140.3, C				
3′	114.6, C				
4′	175.9, C				
4′a	125.2, C				
5′	117.2, CH	7.88, ddd (8.3, 1.2, 0.3)	6′	4′, 6′, 7′, 8′[c], 8′a	
6′	122.9, CH	7.22, dd (8.3, 7.8)	5′, 7′	4′a, 8′, 8′a[c]	
7′	110.4, CH	7.01, dd (7.8, 1.2)	6′	5′, 8′, 8′a	OMe-8′
8′	148.1, C				
8′a	130.6, C				
9′	63.1, CH_2	a: 5.04, d (15.1)	9′b	14, 2′, 3′, 4′	
		b: 4.55, d (15.1)	9′a	14, 2′, 3′	14
OMe-8′	56.0, CH_3	3.97, s		8′	7′, NH-1′

[a–b]Signals are overlapped. [c]Long-range heteronuclear correlations.

Table S8 ^1H (500.18 MHz) and $^{13}C\{^1H\}$ (125.78 MHz) NMR chemical shifts and 2D NMR data for **2** in $(CD_3)_2CO$ at 298K

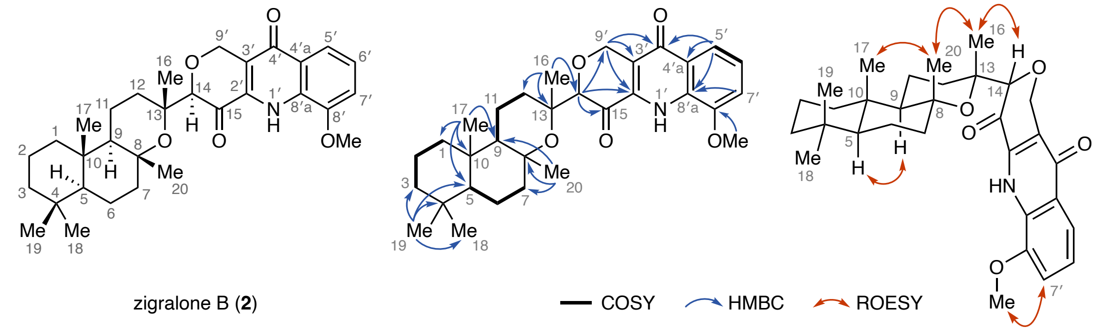

no.	δ_C, type	δ_H, mult. (*J* in Hz)	COSY	HMBC	ROESY
1	39.7, CH_2	a: 1.62, m[a]			
		b: 0.83, td (13.2, 4.0)	2a, 2b	17	
2	19.2, CH_2	a: 1.62, m[a]	1b		
		b: 1.41, m	1b, 3b		
3	42.8, CH_2	a: 1.32, m			
		b: 1.12, m	2b		5
4	33.8, C				
5	57.1, CH	0.73, dd (10.0, 2.7)	6b	6, 17	3b, 6a, 9
6	20.1, CH_2	a: 1.35, m	6b, 7b		5
		b: 1.15, m	5, 6a		
7	43.0, CH_2	a: 1.27, m	7b	5	
		b: 0.69, m	6a, 7a	8	
8	76.0, C				
9	58.7, CH	0.90, dd (10.5, 3.4)	11a, 11b	8, 10, 20	5
10	37.5, C				
11	15.5, CH_2	a: 1.62, m[a]	9		
		b: 1.59, m	9, 12a		
12	34.3, CH_2	a: 2.15, m	11b, 12b		14
		b: 1.62, m[a]	12a		14
13	79.7, C				
14	86.0, CH	3.95, s		12, 13, 15, 2′, 9′	12a, 12b, 16, 9′b
15	193.3, C				
16	25.5, CH_3	1.57, s		12, 13, 14	14, 20, 9′a
17	16.2, CH_3	0.75, s		1, 5, 9, 10	20
18	33.5, CH_3	0.70, s		3, 4, 5, 19	
19	21.5, CH_3	0.73, s		3, 4, 5, 18	
20	24.7, CH_3	1.27, d (0.7)		7, 8, 9	16, 17
1′		9.32, br s			
2′	134.3, C				
3′	124.3, C				
4′	177.3, C				
4′a	126.5, C				
5′	117.3, CH	7.74, m	6′	4′, 4′a, 7′, 8′[c],8′a	
6′	124.1, CH	7.30, m[b]	5′	4′a, 8′	
7′	112.4, CH	7.30, m[b]		5′, 8′a	OMe-8′
8′	149.8, C				
8′a	130.9, C				
9′	62.0, CH_2	a: 5.20, d (15.9)	9′b	2′, 3′	16
		b: 4.82, d (15.9)	9′a	14, 2′, 3′, 4′	14
OMe-8′	56.8, CH_3	4.11, s		8′	7′

[a–b]Signals are overlapped. [c]Long-range heteronuclear correlations.

Table S9 ^1H (500.18 MHz) and $^{13}C\{^1H\}$ (125.78 MHz) NMR chemical shifts and 2D NMR data for **3** in $(CD_3)_2CO$ at 298K

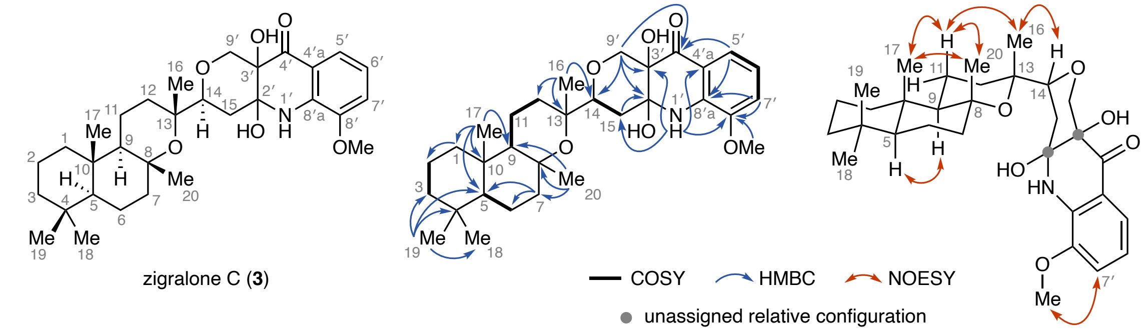

zigralone C (**3**)

no.	δ_C, type	δ_H, mult. (J in Hz)	COSY	HMBC	NOESY
1	39.9, CH_2	a: 1.66, m[a]	1b		1b
		b: 0.89, m	1a	2	1a, 9
2	19.3, CH_2	a: 1.66, m[a]			
		b: 1.43, m	3b		
3	42.9, CH_2	a: 1.39, m[c]	3b		
		b: 1.18, m	3a, 2b	2, 4, 18, 19	
4	33.9, C				
5	57.4, CH	0.99, dd (11.8, 2.6)	6a, 6b	6, 7, 10, 18	9
6	20.5, CH_2	a: 1.66, m[a]	5		
		b: 1.37, m[c]	5		7a
7	44.0, CH_2	a: 1.71, m	7b	5, 6, 8, 9, 20	6b, 7b
		b: 1.37, m[c]	7a		7a
8	75.3, C				
9	59.3, CH	1.16, m	11a, 11b	8, 11, 12, 17, 20	1b, 5
10	37.59, C				
11	15.8, CH_2	a: 1.57, m	9, 12a		12a, 16, 17, 20
		b: 1.52, m[b]	9, 12a		12a
12	37.57, CH_2	a: 1.83, dt (12.7, 3.5)	11a, 11b, 12b	9, 11, 13, 16	11a, 11b, 12b, 16
		b: 1.51, m[b]	12a		12a, 14
13	74.0, C				
14	84.9, CH	3.51, dd (11.4, 2.3)	15a, 15b	12, 13, 16, 9′	12b, 15a, 16, NH-1′
15	35.4, CH_2	a: 2.11, dd (13.4, 2.3)	14, 15b	2′, 3′	14, NH-1′
		b: 1.96, dd (13.4, 11.4)	14, 15a	13, 14, 2′	16
16	22.4, CH_3	1.24, s		12, 13, 14	11a, 12a, 14, 15b
17	16.1, CH_3	0.82, s		1, 5, 9, 10	11a, 20
18	33.7, CH_3	0.89, s		3, 4, 5, 19	
19	21.6, CH_3	0.83, s		3, 4, 5, 18	
20	25.3, CH_3	1.30, s		7, 8, 9	11a, 17
1′		5.79, s		15, 3′, 4′a, 8′	14, 15a
2′	86.0, C				
3′	77.9, C				
4′	196.2, C				
4′a	117.4, C				
5′	118.5, CH	7.29, dd (7.9, 1.1)	6′	4′, 7′, 8′a	
6′	118.4, CH	6.73, t (7.9)	5′, 7′	4′a, 7′, 8′, 8′a[e]	
7′	115.8, CH	7.08, dd (7.9, 1.1)	6′	5′, 8′, 8′a	OMe-8′
8′	148.3, C				
8′a	140.3, C				
9′	72.2, CH_2	a: 3.62, d (11.4)	9′b	14, 3′	
		b: 3.49, d (11.4)	9′a	14, 2′, 3′, 4′	
OH-2′		4.33, br s[d]			15b
OH-3′		4.32, br s[d]			15b
OMe-8′	56.2, CH_3	3.91, s		8′	7′

[a–c]Signals are overlapped. [d]Signals may be interchanged. [e]Long-range heteronuclear correlations.

Table S10 ^{1}H (500.18 MHz) and ^{13}C{^{1}H} (125.78 MHz) NMR chemical shifts and 2D NMR data for **4** in $(CD_3)_2CO$ at 298K

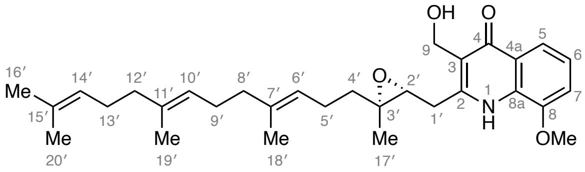

zigralone D (**4**)

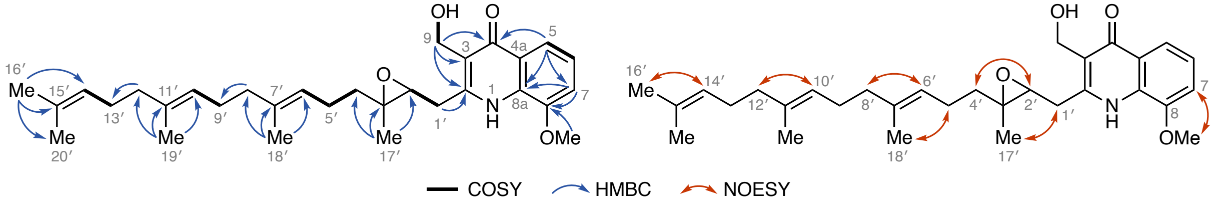

no.	δ_C, type	δ_H, mult. (*J* in Hz)	COSY	HMBC	NOESY
1		9.84, br s			
2	148.1, C				
3	120.8, C				
4	178.1, C				
4a	126.1, C				
5	117.6, CH	7.76, dd (7.8, 1.6)	6	4, 7, 8a	
6	123.5, CH	7.23, t (7.8)	5	4a, 5, 7, 8	
7	111.4, CH	7.20, dd (7.8, 1.6)		5, 6, 8, 8a	OMe-8
8	149.0, C				
8a	131.5, C				
9	57.3, CH_2	4.67, m	OH-9	2, 3, 4	1′a, 1′b
OMe-8	56.6, CH_3	4.02, s		8	7
OH-9		4.17, t (6.0)	9		
1′	31.2, CH_2	3.46, dd (14.9, 3.4)	1′b, 2′	2, 3, 2′, 3′	9, 17′
		2.95, dd (14.9, 8.4)	1′a, 2′	2, 3, 2′, 3′	9, 17′
2′	62.5, CH	3.17, dd (8.4, 3.6)	1′a, 1′b	2, 1′, 4′	4′a, 4′b, 5′
3′	61.9, C				
4′	39.2, CH_2	1.49, dt (13.7, 8.2)	4′b, 5′	2′, 3′, 5′, 6′, 17′	2′, 5′, 6′
		1.69, dt (13.7, 7.7)	4′a, 5′	2′, 3′, 5′, 6′, 17′	2′, 6′
5′	24.3, CH_2	2.13, br q (7.7)	4′a, 4′b, 6′	3′, 4′, 6′, 7′	2′, 4′a, 17′, 18′
6′	124.5, CH	5.14, tq (7.2, 1.3)	5′, 18′[d]	5′, 8′	4′a, 4′b, 8′
7′	136.1, C				
8′	40.3, CH_2	1.95, m[a]		6′, 7′, 9′	6′
9′	27.5, CH_2	2.06, m[c]	10′	8′, 10′, 11′	18′
10′	125.0, CH	5.10, m	9′	9′, 12′	12′
11′	135.5, C				
12′	40.5, CH_2	1.96, m[a]		11′, 13′, 14′	10′
13′	27.2, CH_2	2.05, m[c]	14′	11′, 12′, 14′, 15′	
14′	125.2. CH	5.08, m	13′, 16′[d]	16′, 20′	16′
15′	131.6, C				
16′	25.8, CH_3	1.64, q (1.3)	14′[d]	14′, 15′, 20′	14′
17′	17.1, CH_3	1.46, s		2′, 3′, 4′	1′a, 1′b, 5′
18′	16.08, CH_3	1.60, s	6′[d]	6′, 7′, 8′	5′, 9′
19′	16.05, CH_3	1.581, br s[b]		10′, 11′, 12′	
20′	17.7, CH_3	1.576, br s[b]		14′, 15′, 16′	

[a–b]Signals are overlapped. [c]Signals are overlapped with the solvent signal. [d]Long-range homonuclear correlations.

Table S11 ^{1}H (500.18 MHz) and $^{13}C\{^{1}H\}$ (125.78 MHz) NMR chemical shifts and 2D NMR data for **5** in $(CD_3)_2CO$ at 298K

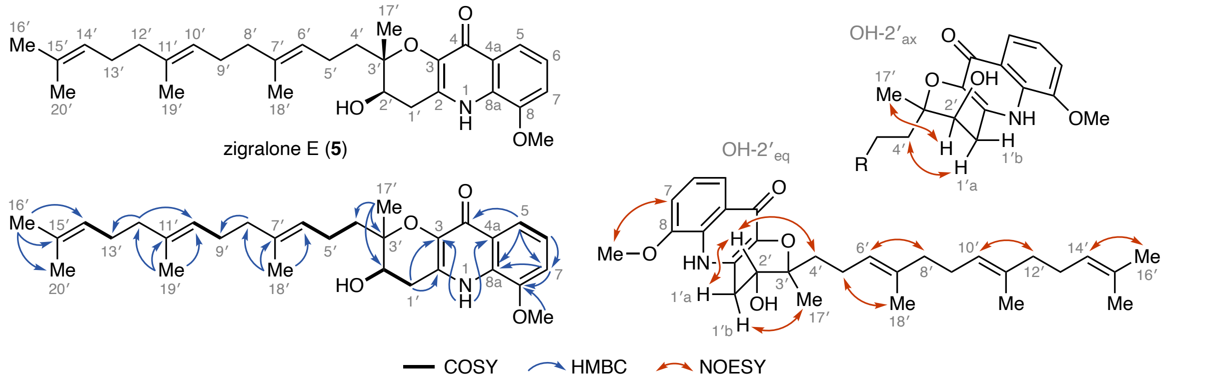

no.	δ_C, type	δ_H, mult. (J in Hz)	COSY	HMBC	NOESY
1		9.79, br s		3, 4a	
2	130.0, C				
	129.9, C[a]				
3	137.27, C				
	137.25, C[a]				
4	170.6, C				
4a	126.21, C				
	126.19, C[a]				
5	117.7, CH	7.77, dd (7.7, 1.8)	6	4, 7, 8[e], 8a	
6	121.6, CH	7.09, t (7.7)	5	4a, 7, 8	
7	109.9, CH	7.06, dd (7.7, 1.8)		5, 6, 8, 8a	OMe-8
8	148.93, C				
	148.90, C[a]				
8a	130.4, C				
	130.3, C[a]				
OMe-8	56.3, CH_3	3.97, s		8	7
1′	31.3, CH_2	a: 3.20, dd (17.4, 5.7)	1′b, 2′	2, 3, 2′, 3′	1′b, 2′, 4′
	31.2, CH_2[a]	b: 2.89, dd (17.4, 7.1)	1′a, 2′	2, 3, 2′, 3′	1′a, 17′
2′	67.5, CH	3.98, m	1′a, 1′b, OH-2′	2, 17′	1′a, 4′, 5′, 17′
	67.4, CH[a]				
3′	78.9, C				
4′	38.1, CH_2	1.71, m	5′	2′, 3′, 5′, 17′	1′a, 2′, 6′, 17′
5′	22.2, CH_2	2.24, m	4′, 6′	4′, 6′, 7′	2′, 6′, 17′, 18′
6′	125.4, CH	5.17, tq (7.1, 1.3)	5′	4′, 5′, 8′, 18′	4′, 5′, 8′
7′	135.6, C				
8′	40.4, CH_2[b]	1.97, m		6′, 9′	6′, 18′
9′	27.3, CH_2	2.06, m[c]	10′		
10′	125.1, CH	5.12, m	9′	9′, 19′	12′
11′	135.4, C				
12′	40.5, CH_2[b]	1.95, m		10′, 13′, 14′	10′
13′	27.4, CH_2	2.05, m[c]	14′		
14′	125.2, CH	5.09, m	13′	16′, 20′	16′
15′	131.6, C				
16′	25.8, CH_3	1.64, q (1.3)		14′, 15′, 20′	14′
17′	18.3, CH_3	1.27, s		2′, 3′, 4′	1′b, 2′, 4′, 5′
18′	16.11, CH_3	1.61, s		6′, 7′, 8′	5′, 8′
19′	16.08, CH_3	1.58, s[d]			
20′	17.7, CH_3	1.58, s[d]			
OH-2′		4.36, br d (4.7)	2′		

[a]Split signals may be attributable to a rotamer. [b]Signals may be interchanged. [c]Signals are overlapped with the solvent signal. [d]Signals are overlapped. [e]Long-range heteronuclear correlations.

Table S12 Antimicrobial activity of **1–3**

		MIC[a] (μg/mL)			
classification	organism	**1**	**2**	**3**	apramycin
Gram-positive	*E. faecium* DSM 25390	> 128	128	8	16
	S. aureus DSM 2569	> 128	2	2	2
Gram-negative	*K. pneumoniae* DSM 109340	> 128	> 128	128	2
	A. baumannii ATCC 19606	> 128	128	128	32
	P. aeruginosa DSM 19880	> 128	> 128	128	2
	E. cloacae DSM 30054	> 128	128	128	4

[a]Minimum inhibitory concentration

Table S13 ^{1}H (500.18 MHz) and $^{13}C\{^{1}H\}$ (125.78 MHz) NMR chemical shifts and 2D NMR data for **6** in $CDCl_3$ at 298K

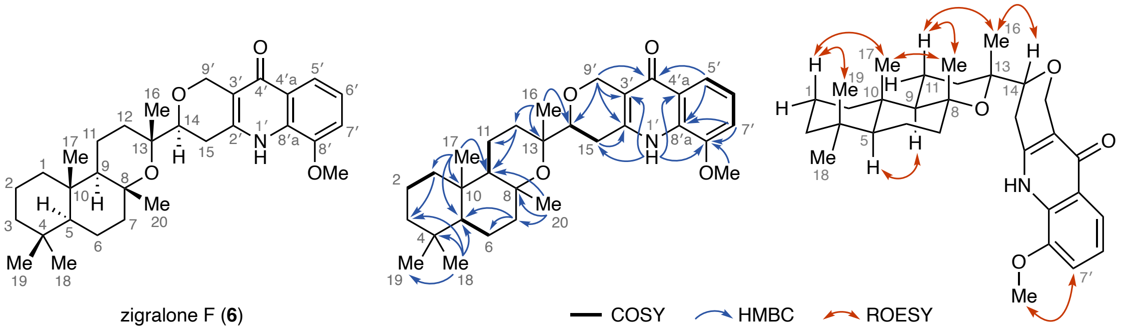

no.	δ_C, type	δ_H, mult. (*J* in Hz)	COSY	HMBC	NOESY
1	39.3, CH_2	a: 1.65, m[a]	1b	3, 5, 10	1b, 2a
		b: 0.86, m[b]	1a, 2b		1a, 9
2	18.7, CH_2	a: 1.62, m[c]	2b		1a, 17, 19
		b: 1.44, m	1b, 2a		3b
3	42.3, CH_2	a: 1.38, m	3b		3b, 18, 19
		b: 1.13, m[d]	3a		2b, 3a
4	33.4, C				
5	56.7, CH	0.92, dd (11.9, 2.6)	6b	4, 6, 7, 10, 17, 18, 19	1b, 6a, 9
6	20.0, CH_2	a: 1.65, m[a]	6b	5, 8, 10	
		b: 1.30, m[e]	5, 6a		
7	43.2, CH_2	a: 1.76, dd (8.8, 3.2)	7b	5, 6, 8, 9, 20	7b
		b: 1.30, m[e]	7a		7a
8	75.1, C				
9	58.3, CH	1.13, m[d]	11b		5, 14
10	37.1, C				
11	15.1, CH_2	a: 1.62, m[c]			12a
		b: 1.54, m[f]	9		12a, 16, 17
12	36.0, CH_2	a: 1.89, dd (8.9, 3.5)	12b	9, 11, 13, 16	11a, 11b, 14, 16
		b: 1.54, m[f]	12a	11, 13, 14, 16	14, 15b
13	73.7, C				
14	82.0, CH	3.38, dd (10.7, 3.0)	15a, 15b	12, 13, 16, 9′	9, 12a, 12b, 16, 9′b
15	27.2, CH_2	a: 2.95, br dd (16.5, 10.7)	14, 15a	13, 14, 2′, 3′	16, NH-1′
		b: 2.71, ddd (16.5, 3.0, 1.5)	14, 15a	2′, 3′	12b, 16, NH-1′
16	22.3, CH_3	1.302, s[e]		12, 13, 14	11b, 12a, 14, 15a, 15b
17	15.8, CH_3	0.79, s		1, 5, 9, 10	2a, 11b, 20
18	33.5, CH_3	0.85, s[b]		3, 4, 5, 19	3a
19	21.4, CH_3	0.80, s		3, 4, 5, 18	2a, 3a
20	25.0, CH_3	1.299, s[e]		7, 8, 9	17
1′		8.43, br s		15, 3′, 4′a, 8′	15a, 15b
2′	143.8, C				
3′	116.0, C				
4′	175.7, C				
4′a	125.2, C				
5′	117.5, CH	7.89, d (8.0)	6′	4′, 7′, 8′[g], 8′a	
6′	122.5, CH	7.20, t (8.0)	5′, 7′	4′a, 7′, 8′, 8′a[g]	
7′	110.1, CH	7.00, dd (8.0, 1.0)	6′	5′, 8′, 8′a	OMe-8′
8′	147.3, C				
8′a	130.4, C				
9′	65.2, CH_2	a: 5.02, d (15.0)	9′b	14, 2′, 3′, 4′	
		b: 4.55, dt (15.0, 1.9)	9′a	2′, 3′	14
OMe-8′	56.1, CH_3	3.99, s		8′	7′

[a–f]Signals are overlapped. [g]Long-range heteronuclear correlations.

Table S14 ^{1}H (500.18 MHz) and $^{13}C\{^{1}H\}$ (125.78 MHz) NMR chemical shifts and 2D NMR data for **7** in $CDCl_3$ at 298K

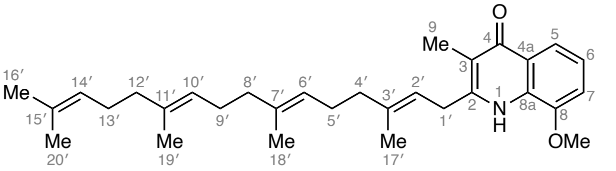

zigralone G (**7**)

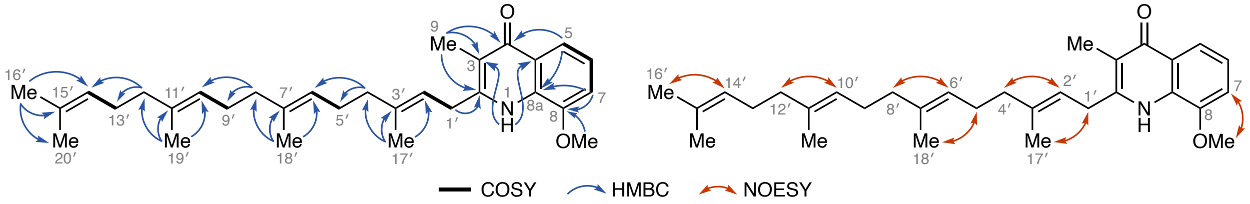

no.	δ_C, type	δ_H, mult. (J in Hz)	COSY	HMBC	NOESY
1		8.70, s		3, 4a	1′, 2′
2	145.1, C				
3	116.0, C				
4	177.8, C				
4a	124.5, C[a]				
5	117.9, CH	7.93, d (8.0)	6	4, 6, 7, 8[f], 8a	
6	122.4, CH	7.19, t (8.0)	5, 7	4a, 5, 7, 8, 8a[f]	
7	109.5, CH	6.96, dd (8.0, 0.9)	6	5, 8, 8a	OMe-8
8	147.5, C				
8a	129.8, C				
9	10.4, CH_3	2.16, s	1′	2, 3, 4	
OMe-8	56.1, CH_3	3.97, s		8	7
1′	30.7, CH_2	3.49, d (7.4)	2′, 9′[e], 17′[e]	2, 3, 2′, 3′, 4′[f]	2′, 9′, 17′, NH-1
2′	116.7, CH	5.39, tq (7.4, 1.2)	1′, 17′[e]	2, 1′, 4′, 17′	1′, 4′, NH-1
3′	143.9, C				
4′	40.0, CH_2	2.20, m[c]		2′, 3′, 5′, 6′, 17′	2′, 17′
5′	27.2, CH_2	2.21, m[c]	6′	4′, 6′, 7′	6′, 18′
6′	123.6, CH	5.19, m	5′, 18′[e]	4′, 5′, 8′, 18′	5′, 8′
7′	136.0, C				
8′	39.8, CH_2	2.01, m		6′, 7′, 9′, 10′, 18′	6′
9′	26.7, CH_2	2.05, m[d]	1′[e], 10′	9′, 19′	1′
10′	124.2, CH	5.10, m[b]	9′		12′
11′	135.2, C				
12′	39.9, CH_2	1.98, m		10′, 11′, 13′, 14′, 19′	10′
13′	26.9, CH_2	2.05, m[d]	14′, 16′[e]		
14′	124.5, CH[a]	5.09, m[b]	13′, 16′[e]		16′
15′	131.5, C				
16′	25.8, CH_3	1.68, br q (1.1)	13′[e], 14′[e]	14′, 15′, 20′	14′
17′	16.7, CH_3	1.76, s	1′[e], 2′[e]	1′[f],2′, 3′, 4′	1′, 4′
18′	16.2, CH_3	1.64, s	6′[e]	6′, 7′, 8′	5′
19′	16.1, CH_3	1.58, s		10′, 11′, 12′	
20′	17.8, CH_3	1.60, s		14′, 15′, 16′	

[a–d]Signals are overlapped. [e]Long-range homonuclear correlations. [f]Long-range heteronuclear correlations.

Table S15 ^{1}H (500.18 MHz) and $^{13}C\{^{1}H\}$ (125.78 MHz) NMR chemical shifts and 2D NMR data for **9** in $CDCl_3$ at 298K

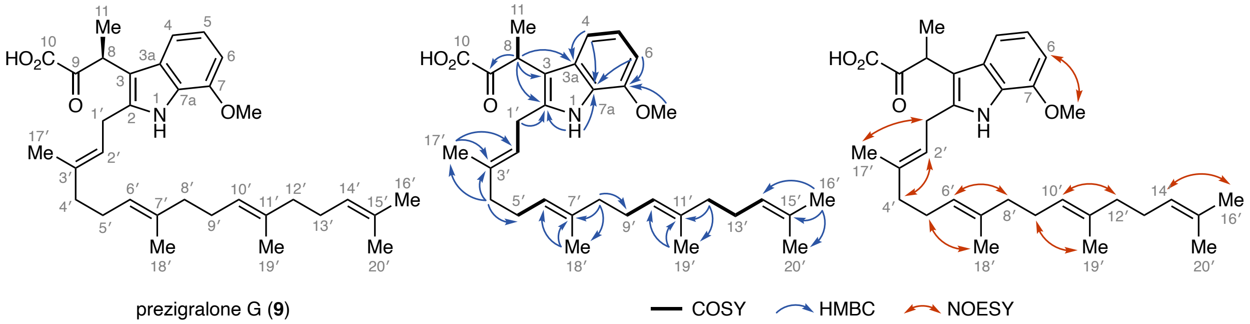

no.	δ_C, type	δ_H, mult. (J in Hz)	COSY	HMBC	NOESY
1		8.18, br s		2, 3, 3a, 7a	1′a, 1′b, 2′, 17′
2	137.3, C				
3	104.9, C				
3a	128.4, C				
4	111.6, CH	7.09, d (8.0)	5	3, 3a, 6, 7[c], 7a	8, 11
5	120.5, CH	6.98, t (8.0)	4, 6	3a, 6, 7, 7a[c]	
6	101.7, CH	6.58, d (8.0)	5	4, 7, 7a	OMe-7
7	145.9, C				
7a	125.7, C				
8	37.5, CH	4.87, q (7.0)	11	2, 3, 3a, 9, 11	4, 1′a, 1′b
9	192.9, C				
10	160.1, C				
	160.0, C[a]				
11	14.6, CH_3	1.581, d (7.0)	8	3, 8, 9	4, 1′a, 1′b
OMe-7	55.4, CH_3	3.92, s		7	6
1′	25.3, CH_2	a: 3.63, dd (16.7, 7.4)	2′	2, 3, 2′, 3′	NH-1, 8, 11, 2′, 17′
		b: 3.57, dd (16.7, 7.1)	2′	2, 3, 2′, 3′	NH-1, 8, 11, 2′, 17′
2′	119.3, CH	5.35, m	1′a, 1′b	2, 1′, 17′	NH-1, 1′a, 1′b, 4′
3′	139.4, C				
4′	39.9, CH_2	2.13, m		2′, 3′, 5′, 6′, 17′	2′, 6′, 17′
5′	26.6, CH_2	2.15, m	6′	3′, 6′, 7′	18′
6′	123.9, CH	5.15, m	5′	5′, 18′	4′, 8′
7′	135.7, C				
8′	39.83, CH_2[b]	2.02, m		6′, 7′, 9′, 10′, 18′	6′, 10′
9′	26.8, CH_2	2.08, m	10′		19′
10′	124.3, CH	5.11, m	9′	9′, 19′	8′, 12′
11′	135.1, C				
12′	39.82, CH_2[b]	1.97, m	13′	10′, 11′, 13′, 14′, 19′	10′, 14′
13′	26.9, CH_2	2.06, m	12′, 14′	15′	
14′	124.5, CH	5.09, m	13′	16′, 20′	12′, 16′
15′	131.4, C				
16′	25.8, CH_3	1.68, s		14′, 15′, 20′	14′
17′	16.5, CH_3	1.76, s		2′, 3′	NH-1, 1′a, 1′b, 4′
18′	16.2, CH_3	1.63, s		6′, 7′	5′
19′	16.1, CH_3	1.584, s		10′, 11′	9′
20′	17.8, CH_3	1.60, s		14′, 15′, 16′	

[a]Split signals may be attributable to a rotamer. [b]Signals may be interchanged. [c]Long-range heteronuclear correlations.

Table S16 ^{1}H (500.18 MHz) and $^{13}C\{^{1}H\}$ (125.78 MHz) NMR chemical shifts and 2D NMR data for **10** in $CDCl_3$ at 298K

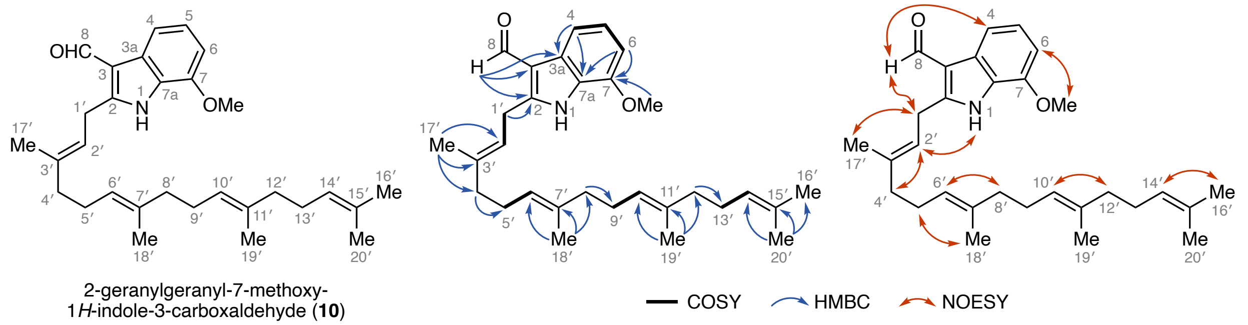

no.	δ_C, type	δ_H, mult. (J in Hz)	COSY	HMBC	NOESY
1		8.56, br s			1′, 2′
2	148.6, C				
3	114.5, C				
3a	127.9, C				
4	113.5, CH	7.80, d (8.0)	5	3, 3a, 6, 7[d], 7a	8
5	123.5, CH	7.18, t (8.0)	4, 6	3a, 6, 7	
6	103.7, CH	6.71, d (8.0)	5	4, 7, 7a	OMe-7
7	145.6, C				
7a	125.1, C				
8	184.7, CH	10.22, s		2, 3, 3a	4, 1′
OMe-7	55.5, CH_3	3.95, s		7	6
1′	25.5, CH_2	3.87, d (7.3)	2′, 17′[c]	2, 3, 2′, 3′, 4′[d]	NH-1, 8, 2′, 17′
2′	117.8, CH	5.40, tq (7.3, 1.3)	1′, 17′[c]	2, 1′, 4′, 17′	NH-1, 1′, 4′
3′	141.0, C				
4′	39.7, CH_2	2.15, m[a]		2′, 3′, 5′, 6′, 17′	2′, 6′, 17′
5′	26.5, CH_2	2.16, m[a]	6′	3′, 4′, 6′, 7′	18′
6′	123.6, CH	5.13, m	5′, 18′[c]	5′, 18′	4′, 8′
7′	136.0, C				
8′	39.8, CH_2	2.01, m		6′, 7′, 9′, 18′	6′, 18′
9′	26.8, CH_2	2.06, m[b]	10′	7′, 8′, 10′, 11′	
10′	124.3, CH	5.10, m	9′, 19′[c]	9′, 19′	12′
11′	135.2, C				
12′	39.9, CH_2	1.96, m		10′, 11′, 13′, 14′, 19′	10′
13′	26.9, CH_2	2.05. m[b]	14′	12′, 15′	
14′	124.5, CH	5.08, m	13′, 16′[c], 20′[c]	13′, 16′, 20′	16′
15′	131.4, C				
16′	25.8, CH_3	1.67, s	14′[c]	14′, 15′, 20′	14′
17′	16.6, CH_3	1.76, s	1′[c], 2′[c]	2′, 3′, 4′	1′, 4′
18′	16.2, CH_3	1.62, s	6′[c]	6′, 7′, 8′	5′, 8′
19′	16.1, CH_3	1.57, s	10′[c]	10′, 11′, 12′	
20′	17.8, CH_3	1.59, s	14′[c]	14′, 15′, 16′	

[a,b]Signals are overlapped. [c]Long-range homonuclear correlations. [d]Long-range heteronuclear correlations.

Table S17 ^{1}H (500.18 MHz) and $^{13}C\{^{1}H\}$ (125.78 MHz) NMR chemical shifts and 2D NMR data for **12** in $CDCl_3$ at 298K

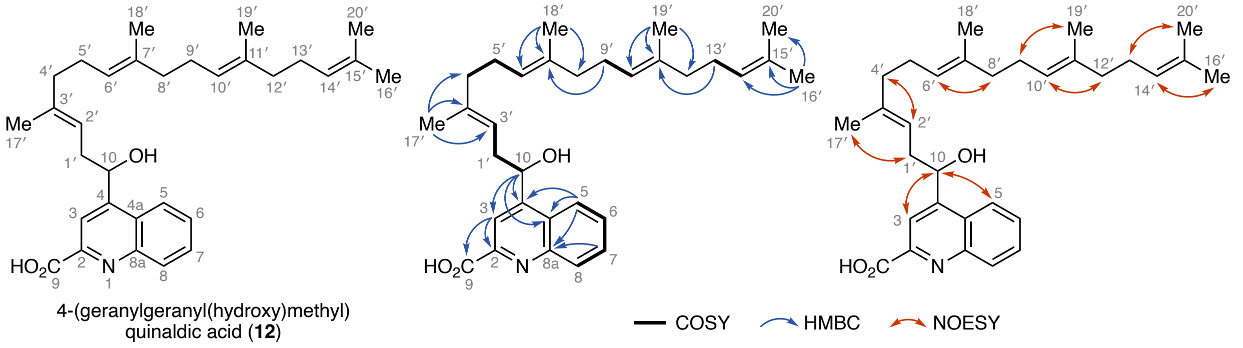

no.	δ_C, type	δ_H, mult. (J in Hz)	COSY	HMBC	NOESY
2	146.8, C				
3	116.3, CH	8.54, s		2, 4a, 9, 10	10, 1′
4	153.6, C				
4a	127.5, C				
5	123.8, CH	7.75, d (8.5)	6	4, 4a, 7, 8a	1′, 10
6	128.4, CH[a]	7.16[a]	5, 7	8	
7	130.0, CH	7.29, t (7.6)	6, 8	5, 8a	
8	130.6, CH	7.85, d (8.5)	7	4a, 6	
8a	146.3, C				
9	163.9, C				
10	70.2, CH	4.94, dd (7.4, 4.7)	1′	3, 4, 4a, 1′, 2′	3, 5, 1′
1′	37.2, CH_2	2.34, m	10, 2′	4, 10, 2′, 3′	3, 5, 10, 2′, 17′
2′	119.5, CH	5.17, br t (7.4)	1′, 17′[d]	10, 1′, 4′, 17′	1′, 4′
3′	139.9, C				
4′	40.17, CH_2	2.00, t (7.4)	5′	2′, 3′, 5′, 6′, 17′	2′, 6′, 17′
5′	26.9, CH_2	2.09, m[c]	4′, 6′	3′	17′
6′	124.4, CH	5.22, m	5′, 18′[d]	4′, 5′, 8′, 18′	4′, 8′
7′	135.7, C				
8′	40.22, CH_2	2.11, m[c]		6′, 7′, 9′, 10′	6′
9′	27.1, CH_2	2.21, m	10′	7′, 8′, 10′, 11′	19′
10′	124.8, CH	5.30, tq (7.0, 1.3)	9′, 19′[d]	8′, 9′, 12′, 19′	12′
11′	135.2, C				
12′	40.3, CH_2	2.10, m[c]		11′, 13′, 14′	10′
13′	27.3, CH_2	2.18, m	14′	11′, 12′, 14′, 15′	20′
14′	125.0, CH	5.24, m	13′, 16′[d], 20′[d]	16′, 20′	16′
15′	131.2, C				
16′	25.9, CH_3	1.68, q (1.3)	14′[d]	14′, 15′, 20′	14′
17′	16.3, CH_3	1.36, s	2′[d]	2′, 3′, 4′	1′, 4′, 5′
18′	16.2, CH_3[b]	1.61, s	6′[d]	6′, 7′, 8′	
19′	16.2, CH_3[b]	1.62, s	10′[d]	10′, 11′, 12′	9′
20′	17.8, CH_3	1.56, s	14′[d]	14′, 15′, 16′	13′

[a]Signals are overlapped with the solvent signal and assigned by the HMBC cross-peak. [b,c]Signals are overlapped. [d]Long-range homonuclear correlations.

3. Experimental procedures

General materials and methods

All chemical and biological reagents were purchased from Carl Roth, Sigma Aldrich, or Thermo Fisher Scientific, unless noted otherwise. Oligonucleotide primer synthesis and plasmid sequencing were performed by Microsynth AG. Restriction endonucleases, Q5 High-Fidelity DNA Polymerase, and deoxynucleotides, NEBuilder HiFi DNA Assembly Master Mix, Monarch Plasmid DNA Miniprep Kit, Monarch DNA Gel Extraction Kit, and Monarch Genomic DNA Purification Kit were purchased from New England Biolabs and used according to the manufacturer's protocols.

LC-MS analysis was performed with a Thermo Scientific Dionex Ultimate 3000 UHPLC system coupled to a Bruker impact II QTOF mass spectrometer. Chromatographic separation was performed with a Kinetex EVO C18 column (2.1 × 100 mm, 2.6 μm) using a biphasic mobile phase of 0.1% formic acid in H_2O (A) and 0.1% formic acid in MeCN (B) using the following gradient: 2% B (0–1 min); 2–35% B (1–5 min); 35–98% B (5–20 min); 98% B (20–22.5 min); 2% B (22.6–26 min) at a flow rate of 0.6 mL/min. The ESI parameters in positive ion mode were set to a capillary voltage of 2.8 kV, dry temperature of 200°C, nebulizer gas of 2.8 bar, and dry gas flow of 9.0 L/min. MS acquisition was recorded in data-dependent acquisition mode. MS events were composed of a full MS scan at 100−1200 Da for 3.5 Hz followed by MS^2 scans of the three most intense ions above 5000 counts for 12.0 Hz. Medium-pressure column chromatography (MPLC) was performed with a BÜCHI Pure C-850 FlashPrep system. Preparative and semipreparative HPLC were performed with an Agilent 1260 Infinity II system.

Optical rotations were measured on a JASCO P-1020 spectropolarimeter equipped with a 5 cm path-length cell. UV spectra were recorded on a JASCO V-630 spectrophotometer using a 10 mm path-length cell. ECD spectra were recorded on a JASCO J-810 spectropolarimeter using a 1 mm path precision cell at 25°C. NMR spectra were recorded on a Bruker Avance III HD 500, Avance III HD 400, or DRX 600 spectrometer. Chemical shifts (δ) were acquired in ppm with reference to $CHCl_3$ (δ 7.26 for ^1H NMR), $CDCl_3$ (δ 77.16 for $^{13}C\{^1H\}$ NMR), $(CHD_2)(CD_3)CO$ (δ 2.05 for ^1H NMR), $(CD_3)_2CO$ (δ 29.84 for $^{13}C\{^1H\}$ NMR), C_6D_5H (δ 7.16 for ^1H NMR), and C_6D_6 (δ 128.06 for $^{13}C\{^1H\}$ NMR). Signal patterns are indicated as s, singlet; d, doublet; t, triplet; q, quartet; m, multiplet; br, broaden signal. Structural assignments were performed using additional information from gCOSY, gHSQC, gHMBC, gNOESY, and gROESY experiments. HRMS data were recorded using a Bruker impact II QTOF mass spectrometer in positive or negative mode equipped with a Thermo Scientific Dionex Ultimate 3000 UHPLC system.

Bioinformatics analysis

A pHMM of Pyr4-like TCs has recently become publicly available (PF25129). However, the seed sequences used for model construction are solely of fungal origin. To obtain bacterial Pyr4-like TC sequences with higher confidence, we constructed a custom pHMM based on 62 previously identified Pyr4-like TCs including six of bacterial origin (Table S1). A multiple sequence alignment of known 62 Pyr4-like TCs from fungi and bacteria was performed using MUSCLE 5.2[6] and then converted into a pHMM using the hmmbuild tool in the HMMER 3.4 software package.[7] The custom pHMM was used to search Pyr4-like TC candidates from the NCBI non-redundant database restricted to bacterial taxa using the hmmsearch tool in the HMMER 3.4 with an *e*-value of $1e^{-5}$. After removal of sequences over 95% sequence identity using CD-HIT v4.8.1,[8] 1107 sequences were obtained. Recent studies have suggested that a subset of Pyr4-

like proteins is unlikely involved in meroterpenoid biosynthesis, as their encoding genes appear as standalone loci rather than within BGCs.[9] Guided by this observation, we filtered candidates based on genomic neighborhood context by the presence of terpenoid biosynthesis-related genes. For each candidate protein, genomic coordinates were obtained from the NCBI Identical Protein Groups (IPG) database, and ±20 kb flanking regions were extracted from the corresponding nucleotide records using Entrez Programming Utilities (E-utilities). Subsequently, proteins encoded in neighboring genes were analyzed by pHMMs for polyprenyl synthases (PF00348), prenyltransferases and squalene oxidase repeat-containing proteins (PF00432), squalene/phytoene synthases (PF00494), UbiA prenyltransferases (PF01040), aromatic prenyltransferases (PF11468), squalene-hopene cyclases (PF13242), and type I terpene synthases (PF19086) using the hmmscan tool in the HMMER 3.4. Out of 1107 candidates, the flanking region of 264 candidates encode at least one terpenoid biosynthesis-related enzymes. A sequence similarity network of 264 sequences was constructed by all-versus-all BLASTp comparison following the procedure described previously.[10] The constructed SSN at an *e*-value of 10^{-60} was visualized by Cytoscape v3.10.3.[11]

To infer a maximum-likelihood phylogenetic tree, sequences of 264 Pyr4-like TCs and four fungal Pyr4-like TCs (Pyr4, XP_751270.1; PaxB, ADO29934.1; MacJ, QBC75443.1; Cle7, BBG28477.1) were aligned using MAFFT v.7.526[12] with the L-INS-I method and then trimmed using trimAl v1.5.rev0[13] with the gappyout method. A maximum-likelihood phylogenetic tree was constructed by IQ-TREE v3.0.1[14] using the LG+F+R7 as a best fit model according to the Bayesian Information Criterion.[15] Branch supports were estimated using 10000 ultrafast bootstrap approximation.[16] Pyr4, PaxB, MacJ, and Cle7 were used as outgroups for generating the phylogenetic tree. The tree was visualized using iTOL v7.5.[17]

For the synteny analysis of BGCs encoding ZigF or its homologs clustered together in the SSN, global alignments of gene clusters and visualization of the cluster architecture were performed in clinker v0.0.32.[18] Predicted protein structures were generated using AlphaFold 3.[19] Multiple sequence alignments were visualized in JalView v2.11.5.1.[20]

Strains and culture conditions

M. zingiberis PLAI 1-1 was grown on N-Z-Amine agar (D-glucose 10 g/L, soluble starch 20 g/L, yeast extract 5 g/L, N-Z-Amine A 5 g/L, $CaCO_3$ 1 g/L, Agar 15 g/L, pH 7.2), and cultured in tryptic soy broth (TSB) medium for genomic DNA extraction at 30°C. *E. coli* DH5α, *E. coli* ET12567/pUZ8002, and *E. coli* Rosetta(DE3)pLysS were used for plasmid cloning, intergenic conjugation, and protein expression, respectively. *E. coli* strains were grown in Luria-Bertani (LB) broth, LB agar, or Terrific Broth (TB) medium supplemented with the appropriate antibiotics; apramycin (50 μg/mL), kanamycin (50 μg/mL), and chloramphenicol (25 μg/mL). *S. albus* J1074, *S. lividans* TK64, *S. coelicolor* M1154, and their recombinant strains were grown on MS agar (D-mannitol 20 g/L, soya flour 20 g/L, agar 20 g/L, pH 7.3). The fermentation of recombinant *Streptomyces* strains was conducted in PTMM (dextrin 40 g/L, α-lactose 40 g/L, yeast extract 5 g/L, MOPS 20 g/L, trace element solution 10 mL/L, pH 7.3; trace element solution, $ZnCl_2$ 40 mg/L, $FeCl_3 \cdot 6H_2O$ 200 mg/L, $CuCl_2$ 10 mg/L, $MnCl_2 \cdot 4H_2O$ 10 mg/L, $Na_2B_4O_7 \cdot 10H_2O$ 10mg/L, $(NH_4)_6Mo_7O_{24} \cdot 4H_2O$ 10 mg/L). For small-scale fermentations, fresh spores of recombinant *Streptomyces* strains were inoculated into 250 mL Erlenmeyer flasks containing PTMM (50 mL) and cultivated at 30°C for 7 days on a rotary shaker (200 rpm). For large-scale fermentations, fresh spores of recombinant strains grown on MS agar for 6–7 days were inoculated into 1 L Erlenmeyer flasks containing PTMM (200 mL) and cultivated at 30°C for 7 days on a rotary shaker (200 rpm). Small-scale fermentations of *M. zingiberis* were conducted in TSB, PTMM, F (sucrose 20 g/L, D-glucose 10g/L, casamino acids 0.1g/L, yeast extract

5 g/L, K_2SO_4 0.25 g/L, $MgCl_2·6H_2O$ 1 g/L, trace element solution 1 mL/L, pH 7.3), ISM3 (yeast extract 15 g/L, malt extract 10 g/L, D-glucose 20g/L, $FeCl_3·6H_2O$ 0.3 g/L, pH 7.0), or XTM (yeast extract 10 g/L, malt extract 10 g/L, D-maltose 10 g/L, trace element solution 10 mL/L, pH 7.2). Mycelia of *M. zingiberis* grown on N-Z-Amine agar for 7 days were inoculated into 250 mL Erlenmeyer flasks containing fermentation media (50 mL) and cultivated at 30°C for 7 days on a rotary shaker (200 rpm).

Cloning of the *zig* biosynthetic genes

For heterologous expression of the *zig* BGC in *Streptomyces*, a pSET152-based plasmid was constructed by introducing the strong constitutive promoter SP44 and ribosome binding site SR41.[21] The SP44–SR41 cassette, amplified using primer pairs SP44SR41-F/R (Table S4), was assembled with pSET152 linearized using primer pairs pSET152-SP44-F/R by HiFi assembly to obtain pSET152-SP44-SR41 (Table S5).

The *zig* BGC (ca. 16.6 kb) was assembled in two steps. First, the *zig* BGC was divided into four fragments (f1–f4) and amplified from genomic DNA of *M. zingiberis* PLAI 1-1 using primer pairs *zig*-f1-F/R, *zig*-f2-F/R, *zig*-f3-F/R, and *zig*-f4-F/R. The plasmid pSET152-SP44-SR41 was PCR-linearized using primer pairs pSET152-inv-F/R and treated with *Dpn*I. The linearized vector was assembled with fragments f1 and f2 by HiFi assembly to obtain pSET152-*zig*-f1-f2. In the second step, pSET152-*zig*-f1-f2 was digested with *Eco*RI and assembled with fragments f3 and f4 by HiFi assembly to obtain pSET152-*zig*.

For plasmid construction of the Δ*zigA* variant, four fragments were amplified using primer pairs Δ*zigA*-f1-F/*zig*-f1-R, *zig*-f2-F/R, *zig*-f3-F/R, and *zig*-f4-F/R from *M. zingiberis* genomic DNA. Plasmid assembly was performed as described above to yield pSET152-*zig*Δ*zigA*.

For the construction of the *zig* BGC expression plasmids with in-flame deletion of *zigC*, *zigD*, or *zigE*, fragments were amplified using primer pairs *zig*-f1-F/Δ*zigX*-f1-R (*X* = *C*, *D*, *E*), Δ*zigX*-f2-F/*zig*-f2-R (*X* = *C*, *D*, *E*), *zig*-f3-F/R, and *zig*-f4-F/R (Table S4) from *M. zingiberis* genomic DNA. HiFi assembly of fragments with a linearized pSET152-SP44-SR41 followed same procedure as described above to obtain pSET152-*zig*Δ*zigC*, pSET152-*zig*Δ*zigD*, and pSET152-*zig*Δ*zigE*.

For the construction of the *zig* BGC expression plasmids with in-flame deletion of *zigF*, *zigG*, or *zigH*, fragments (f1–f4) were amplified using primer pairs *zig*-f1-F/R, *zig*-f2-F/Δ*zigX*-f2-R (*X* = *F*, *G*, *H*), Δ*zigX*-f3-F/*zig*-f3-R (X = *F*, *G*, *H*), and *zig*-f4-F/R from *M. zingiberis* genomic DNA. The plasmid pSET152-SP44-SR41 was PCR-linearized using primer pairs pSET152-Δ*zigX*-inv-F/pSET152-inv-R (*X* = *F*, *G*, *H*) and treated with *Dpn*I. The linearized vectors were assembled with fragments f1 and f2 by HiFi assembly to obtain plasmids pSET152-*zig*Δ*zigX*-f1-f2 (*X* = *F*, *G*, *H*). The assembled plasmids pSET152-*zig*Δ*zigX*-f1-f2 (*X* = *F*, *G*, *H*) were individually digested with *Eco*RI and assembled with fragments f3 and f4 by HiFi assembly to obtain pSET152-*zig*Δ*zigF*, pSET152-*zig*Δ*zigG*, and pSET152-*zig*Δ*zigH*, respectively.

For the construction of the *zig* BGC expression plasmids with in-flame deletion of *zigI*, *zigJ*, *zigK*, *zigL*, or *zigM*, fragments (f3, f4) were amplified using primer pairs *zig*-f3-F/Δ*zigX*-f3-R and Δ*zigX*-f4-F/*zig*-f4-R (X = *I*, *J*, *K*, *L*, *M*) from genomic DNA of *M. zingiberis*. The plasmid pSET152-*zig*-f1-f2 was digested with *Eco*RI and assembled with fragments f3 and f4 by HiFi assembly to obtain pSET152-*zig*Δ*zigI*, pSET152-*zig*Δ*zigJ*, pSET152-*zig*Δ*zigK*, pSET152-*zig*Δ*zigL*, and pSET152-*zig*Δ*zigM*, respectively.

For plasmid construction of the Δ*zigN* variant, fragments were amplified using primer pairs *zig*-f3-F/R and *zig*-f4-F/Δ*zigN*-f4-R from *M. zingiberis* genomic DNA. The plasmid pSET152-*zig*-f1-f2 was digested with *Eco*RI and subsequently assembled with the fragments by HiFi assembly to obtain pSET152-*zig*Δ*zigN*.

For plasmid construction of the Δ*zigLN* variant, a fragment was amplified using primer pairs *zig*-f3-F/Δ*zigN*-f4-R from plasmid DNA of pSET152-*zig*Δ*zigM*. The plasmid pSET152-*zig*-f1-f2 was digested with *Eco*RI and subsequently assembled with the fragment by HiFi assembly to obtain pSET152-*zig*Δ*zigLN*.

For construction of a *zigE* expression plasmid, *zigE* was amplified using primer pairs *zigE*-F/R from *M. zingiberis* genomic DNA. The plasmid pSET152-SP44-SR41 was digested with *Not*I and *Eco*RV and subsequently assembled with the *zigE* fragment by HiFi assembly.

For construction of a *zigF* expression plasmid, *zigF* was amplified using primer pairs *zigF*-F/R from *M. zingiberis* genomic DNA. The plasmid pET-28c(+) was digested with *Nco*I and *Eco*RI and subsequently assembled with the *zigF* fragment by HiFi assembly.

The sequences of all plasmids were confirmed by DNA sequencing.

Heterologous expression and metabolite analysis

The expression plasmids harboring the *zig* BGC and its derivatives were introduced into *E. coli* ET12567/pUZ8002 and subsequently transferred into *S. albus* J1074, *S. lividans* TK64, or *S. coelicolor* M1154 by intergenic conjugation as described previously.[22] Briefly, *E. coli* ET12567/pUZ8002 carrying the plasmid was cultured in LB medium (40 mL) supplemented with appropriate antibiotics at 37°C on a rotary shaker (130 rpm) until an OD_{600} of 0.4–0.6. Cells were harvested, washed twice with LB medium (2 × 10 mL), and resuspended in LB medium (400 μL). The suspension was mixed with 2xYT medium (400 μL) containing *Streptomyces* spores and serially diluted 10-fold with LB medium. Aliquots (200 μL) were plated on MS agar containing 10 mM $MgCl_2$. After incubation at 30°C for 16–20 h, plates were overlaid with 1 mL of H_2O containing apramycin (1 mg) and nalidixic acid (0.5 mg), followed by further incubation at 30°C for 4–7 days. Exconjugant colonies were picked and streaked onto MS agar containing apramycin (50 μg/mL) and nalidixic acid (25 μg/mL) and incubated at 30°C for 4–7 days. Five positive clones were randomly selected for small-scale fermentation and metabolite analysis.

Small-scale fermentation of recombinant *Streptomyces* strains was performed in PTMM. After 7 days of cultivation, supernatant and mycelia were separated by centrifugation (3,810 × *g*, 10 min). An aliquot of supernatant (10 mL) was extracted with an equal volume of EtOAc, while the mycelial pellet was extracted with acetone (20 mL) under ultrasonication for 15 min. The EtOAc extract (2.5 mL) and acetone extract (1.0 mL) were combined and concentrated in vacuo. The resulting crude extract was dissolved in MeOH (1.0 mL) and subjected to LC-MS analysis.

Large-scale fermentation and isolation of 1–7, 9, 10, and 12

For the isolation of **1**–**5**, *S. albus*::*zig* was fermented in 11 L of PTMM broth. After 7 days of cultivation, supernatant and mycelium were separated by centrifugation (10826 × *g*, 15 min, 4°C). The supernatant was extracted with an equal volume of EtOAc for three times. The mycelium was extracted three times with acetone (3 L) under ultrasonication for 30 min. The acetone extract was concentrated in vacuo and resuspended in H_2O (1 L), followed by extraction with EtOAc (4 × 1 L). The extracts from supernatant and mycelia were combined and concentrated in vacuo to obtain a crude extract (2.42 g). The crude extract was

subjected to MPLC equipped with a FlashPure Select C_{18} column (80 g, 30 μm) eluting with MeOH–H_2O (5:95, 5 min; 5:95–100:0, 65 min; 100:0, 15 min; 45 mL/min) to yield 27 fractions (AN1–AN22). The fraction AN23 (354.7 mg) was separated by preparative HPLC equipped with a Luna C18(2) column (21.2 × 250 mm, 5 μm) eluted with MeCN–H_2O (85:15–95:5; 15 mL/min) to yield 11 subfractions (AN23a–AN23k). The subfraction AN23d (30.1 mg) was separated by semipreparative HPLC equipped with a COSMOSIL πNAP column (10 × 250 mm, 5 μm) eluted with MeOH–H_2O (93:7; 6 mL/min) to yield **1** (4.7 mg, t_R 17.2 min). The subfraction AN23c (99.5 mg) was separated by semipreparative HPLC equipped with a COSMOSIL πNAP column (250 × 10 mm, 5 μm) eluted with MeOH–H_2O (64:36; 6 mL/min) to yield **3** (5.8 mg, t_R 17.5 min). The fraction AN24 (49.0 mg) was purified by semipreparative HPLC equipped with a Luna C18(2) column (250 × 10 mm, 5 μm) eluted with MeCN–H_2O (88:12; 6 mL/min) to yield **2** (5.4 mg, t_R 21.6 min). The fraction AN22 (280.1 mg) was separated by preparative HPLC equipped with a Luna C18(2) column (250 × 21.2 mm, 5 μm) eluted with MeCN–H_2O (60:40–100:0; 15 mL/min) to yield **5** (1.7 mg, t_R 21.4 min) and a fraction containing **4** (8.9 mg). This fraction was further separated by semipreparative HPLC equipped with a COSMOSIL πNAP column (250 × 10 mm, 5 μm) eluted with MeOH–H_2O (64:36; 6 mL/min) to yield **4** (1.5 mg, t_R 19.1 min).

For the isolation of **6**, *S. albus*::*zig*Δ*zigG* was fermented in 14 L of PTMM broth. Following the extraction procedure described above, a crude extract of supernatant and mycelium (2.48 g) was obtained. The crude extract was subjected to MPLC equipped with a a FlashPure Select C_{18} column (80 g, 30 μm) eluted with MeOH–H_2O (15:85, 5min, 15:85–100:0, 55 min; 100:0, 15 min; 45 mL/min) to yield 11 fractions. A fraction containing **6** (354.1 mg) was separated by preparative HPLC equipped with a Luna C18(2) column (250 × 21.2 mm, 5 μm) eluted with MeCN–H_2O (90:10; 15 mL/min), followed by semipreparative HPLC equipped with a COSMOSIL πNAP column (250 × 10 mm, 5 μm) eluted with MeOH–H_2O (89:11; 6 mL/min) to yield **6** (2.1 mg, t_R 26.5 min).

For the isolation of **7**, *S. albus*::*zig*Δ*zigK* was fermented in 14 L of PTMM broth. Following the extraction procedure described above, a crude extract of supernatant and mycelium (2.49 g) was obtained. The crude extract was subjected to MPLC equipped with a FlashPure Select C_{18} column (80 g, 30 μm) eluted with MeOH–H_2O (15:85, 5min, 15:85–100:0, 55 min; 100:0, 15 min; 45 mL/min) to yield 16 fractions. A fraction containing **7** (48.0 mg) was separated by semipreparative HPLC equipped with a COSMOSIL πNAP column (250 × 10 mm, 5 μm) eluted with MeOH–H_2O (87:13; 6 mL/min), followed by semipreparative HPLC equipped with a Luna C18(2) column (250 × 10 mm, 5 μm) eluted with MeCN–H_2O (87:13; 6 mL/min) to yield **7** (1.1 mg, t_R 23.9 min).

For the isolation of **9**, *S. albus*::*zig*Δ*zigE* was fermented in 12 L of PTMM broth. Following the extraction procedure described above, a crude extract of supernatant and mycelium (2.40 g) was obtained. The crude extract was subjected to silica gel flash column chromatography eluted with *n*-hexane–EtOAc (10:1, 4:1, 3;1, 2:1, 1:1, 0:1, each 1 L), followed by CH_2Cl_2–MeOH (9:1, 8:1, 6:1, 4:1, each 1 L) to yield 10 fractions. The fraction containing **9** (644.7 mg) was separated by MPLC equipped with a FlashPure EcoFlex C18 column (20 g, 50 μm) eluted with MeCN–H_2O (35:80–100:0, 50 min; 100:0, 5 min; 32 mL/min), followed by preparative HPLC equipped with a Luna C18(2) column (250 × 21.2 mm, 5 μm) eluted with MeCN–H_2O (97:3; 15 mL/min) with 0.1% formic acid to yield **9** (36.8 mg, t_R 13.0 min).

For the isolation of **10**, *S. albus*::*zig*Δ*zigD* was fermented in 12 L of PTMM broth. Following the culture extraction procedure described above, a crude extract of supernatant and mycelium (1.85 g) was obtained. The crude extract was subjected to MPLC equipped with a FlashPure Select C_{18} column (80 g, 30 μm) eluted with MeOH–H_2O (15:85, 5min, 15:85–100:0, 55 min; 100:0, 15 min; 45 mL/min) to yield 12

fractions. The fraction containing **10** (432.4 mg) was separated by preparative HPLC equipped with a Luna C18(2) column (250 × 21.2 mm, 5 μm) eluted with MeCN–H_2O (86:14; 15 mL/min), followed by semipreparative HPLC equipped with a COSMOSIL πNAP column (250 × 10 mm, 5 μm) eluted with MeOH–H_2O (87:13; 6 mL/min) to yield **10** (5.8 mg, t_R 19.2 min).

For the isolation of **12**, *S. albus*::*zig*Δ*zigI* was fermented in 13 L of PTMM broth. Following the extraction procedure described above, a crude extract of supernatant and mycelium (2.87 g) was obtained. The crude extract was subjected to MPLC equipped with a FlashPure Select C_{18} column (80 g, 30 μm) eluted with MeOH–H_2O (15:85, 5min, 15:85–100:0, 55 min; 100:0, 15 min; 45 mL/min) to yield 9 fractions (I1–I9). The fraction I9 (307.9 mg) was separated by preparative HPLC equipped with a Luna C18(2) column (250 × 21.2 mm, 5 μm) eluted with MeCN–H_2O (92:8; 15 mL/min), followed by semipreparative HPLC equipped with a COSMOSIL πNAP column (250 × 10 mm, 5 μm) eluted with MeCN–H_2O (67:37; 6 mL/min) to afford a fraction containing **12** (34.4 mg). This fraction was further separated by a semipreparative HPLC equipped with a COSMOSIL πNAP column (250 × 10 mm, 5 μm) eluted with MeOH–H_2O (82:18; 6 mL/min) to yield **12** (2.9 mg, t_R 24 min).

Physical and spectroscopic properties of 1–7, 9, 10, and 12

Zigralone A (**1**): white solid; $[\alpha]_D^{20}$ −10.1 (*c* 0.1, $CHCl_3$); UV (MeOH) λ_{max} (log ε) 237 (4.54), 268 (sh, 3.57), 297 (3.67), 325 (3.96), 338 (sh, 3.92) nm; ECD (*c* 0.6 mM, MeOH) λ_{max} ($\Delta\varepsilon$) 197 (−5.0), 205 (−5.5), 220 (sh, +2.1), 237 (+7.3), 266 (+1.4), 292 (+1.0), 328 (−3.0), 337 (sh, −2.9) nm; ^1H and $^{13}C\{^1H\}$ NMR data, see Table S7; HRMS (ESI/QTOF) *m/z*: $[M + H]^+$ calcd for $C_{31}H_{42}NO_6^+$, 524.3007, found 524.3007.

Zigralone B (**2**): colorless prism; $[\alpha]_D^{20}$ +33.1 (*c* 0.1, $CHCl_3$); UV (MeOH) λ_{max} (log ε) 216 (4.35), 244 (4.35), 264 (4.50), 382 (3.85) nm; ECD (*c* 0.6 mM, MeOH) λ_{max} ($\Delta\varepsilon$) 200 (−2.2), 213 (+3.3), 235 (+2.6), 260 (−3.2), 273 (+1.7), 299 (+0.2), 353 (−2.7), 401 (+2.2) nm; ^1H and $^{13}C\{^1H\}$ NMR data, see Table S8; HRMS (ESI/QTOF) *m/z*: $[M + H]^+$ calcd for $C_{31}H_{42}NO_5^+$, 508.3057, found 508.3057.

Zigralone C (**3**): yellow gum; $[\alpha]_D^{20}$ −13.3 (*c* 0.1, $CHCl_3$); UV (MeOH) λ_{max} (log ε) 201 (4.09), 232 (4.36), 276 (3.68), 376 (3.53) nm; ECD (*c* 0.6 mM, MeOH) λ_{max} ($\Delta\varepsilon$) 196 (−11.2), 213 (−2.2), 231 (−1.0), 249 (−1.0), 276 (+2.2), 326 (−3.3), 368 (+2.5) nm; ^1H and $^{13}C\{^1H\}$ NMR data, see Table S9; HRMS (ESI/QTOF) *m/z*: $[M + H - H_2O]^+$ calcd for $C_{31}H_{44}NO_5^+$, 510.3214, found 510.3214, $[M + Na]^+$ calcd for $C_{31}H_{45}NO_6Na^+$, 550.3139, found 550.3135.

Zigralone D (**4**): colorless oil; $[\alpha]_D^{20}$ +19.7 (*c* 0.03, acetone); UV (MeCN) λ_{max} (log ε) 230 (4.51) nm, 297 (3.88), 320 (4.04), 332 (4.01); ECD (*c* 0.5 mM, MeCN) λ_{max} ($\Delta\varepsilon$) 205 (+4.4), 225 (−4.7), 296 (−0.4), 336 (+0.3) nm; ^1H and $^{13}C\{^1H\}$ NMR data, see Table S10; HRMS (ESI/QTOF) *m/z*: $[M + H]^+$ calcd for $C_{31}H_{44}NO_4^+$, 494.3265, found 494.3265.

Zigralone E (**5**): yellow gum; $[\alpha]_D^{20}$ +15.3 (*c* 0.2, acetone); UV (MeOH) λ_{max} (log ε) 203 (4.40), 248 (4.60), 340 (4.02), 355 (4.00) nm; ECD (*c* 0.5 mM, MeOH) λ_{max} ($\Delta\varepsilon$) 200 (−4.3), 210 (−4.5), 248 (+5.2), 304 (+0.9), 340 (+0.4) nm; ^1H and $^{13}C\{^1H\}$ NMR data, see Table S11; HRMS (ESI/QTOF) *m/z*: $[M + H]^+$ calcd for $C_{30}H_{42}NO_4^+$, 480.3108, found 480.3108.

Zigralone F (**6**): pale-brown solid; $[\alpha]_D^{20}$ −76.7 (*c* 0.2, $CHCl_3$); UV (MeOH) λ_{max} (log ε) 232 (sh, 4.47), 238 (4.53), 322 (3.90), 334 (3.85) nm; ECD (*c* 0.5 mM, MeOH) λ_{max} ($\Delta\varepsilon$) 201 (−0.3), 226 (−2.5), 242 (+1.0), 264 (+0.4), 282 (+0.5), 322 (−1.3), 333 (−1.0) nm; ^1H and $^{13}C\{^1H\}$ NMR data, see Table S13; HRMS (ESI/QTOF) *m/z*: $[M + H]^+$ calcd for $C_{31}H_{44}NO_4^+$, 494.3265, found 494.3265.

Zigralone G (**7**): colorless gum; ^{1}H and $^{13}C\{^{1}H\}$ NMR data, see Table S14; HRMS (ESI/QTOF) *m/z*: $[M + H]^+$ calcd for $C_{31}H_{44}NO_2^+$, 462.3367, found 462.3367.

Prezigralone G (**9**): dark yellow oil; $[\alpha]_D^{20}$ +100.4 (*c* 1.0, $CHCl_3$); UV (MeOH) λ_{max} (log ε) 205 (4.58), 224 (4.62), 269 (3.88) nm; ECD (*c* 0.4 mM, MeOH) λ_{max} ($\Delta\varepsilon$) 196 (+6.1), 203 (+0.9), 227 (−7.6), 292 (−1.1), 347 (+2.1); ^{1}H and $^{13}C\{^{1}H\}$ NMR data, see Table S15; HRMS (ESI/QTOF) *m/z*: $[M - H]^-$ calcd for $C_{33}H_{44}NO_4^-$, 518.3276, found 518.3271.

2-Geranylgeranyl-7-methoxy-1*H*-indole-3-carboxaldehyde (**10**): pale-brown solid; ^{1}H and $^{13}C\{^{1}H\}$ NMR data, see Table S16; HRMS (ESI/QTOF) *m/z*: $[M + H]^+$ calcd for $C_{30}H_{42}NO_2^+$, 448.3210, found 448.3210.

4-(Geranylgeranyl(hydroxy)methyl)quinaldic acid (**12**): colorless gum; $[\alpha]_D^{20}$ +41.7 (*c* 0.3, $CHCl_3$); UV (MeCN) λ_{max} (log ε) 201 (4.56), 240 (4.52), 292 (3.65) nm; ECD (*c* 0.5 mM, MeCN) λ_{max} ($\Delta\varepsilon$) 197 (+2.1), 213 (−1.2), 236 (+2.4), 252 (−0.4), 295 (+0.9), 334 (−0.2) nm; ^{1}H and $^{13}C\{^{1}H\}$ NMR data, see Table S17; HRMS (ESI/QTOF) *m/z*: $[M + H]^+$ calcd for $C_{31}H_{42}NO_3^+$, 476.3159, found 476.3160.

Single crystal X-ray diffraction analysis

Single crystals of **2** were grown in a mixture of MeCN–H_2O (ca. 95:5) by a slow evaporation process at room temperature. Single crystal X-ray diffraction data was collected at 173K on a STOE IPDS two-circle diffractometer equipped with a Genix 3D IμS microfocus X-ray source using Mo *K*α radiation (λ = 0.71073 Å). The finalization of the data, including empirical absorption corrections, was performed using the CrysAlisPro v.1.171.42.43a software.[23] The structure was solved using the SHELXT program[24] and refined against $|F|^2$ with full-matrix least-squares techniques using the SHELXL2019/2 program.[25] Anisotropic atomic displacement parameters were introduced for all non-hydrogen atoms. All hydrogen atoms were located geometrically and refined riding on the pivot atom.

General computational methods

Conformational searches were performed using the Spartan'20 v1.1.4 program[26] employing the Merk Molecular Force Field (MMFF)[27] within an energy window of 10 kcal/mol. All DFT and TD-DFT calculations were carried out using the Gaussian 16 Rev.C.02 program.[28] Calculated ECD spectra were visualized and compared with experimental ECD spectra using the SpecDis v1.71 program.[29]

ECD calculations

To elucidate the absolute configuration of **1**, conformational searches of (5*S*,8*R*,9*R*,10*S*,13*S*,14*R*,15*R*)-**1** and (5*S*,8*R*,9*R*,10*S*,13*S*,14*R*,15*S*)-**1** were performed to afford 12 and 7 conformers, respectively. The geometries of the obtained conformers were optimized at the M06-2X[30]/def2-SVP[31] level of theory with the solvation model based on density (SMD)[32] for MeOH. After removal of duplicated conformers, low-lying conformers within an energy window of 5 kcal/mol were further optimized at M06-2X/def2-TZVP/SMD(MeOH) level of theory. Frequency calculations were performed at the same level to confirm the absence of imaginary frequencies and to obtain thermal corrections. Energy evaluation at the M06-2X/def2-TZVP/SMD(MeOH) level afforded two conformers for each diastereomer with Boltzmann populations greater than 1%. The excitation energy, rotator strength, dipole velocity, and dipole length of the conformers were calculated by TD-DFT at the *ω*B97X-D[33]/aug-cc-pVTZ[34]/SMD(MeOH) level of

theory. The number of excited states for each conformer was set to 40. Boltzmann-weighted ECD spectra were generated using a Gaussian band shape with a bandwidth of 0.30 eV and UV correction of +16 nm.

To elucidate the absolute configuration of **2**, conformational search of (5*S*,8*R*,9*R*,10*S*,13*S*,14*R*)-**2** was performed to afford 14 conformers. The geometries of the obtained conformers were optimized at the PBE0[35]/def2-SVP level of theory with the conductor-like polarizable continuum model (CPCM)[36] for MeOH. After removal of duplicated conformers, 10 conformers were further optimized at the PBE0/def2-TZVP/CPCM(MeOH) level of theory. Frequency calculations were performed at the same level of theory to confirm the absence of imaginary frequencies and to obtain thermal corrections. Single-point energies were calculated at the PBE0-D3(BJ)[37]/def2-TZVPP/CPCM(MeOH) level of theory. TD-DFT calculations of excitation energies, rotator strengths, dipole velocities, and dipole lengths were performed for two conformers with Boltzmann populations greater than 1% at the ωB97X-D/aug-cc-pVTZ/CPCM(MeOH) level of theory. Forty excited states were calculated for each conformer. A Boltzmann-weighted ECD spectrum was generated using a Gaussian band shape with a bandwidth of 0.28 eV and UV correction of +20 nm.

To elucidate the absolute configuration of **4**, a simplified model **4a** was constructed by replacing the farnesyl group with a methyl group to reduce the computational cost. Conformational search of (2′*R*,3′*R*)-**4a** yielded 56 conformers. The geometries of these conformers were optimized at the M06-2X/def2-SVP level of theory, followed by frequency calculations at the same level of theory to confirm the absence of imaginary frequencies and to obtain thermal corrections. Single-point energy calculations were performed at the M06-2X/def2-TZVPP level of theory. After removal of duplicate conformers, TD-DFT calculations of excitation energies, rotator strengths, dipole velocities, and dipole lengths were carried out for 12 conformers with Boltzmann populations greater than 1% at the ωB97X-D/def2-TZVPP/SMD(MeCN) level of theory. Forty excited states were calculated for each conformer. A Boltzmann-weighted ECD spectrum was generated using a Gaussian band shape with a bandwidth of 0.28 eV and UV correction of +13 nm.

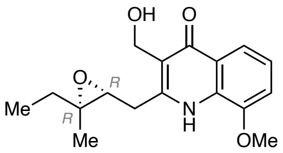

4a

To elucidate the absolute configuration of **5**, a simplified model **5a** was constructed by replacing the farnesyl side chain with a methyl group to reduce the computational cost. Conformational search of (2′*R*,3′*S*)-**5a** yielded 17 conformers. The geometries of these conformers were optimized at the M06-2X/def2-TZVP/SMD(MeOH) level of theory. Frequency calculations were performed at the same level of theory to confirm the absence of imaginary frequencies and to obtain thermal corrections. After removal of duplicate conformers, TD-DFT calculations of excitation energies, rotator strengths, dipole velocities, and dipole lengths were performed for 12 conformers with Boltzmann populations greater than 1% at the ωB97X-D/def2-TZVPP/SMD(MeOH) level of theory. Forty excited states were calculated for each conformer. A Boltzmann-weighted ECD spectrum was generated using a Gaussian band shape with a bandwidth of 0.28 eV and UV correction of +19 nm.

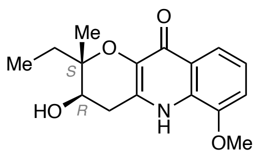

5a

To elucidate the absolute configuration of **9**, a simplified model of **9a** was constructed by replacing the geranylgeranyl side chain with a prenyl group to reduce the computational cost. Conformational search of (*R*)-**9a** yielded 37 conformers. The geometries of these conformers were optimized at the M06-2X/def2-TZVP/SMD(MeOH) level of theory. Frequency calculations were performed at the same level of theory to confirm the absence of imaginary frequencies and to obtain thermal corrections. After removal of duplicate conformers, the excitation energy, rotator strength, dipole velocity, and dipole length of 16 conformers with Boltzmann populations greater than 1% were calculated by TD-DFT at the *ω*B97X-D/def2-TZVPP/SMD(MeOH) level of theory. The number of excited states for each conformer was set to 40. Boltzmann-weighted ECD spectra were generated using a Gaussian band shape (0.26 eV) and UV correction (+17 nm).

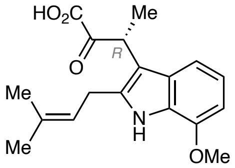

9a

DFT calculations to investigate the nonenzymatic formation of 1 from 8

Geometry optimizations were carried out at the *ω*B97X-D/def2-SVP/SMD(H_2O) level of theory without symmetry restrictions. Frequency calculations were performed at the same level of theory to confirm each stationary point as either a local minimum (no imaginary frequency) or a transition state (one imaginary frequency) and to obtain thermal corrections. Intrinsic reaction coordinate (IRC) calculations[38] were performed to verify the connectivity of each transition state to the corresponding reactants and products. Single-point energy calculations were performed at the *ω*B97X-D/def2-TZVPP/SMD(H_2O) level of theory. Quashi-harmonic corrections to enthalpy and entropy were applied using the Grimme's method[39] with a frequency cutoff of 100 cm^{-1}, as implemented in the GoodVibes v3.2 program.[40] Relative Gibbs free energies (ΔG, 298.15K, 1 atm) are reported in kcal/mol.

In vitro assay of ZigF

The expression plasmid pET28-*zigF* was transferred into *E. coli* Rosetta(DE3)pLysS. Transformants were cultivated in 1 L Erlenmeyer flasks containing TB medium (200 mL) supplemented with 50 μg/mL kanamycin and 25 μg/mL chloramphenicol. Cultures (total volume, 1 L) were grown at 37°C with shaking at 200 rpm until an OD_{600} of 0.6–0.8 was reached. The cultures were cooled on ice for 10 min and induced with 0.2 mM isopropyl *β*-D-thiogalactopyranoside (IPTG). After cultivation at 16°C for 20 h with shaking at 200 rpm, cells were harvested by centrifugation (4000 × *g*, 10 min, 4°C). The cell pellet was washed twice with 50mM KPi buffer (pH 7.5) and resuspended in 15 mL of the same buffer. Cell disruption was performed by sonication using a 0.5-inch probe (35% amplitude; 1s on, 3 s off; total sonication 7 min). Cell debris was removed by centrifugation (7000 × *g*, 15 min, 4°C). The resulting supernatant was used directly

for *in vitro* reactions with **4**. A control cell lysate was prepared from *E. coli* Rosetta(DE3)pLysS harboring pET-28c(+) in the same manner.

For the enzymatic assay, **4** (20 mM in DMSO, 1 μL) was added to cell lysate (49 μL) and the reaction mixture was incubated at 30°C for 18 h with shaking at 300 rpm. Reactions were quenched by addition of MeOH (50 μL), followed by centrifugation (17000 × *g*, 20 min). The supernatant (25 μL) was diluted with MeOH (75 μL) and analyzed by LC-MS.

Biotransformation of 9 in *S. albus*::*zigE*

Flesh spores of recombinant *S. albus* strains harboring pSET152-*zigE* or the empty vector pSET152-SP44-SR41 were inoculated into PTMM (20 mL) in 100 mL Erlenmeyer flasks and cultured at 30°C for 2 days with shaking (200 rpm). Compound **9** (0.5 mg in 20 μL of DMSO) was added to the cultures (final concentration, 48 μM), followed by incubation at 30°C for an additional 24 h with shaking (200 rpm). After centrifugation (3810 × *g*, 10 min) of cultures, aliquot of supernatant (10 mL) was extracted with an equal volume of EtOAc, while the cell pellet was extracted with acetone (8.0 mL) under ultrasonication for 15 min. The EtOAc extract (2.5 mL) and acetone extract (1.0 mL) were combined and concentrated in vacuo. The resulting crude extract was dissolved in MeOH (0.4 mL) and subjected to LC-MS analysis.

Antibacterial activity assay

Enterococcus faecium DSM 25390, *Staphylococcus aureus* DSM 2569, *Klebsiella pneumoniae* DSM 109340, *Pseudomonas aeruginosa* DSM 19880, and *Enterobacter cloacae* DSM 30054 were obtained from the German Collection of Microorganisms and Cell Cultures (DSMZ). *Acinetobacter baumannii* ATCC 19606 was provided by Prof. Volker Müller, Institute of Molecular Biosciences, Goethe University Frankfurt, Germany. Antibacterial activity of **1–3** was evaluated by minimum inhibitory concentrations (MICs) using the broth dilution method as described previously.[41] Briefly, serial two-fold dilutions of compounds (128–0.25 μg/mL) were prepared in 50 μL of Mueller–Hinton broth (MHB) medium in 96-well plates. Equal volume of the bacterial suspension was added and incubated at 37°C for 20 h. The MIC was determined as the lowest compound concentration that inhibited 90% of bacterial growth. Apramycin was used as a positive control. The assay was performed in biological triplicates.

Structure characterization of 1–7, 9, 10, and 12

<u>Compound **1**</u>

Compound **1** was isolated as a white solid. Its molecular formula was determined as $C_{31}H_{41}NO_6$ based on a protonated molecular ion at *m/z* 524.3007 (calcd. for $C_{31}H_{42}NO_6^+$, 524.3007) in HRMS data. The UV spectrum of **1** showed characteristic absorption maxima at 237, 325, and 335 nm, indicating the presence of a 4-quinolone chromophore. The ^{1}H NMR spectrum showed resonances for a secondary amino proton (δ_H 9.14), three aromatic protons corresponding to a 1,2,3-trisubstituted phenyl group (δ_H 7.88, 7.22, 7.01), a hydroxy proton (δ_H 5.30), two oxymethylenes exhibiting geminal spin coupling (δ_H 5.04, 4.55, 4.48, 3.87), an aryl methoxy group (δ_H 3.97), an oxymethine (δ_H 3.77), and four methyl singlets (δ_H 1.43, 0.87, 0.81, 0.80) (Table S7). Analysis of the ^{13}C and DEPT135 NMR spectra, with the aid of the HSQC spectrum, revealed the presence of a carbonyl carbon, five unprotonated sp^2 carbons, three protonated sp^2 carbons, a hemiketal, two oxygenated quaternary carbons, one oxymethine, two oxymethylenes, one methoxy carbon,

two sp^3 quaternary carbons, two aliphatic methines, seven aliphatic methylenes, and four methyls. The carbonyl and sp^2 carbons, together with the amino proton and three aromatic protons, are attributed to a trisubstituted 4-quinolone moiety. The 7-methoxy substitution of the quinolone moiety was assigned based on the HMBC correlations from the methoxy protons to C-8′ (δ_C 148.1) and from H-5′ (δ_H 7.88) to the quinolone carbonyl carbon. The HMBC correlations from H_2-9′ (δ_H 5.04, 4.55), H-14 (δ_H 3.77), and OH-15 (δ_H 5.30) to quinolone carbons revealed that the quinolone moiety is fused to a hemiketal-containing dihydropyran ring at C-2′/C-3′ (Table S7). The HMBC correlations from H_2-16 (δ_H 4.48, 3.87) to C-12 (δ_C 25.9), C-13 (δ_C 83.1), C-14 (δ_C 86.3), C-15 (δ_C 98.4) established the fusion of the dihydropyran ring to a tetrahydrofuran ring at C-14/C-15. Analysis of the COSY spectrum revealed the presence of three continuous spin systems (Table S7): H_2-1 (δ_H 1.64, 0.92)/H_2-2 (δ_H 1.62, 1.47)/H_2-3 (δ_H 1.40, 1.16); H-5 (δ_H 0.98)/H_2-6 (δ_H 1.71, 1.31)/H_2-7 (1.86, 1.46); and H_2-11 (δ_H 1.77, 1.41)/H_2-12 (δ_H 2.03, 1.98). Together with the HMBC correlations from methyl groups and H_2-12 to unassigned carbons (Table S7), these data supported the presence of a 6/6/6-tricyclic manoyl oxide-like skeleton. The HMBC correlations from H_2-12 to C-14 and C-16 further indicated that the manoyl oxide-like moiety is spiro-fused to the tetrahydrofuran ring via C-13, establishing the planar structure of **1** as a 6/6/6/5/6/6/6-heptacyclic scaffold.

The relative configuration of **1** was determined based on the NOESY correlations (Table S7). The NOESY correlations of H-5/H-9, H_b-2/H_3-19, H_b-2/H_3-17, and H_3-17/H_3-20 supported a *trans-anti-trans* fusion of the tricyclic manoyl oxide-like skeleton. The NOESY correlation between H-9 and H-14 indicated their cofacial relationship. The NOESY correlations of H_a-16/H_3-20, H-14/OH-15, and H_3-20/OH-15 suggested the *S**-configuration of the spiro center C-13 and the *cis*-fusion of a tetrahydrofuran–dihydropyran ring system. To further support the relative configuration at the hemiketal carbon C-15 and to determine the absolute configuration of **1**, we calculated theoretical ECD spectra of (5*S*,8*R*,9*R*,10*S*,13*S*,14*R*,15*R*)-**1** and (5*S*,8*R*,9*R*,10*S*,13*S*,14*R*,15*S*)-**1** using the TD-DFT method. The Boltzmann-weighted ECD spectrum of (5*S*,8*R*,9*R*,10*S*,13*S*,14*R*,15*R*)-**1** showed good agreement with the experimental ECD spectrum (Figure S6), thereby establishing the absolute configuration of **1**. Compound **1** was named zigralone A.

Compound **2**

Compound **2** was isolated as colorless prisms. Its molecular formula was determined as $C_{31}H_{41}NO_5$ based on a protonated molecular ion at *m/z* 508.3057 (calcd. for $C_{31}H_{42}NO_5^+$, 508.3057) in HRMS data. The ^{13}C NMR spectrum of **2** resembled that of **1**, but lacked signals for the hemiketal carbon C-15 and the oxymethylene C-16 in **1**. Instead, signals attributable to an *α*,*β*-unsaturated ketone (δ_C 193.3, C-15) and a methyl (δ_C 25.5, C-16) were observed. The HMBC correlations from H_3-16 (δ_H 1.57) to C-12 (δ_C 34.3), C-13 (δ_C 79.7), and C-14 (δ_C 86.0), and from H-14 (δ_H 3.95) to C-15 established a bilobed structure comprising a quinolone core fused to dihydro-3-pyrone and a manoyl oxide-type tricyclic system, linked via a C(sp^3)–C(sp^3) bond (Table S8). The NOESY correlations of H-5/H-9, H_3-17/H_3-20, and H_3-20/H_3-16 suggested that the relative stereochemistry at C-5, C-8, C-9, C-10, and C-13 in **2** is identical to those of **1**. The relative configuration of **2** was further determined to be (5*S**,8*R**,9*R**,10*S**,13*S**,14*R**) by single-crystal X-ray diffraction analysis using Mo Kα radiation (CCDC 2542513). The TD-DFT-calculated ECD spectrum of (5*S*,8*R*,9*R*,10*S*,13*S*,14*R*)-**2** showed good agreement with the experimental ECD spectrum of **2** (Figure S7), establishing its absolute configuration. Compound **2** was named zigralone B.

Compound **3**

Compound **3** was isolated as a yellow gum. Its molecular formula was determined as $C_{31}H_{45}NO_6$ based on a sodium adduct ion at *m/z* 550.3135 (calcd. for $C_{31}H_{45}NO_6Na^+$, 550.3139) in HRMS data. Comparison of the ^{13}C NMR data of **3** with those of **2** revealed the absence of olefinic carbons attributable to the

quinolone core, as well as one carbonyl carbon. Instead, resonances for two oxygenated quaternary carbon (δ_C 86.0, C-2′; δ_C 77.9, C-3′) and one methylene (δ_C 35.4, C-15) were observed (Table S9). The ^{1}H NMR spectrum exhibited two overlapping broad signals (δ_H 4.32, 4.33) for hydroxy protons. These 1D NMR data, together with the number of oxygen atoms from the molecular formula, suggested that **3** possesses a 2,3-dihydroxy-2,3-dihydro-4-quinolone moiety, in place of the 4-quinolone moiety, which is fused to a tetrahydropyran ring at C-2′/C-3′. This assignment was supported by the HMBC correlations from an oxymethylene H_2-9′ (δ_H 3.62, 3.49) to C-2′, C-3′, and C-4′ (δ_C 196.2), and from an amino proton NH-1′ (δ_H 5.79) to C-15, C-2′, and C-4′a (δ_C 117.4). The relative configurations of C-5, C-8, C-9, C-10, and C-13 were assigned to be identical to those of **2** based on the NOESY cross-peaks of H-5/H-9, H_3-17/H_3-20, H_3-20/H_a-11, H_a-11/H_3-16. Additionally, the *S**-configuration at C-14 in **3** was tentatively assigned based on consideration of a common biosynthetic origin of **1** and **2**. Note that the inversion of *S*/*R* designation at C-14 in **3** compared to those of **1** and **2** arises from the Cahn-Ingold-Prelog priority rules. The relative configuration of the bridgeheads C-2′ and C-3′ remains unresolved. Compound **3** was named zigralone C.

Compound **4**

Compound **4** was isolated as a colorless oil. Its molecular formula was determined as $C_{31}H_{43}NO_4$ based on a protonated molecular ion at *m/z* 494.3265 (calcd. for $C_{31}H_{44}NO_4^+$, 494.3265) in HRMS data. Analysis of the ^{1}H and ^{13}C NMR data of **4**, together with the characteristic UV absorptions (λ_{max} 230, 320, 332 nm), indicated the presence of a 7-methoxy-4-quinolone moiety. In addition to signals for a 7-methoxy-4-quinolone substructure, the ^{1}H NMR spectrum of **4** exhibited resonances for three olefinic protons (δ_H 5.14, 5.10, 5.18), a hydroxymethyl group (δ_H 4.67, 4.17), four allyl methyls (δ_H 1.64, 1.60, 1.581, 1.576), and a deshielded methyl (δ_H 1.46), as well as signals attributable to an AMX spin system of an oxymethine (δ_H 3.17) and an allyl methylene (δ_H 3.46, 2.95) (Table S10). Analysis of the ^{13}C NMR spectrum, with the aid of HSQC spectrum, revealed the presence of eight methylene carbons. The HMBC correlations from H_2-9 (δ_H 4.67) to C-2, C-3, and C-4 of a quinolone core indicated substitution at C-3 by a hydroxymethyl group. The HMBC correlations from H_3-17′ (δ_H 1.46) to C-2′ (δ_C 62.5), C-3′ (δ_C 61.9), and C-4′ (δ_C 39.2), together with 2D NMR correlations observed in the aliphatic region (Table S10) revealed the presence of a modified geranylgeranyl moiety with oxygenated functionalities at C-2′ and C-3′. Two trisubstituted allyl groups at C-6′/C-7′ and C-10′/C-11′ in the modified geranylgeranyl moiety were assigned as *E*-configurations based on the long-range COSY correlations and NOESY correlations of H-6′/H_2-8′, H_2-5′/H_3-18′, and H-10′/H_2-12′. The HMBC correlations from H_2-1′ (δ_H 3.46, 2.95) to C-2 (148.1) confirmed attachment of the modified geranylgeranyl moiety at C-2 of the quinolone core. Based on the number of oxygen atoms from the molecular formula, the oxygenated functionality at C-2′/C-3′ was determined to be an epoxy group. The NOESY correlations of H_3-17′/H-1′ and H-2′/H_2-4′ indicated a *trans*-configuration of the epoxide.

To determine the absolute configuration of **4**, a truncated model (2′*R*,3′*R*)-**4a** (Figure S8) was employed for ECD calculations to reduce the computational cost associated with the conformational flexibility of the linear prenyl chain, which is not expected to significantly influence the Cotton effects in the region of 200–400 nm. The calculated ECD spectrum of (2′*R*,3′*R*)-**4a** showed good agreement with the experimental ECD spectrum of **4**, thereby establishing its absolute configuration. Compound **4** was named zigralone D.

Compound **5**

Compound **5** was isolated as a yellow gum. Its molecular formula was determined as $C_{30}H_{41}NO_4$ based on a protonated molecular ion at *m/z* 480.3108 (calcd. for $C_{30}H_{42}NO_4^+$, 480.3108) in HRMS data. The ^{1}H and ^{13}C NMR spectra of **5** showed signals attributable to a 2,3-disubstituted 7-methoxy-4-quinolone moiety

and a farnesyl side chain, closely resembling to those of **4** (Table S11). Further analysis of the 1D NMR data revealed the absence of a hydroxymethyl group at C-3 observed in **4**. The C-2′ oxymethine proton signal (δ_H 3.98) showed a COSY correlation with a broad doublet signal corresponding to a hydroxy proton (δ_H 4.36), suggesting the presence of a hydroxy group at C-3′. In addition, the deshielded ^{13}C carbon chemical shift of C-3 (δ_C 137.27, 137.25), compared with that of **4**, suggested substitution by an oxygenated functionality. Considering the degrees of unsaturation and the number of oxygen atoms from the molecular formula, together with the presence of an additional oxygenated sp^3 quaternary carbon (δ_C 78.9, C-3′), suggested an ether linkage between C-3 and C-3′. The relative configurations of **5** was assigned as (2′*S**,3′*R**) based on the NOESY correlations (Table S11). In the pseudo-equatorial conformation of OH-2, the NOESY correlations of H$_a$-1′/H-2′, H-2′/H$_2$-4′, and H$_b$-1′/H$_3$-17 were observed, whereas in the pseudo-axial conformation of OH-2, the NOESY correlations of H-2′/H$_3$-17′ and H-1′/H-4′ were detected. The presence of these two conformations were inferred from the duplicated ^{13}C NMR signals for C-1′ and C-2′, as well as for part of the quinolone core (Table S11), likely arising from restricted ring flipping of the dihydropyran ring. Furthermore, two trisubstituted olefins were assigned to be *E*-configurations based on the NOESY correlations of H-6′/H$_2$-8′, H$_2$-5′/H$_3$-18′, and H-10′/H$_2$-12′. To determine the absolute configuration, a truncated model (2′*S*,3′*R*)-**5a** (Figure S8) was employed for ECD calculations. The calculated ECD spectrum showed good agreement with the experimental ECD spectrum of **5**, establishing its absolute configuration as (2′*S*,3′*R*). Compound **5** was named zigralone E.

<u>Compound **6**</u>

Compound **6** was isolated as a pale-brown solid. Its molecular formula was determined as $C_{31}H_{43}NO_4$ based on a protonated molecular ion at *m/z* 494.3265 (calcd. for $C_{31}H_{44}NO_4^+$, 494.3265) in HRMS data. Comparison of the ^1H and ^{13}C NMR spectra of **6** with those of **2** revealed the absence of the C-15 ketone and the presence of an allyl methylene (δ_C 27.2; δ_H 1.89, 1.54) that exhibited ^1H–^1H spin coupling with the oxymethine H-14 (δ_H 3.38) (Table S13). These 1D NMR data suggest that **6** is a 15-deoxo analog of **2**. This assignment was further supported by the HMBC correlations from H-15 (δ_H 1.89, 1.54) to C-2′ (δ_C 143.8) and C-3′ (δ_C 116.0), and from NH-1′ (δ_H 8.43) to C-15 (δ_C 27.2). The relative configurations (5*S**,8*R**,9*R**,10*S**,13*R**) in **6** was established based on the NOESY cross-peaks of H-5/H-9, H$_3$-19/H$_a$-2, H$_a$-2/H$_3$-17, H$_3$-17/H$_3$-20, H$_3$-20/H$_a$-11, and H$_a$-11/H$_3$-16. Additionally, the *S**-configuration at C-14 in **6** was tentatively assigned based on consideration of a common biosynthetic origin of **1** and **2**. Compound **6** was named zigralone F.

<u>Compound 7</u>

Compound **7** was isolated as a colorless gum. Its molecular formula was determined as $C_{31}H_{43}NO_2$ based on a protonated molecular ion at *m/z* 462.3367 (calcd. for $C_{31}H_{44}NO_2^+$, 462.3367) in HRMS data. Comparison of the ^1H and ^{13}C NMR spectra of **7** with those of **4** revealed that the absence of the C-3 hydroxymethyl group and the C-2′/C3′ epoxide moiety. Instead, signals for an additional olefin (δ_C 143.9; δ_C 116.7, δ_H 5.39) and an aryl methyl (δ_C 10.4, δ_H 2.16) were observed, indicating substitution by an unmodified geranylgeranyl group at C-2 and a methyl at C-3 in a 7-methoxy-4-quinolone moiety (Table S14). This assignment was further supported by the COSY correlations between H-1′ (δ_H 3.49) and H-2′ (δ_H 5.39), as well as the HMBC correlations from H-1′ to C-2 (δ_C 145.1), and from H$_3$-9 (δ_H 2.16) to C-2, C-3 (δ_C 116.0), and C-4 (δ_C 177.8). The configuration of trisubstituted olefins at C-2′/C3′, C-6′/C7′, and C-10′/C11′ was determined to be all *E*-configuration based on the long-range COSY correlations and the NOESY correlations of H$_2$-1′/H$_3$-17′, H$_2$-2′/H$_2$-4′, H$_2$-5′/H$_3$-18′, H-6′/H$_2$-8′, and H-10′/H$_2$-12′. Compound **7** was named zigralone G.

Compound **9**

Compound **9** was isolated as a dark yellow oil. Its molecular formula was determined as $C_{33}H_{45}NO_4$ based on a deprotonated molecular ion at *m/z* 518.3271 (calcd. for $C_{33}H_{44}NO_4^-$, 518.3276) in HRMS data. The ^{1}H NMR spectrum of **9** showed resonances for a secondary amino proton (δ_H 8.18), three aromatic protons consistent with a 1,2,3-trisubstituted phenyl group (δ_H 7.09, 6.98, 6.58), four olefinic protons (δ_H 5.35, 5.15, 5.11, 5.09), an AX spin system corresponding to an ethylidene group (δ_H 4.87, 1.581), an aryl methoxy group (δ_H 3.92), five allyl methyls (δ_H 1.76, 1.68, 1.63, 1.60, 1.584), and multiple aliphatic methylenes (Table S15). Analysis of the ^{13}C NMR spectrum revealed the presence of an α,β-unsaturated ketone (δ_C 192.9) and an α,β-unsaturated carboxylic acid (δ_C 160.1, 160.0) attributable to an α-keto acid, as well as nine unprotonated aryl and olefinic carbons. The split signals of the carboxylic acid are likely due to the presence of *cis*- and *trans*-rotamers of the α-keto acid. The HMBC correlations from an amino proton, aromatic protons, and a methoxy protons to unprotonated aryl carbons (Table S15), together with characteristic UV absorption maxima at 205, 224, 269 nm, revealed the presence of 2,3-disubstituted 7-methoxyindole moiety. The HMBC correlations from H-8 (δ_H 4.87) to C-9 (δ_C 192.9), C-2 (δ_C 137.3), C-3 (δ_C 104.9), and C-3a (δ_C 128.4) indicated substitution at C-3 of the indole core by the ethylidene moiety that is linked to an α-keto acid. Analysis of 2D NMR data in the aliphatic region (Table S15) revealed the presence of a geranylgeranyl moiety. The configurations of trisubstituted olefins at C-2′/C3′, C-6′/C7′, and C-10′/C11′ were determined to be *E*-configurations based on the NOESY correlations of H_2-1′/H_3-17′, H_2-2′/H_2-4′, H_2-5′/H_3-18′, H-6′/H_2-8′, H_2-9′/H_3-19′, and H-10′/H_2-12′. The deshielded methylene protons H_2-1′ (δ_H 3.63, 3.57) showed the HMBC correlations to C-2 and C-3, indicating the location of a geranylgeranyl moiety at C-2. To determine the stereochemistry at C-8, a truncated model (*R*)-**9a** (Figure S8) was employed for ECD calculations. The calculated ECD spectrum of (*R*)-**9a** showed almost mirror-image Cotton effects compared with the experimental ECD spectrum of **9**, establishing the *S*-configuration at C-8. Compound **9** was named as prezigralone G.

Compound **10**

Compound **10** was isolated as a pale-brown solid. Its molecular formula was determined as $C_{30}H_{41}NO_2$ based on a protonated molecular ion at *m/z* 448.3210 (calcd. for $C_{30}H_{42}NO_2^+$, 448.3210) in HRMS data. The ^{1}H and ^{13}C NMR spectra of **10** were akin to those of **9** except for the absence of signals for an ethylidene moiety and an α-keto acid (Table S16). Instead, signals for an aldehyde (δ_C 184.7; δ_H 10.22) were observed. The HMBC correlations from an aldehyde proton to C-2 (δ_C 48.6), C-3 (δ_C 114.5), and C-3a (δ_C 127.9) indicated that the C-3 position of an indole moiety is substituted by an aldehyde. Consequently, **10** was identified as 2-geranylgeranyl-7-methoxy-1*H*-indole-3-carboxaldehyde.

Compound **12**

Compound **12** was isolated as a colorless gum. Its molecular formula was determined as $C_{31}H_{41}NO_3$ based on a protonated molecular ion at *m/z* 476.3160 (calcd. for $C_{31}H_{42}NO_3^+$, 476.3159) in HRMS data. The ^{1}H NMR spectrum exhibited resonances for a singlet aromatic proton (δ_H 8.54), four aromatic protons (δ_H 7.85, 7.75, 7.29, 7.16) consistent with a 1,2-disubstituted phenyl group, four olefinic protons (δ_H 5.30, 5.24, 5.22, 5.17), an aryl oxymethine (δ_H 4.94), five allyl methyls (δ_H 1.68, 1.62, 1.61, 1.56, 1.36), and multiple aliphatic methylenes (Table S17). Analysis of the ^{13}C NMR spectrum revealed the presence of an α,β-unsaturated carboxylic acid (δ_C 163.9) and eight unprotonated aryl and olefinic carbons. Analysis of 2D NMR data in the aliphatic region (Table S17) revealed the presence of a geranylgeranyl moiety with *E*-configurations of three trisubstituted olefins at C-3′/C-4′, C-6′/C-7′, and C-10′/C-11′. The COSY correlation

between H_2-1′ (δ_H 2.34) and the aryl oxymethine H-10 (δ_H 4.94) suggested connection of the geranylgeranyl group to the aryl oxymethine. The aromatic moiety of **12** was assigned as a 4-alkyl quinaldic acid based on the HMBC correlations from H-3 (δ_H 8.54) to C-2 (δ_C 146.8), C-4a (δ_C 127.5), and C-9 (δ_C 163.9), from H-5 (δ_H 7.75) to C-4 (δ_C 153.6), C-4a, and C-8a (δ_C 146.3), and from H-7 (δ_H 7.29) to C-8a. The HMBC correlations from H-10 to C-3, C-4, and C-4a established the linkage of the oxymethine carbon C-10 to C-4 of the quinaldic acid moiety. The stereochemistry of C-10 remains unresolved. Consequently, **12** was identified as 4-(geranylgeranyl(hydroxy)methyl)quinaldic acid.

4. References

1. Bierman, M; Logan, R; O'Brien, K; Seno, E. T.; Nagaraja Rao, R.; Schoner, B. E. Plasmid Cloning Vectors for the Conjugal Transfer of DNA from *Escherichia coli* to *Streptomyces* spp. *Gene* **1992**, *116*, 43–49.
2 MacNeil, D. J.; Gewain, K. M.; Ruby, C. L.; Dezeny, G.; Gibbons, P. H.; MacNeil, T. Analysis of *Streptomyces avermitilis* Genes Required for Avermectin Biosynthesis Utilizing a Novel Integration Vector. *Gene* **1992**, *111*, 61–68.
3. Chater, K. F.; Wilde, L. C. Restriction of a Bacteriophage of *Streptomyces albus* G Involving Endonuclease *Sal*I. *J. Bacteriol.* **1976**, *128*, 644–650.
4. Hopwood, D. A.; Kieser, T.; Wright, H. M.; Bibb, M. J. Plasmids, Recombination and Chromosome Mapping in *Streptomyces lividans* 66. *J. Gen. Microbiol.* **1983**, *129*, 2257–2269.
5. Gomez-Escribano, J. P.; Bibb, M. J. Engineering *Streptomyces coelicolor* for Heterologous Expression of Secondary Metabolite Gene Clusters. *Microb. Biotechnol.* **2011**, 4, 207–215.
6. Edgar, R. C. Muscle5: High-Accuracy Alignment Ensembles Enable Unbiased Assessments of Sequence Homology and Phylogeny. *Nat. Commun.* **2022**, *13*, 6968.
7. Eddy, S. R. Accelerated Profile HMM Searches. *PLoS Comput. Biol.* **2011**, *7*, e1002195.
8. Fu, L.; Niu, B.; Zhu, Z.; Wu, S.; Li, W. CD-HIT: Accelerated for Clustering the Next-Generation Sequencing Data. *Bioinformatics* **2012**, *28*, 3150–3152.
9. a) Back, D.; O'Donnell, T. J.; Axt, K. K.; Gurr, J. R.; Vanegas, J. M.; Williams, P. G.; Philmus, B. Identification, Heterologous Expression, and Characterization of the Tolypodiol Biosynthetic Gene Cluster through an Integrated Approach. *ACS Chem. Biol.* **2023**, *18*, 1797–1807. b) Tang, J.; Matsuda, Y. Disocovery of Fungal Onoceroid Triterpenoids through Domainless Enzyme-Targeted Global Genome Mining. *Nat. Commun.* **2024**, *15*, 4312.
10. Copp, J. N.; Akiva, E.; Babbitt, P. C.; Tokuriki, N. Revealing Unexplored Sequence-Function Space Using Sequence Similarity Networks. *Biochemistry* **2018**, *57*, 4651–4662.
11. Shannon, P.; Markiel, A.; Ozier, O.; Baliga, N. S.; Wang, J. T.; Ramage, D.; Amin, N.; Schwikowski, B.; Ideker, T. Cytoscape: A Software Environment for Integrated Models of Biomolecular Interaction Networks. *Genome Res.* **2003**, *13*, 2498–2504.
12. Katoh, K.; Standley, D. M. MAFFT Multiple Sequence Alignment Software Version 7: Improvements in Performance and Usability. *Mol. Biol. Evol.* **2013**, *30*, 772–780.
13. Capella-Gutiérrez, S.; Silla-Martínez, J. M.; Gabaldón, T. trimAI: A Tool for Automated Trimming in Large-Scale Phylogenetic Analyses. *Bioinformatics* **2009**, *25*, 1972–1973.
14. Wong, T. K. F.; Ly-Trong, N.; Ren, H.; Baños, H.; Roger, A. W.; Susko, E.; Bielow, C.; De Maio, N.; Goldman, N.; Hahn, M. W.; Huttley, G.; Lanfear, R.; Minh, B. Q. IQ-TREE 3: Phylogenomic Inference Software Using Complex Evolutionary Models, Ver. 1. *EcoEvoRxiv*, April 7, 2025. DOI: 10.32942/X2P62N
15. Schwarz, G. Estimating the Dimension of a Model. *Ann. Stat.* **1978**, *6*, 461–464.
16. Hoang, D. T.; Chernomor, O.; von Haeseler, A.; Minh, B. Q.; Vinh, L. S. UFBoot2: Improving the Ultrafast Bootstrap Approximation. *Mol. Biol. Evol.* **2017**, *35*, 518–522.
17. Letunic, I.; Bork, P. Interactive Tree of Life (iTOL) v6: Recent Updates to the Phylogenetic Tree Display and Annotation Tool. *Nucleic Acids Res.* **2024**, *52*, W78–W82.
18. Gilchrist, C. L. M.; Chooi, Y.-H. clinker & clustermap.js: Automatic Generation of Gene Cluster Comparison Figures. *Bioinformatics* **2021**, *37*, 2473–2475.
19. Abramson, J.; Adler, J.; Dunger, J.; Evans, R.; Green, T.; Pritzel, A.; Ronneberger, O.; Willmore, L.; Ballard, A. J.; Bambrick, J.; Bodenstein, S. W.; Evans, D. A.; Hung, C.-C.; O'Neill, M.; Reiman, D.; Tunyasuvunakool, K.; Wu, Z.; Žemgulytė, A.; Arvaniti, E.; Beattie, C.; Bertolli, O.; Bridgland, A.; Cherepanov, A.; Congreve, M.; Cown-Rivers, A. I.; Cowie, A.; Figurnov, M.; Fuchs, F. B.; Gladman, H.; Jain, R.; Khan, Y. A.; Low, C. M. R.; Perlin, K.; Potapenko, A.; Savy, P.; Singh, S.; Stecula, A.; Thillaisundaram, A.; Tong, C.; Yakneen, S.; Zhong, E. D.; Zielinski, M.; Žídek, A.; Bapst, V.; Kohli, P.;

Jaderberg, M.; Hassabis, D.; Jumper, J. M. Accurate Structure Prediction of Biomolecular Interactions with AlphaFold3. *Nature* **2024**, *630*, 493–500.

20. Waterhouse, A. M.; Procter, J. B.; Martin, D. M. A.; Clamp, M.; Barton, G. J. Jalview Version 2—A Multiple Sequence Alignment Editor and Analysis Workbench. *Bioinformatics* **2009**, *25*, 1189–1191.
21. Bai, C.; Zhang, Y.; Zhao, X.; Hu, Y.; Xiang, S.; Miao, J.; Lou, C.; Zhang, L. Exploiting a Precise Design of Universal Synthetic Modular Regulatory Elements to Unlock the Microbial Natural Products in *Streptomyces*. *Proc. Natl. Acad. Sci. U. S. A.* **2015**, *112*, 12181–12186.
22. Kieser, T.; Bibb, M. J.; Buttner, M. J.; Chater, K. F.; Hopwood, D. A. *Practical Streptomyces Genetics*; John Innes Foundation, 2000.
23. *CrysAlisPro*, Ver. 1.171.42.43a; Rigaku Oxford Diffraction, 2022.
24. Sheldrick, G. M. SHELXT – Integrated Spece-Group and Crystal-Structure Determination. *Acta Crystallogr., Sect. A:Found. Adv.* **2015**, *71*, 3–8.
25. Sheldrick, G. M. Crystal Structure Refinement with SHELXL. *Acta Crystallogr., Sect. C:Struct. Chem.* **2015**, *71*, 3–8.
26. *Spartan'20*, Ver. 1.1.4; Wavefunction, Inc., 2022.
27. Halgren, T. A. Merck Molecular Force Field. I. Basis, Form, Scope, Parameterization, and Performance of MMFF94. *J. Comput. Chem.* **1996**, *17*, 490–519.
28. Frisch, M. J.; Trucks, G. W.; Schlegel, H. B.; Scuseria, G. E.; Robb, M. A.; Cheeseman, J. R.; Scalmani, G.; Barone, V.; Petersson, G. A.; Nakatsuji, H.; Li, X.; Caricato, M.; Marenich, A. V.; Bloino, J.; Janesko, B. G.; Gomperts, R.; Mennucci, B.; Hratchian, H. P.; Ortiz, J. V.; Izmaylov, A. F.; Sonnenberg, J. L.; Williams-Young, D.; Ding, F.; Lipparini, F.; Egidi, F.; Goings, J.; Peng, B.; Petrone, A.; Henderson, T.; Ranasinghe, D.; Zakrzewski, V. G.; Gao, J.; Rega, N.; Zheng, G.; Liang, W.; Hada, M.; Ehara, M.; Toyota, K.; Fukuda, R.; Hasegawa, J.; Ishida, M.; Nakajima, T.; Honda, Y.; Kitao, O.; Nakai, H.; Vreven, T.; Throssell, K.; Montgomery, J. A., Jr.; Peralta, J. E.; Ogliaro, F.; Bearpark, M. J.; Heyd, J. J.; Brothers, E. N.; Kudin, K. N.; Staroverov, V. N.; Keith, T. A.; Kobayashi, R.; Normand, J.; Raghavachari, K.; Rendell, A. P.; Burant, J. C.; Iyengar, S. S.; Tomasi, J.; Cossi, M.; Millam, J. M.; Klene, M.; Adamo, C.; Cammi, R.; Ochterski, J. W.; Martin, R. L.; Morokuma, K.; Farkas, O.; Foresman, J. B.; Fox, D. J. *Gaussian 16*, Revision C.02; Gaussian, Inc.; Wallingford CT, 2016.
29. Bruhn, T.; Schaumlöffel, A.; Hemberger, Y.; Bringmann, G. SpecDis: Quantifying the Comparison of Calculated and Experimental Electronic Circular Dichroism Spectra. *Chirality* **2013**, *25*, 243–249.
30. Zhao, Y.; Truhlar, D. G. The M06 Suite of Density Functionals for Main Group Thermochemistry, Thermochemical Kinetics, Noncovalent interactions, Excited States, and Transition Elements: Two New Functionals and Systematic Testing of Four M06-Class Functionals and 12 Other Functionals. *Theor. Chem. Acc.* **2008**, *120*, 215–241.
31. Weigend, F.; Ahlrichs, R. Balanced Basis Sets of Split Valence, Triple Zeta Valence and Quadruple Zeta Valence Quality for H to Rn: Design and Assessment of Accuracy. *Phys. Chem. Chem. Phys.* **2005**, *7*, 3297–3305.
32. Marenich, A. V.; Cramer, C. J.; Truhlar, D. G. Universal Solvation Model Based on Solute Electron Density and on a Continuum Model of the Solvent Defined by the Bulk Dielectric Constant and Atomic Surface Tensions. *J. Phys. Chem. B* **2009**, *113*, 6378–6396.
33. Chai, J.-D.; Head-Gordon, M. Long-Range Corrected Hybrid Density Functionals with Damped Atom–Atom Dispersion Corrections. *Phys. Chem. Chem. Phys.* **2008**, *10*, 6615–6620.
34. Kendall, R. A.; Dunning, T. H.; Harrison, R. J. Electron Affinities of the First-Row Atoms Revisited. Systematic Basis Sets and Wave Functions. *J. Chem. Phys.* **1992**, *96*, 6796–6806.
35. Adamo, C.; Barone, V. Toward Reliable Density Functional Methods without Adjustable Parameters: The PBE0 Model. *J. Chem. Phys.* **1999**, *110*, 6158–6170.
36. Cossi, M.; Rega, N.; Scalmani, G.; Barone, V. Energies, Structures, Electronic Properties of Molecules in Solution with the C-PCM Solvation Model. *J. Comput. Chem.* **2003**, *24*, 669–681.

37. Grimme, S.; Ehrlich, S.; Goerigk, L. Effect of the Damping Function in Dispersion Corrected Density Functional Theory. *J. Comput. Chem.* **2011**, *32*, 1456–1465.

38. Fukui, K. The Path of Chemical Reactions. *Acc. Chem. Res.* **1981**, *14*, 363–368.

39. Grimme, S. Supramolecular Binding Thermodynamics by Dispersion-Corrected Density Functional Theory. *Chem. – Eur. J.* **2012**, *18*, 9955–9964.

40. Luchini, G.; Alegre-Requena, J.; Funes-Ardoiz, I.; Paton, R. S. GoodVibes: Automated Thermochemistry for Heterogeneous Computational Chemistry Data. *F1000Research* **2020**, *9*, 291.

41. Wiegand, I.; Hilpert, K.; Hancock, R. E. W. Agar and Broth Dilution Methods to Determine the Minimal Inhibitory Concentration (MIC) of Antimicrobial Substances. *Nat. Protoc.* **2008**, *3*, 163–175.

5. Single Crystal X-ray Diffraction Data

Crystallographic data for **2**

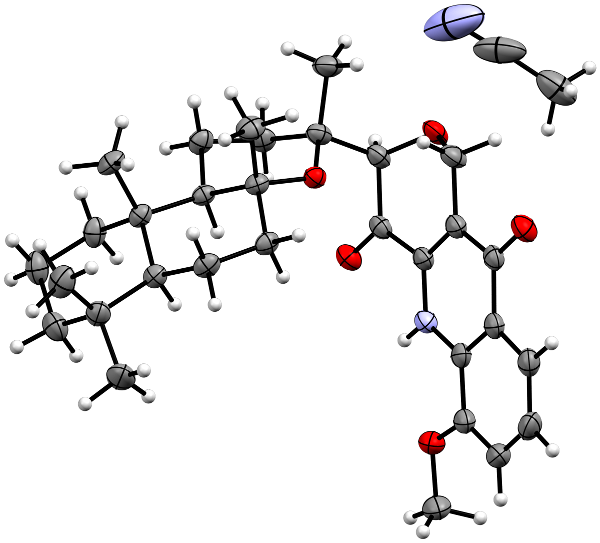

X-ray structure of **2** with thermal ellipsoids at 50% probability

CCDC deposit number	2542513
Empirical formula	$C_{31}H_{41}NO_5{\cdot}C_2H_3N$
Formula weight	548.70
Temperature [K]	173(2)
Crystal system	monoclinic
Space group	$P2_1$
a [Å]	12.8546(5)
b [Å]	7.1295(2)
c [Å]	15.9177(6)
α [°]	90
β [°]	91.080(3)
γ [°]	90
Volume [Å^3]	1458.54(8)
Z	2
ρ_{calc} [g/mm^3]	1.249
μ [mm^{-1}]	0.083
F(000)	592.0
Crystal size (mm^3)	0.4 × 0.11 × 0.09
Crystal color	colorless
Crystal shape	elongated prism
Radiation	Mo $K\alpha$ (λ = 0.71073)
2Θ range for data collection [°]	5.12 to 53.464
Index ranges	$-16 \leq h \leq 16$, $-8 \leq k \leq 9$, $-20 \leq l \leq 20$
Reflections collected	13122
Independent reflections	5991 [R_{int} = 0.0432, R_{sigma} = 0.0550]
Data/restraints/parameters	5991/1/372
Goodness-of-fit on F^2	1.046
Final R indexes [$I \geq 2\sigma(I)$]	R_1 = 0.0424, wR_2 = 0.1076
Final R indexes [all data]	R_1 = 0.0484, wR_2 = 0.1124
Largest diff. peak/hole [e Å^{-3}]	0.26/−0.20

6. Copies of NMR Spectroscopic Data

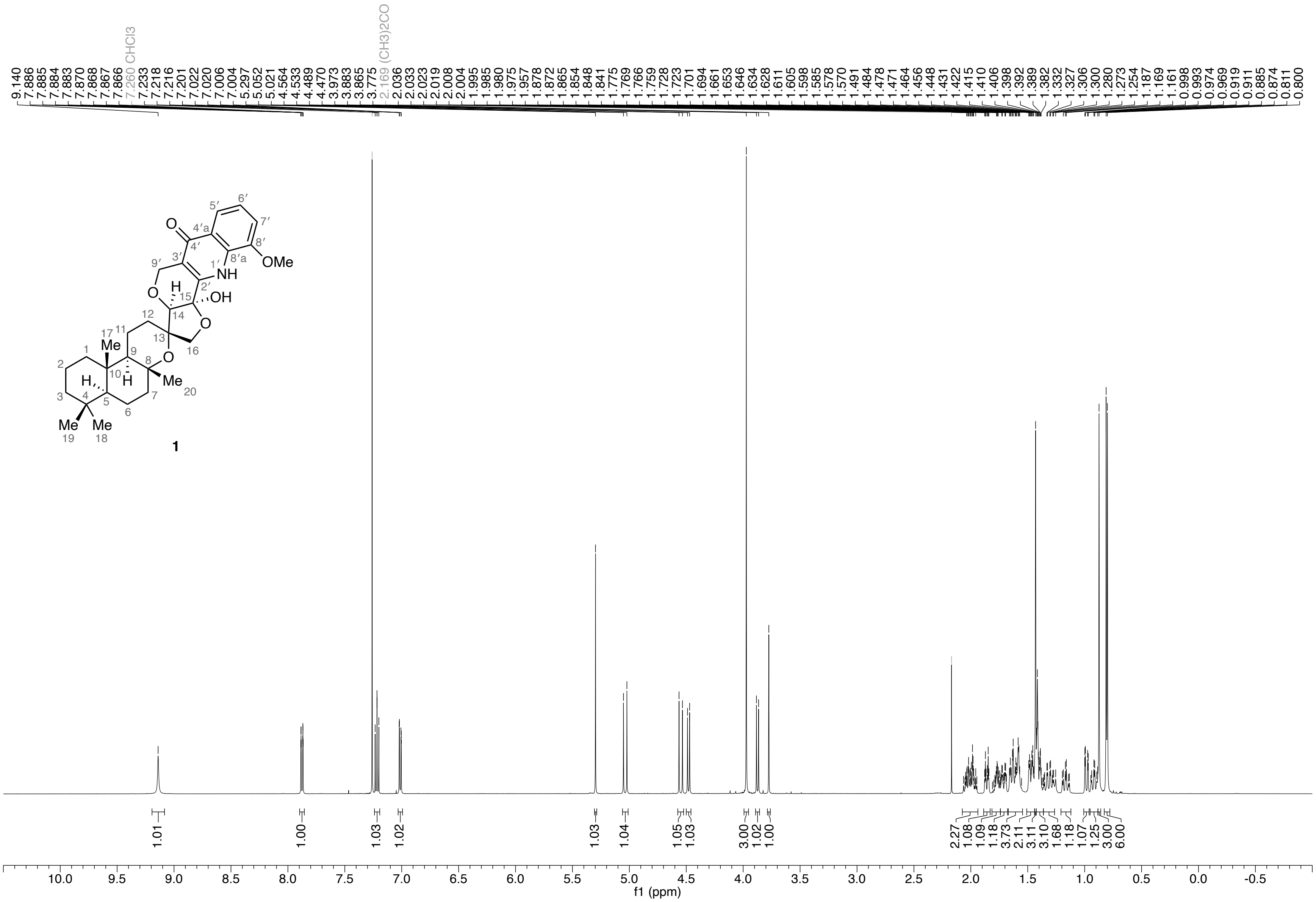

^{1}H NMR spectrum of zigralone A (**1**) (500.18 MHz, 298K, $CDCl_3$)

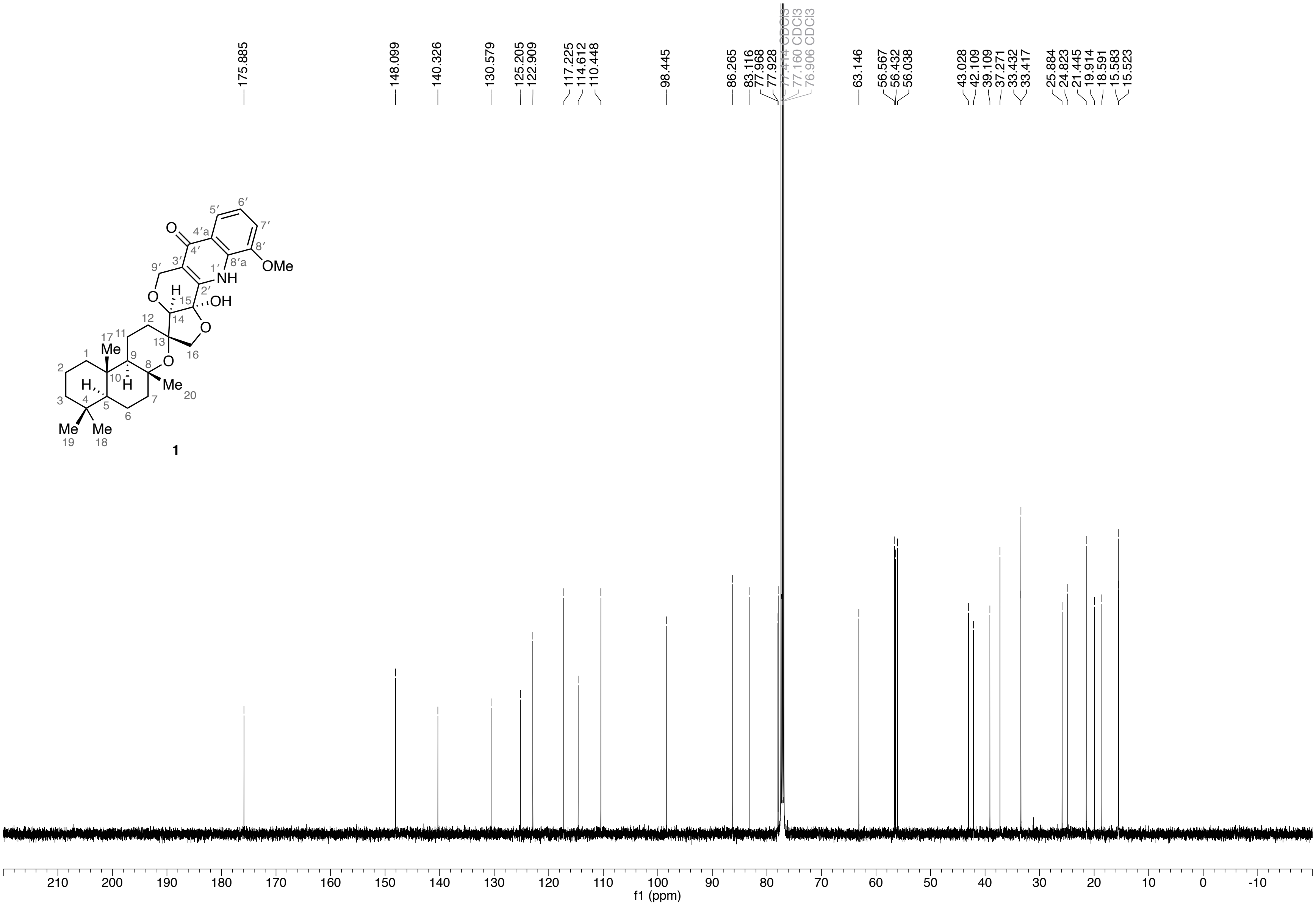

$^{13}C\{^1H\}$ NMR spectrum of zigralone A (**1**) (125.78 MHz, 298K, $CDCl_3$)

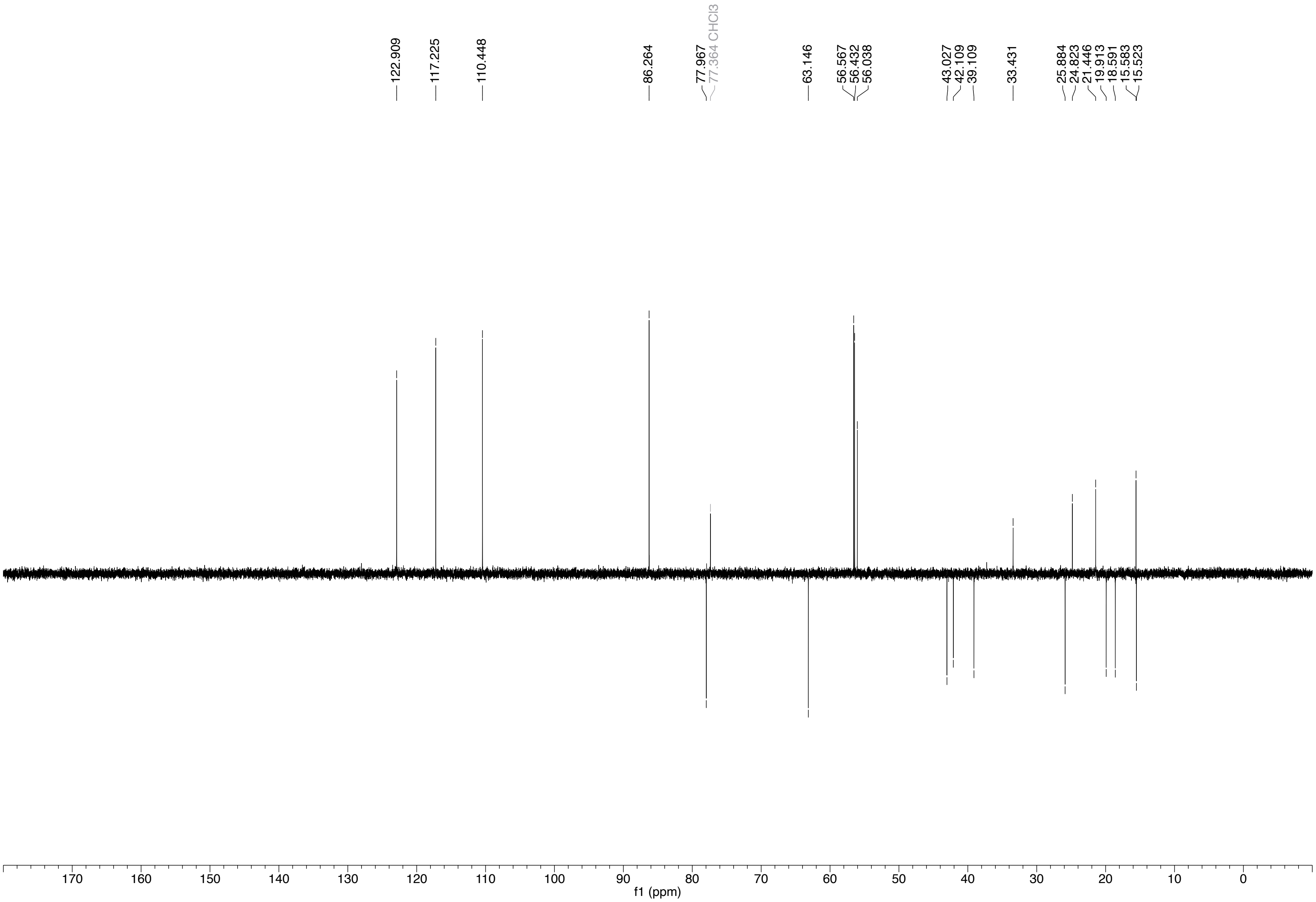

DEPT135 NMR spectrum of zigralone A (**1**) (125.78 MHz, 298K, $CDCl_3$)

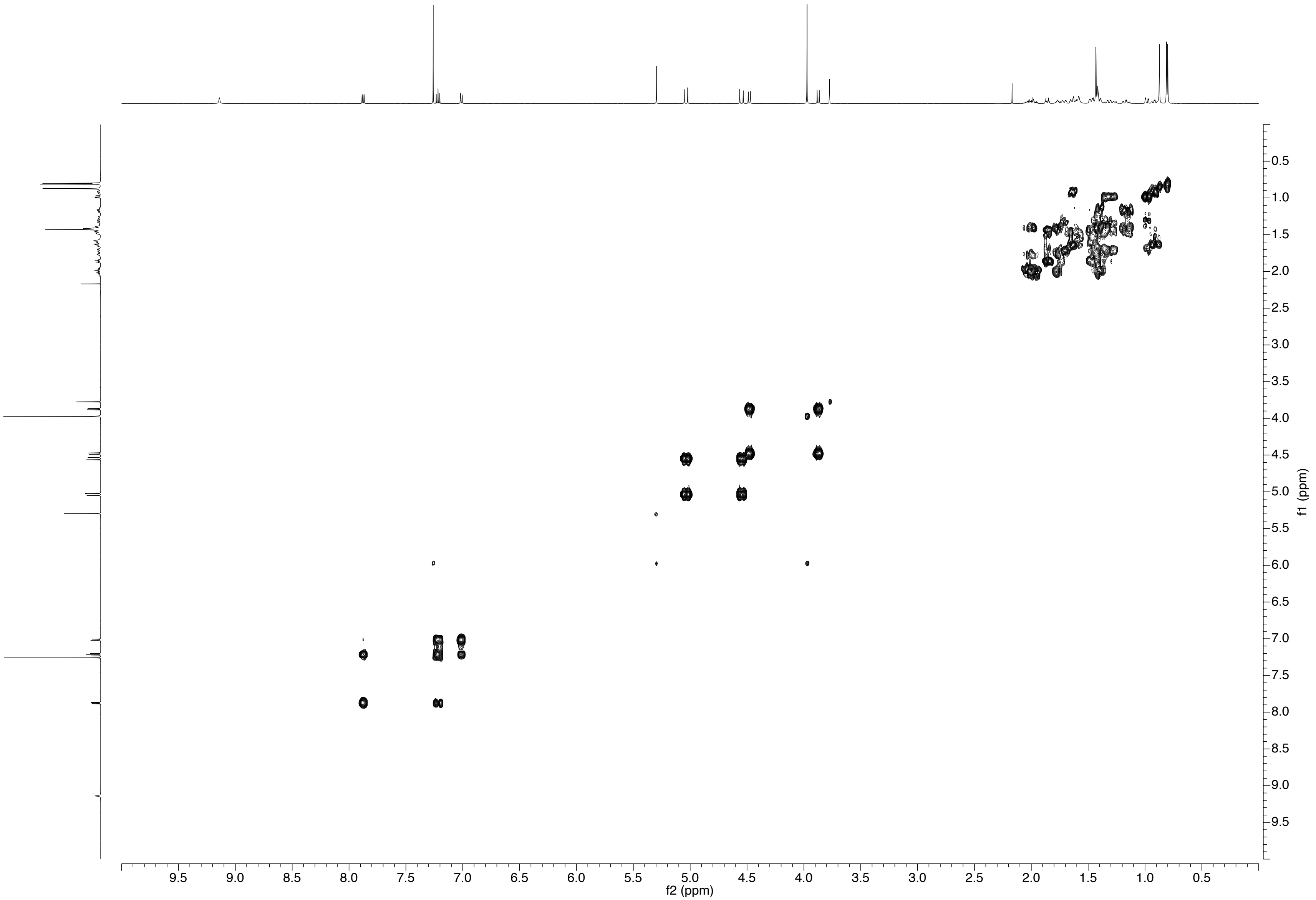

gCOSY spectrum of zigralone A (**1**) (298K, $CDCl_3$)

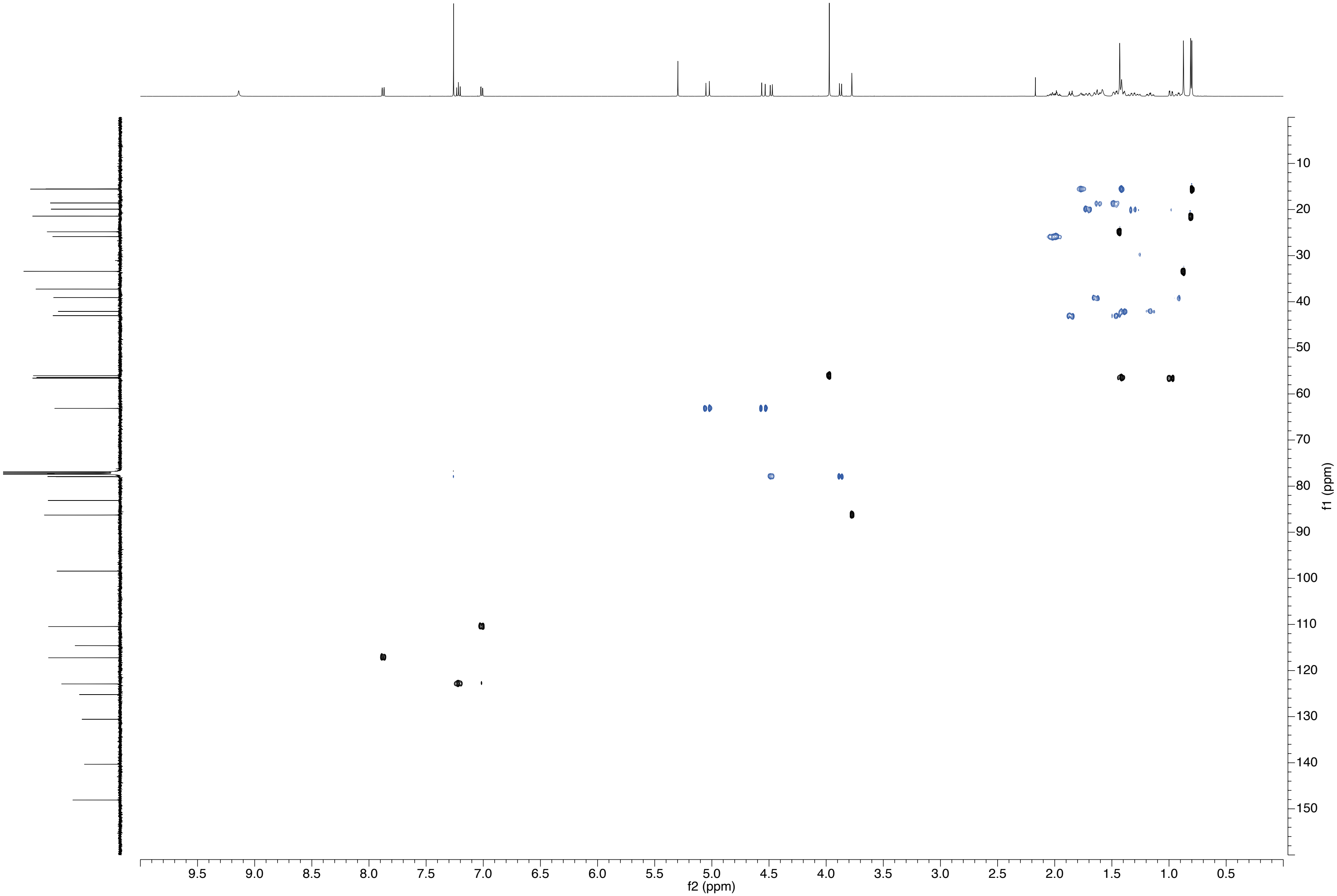

gHSQC spectrum of zigralone A (**1**) (298K, $CDCl_3$)

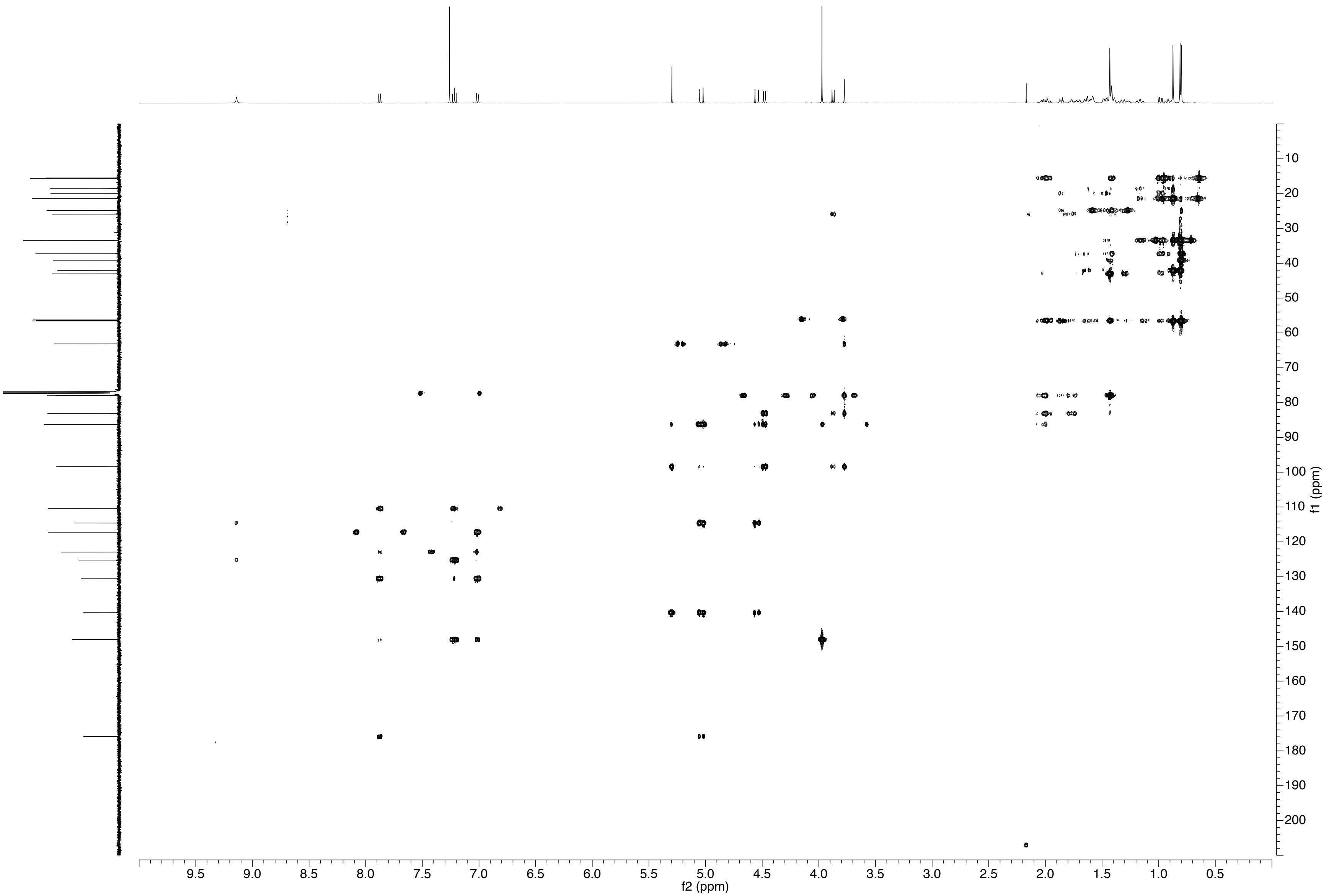

gHMBC spectrum of zigralone A (**1**) (298K, $CDCl_3$)

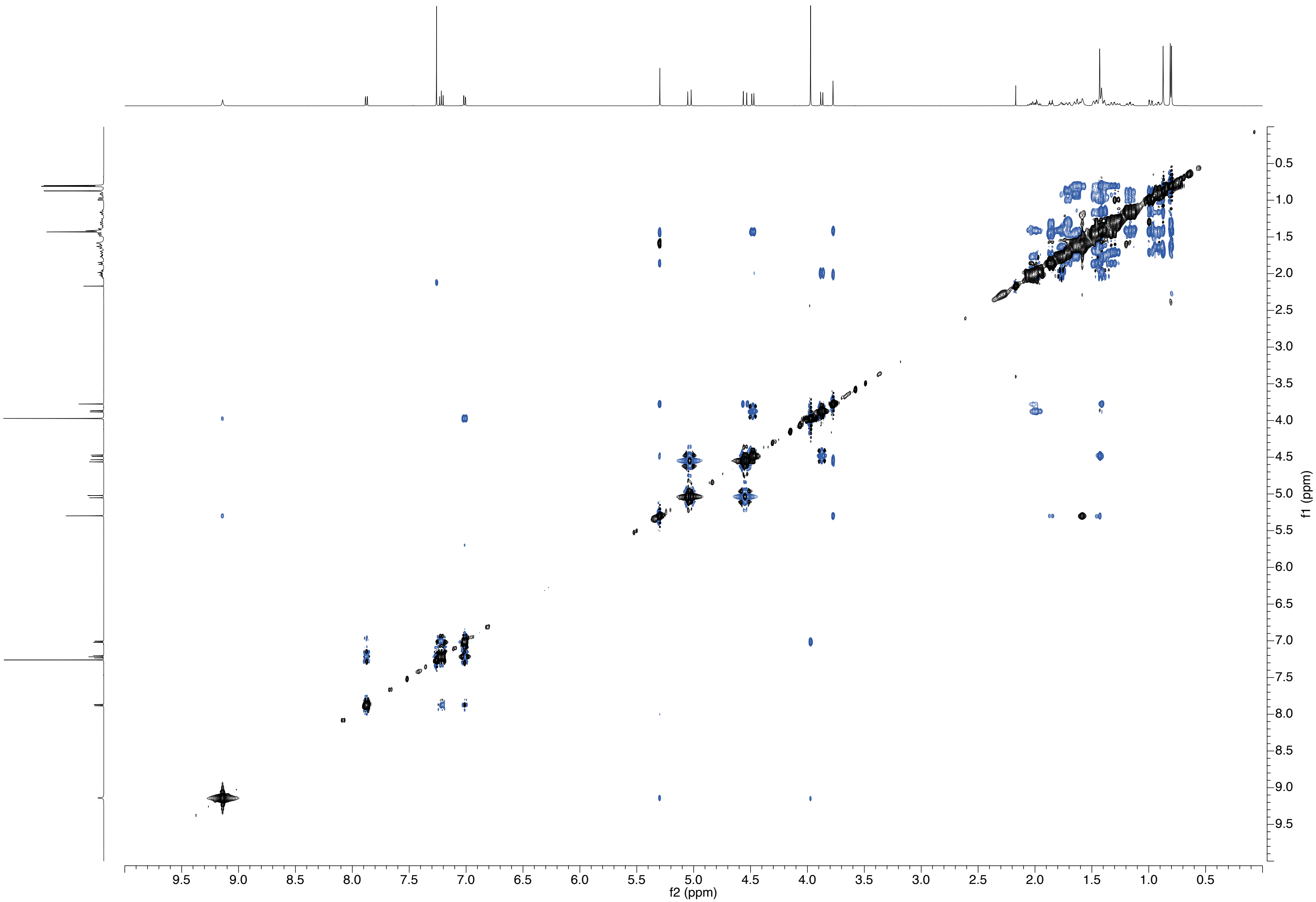

gNOESY spectrum of zigralone A (**1**) (298K, $CDCl_3$)

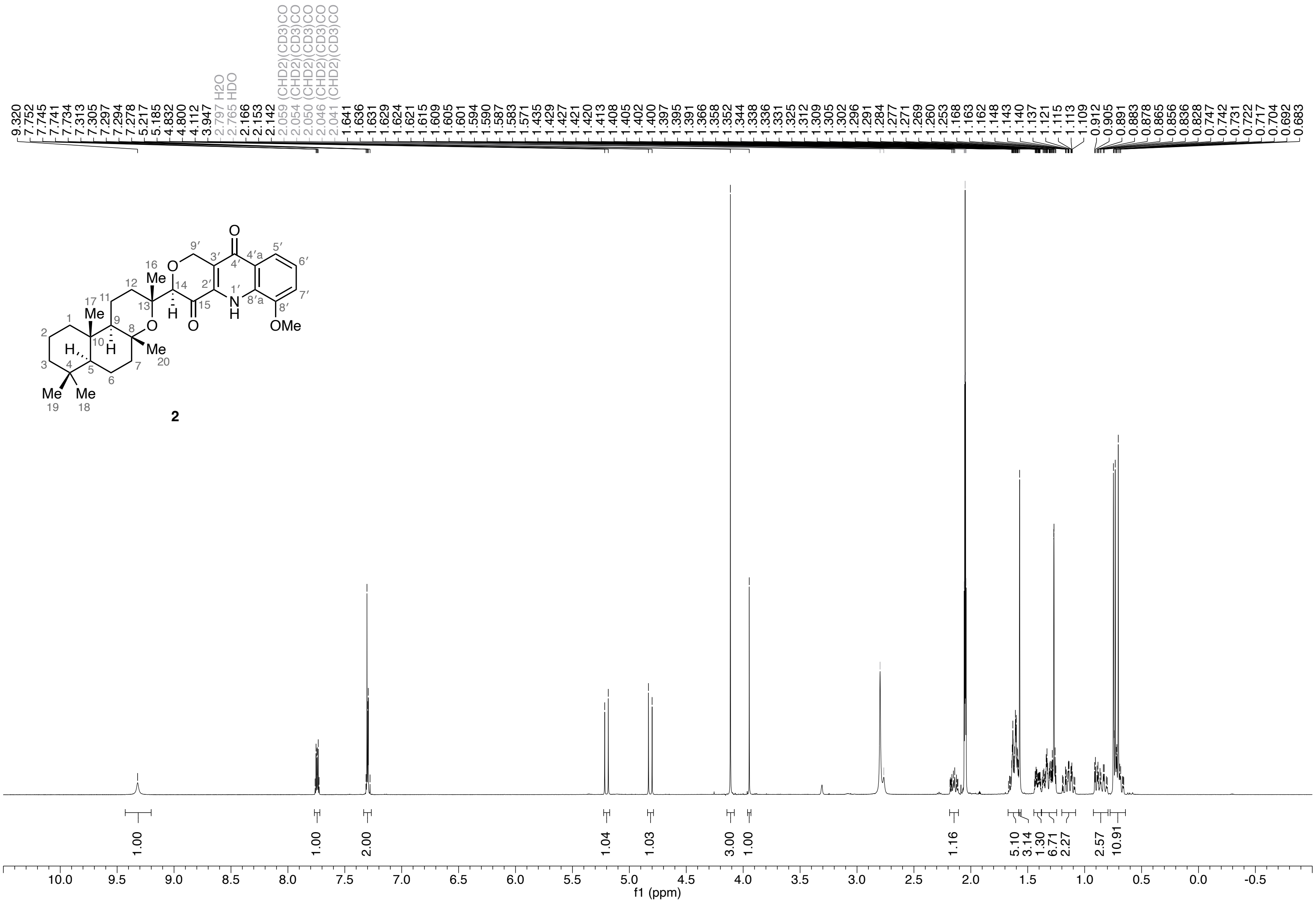

^{1}H NMR spectrum of zigralone B (**2**) (500.18 MHz, 298K, $(CD_3)_2CO$)

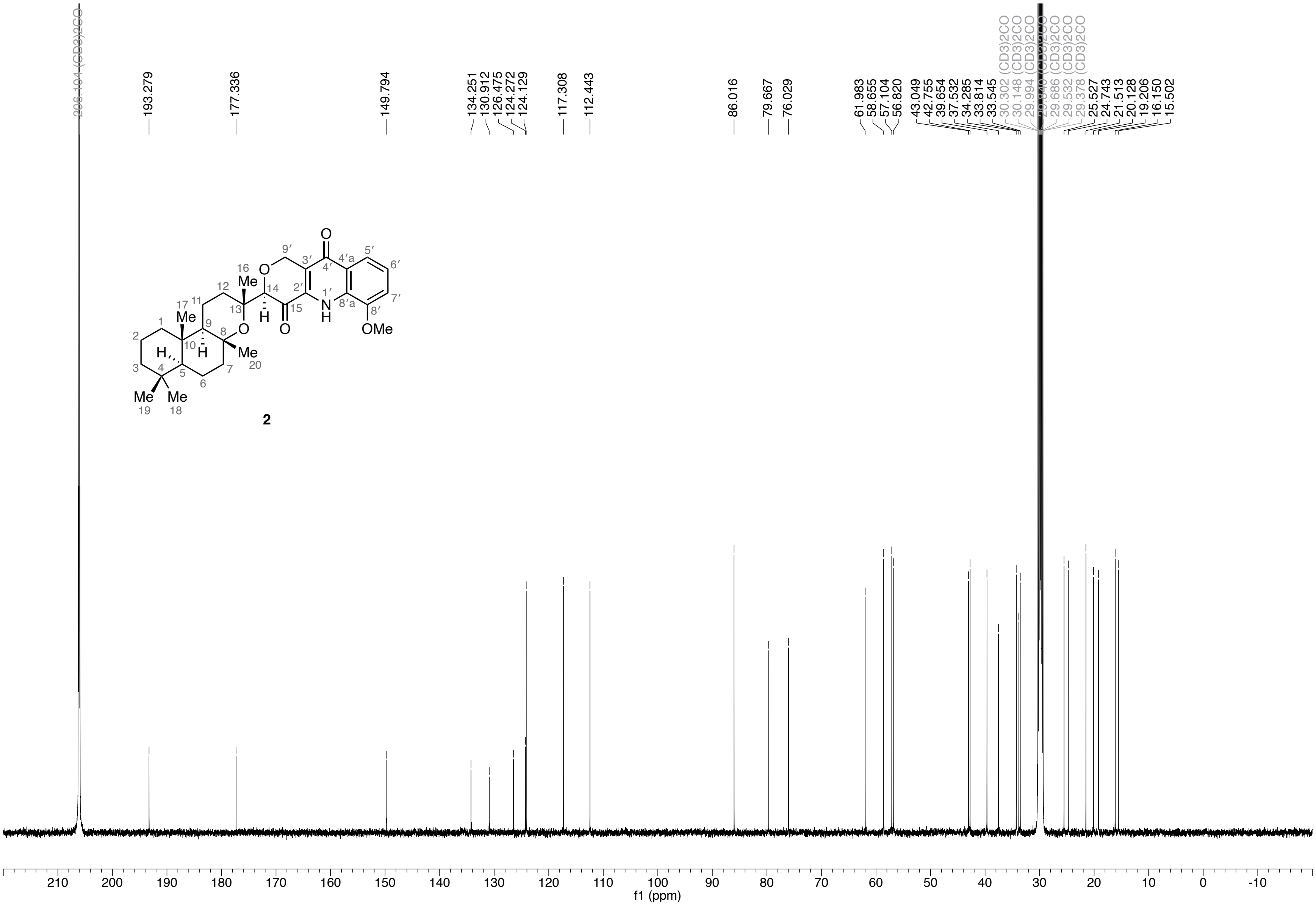

$^{13}C\{^{1}H\}$ NMR spectrum of zigralone B (**2**) (125.78 MHz, 298K, $(CD_3)_2CO$)

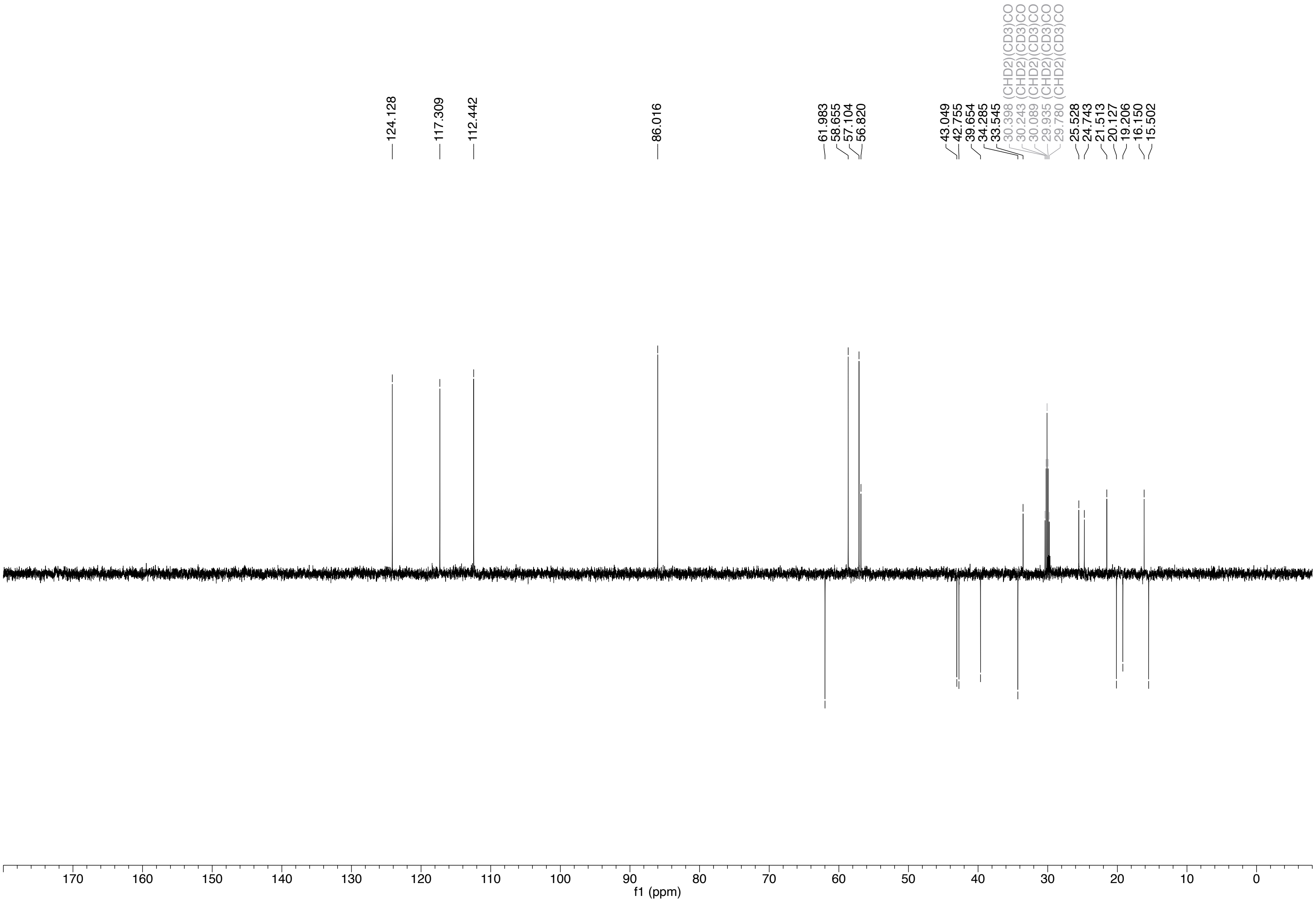

DEPT135 NMR spectrum of zigralone B (**2**) (125.78 MHz, 298K, $(CD_3)_2CO$)

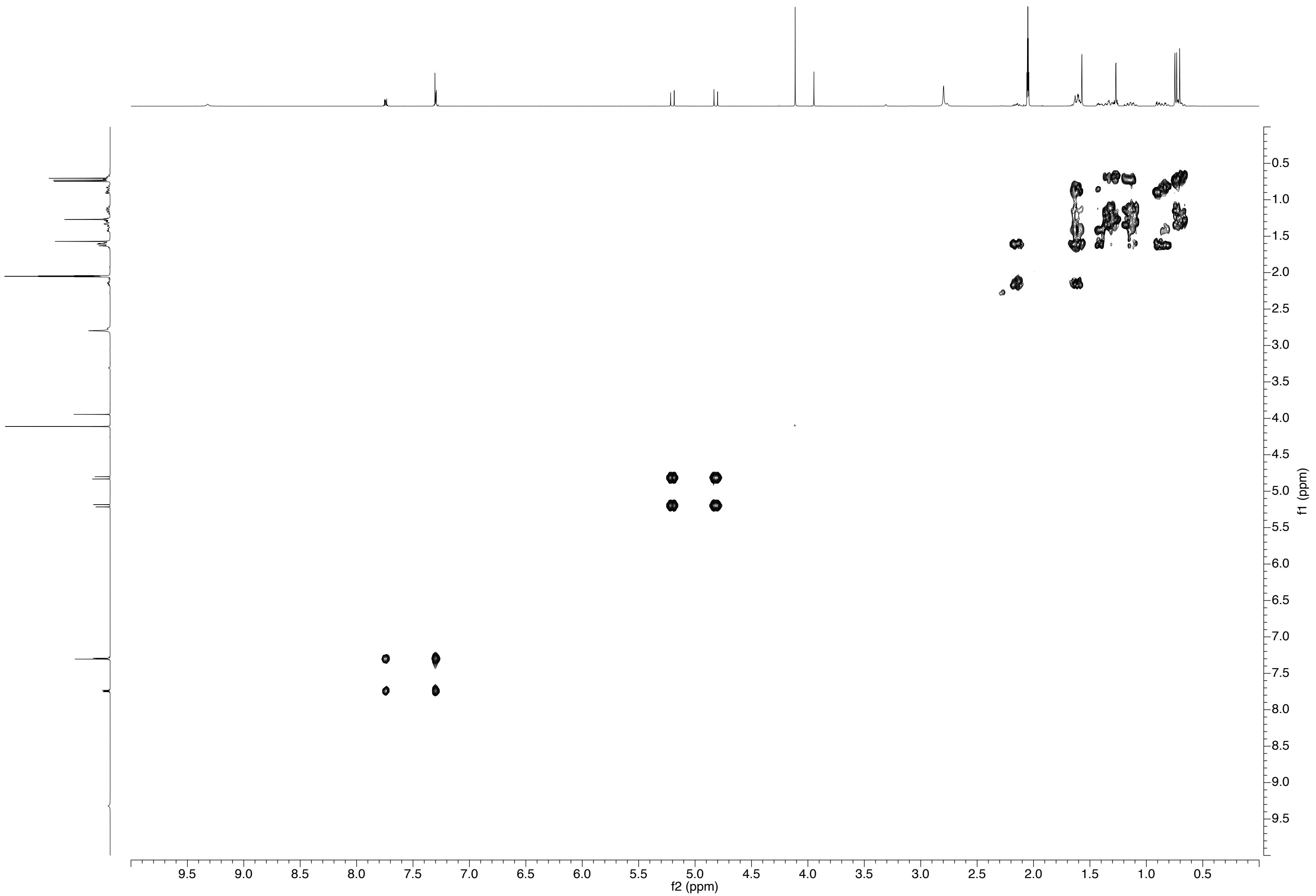

gCOSY spectrum of zigralone B (**2**) (298K, $(CD_3)_2CO$)

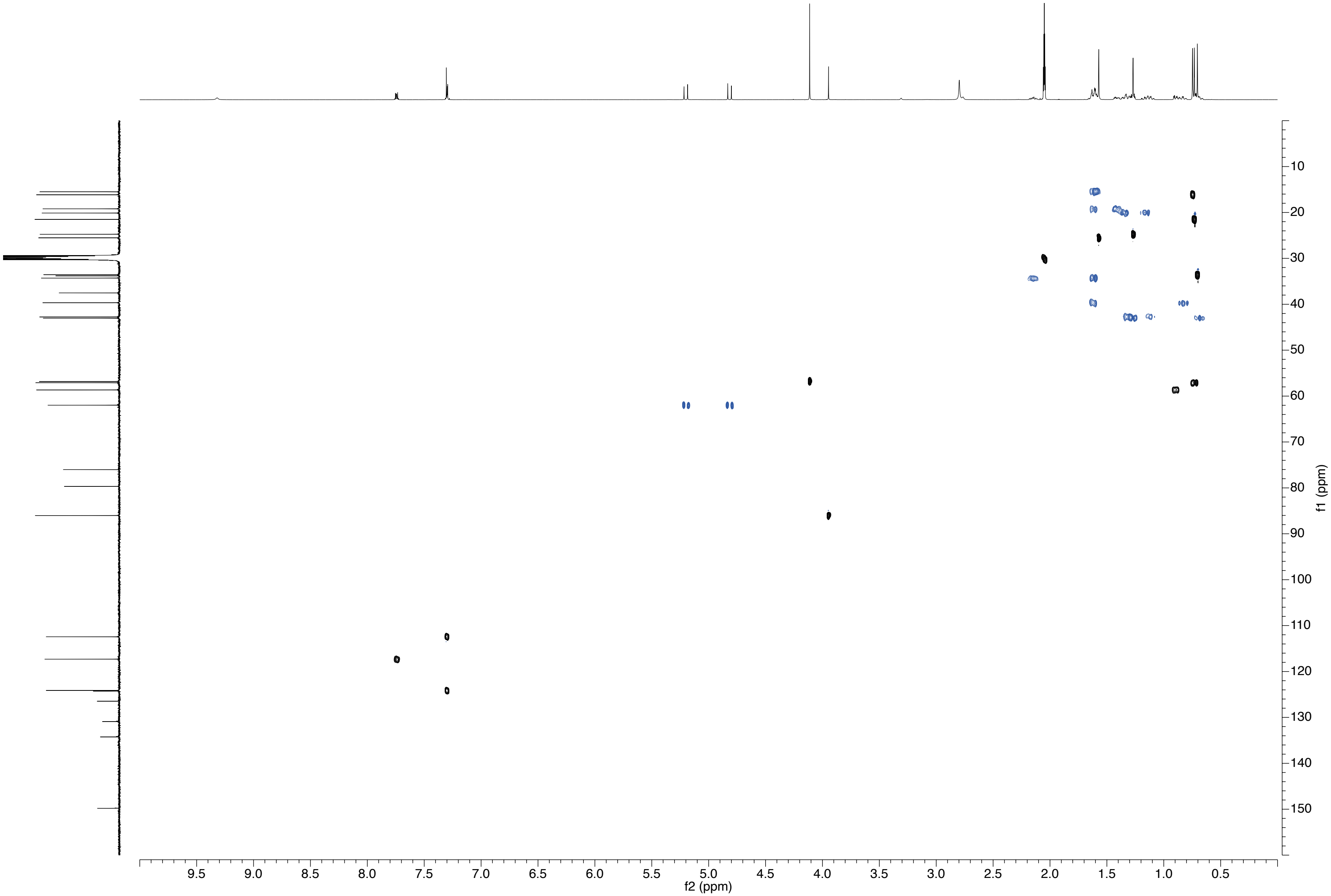

gHSQC spectrum of zigralone B (**2**) (298K, $(CD_3)_2CO$)

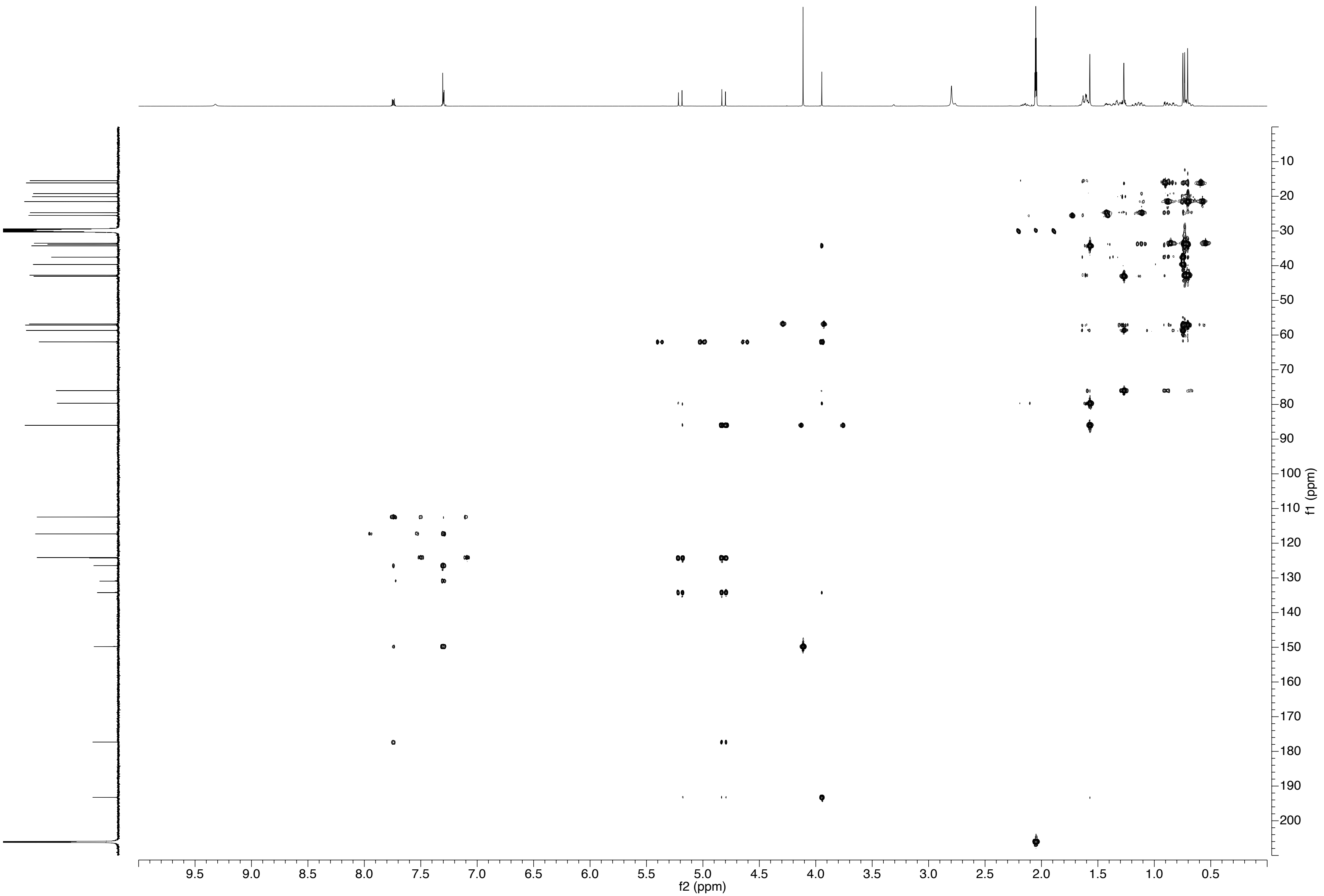

gHMBC spectrum of zigralone B (**2**) (298K, $(CD_3)_2CO$)

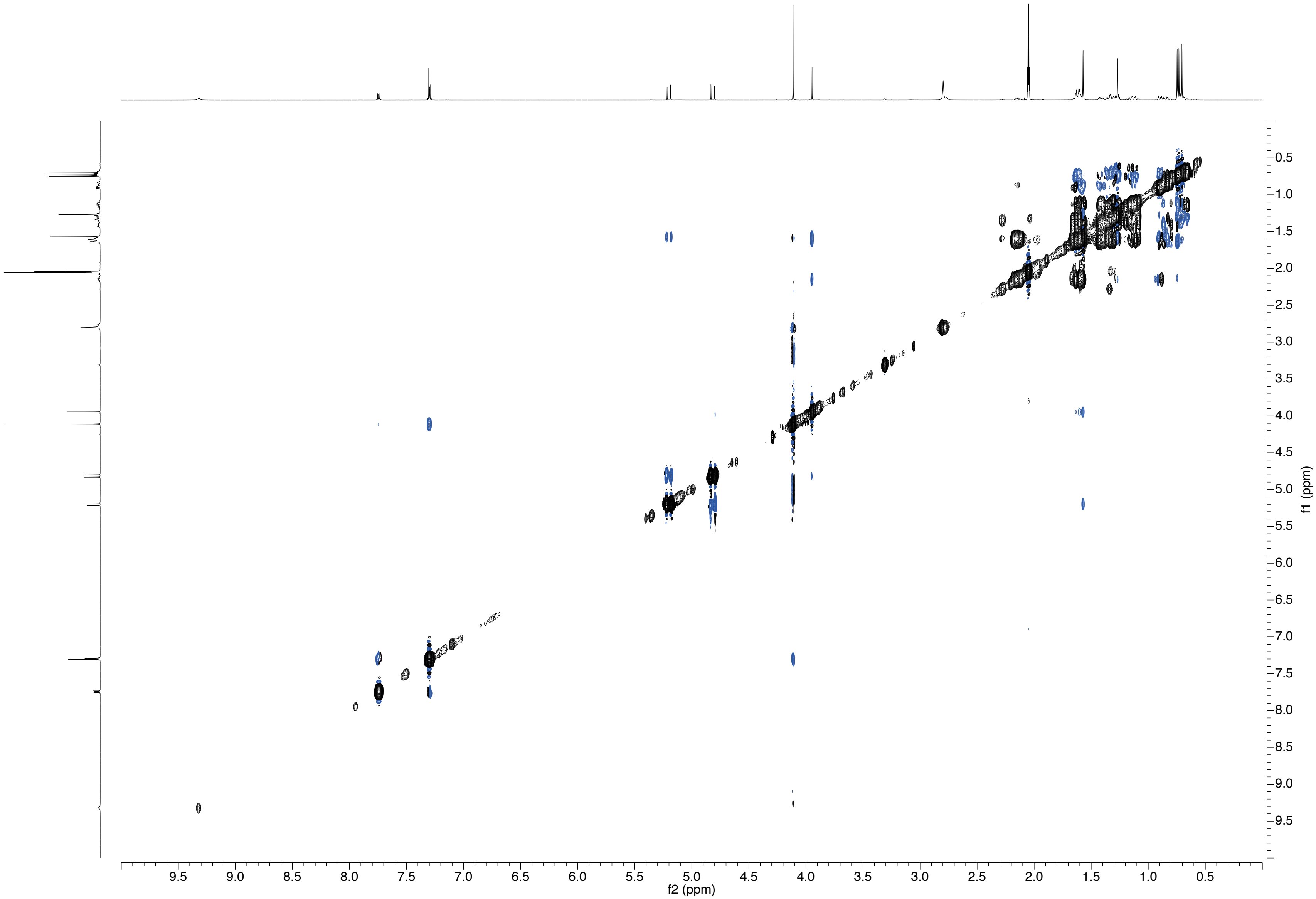

gROESY spectrum of zigralone B (**2**) (298K, $(CD_3)_2CO$)

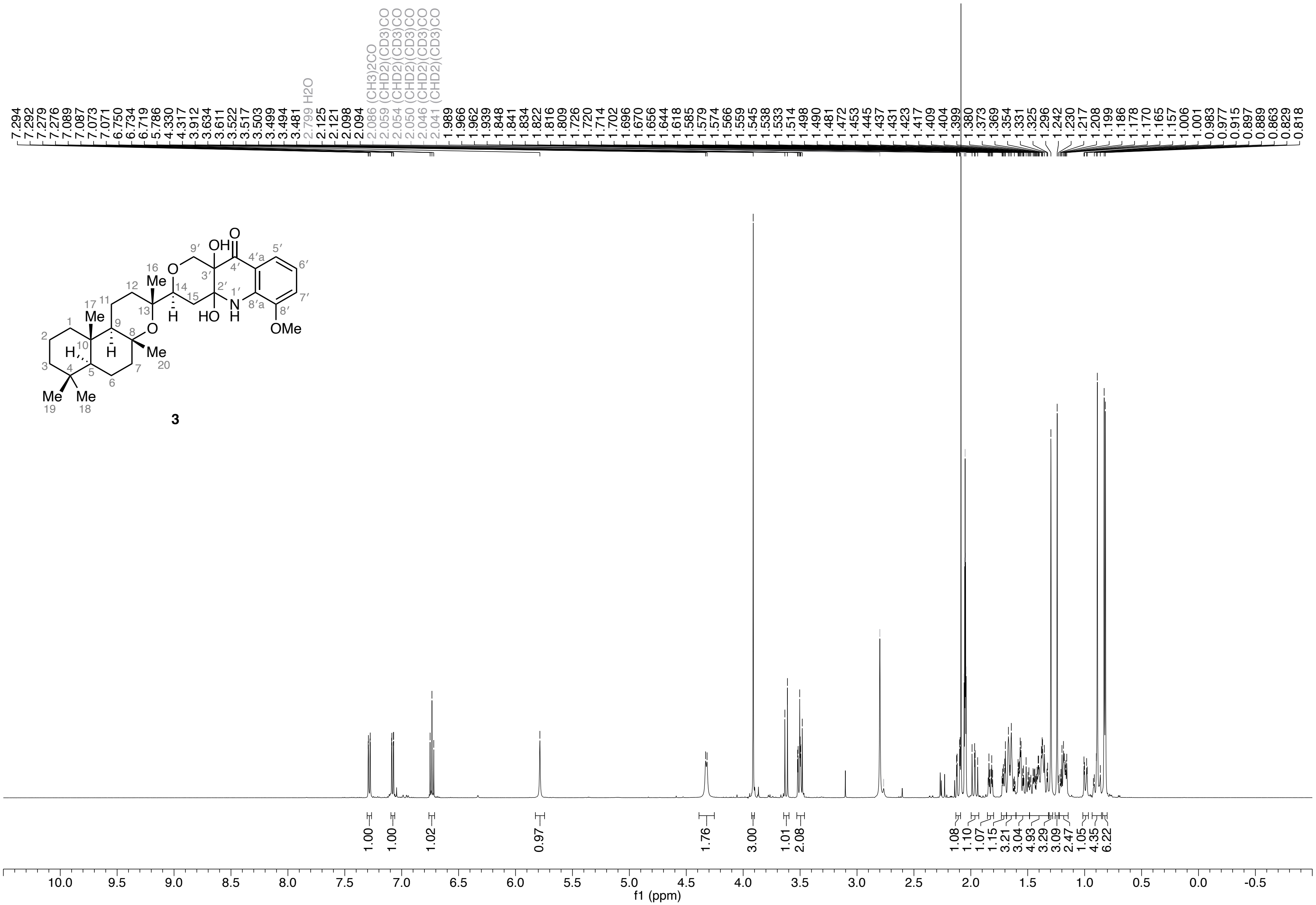

^{1}H NMR spectrum of zigralone C (**3**) (500.18 MHz, 298K, $(CD_3)_2CO$)

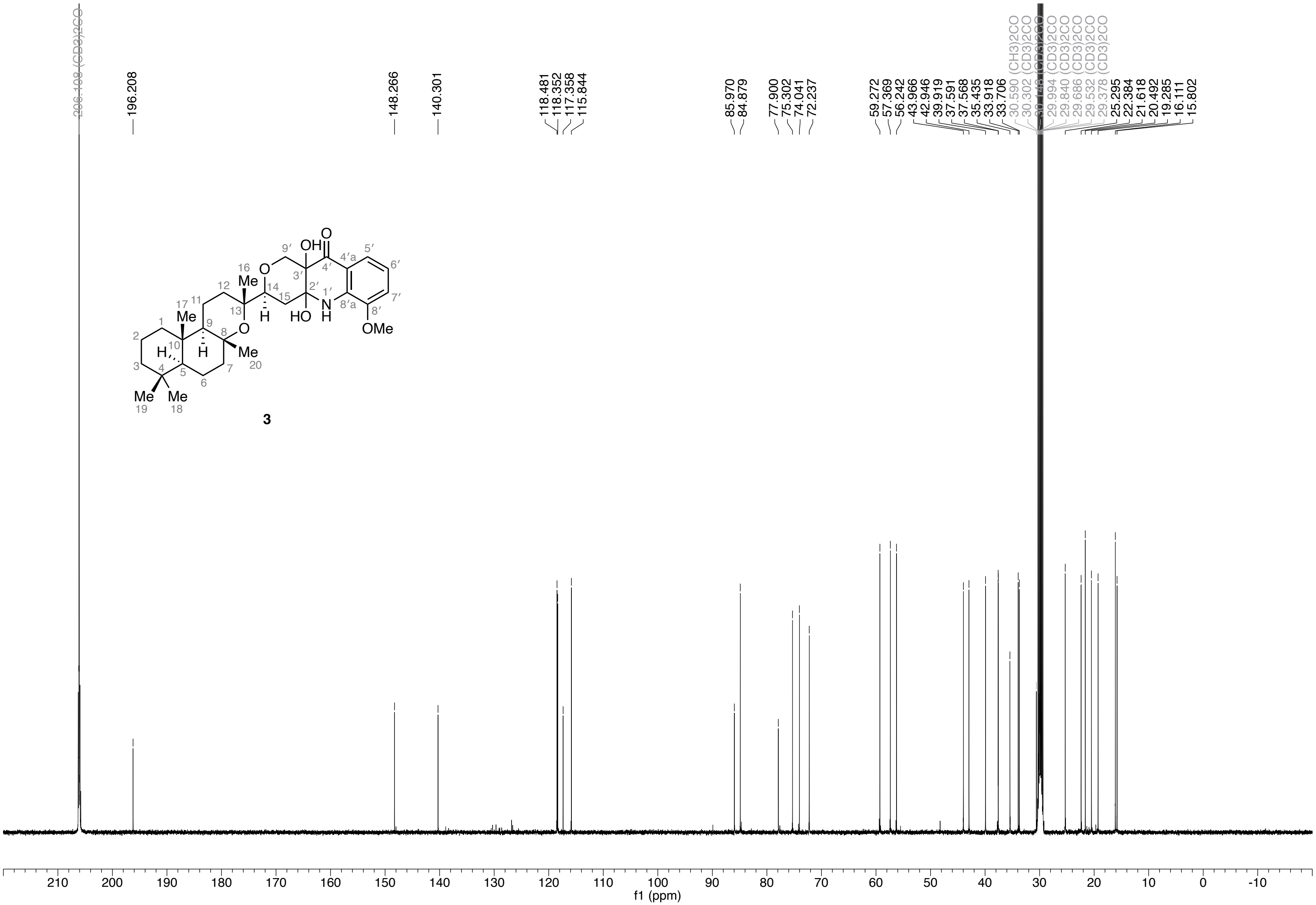

$^{13}C\{^1H\}$ NMR spectrum of zigralone C (**3**) (125.78 MHz, 298K, $(CD_3)_2CO$)

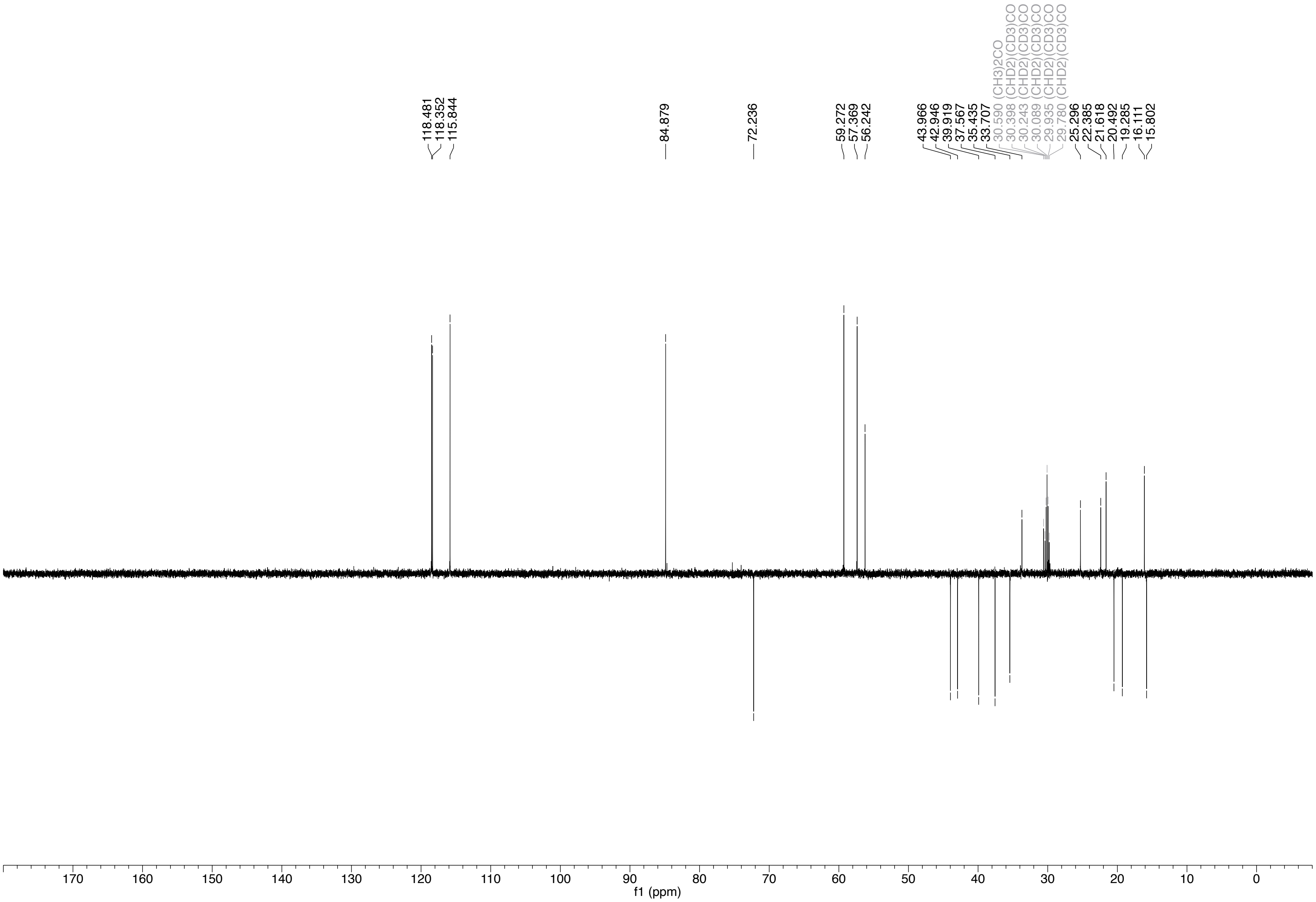

DEPT135 NMR spectrum of zigralone C (**3**) (125.78 MHz, 298K, $(CD_3)_2CO$)

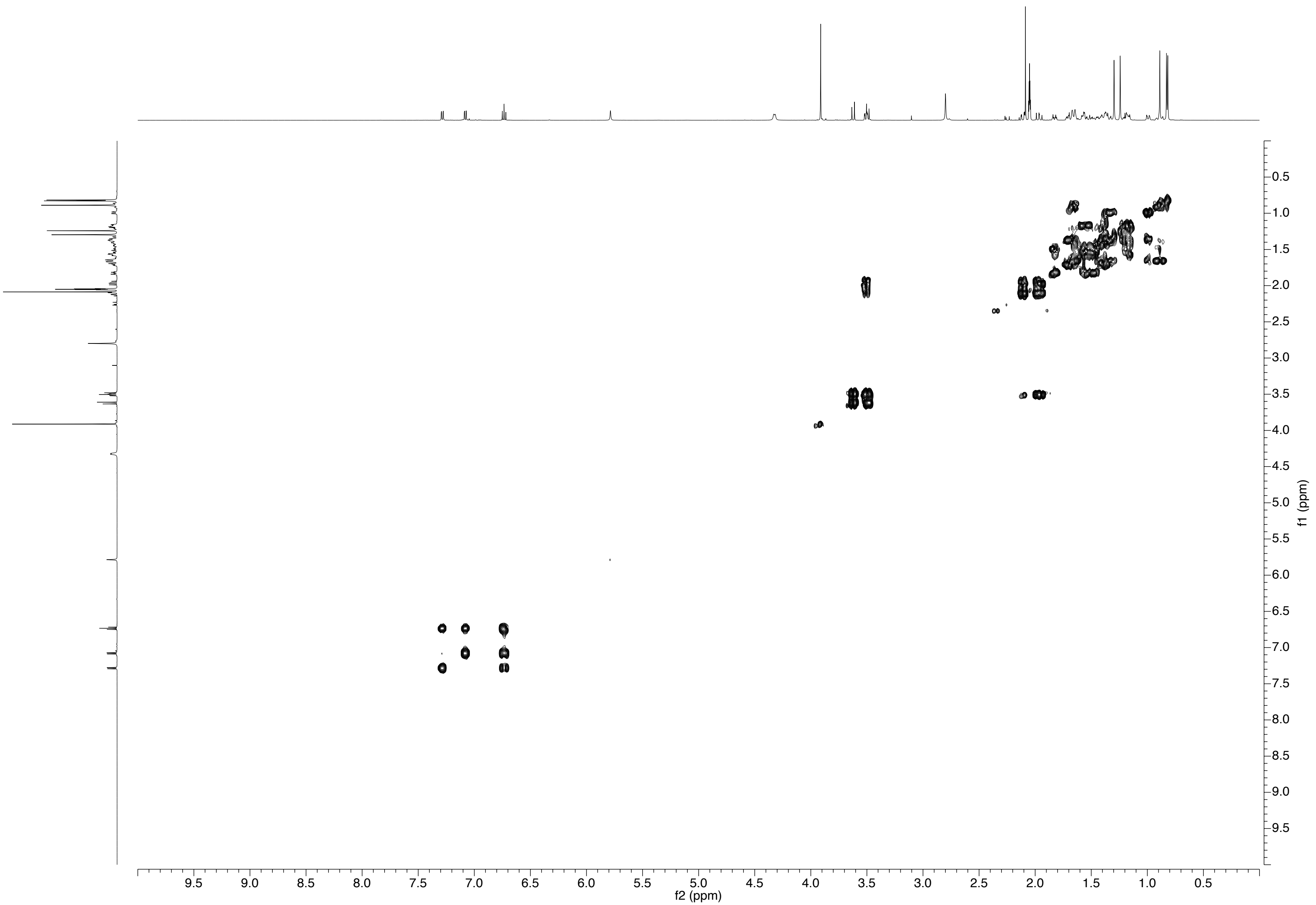

gCOSY spectrum of zigralone C (**3**) (298K, $(CD_3)_2CO$)

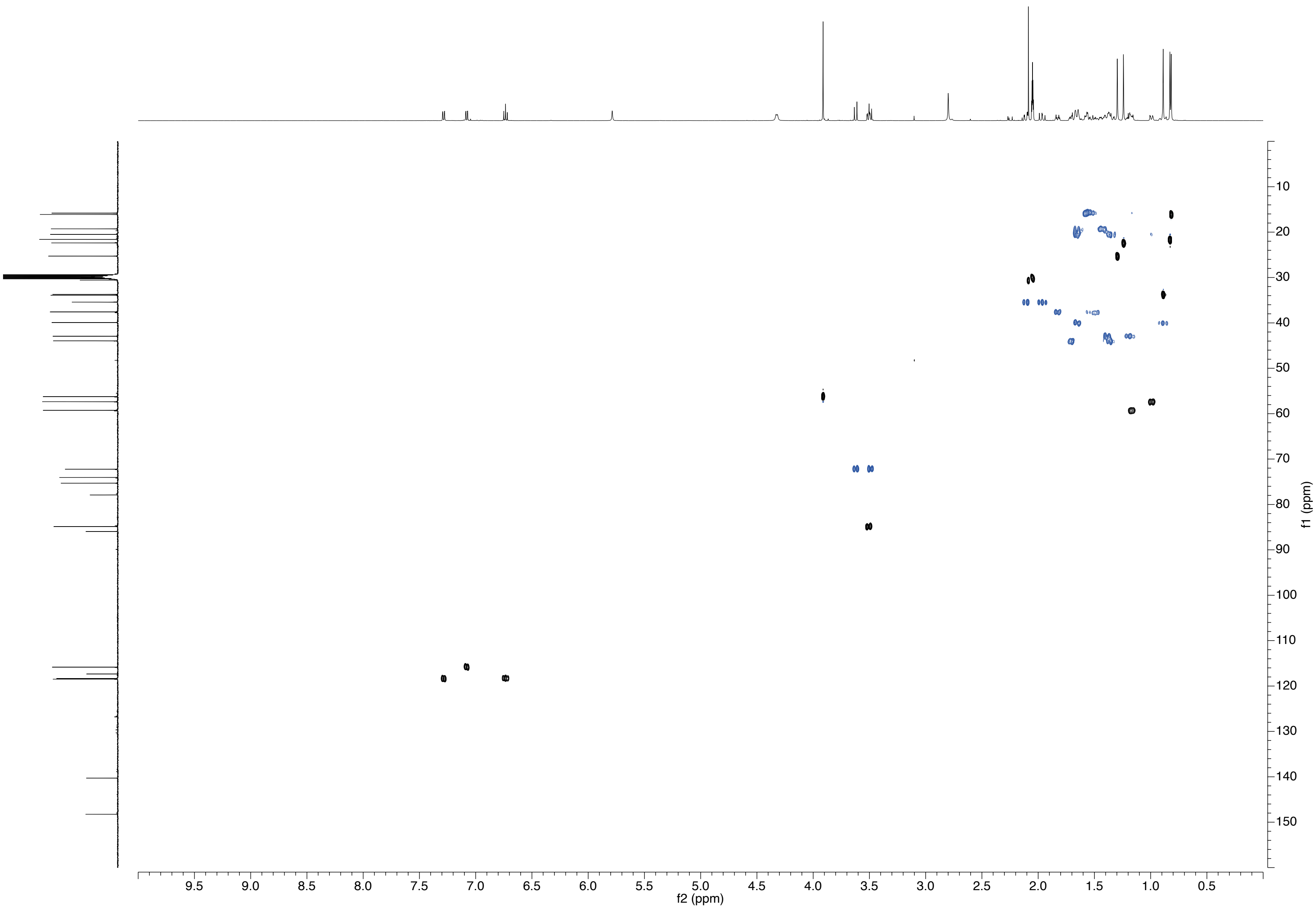

gHSQC spectrum of zigralone C (**3**) (298K, $(CD_3)_2CO$)

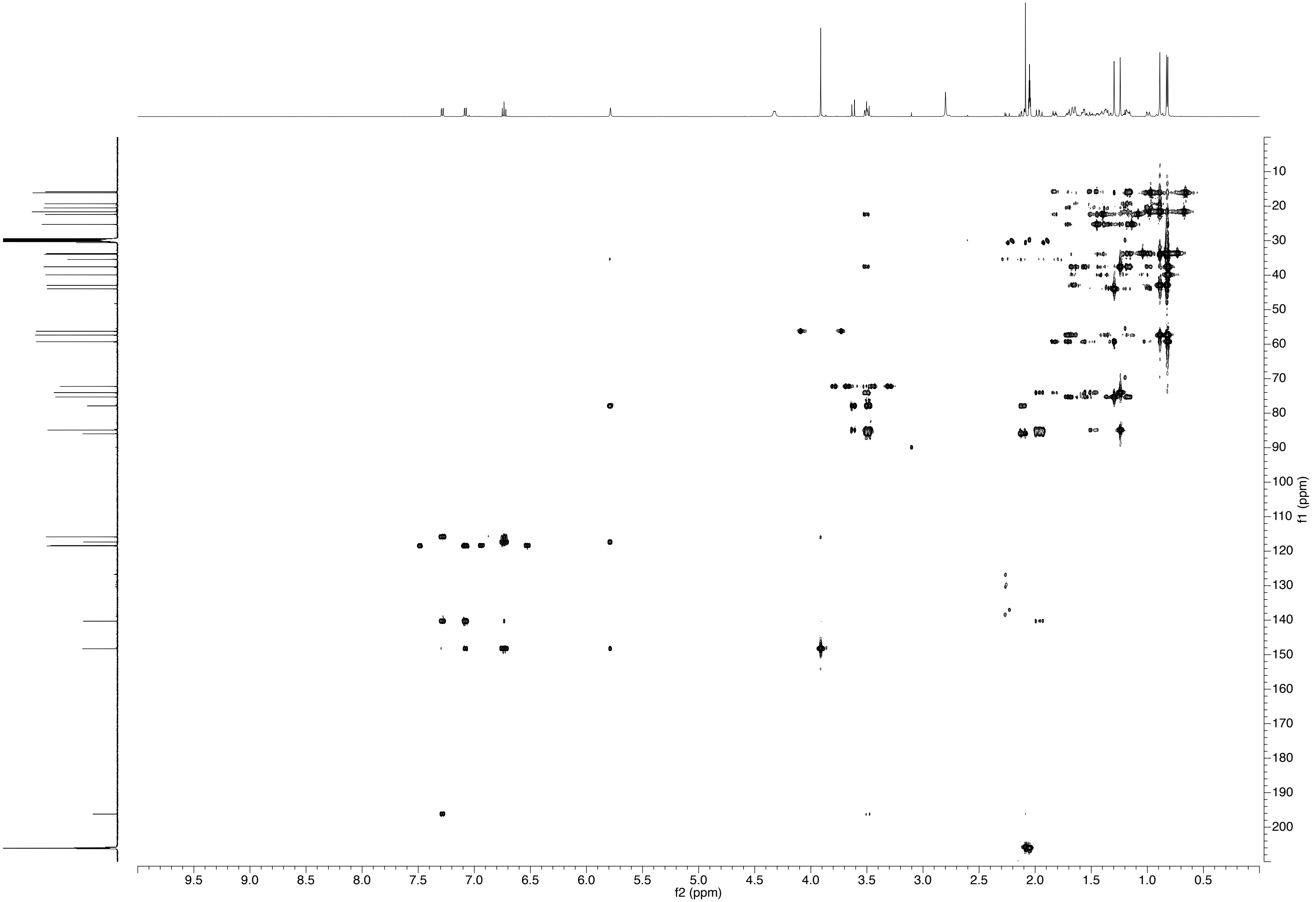

gHMBC spectrum of zigralone C (**3**) (298K, $(CD_3)_2CO$)

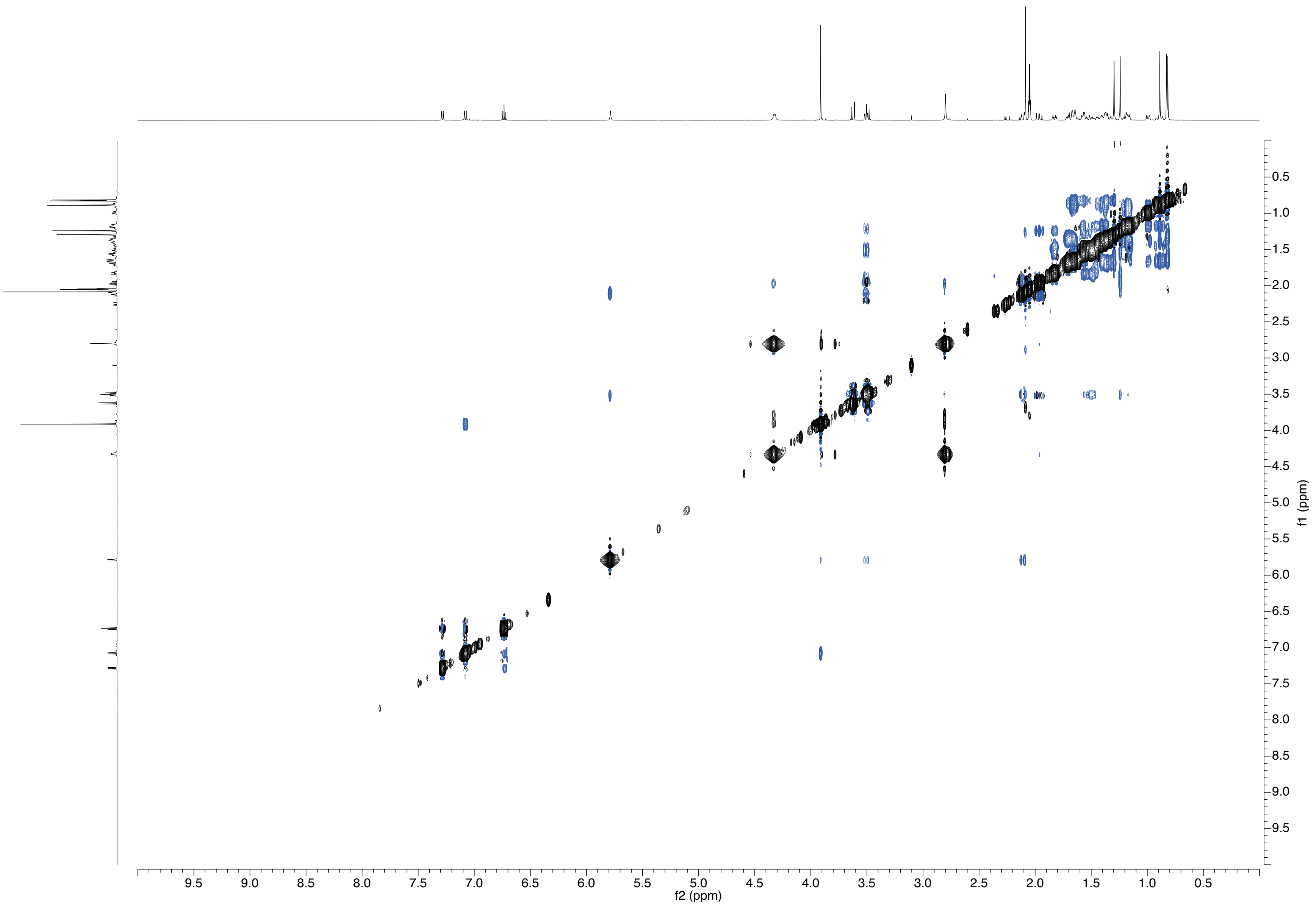

gNOESY spectrum of zigralone C (**3**) (298K, $(CD_3)_2CO$)

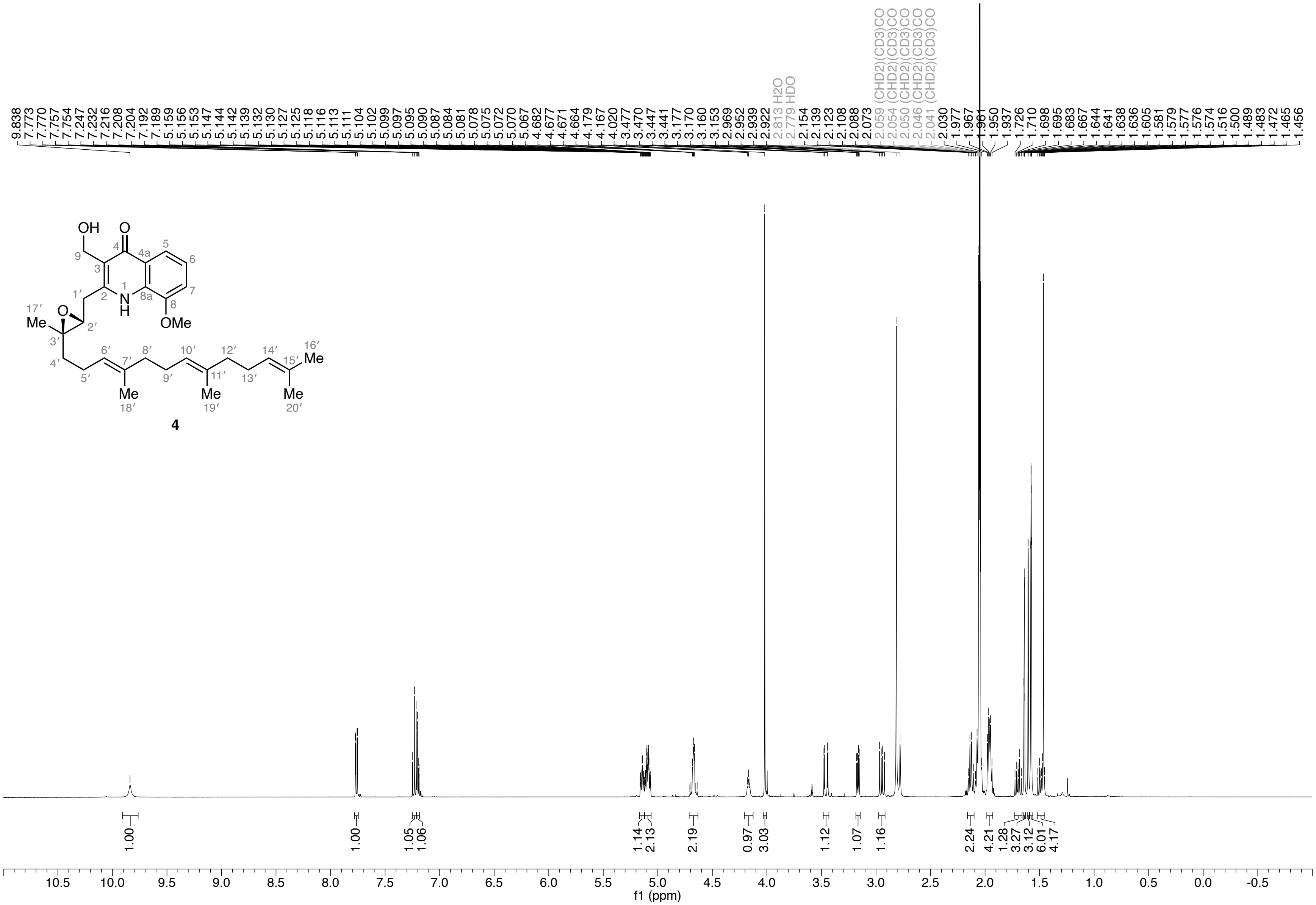

^{1}H NMR spectrum of zigralone D (**4**) (500.18 MHz, 298K, $(CD_3)_2CO$)

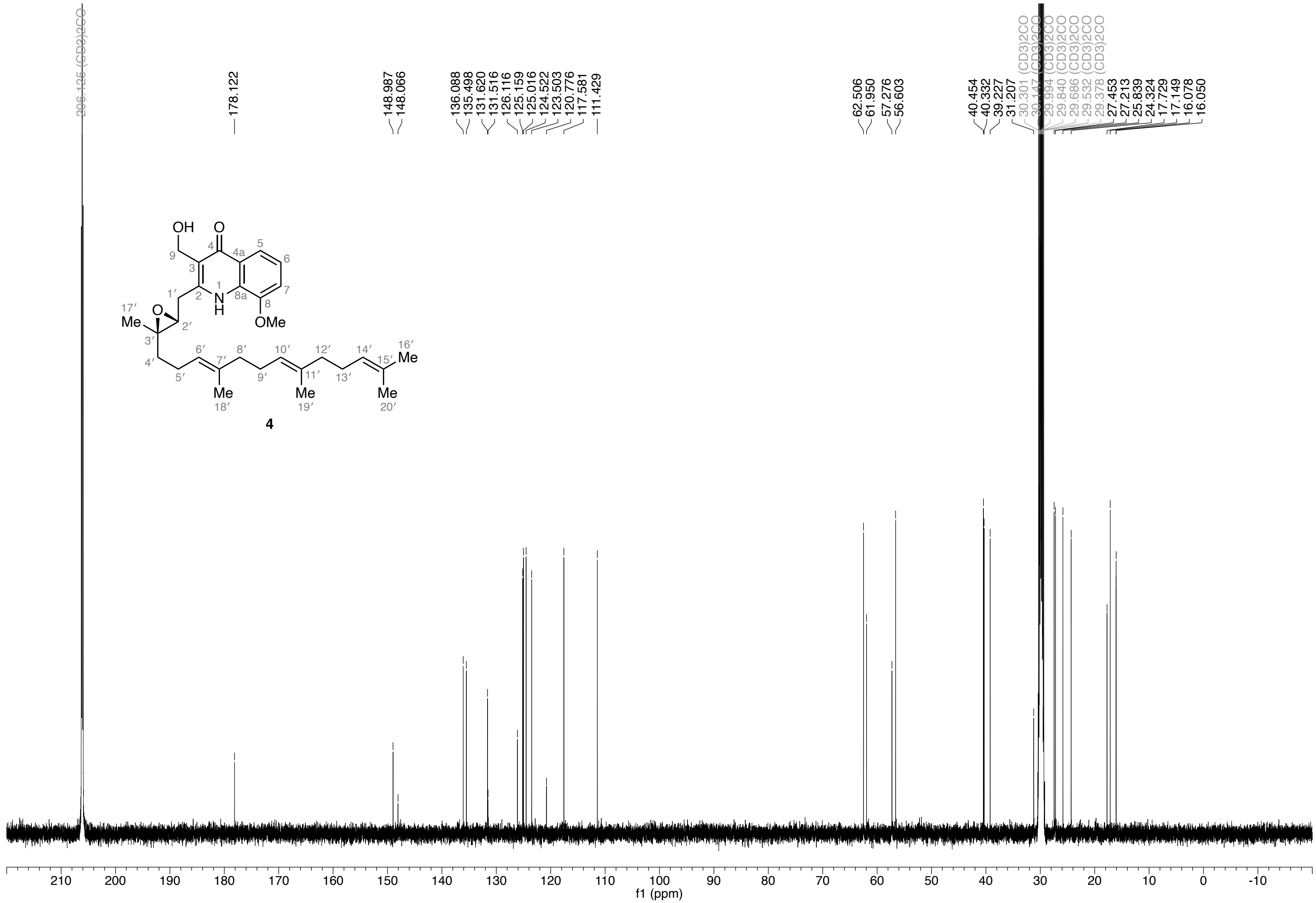

$^{13}C\{^1H\}$ NMR spectrum of zigralone D (**4**) (125.78 MHz, 298K, $(CD_3)_2CO$)

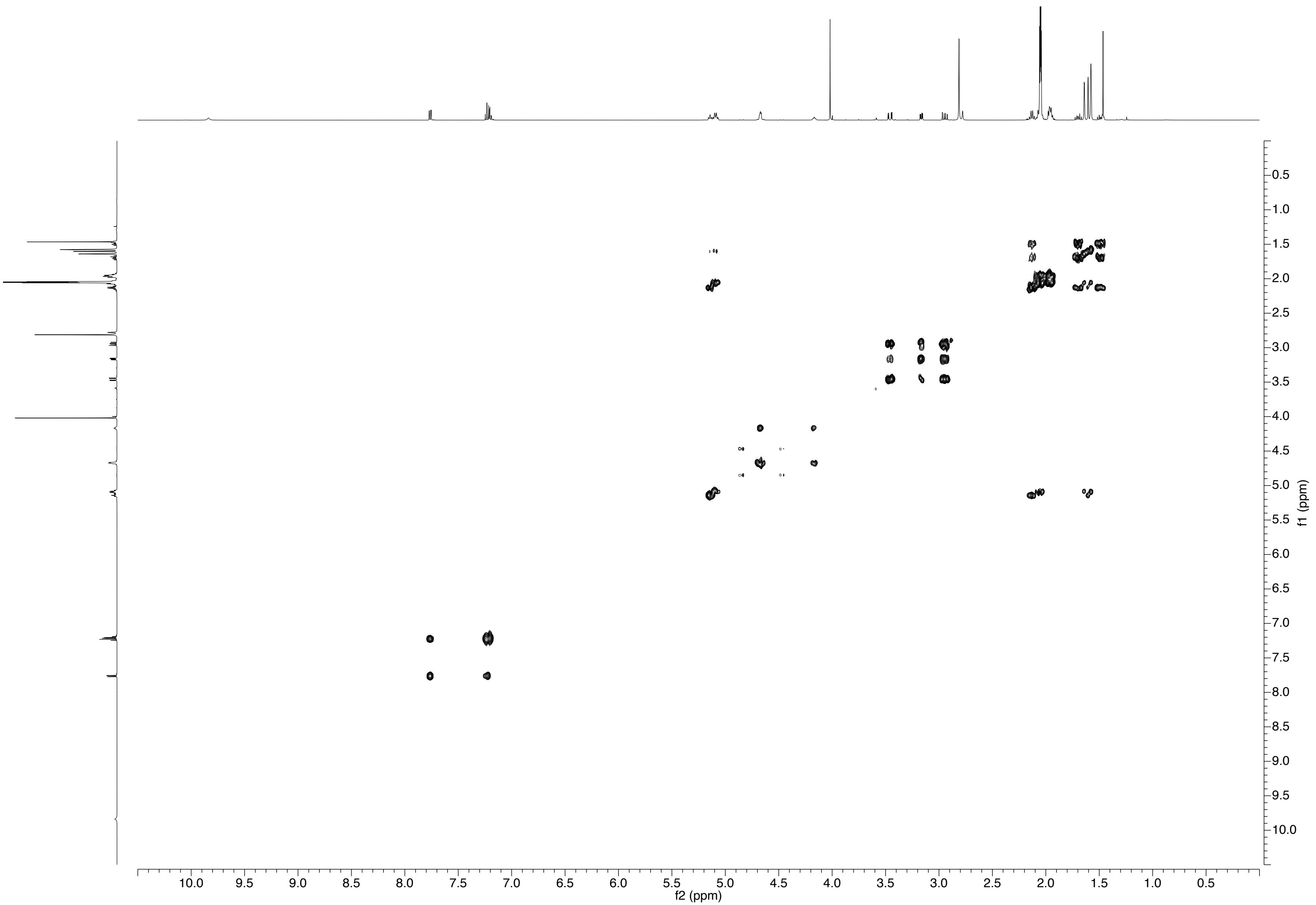

gCOSY spectrum of zigralone D (**4**) (298K, $(CD_3)_2CO$)

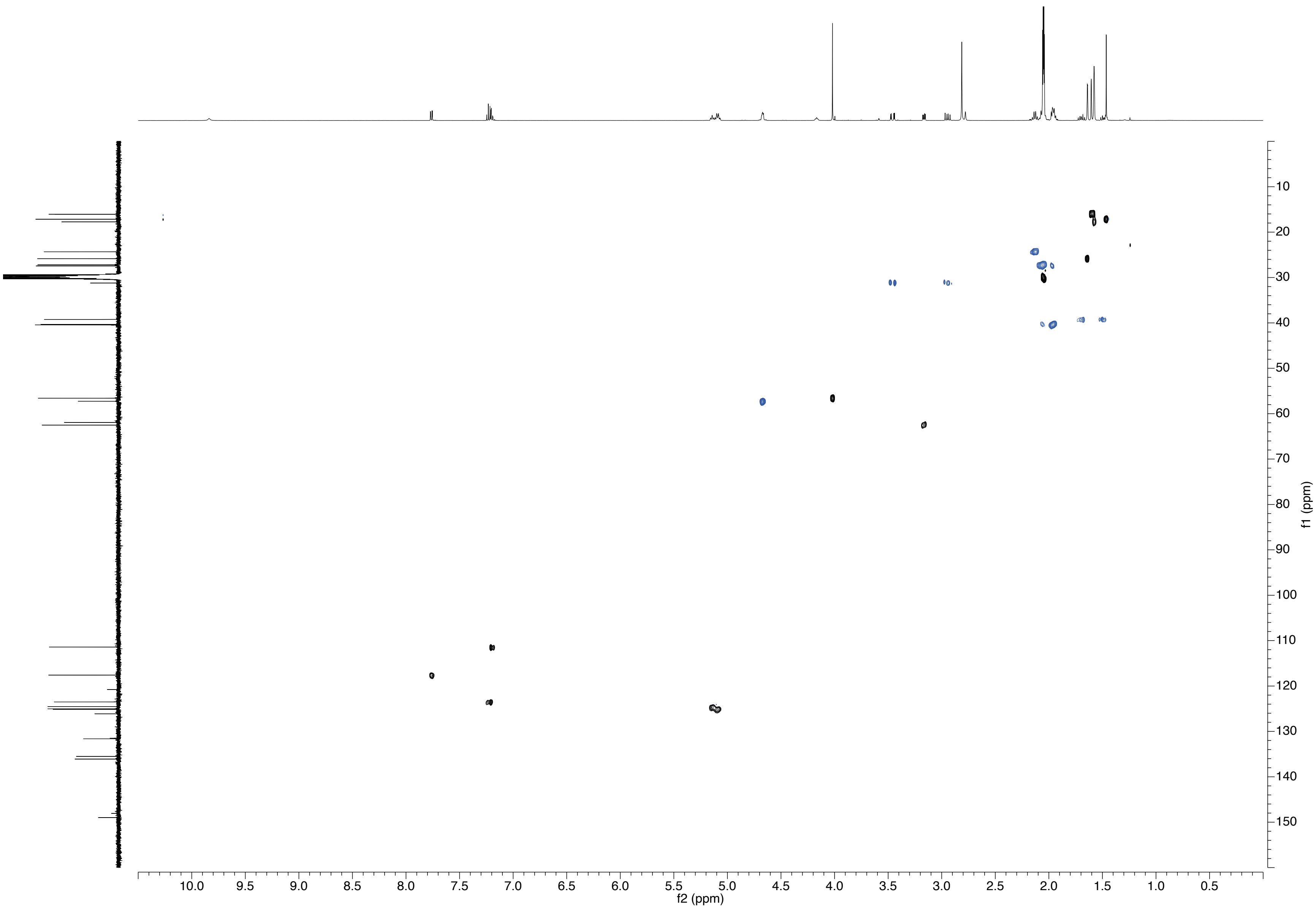

gHSQC spectrum of zigralone D (**4**) (298K, $(CD_3)_2CO$)

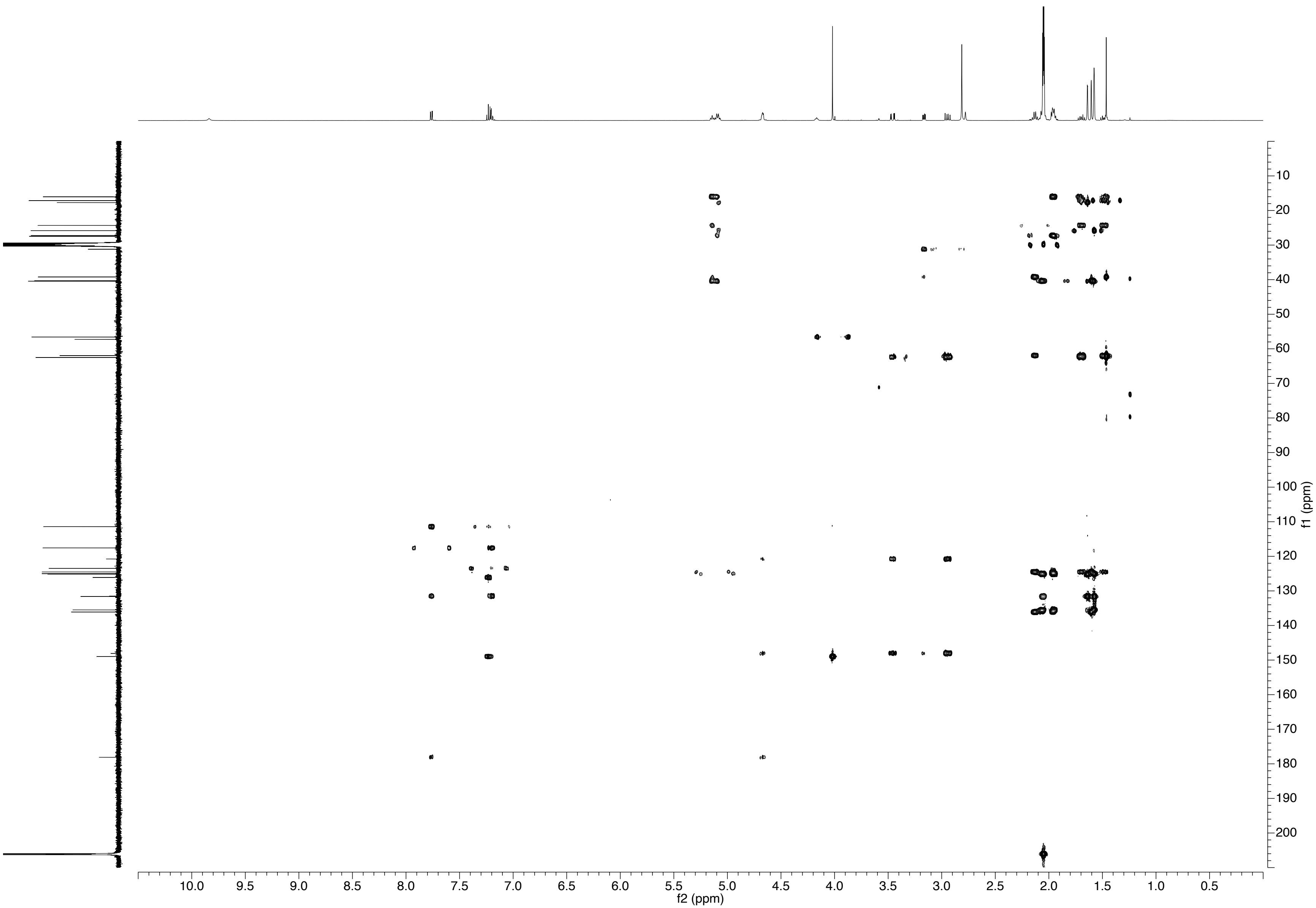

gHMBC spectrum of zigralone D (**4**) (298K, $(CD_3)_2CO$)

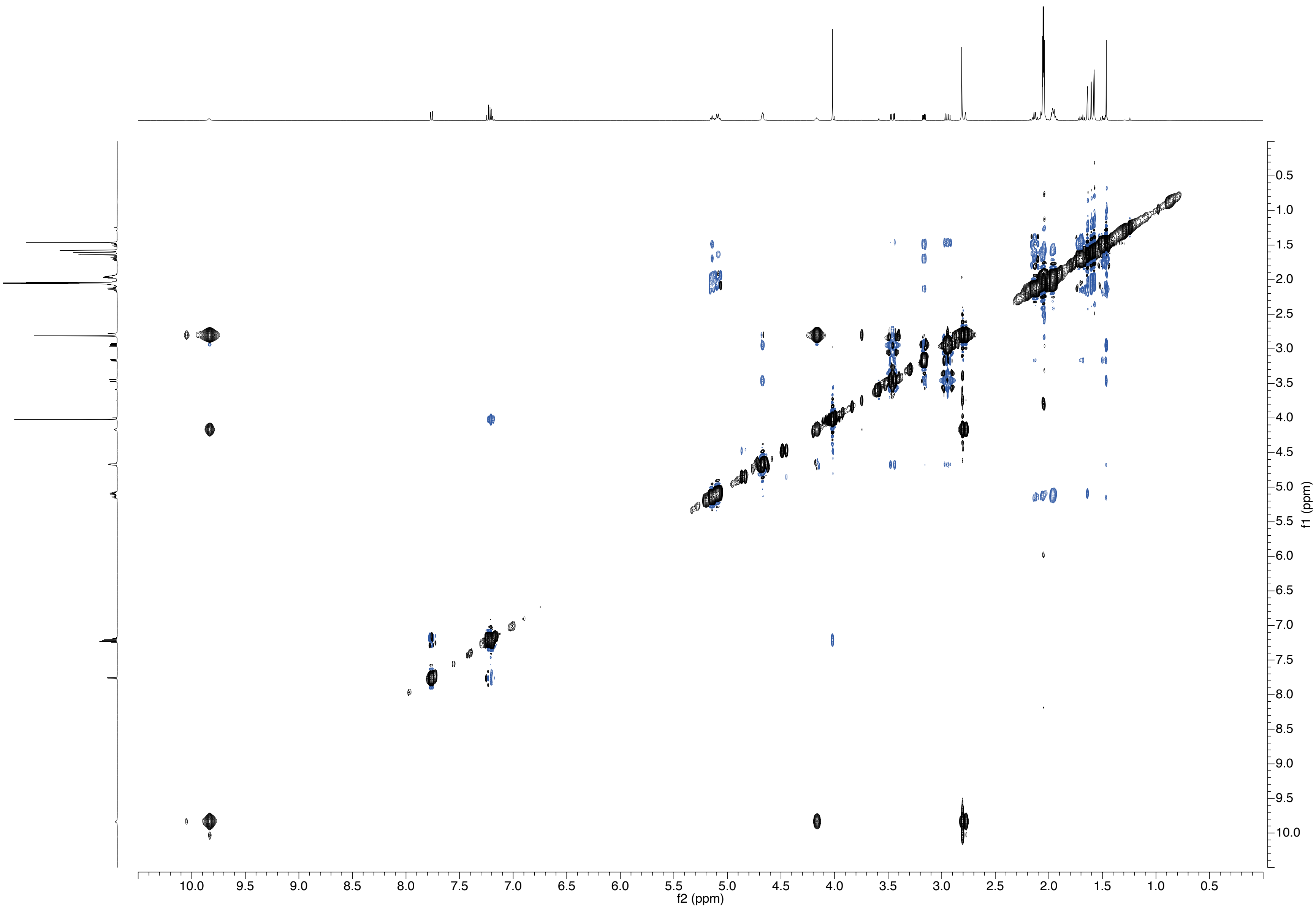

gNOESY spectrum of zigralone D (**4**) (298K, $(CD_3)_2CO$)

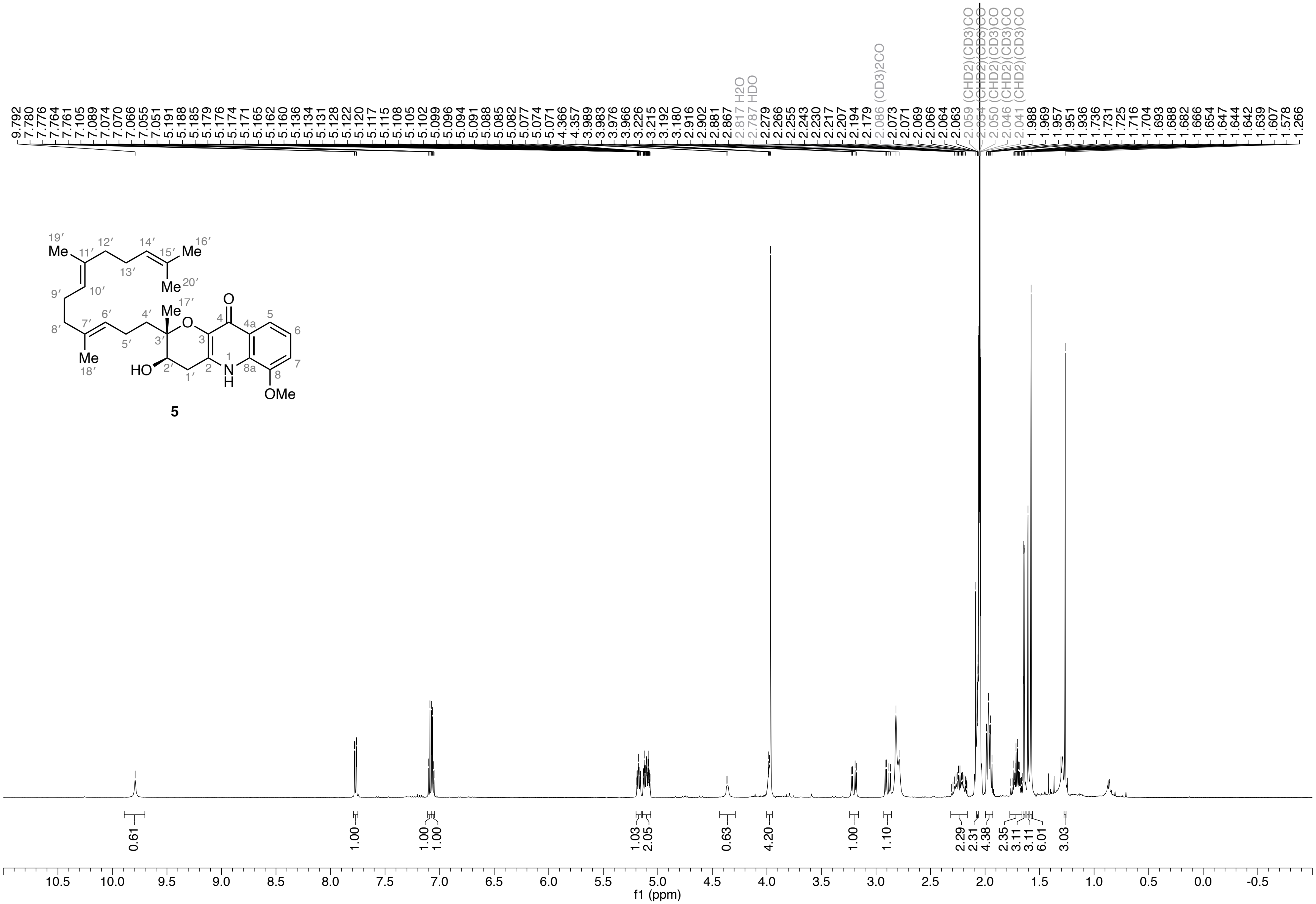

^{1}H NMR spectrum of zigralone E (**5**) (500.18 MHz, 298K, $(CD_3)_2CO$)

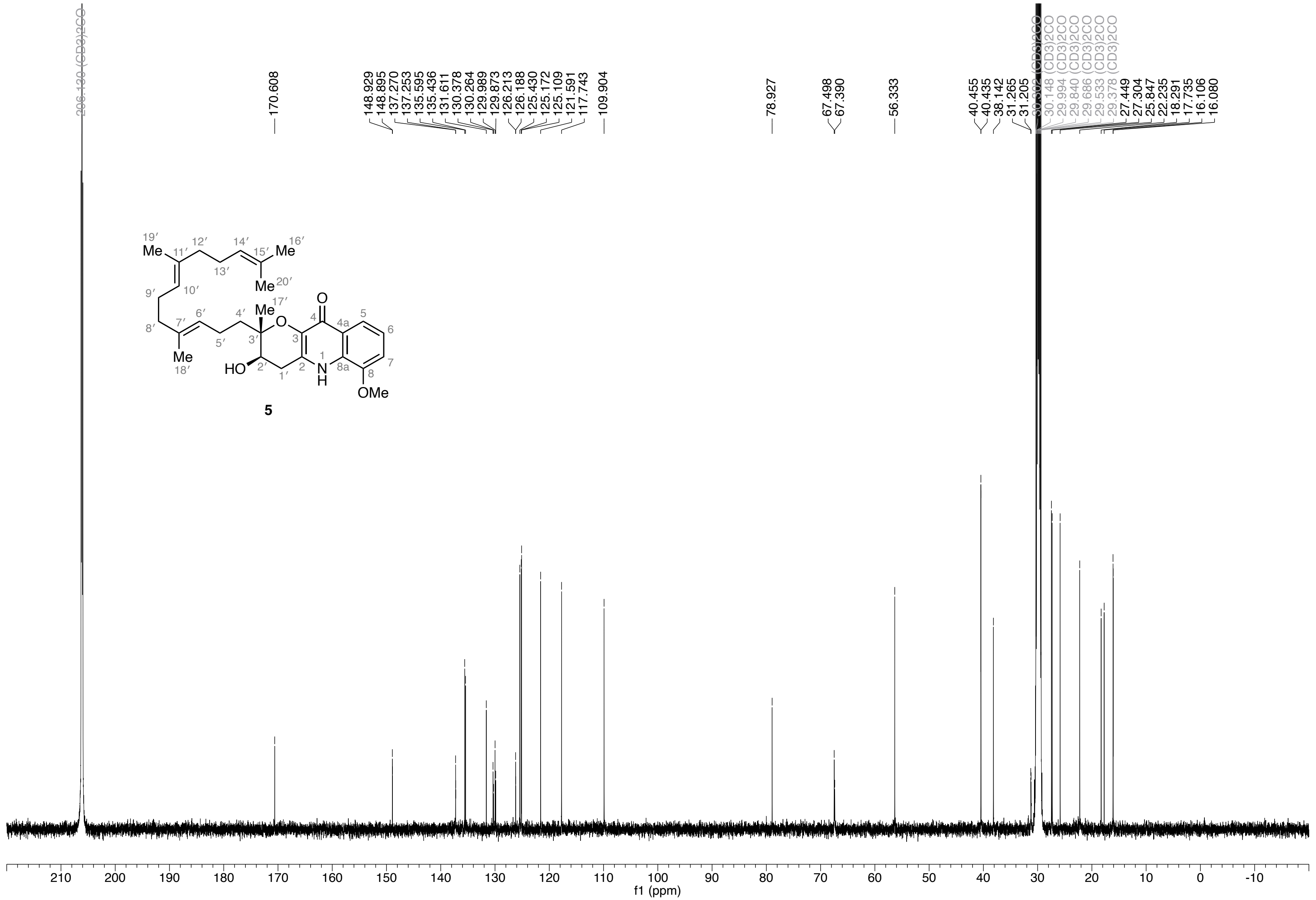

$^{13}C\{^1H\}$ NMR spectrum of zigralone E (**5**) (125.78 MHz, 298K, $(CD_3)_2CO$)

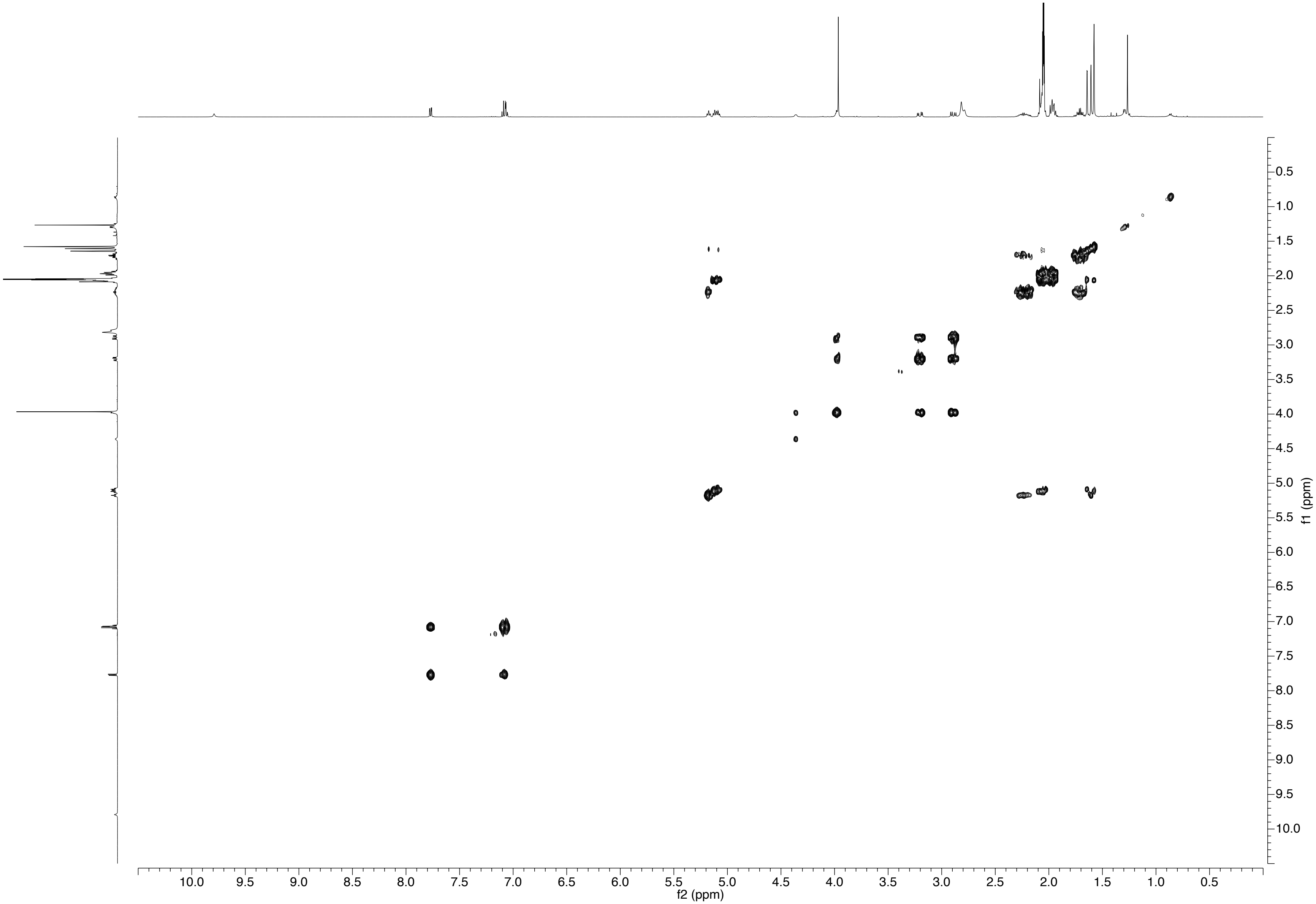

gCOSY spectrum of zigralone E (**5**) (298K, $(CD_3)_2CO$)

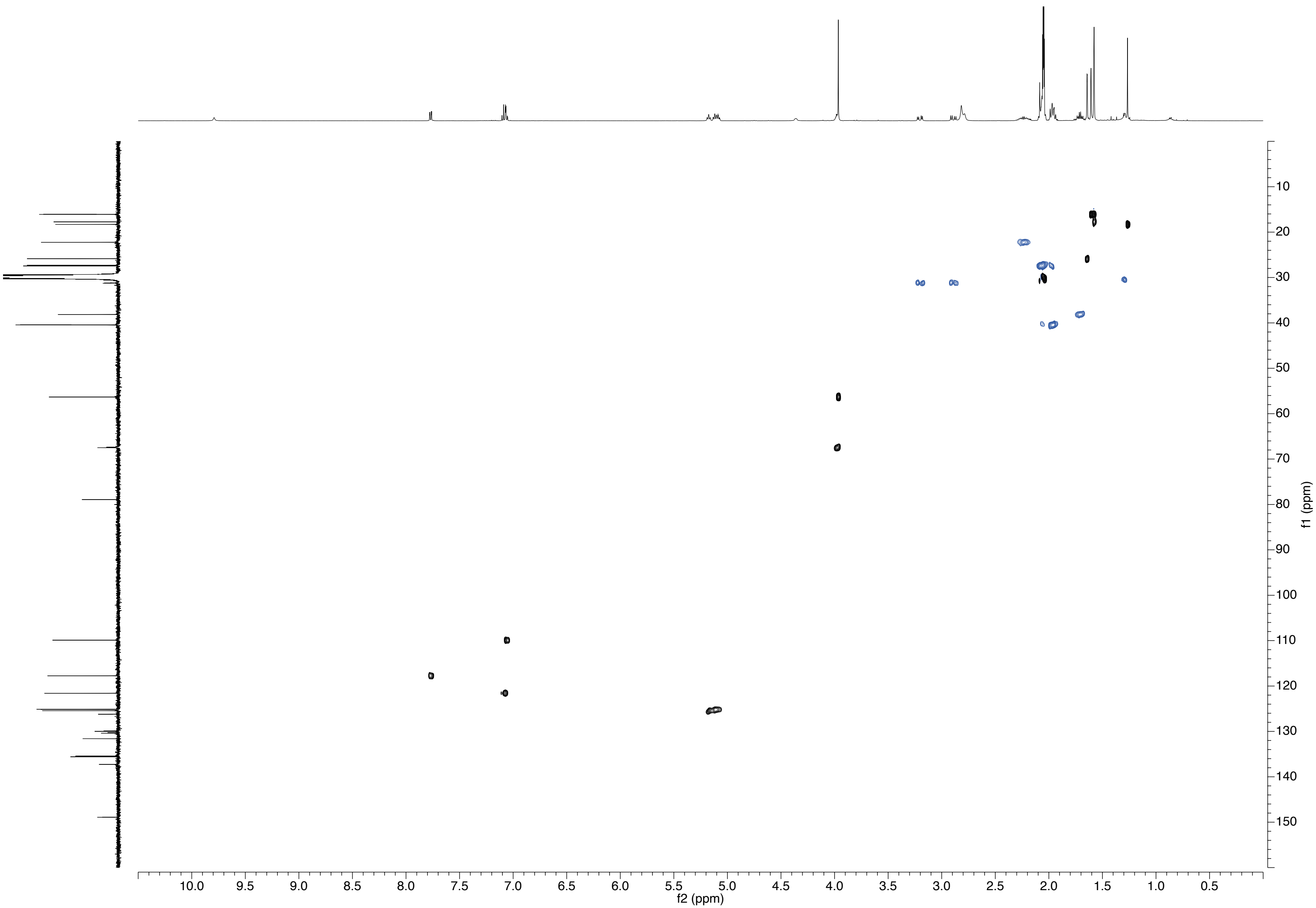

gHSQC spectrum of zigralone E (**5**) (298K, $(CD_3)_2CO$)

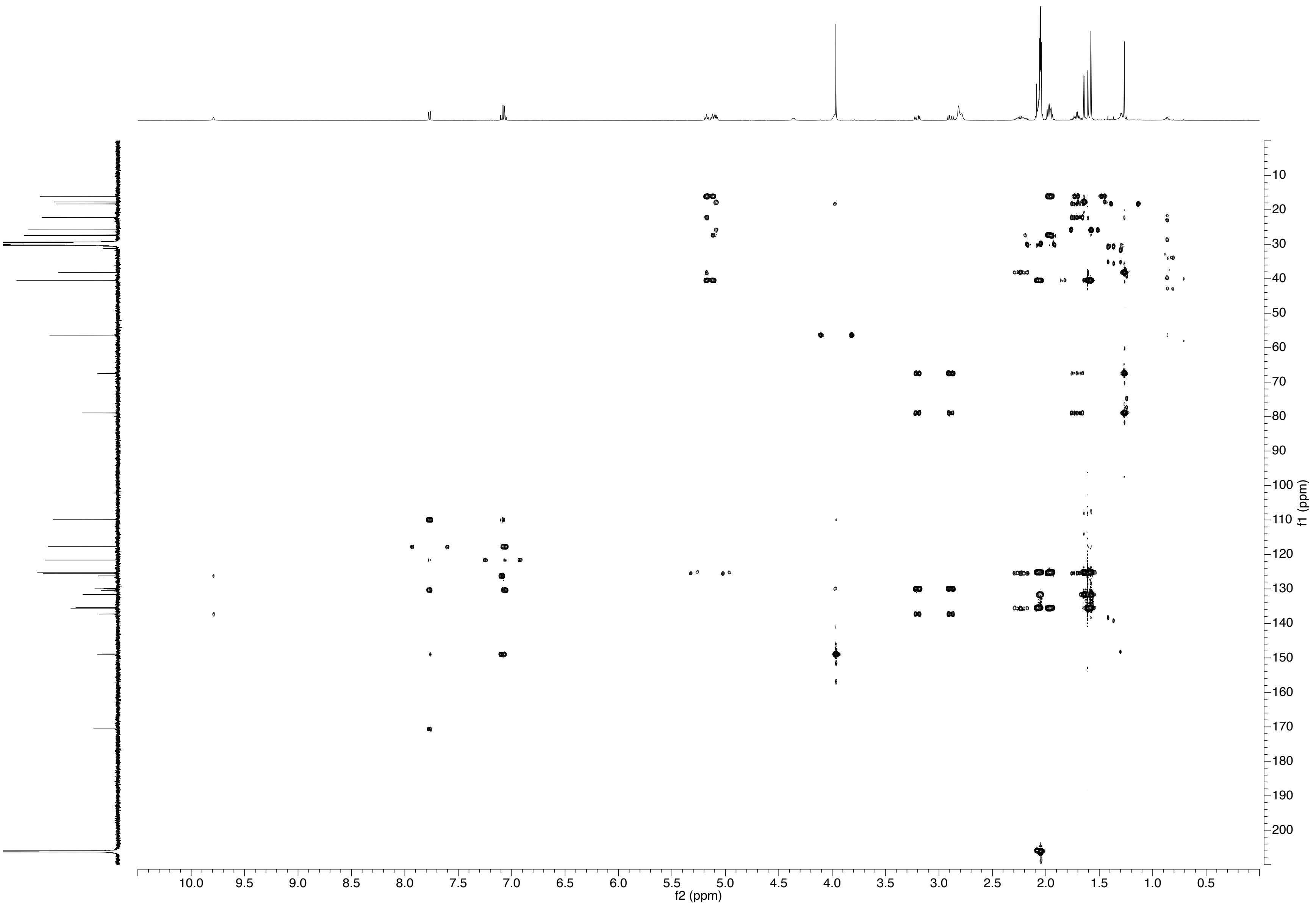

gHMBC spectrum of zigralone E (**5**) (298K, $(CD_3)_2CO$)

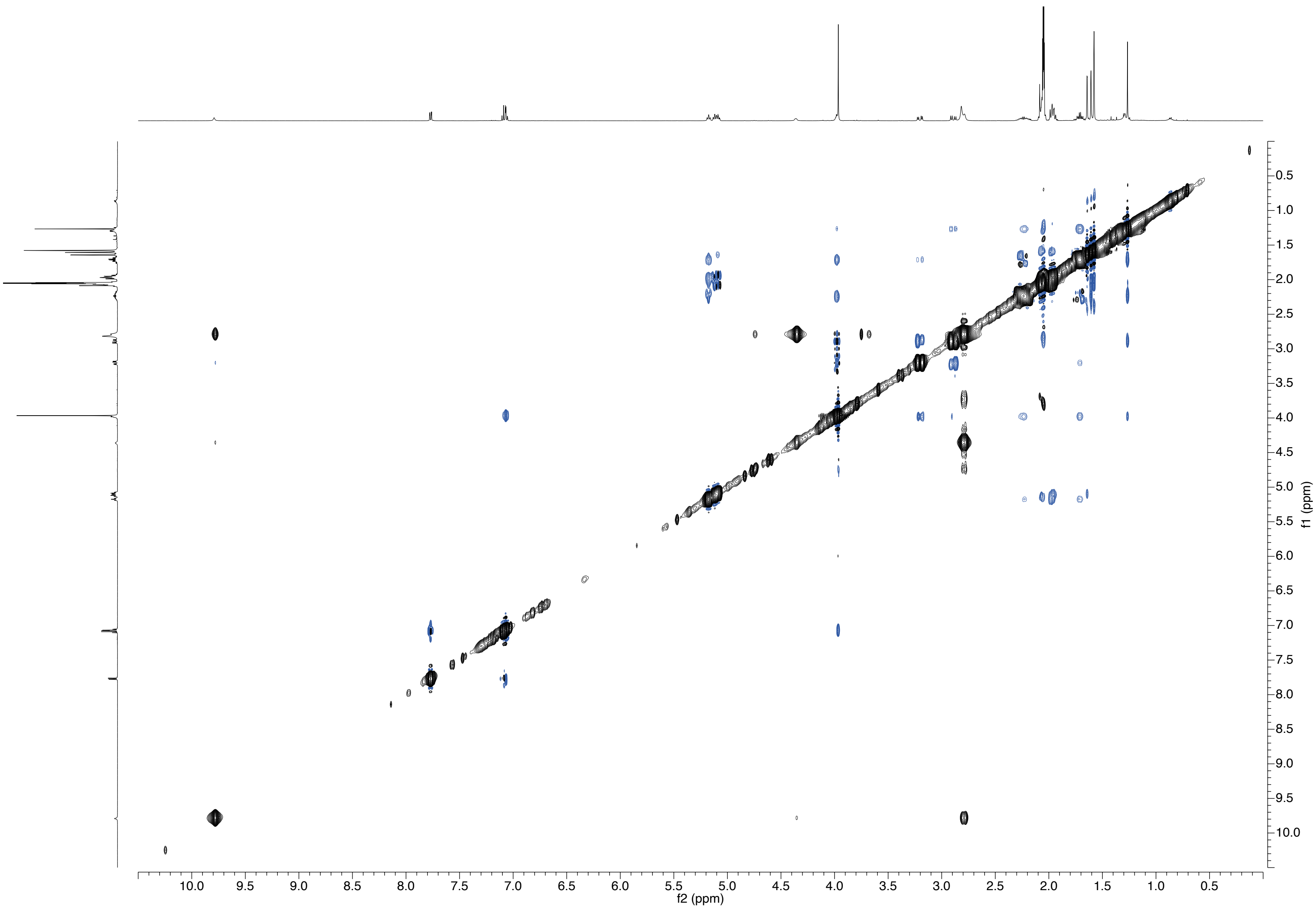

gNOESY spectrum of zigralone E (**5**) (298K, $(CD_3)_2CO$)

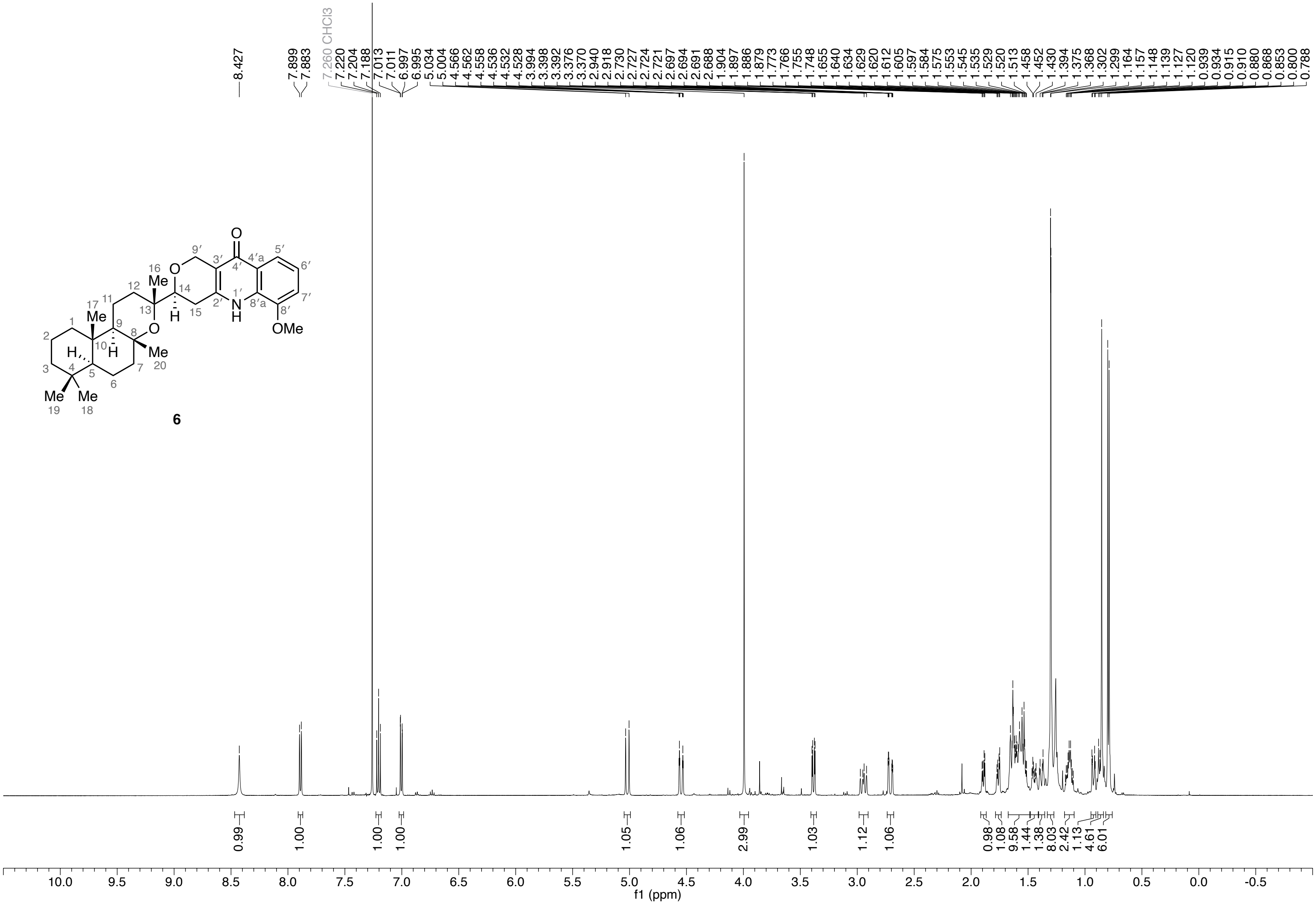

6

^{1}H NMR spectrum of zigralone F (**6**) (500.18 MHz, 298K, $CDCl_3$)

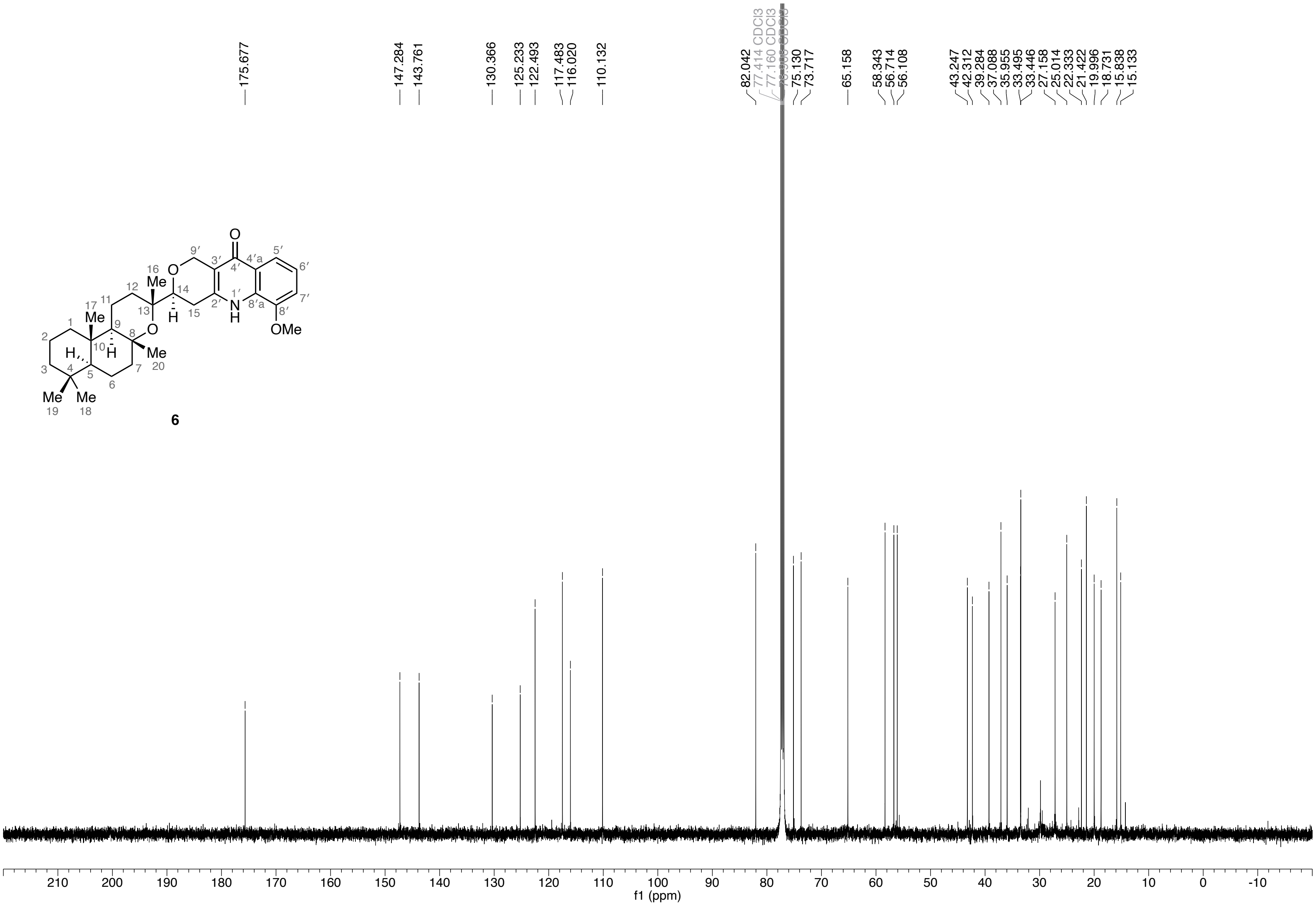

$^{13}C\{^{1}H\}$ NMR spectrum of zigralone F (**6**) (125.78 MHz, 298K, $CDCl_3$)

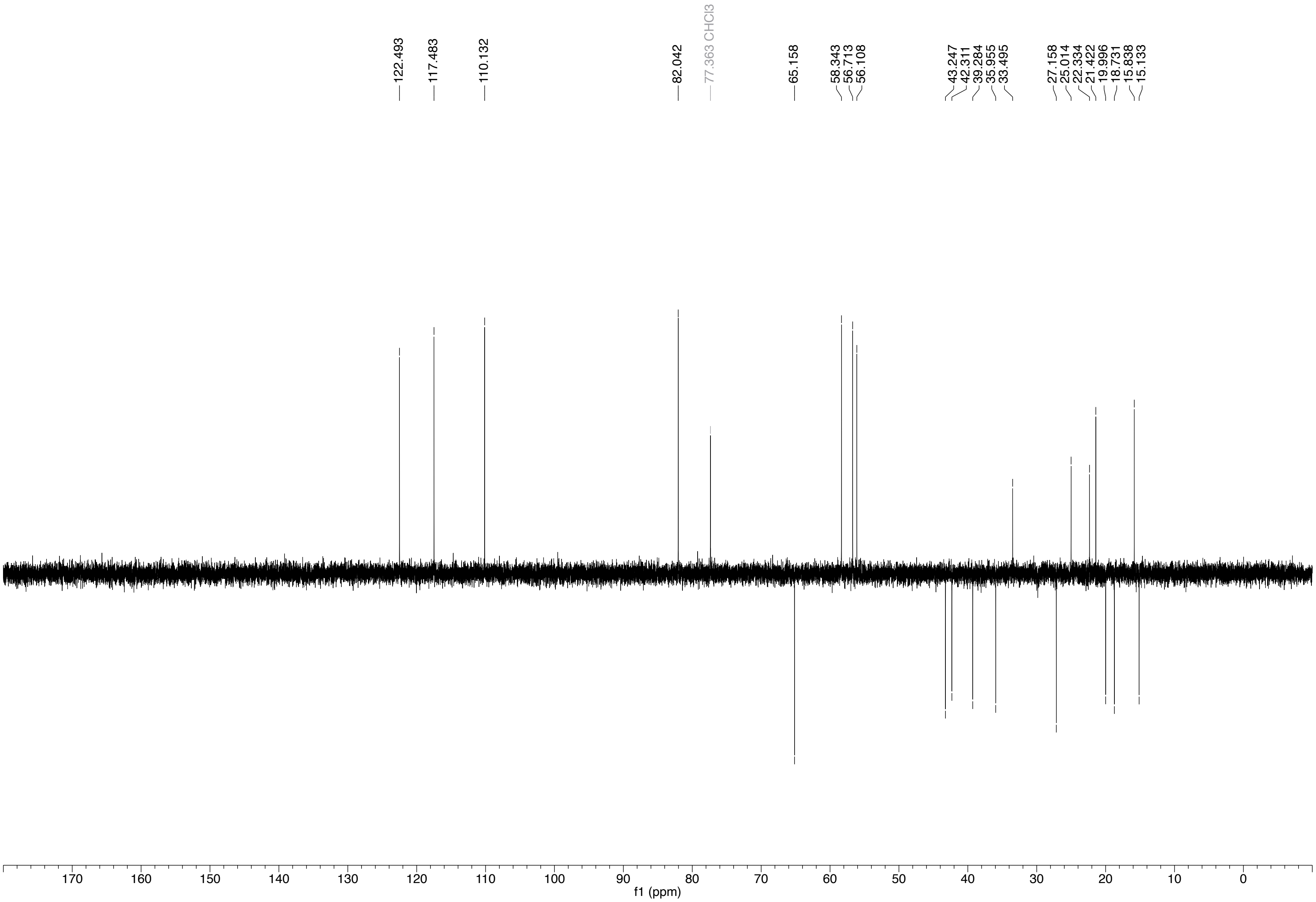

DEPT135 NMR spectrum of zigralone F (**6**) (125.78 MHz, 298K, $CDCl_3$)

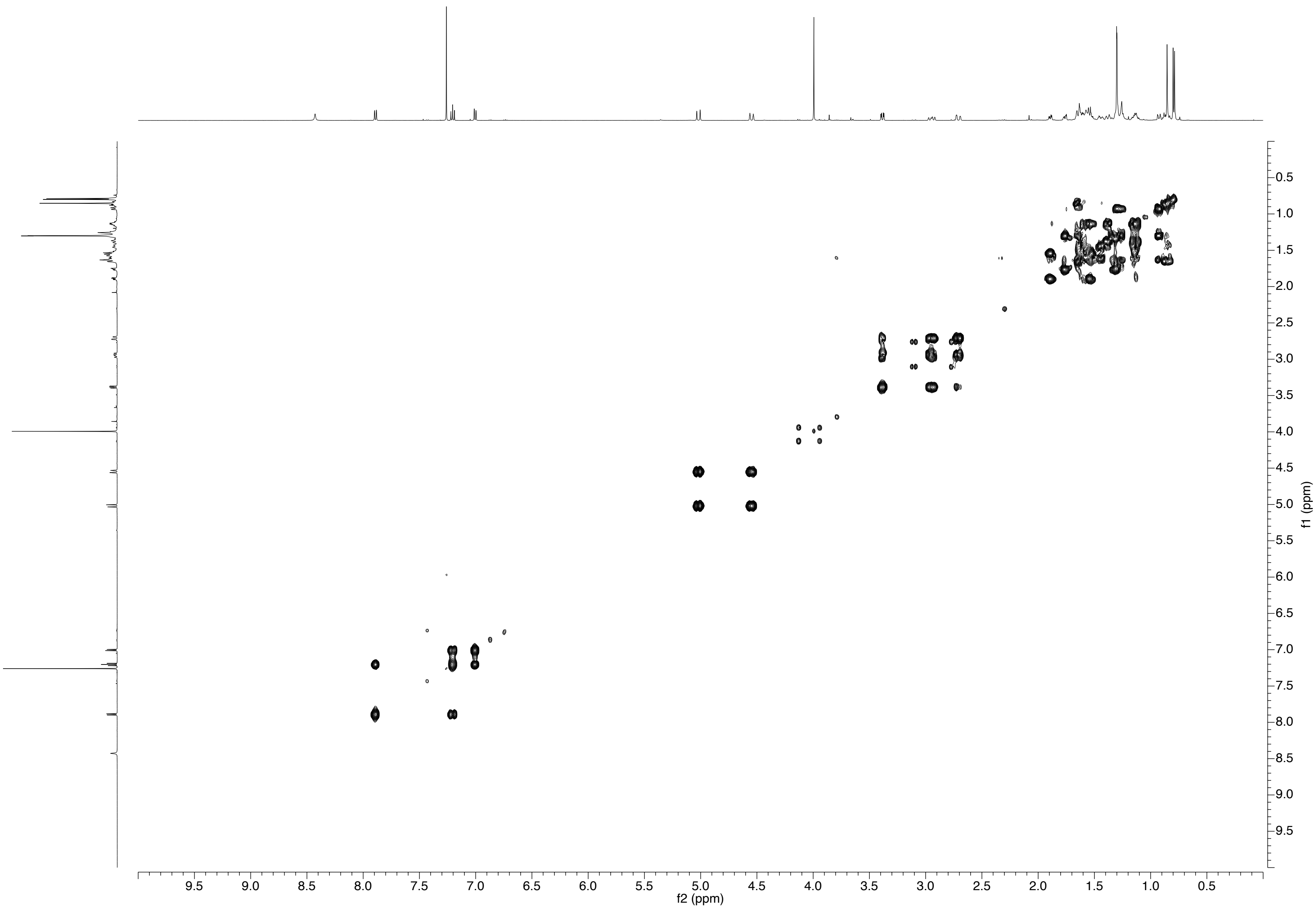

gCOSY spectrum of zigralone F (**6**) (298K, $CDCl_3$)

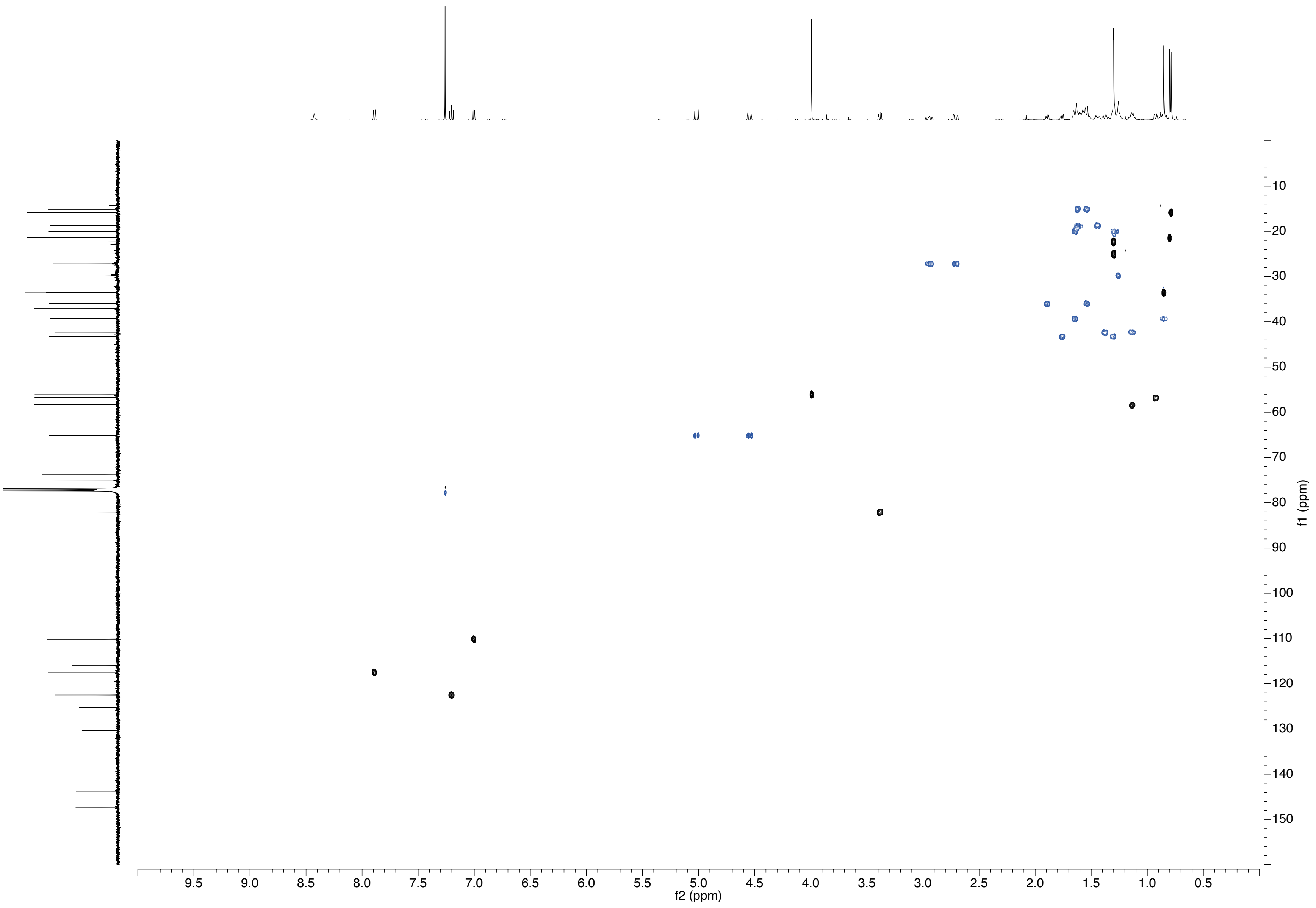

gHSQC spectrum of zigralone F (**6**) (298K, $CDCl_3$)

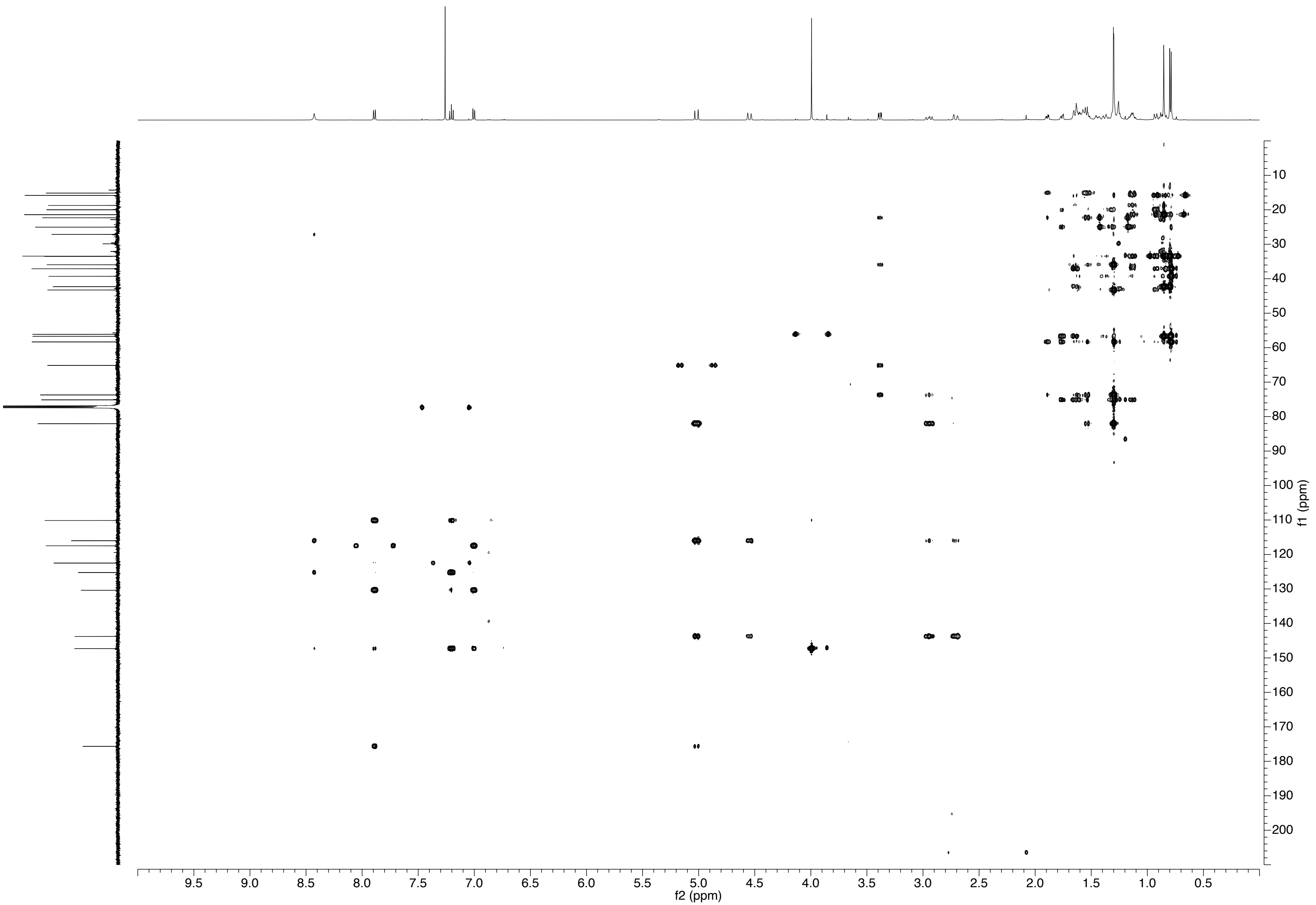

gHMBC spectrum of zigralone F (**6**) (298K, $CDCl_3$)

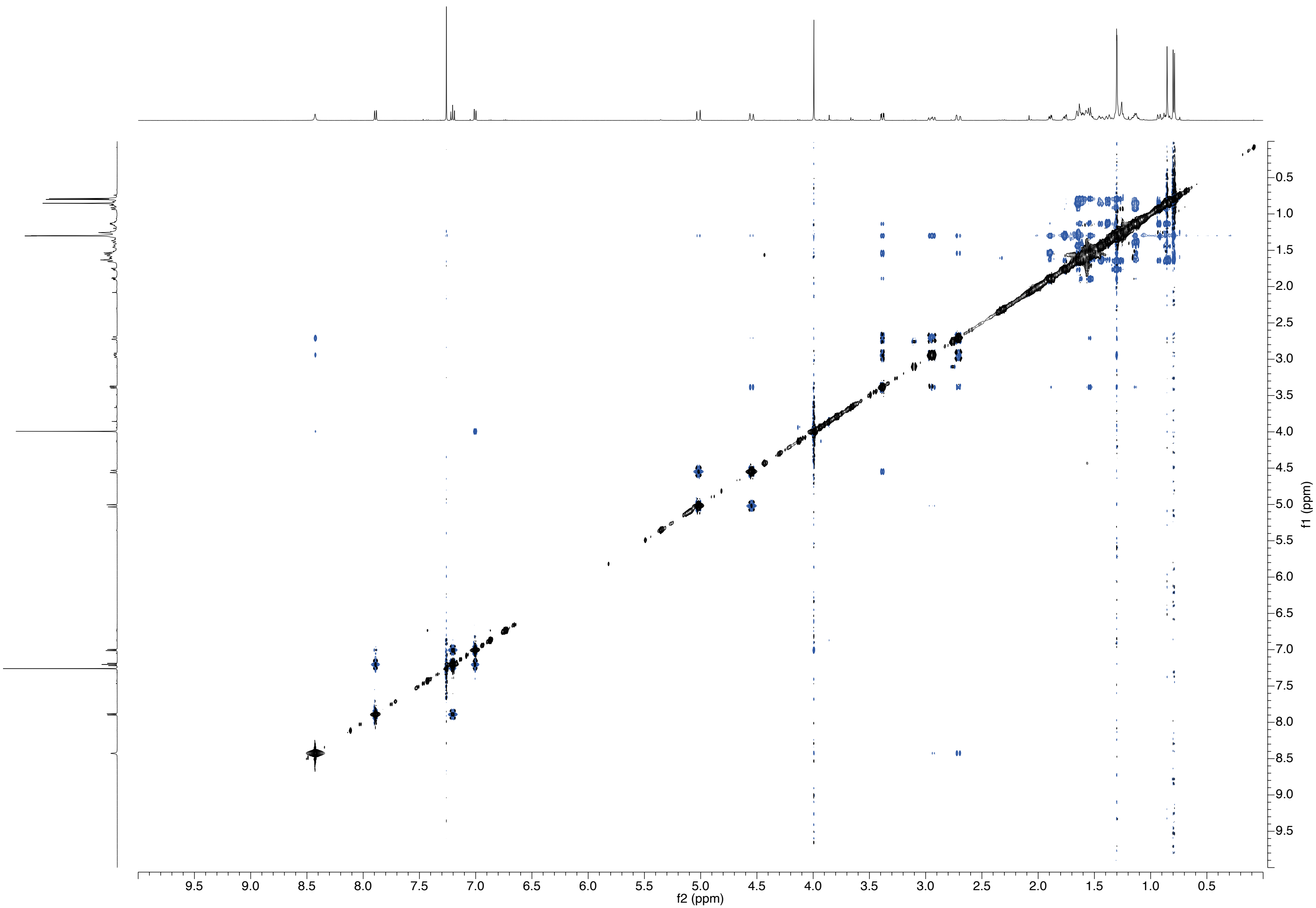

gNOESY spectrum of zigralone F (**6**) (298K, $CDCl_3$)

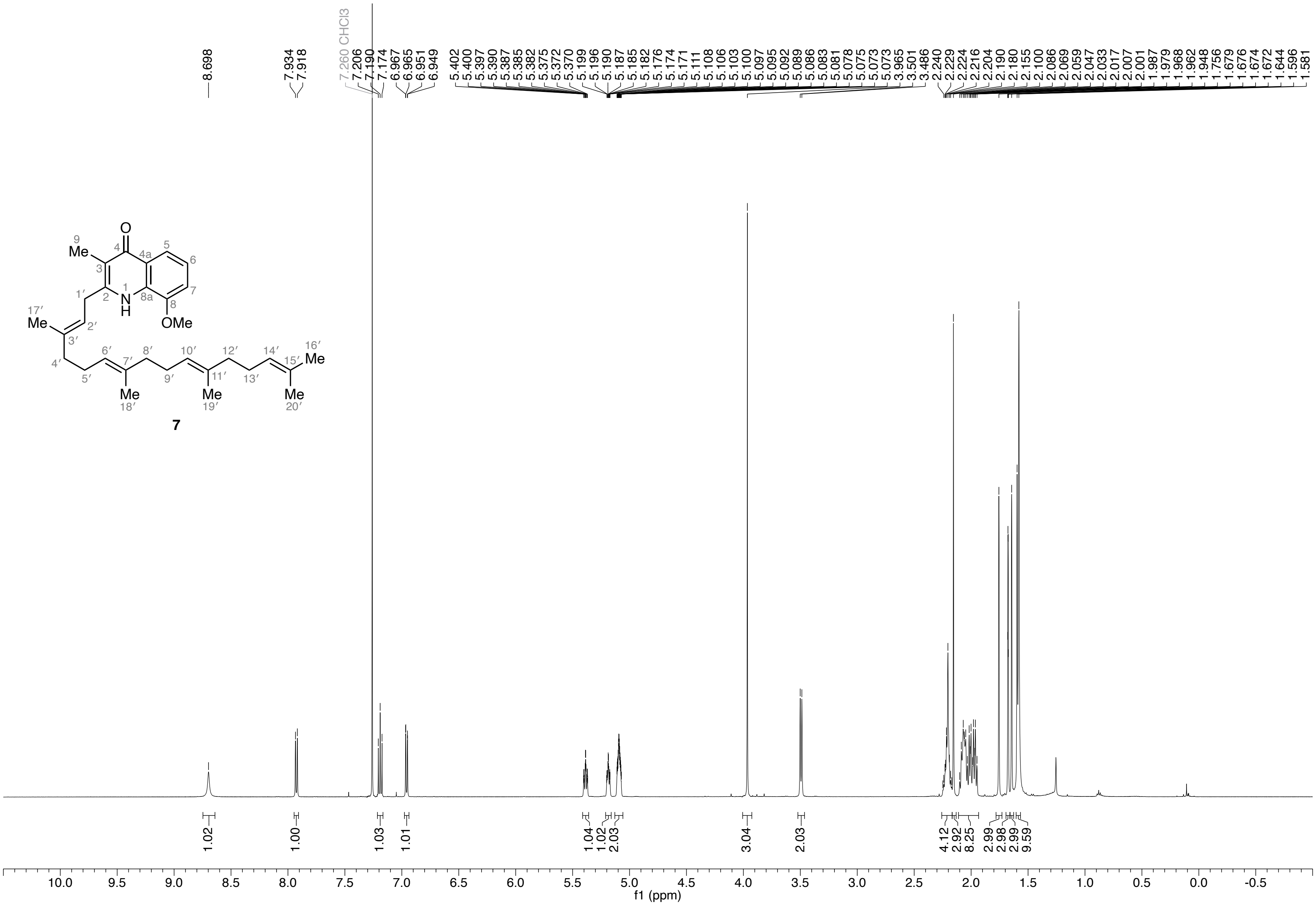

^{1}H NMR spectrum of zigralone G (**7**) (500.18 MHz, 298K, $CDCl_3$)

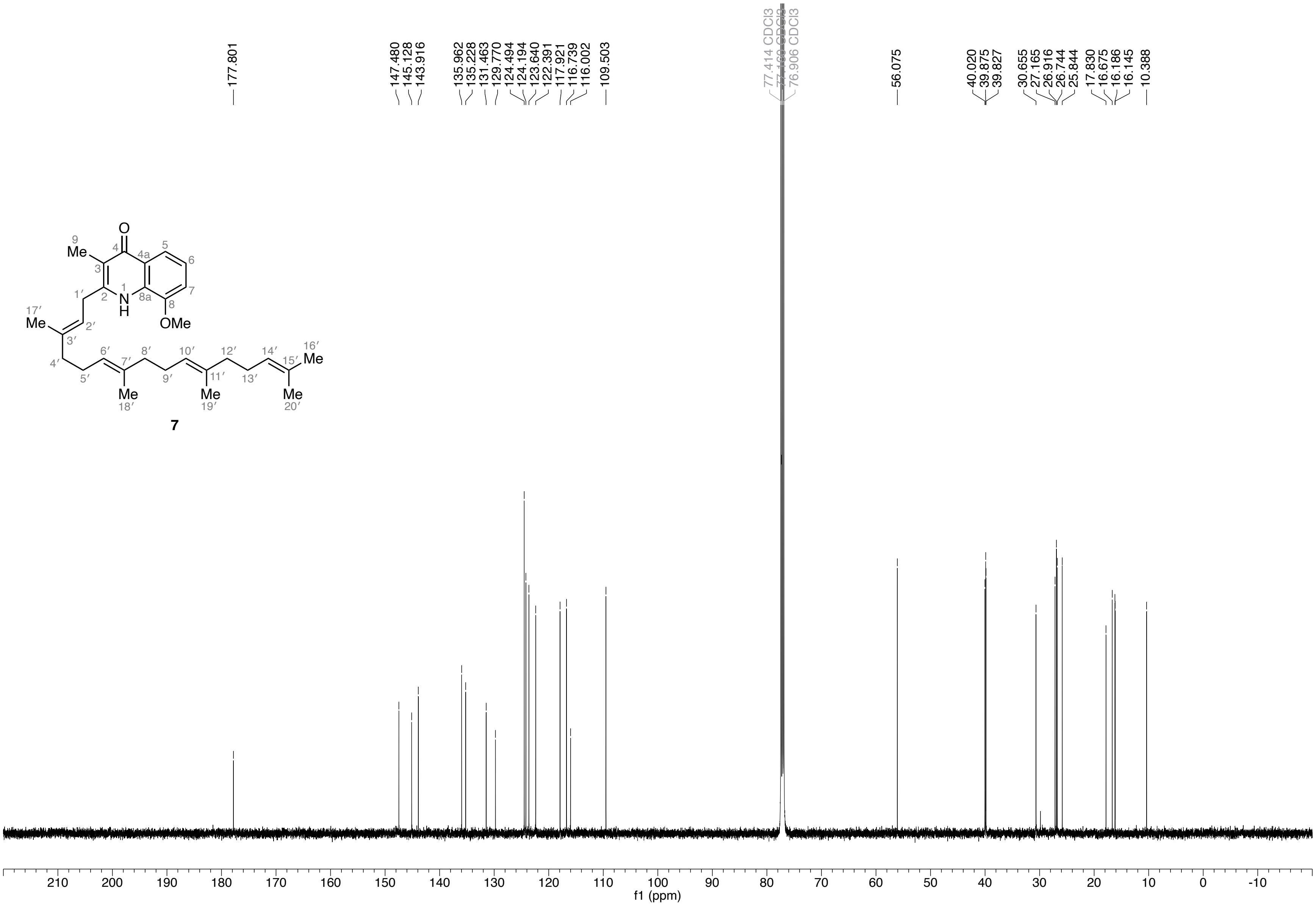

$^{13}C\{^{1}H\}$ NMR spectrum of zigralone G (**7**) (125.78 MHz, 298K, $CDCl_3$)

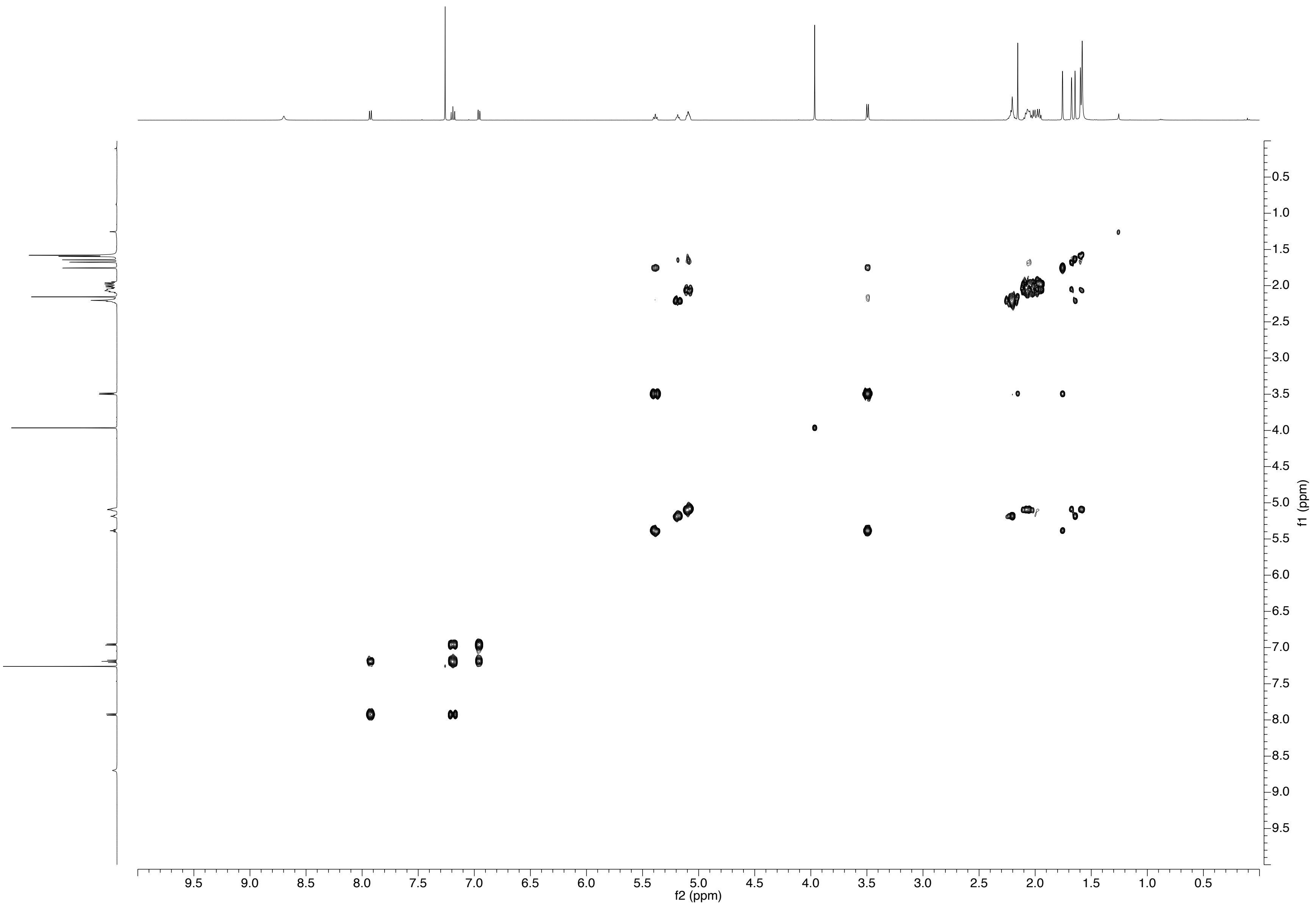

gCOSY spectrum of zigralone G (**7**) (298K, $CDCl_3$)

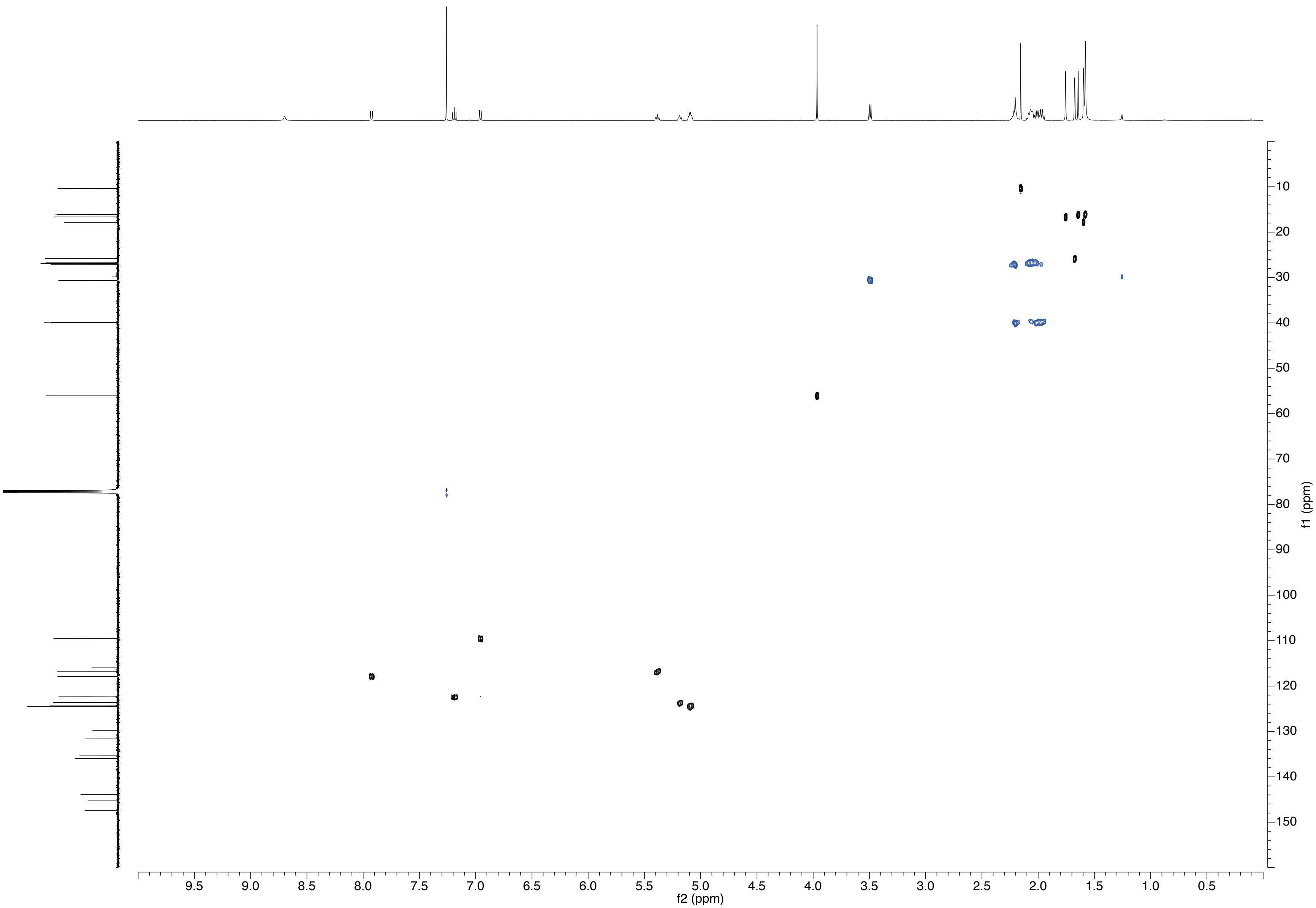

gHSQC spectrum of zigralone G (**7**) (298K, $CDCl_3$)

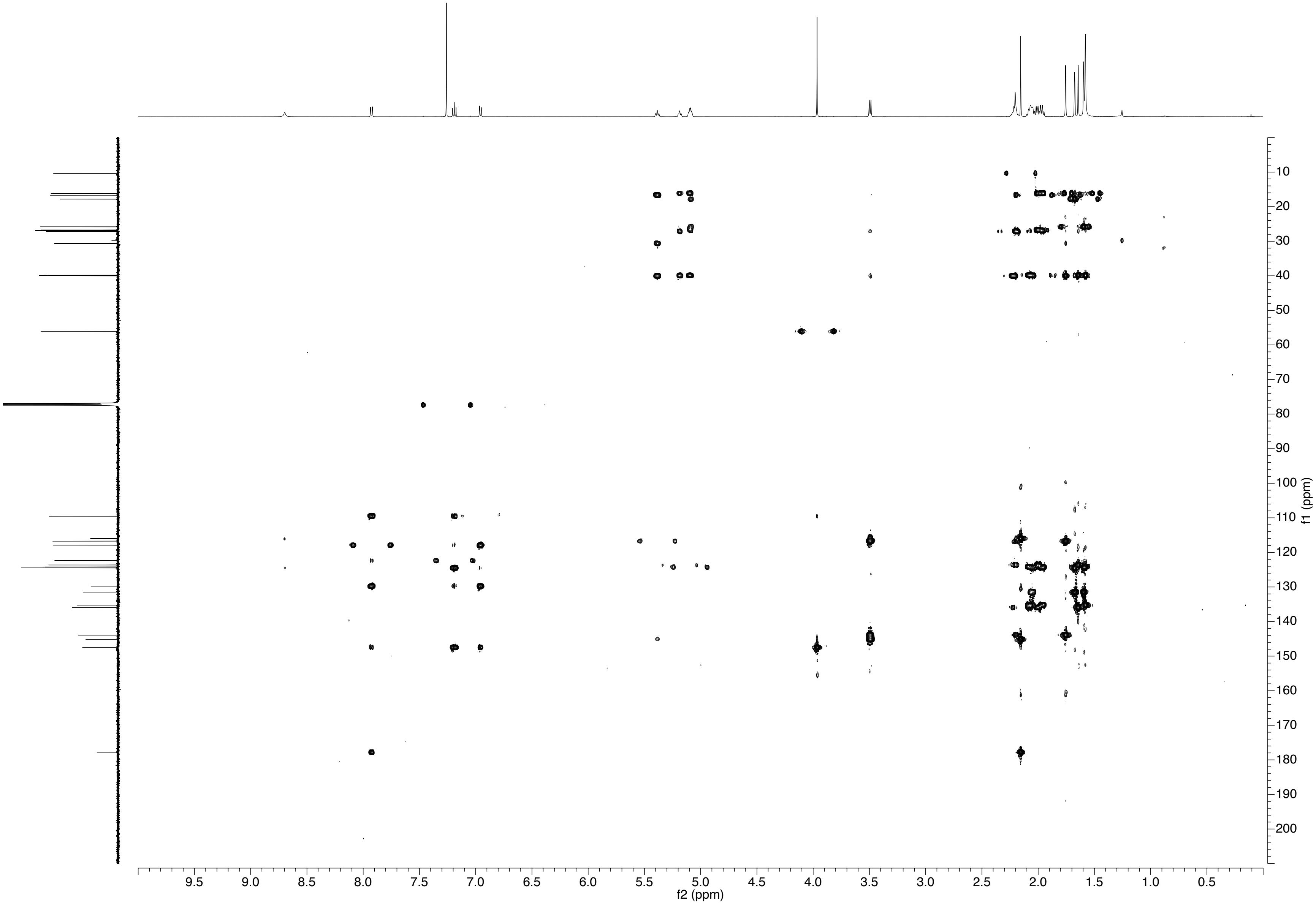

gHMBC spectrum of zigralone G (**7**) (298K, $CDCl_3$)

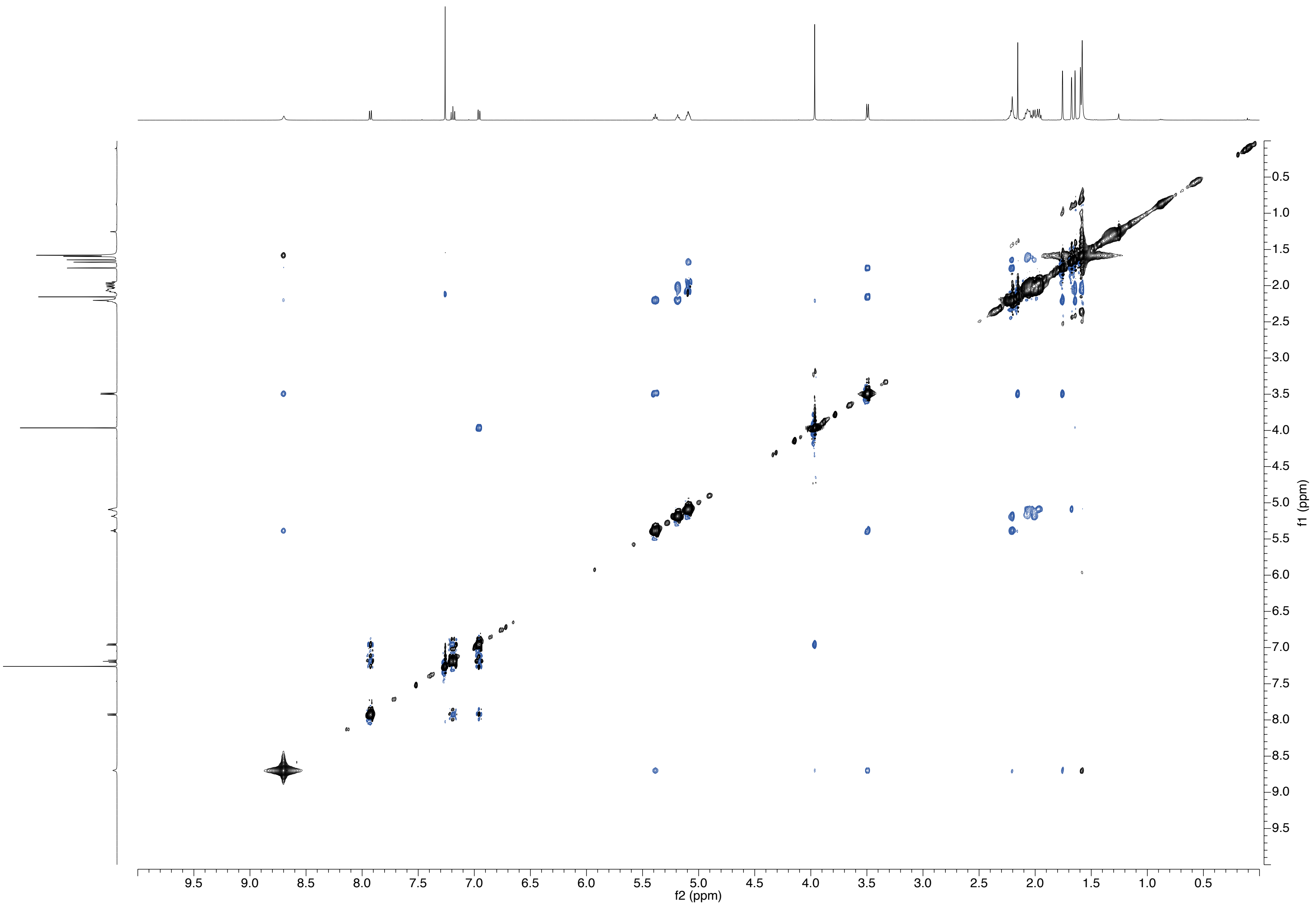

gNOESY spectrum of zigralone G (**7**) (298K, $CDCl_3$)

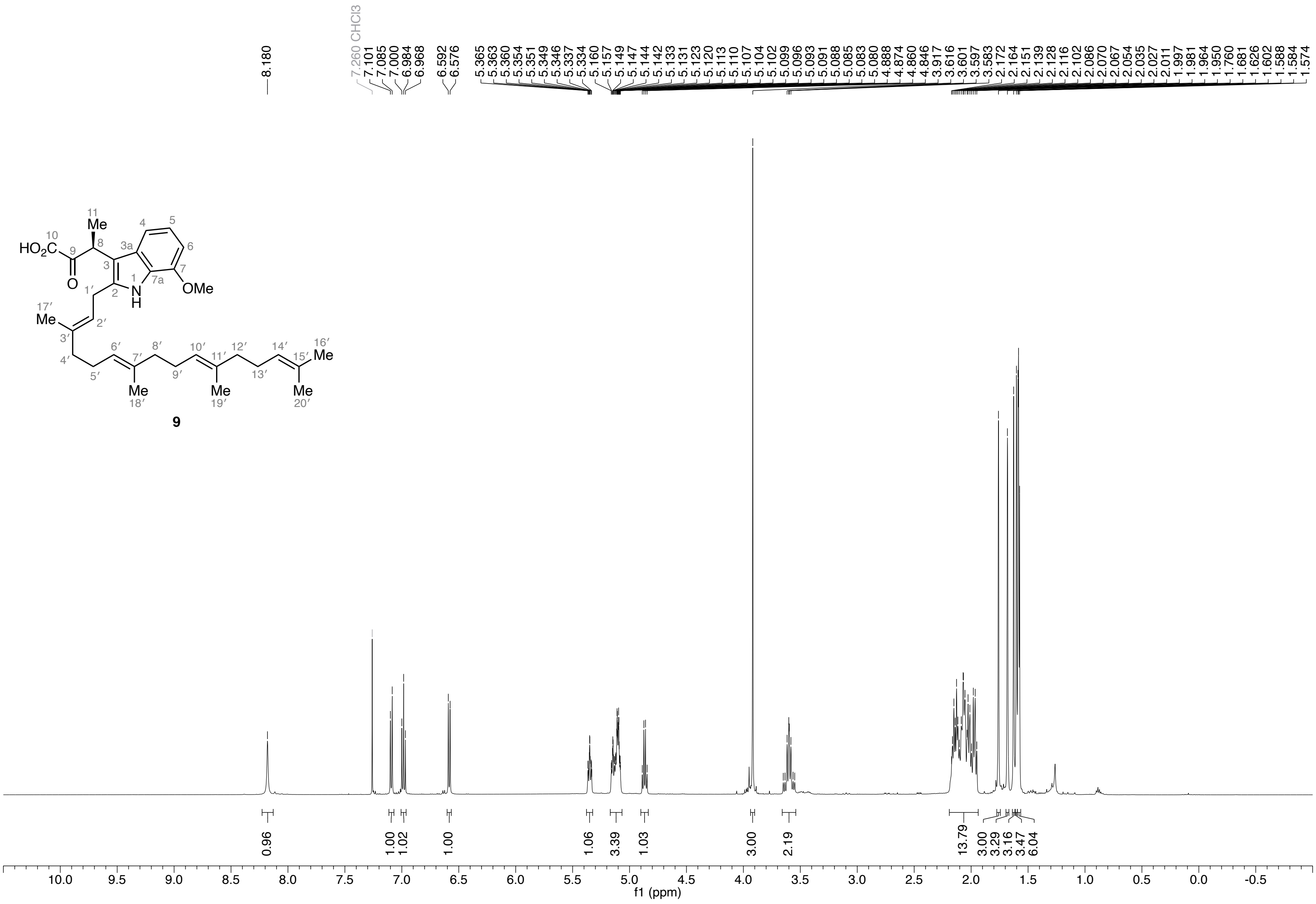

^{1}H NMR spectrum of prezigralone G (**9**) (500.18 MHz, 298K, $CDCl_3$)

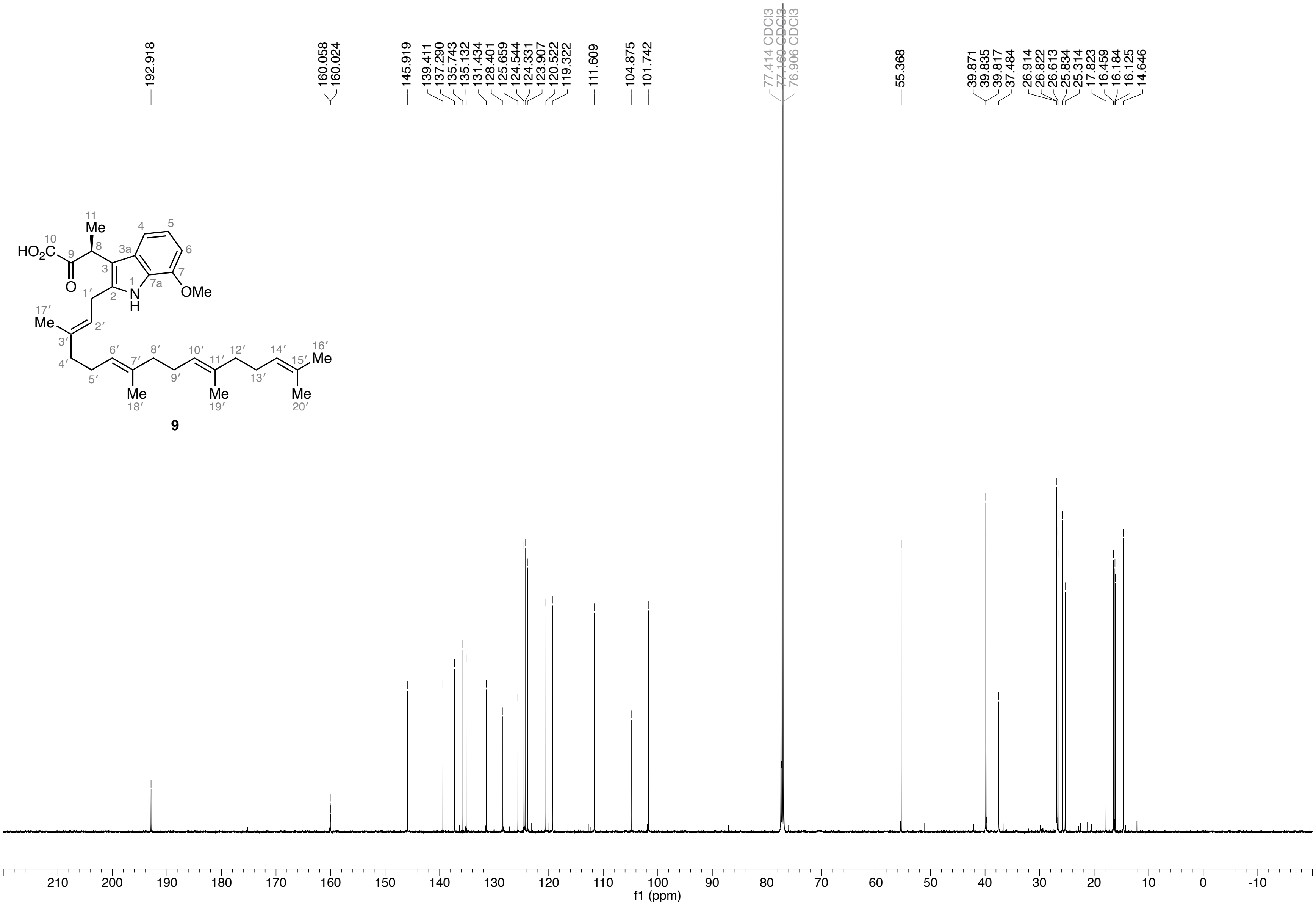

$^{13}C\{^{1}H\}$ NMR spectrum of prezigralone G (**9**) (125.78 MHz, 298K, $CDCl_3$)

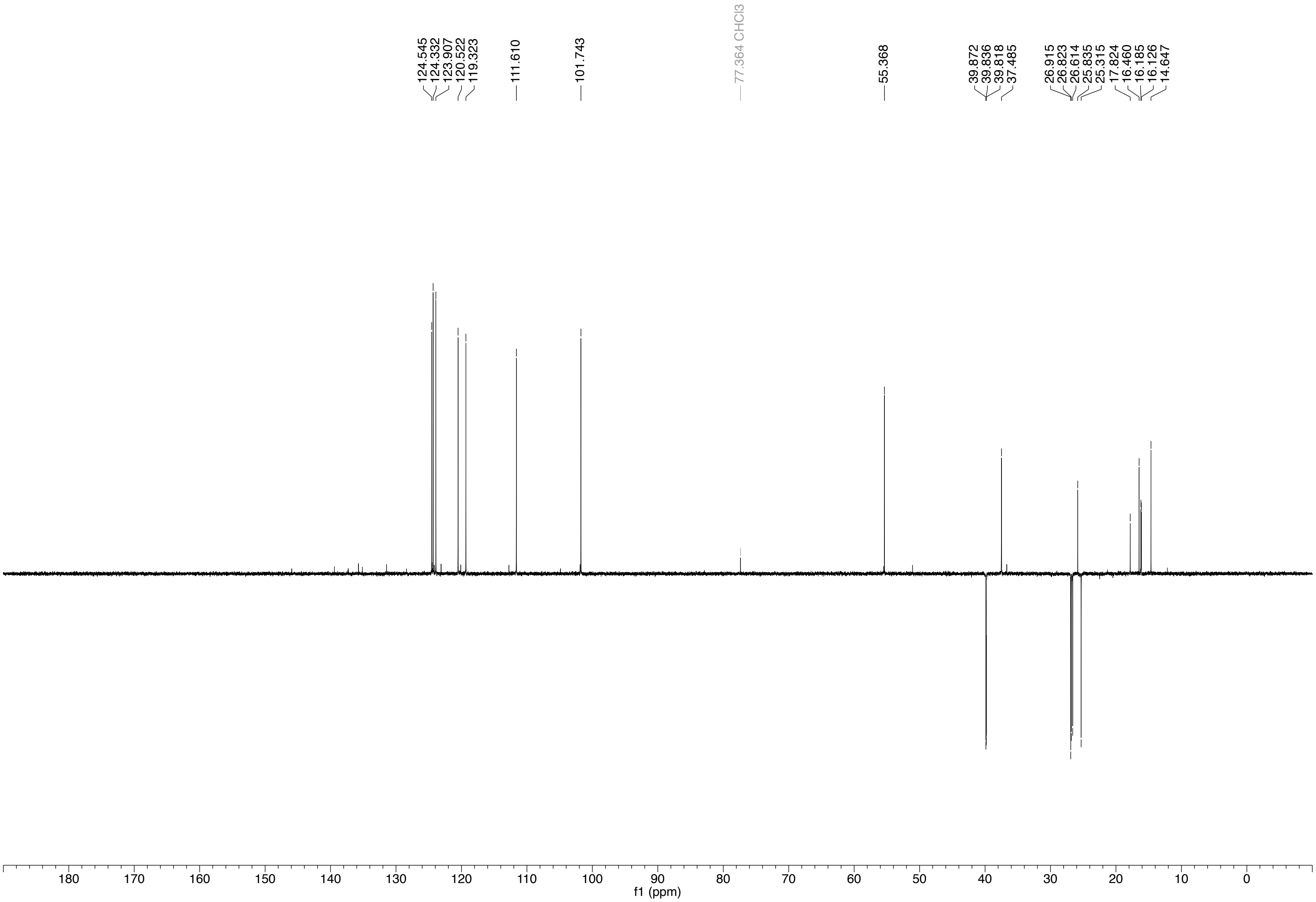

DEPT135 NMR spectrum of prezigralone G (**9**) (125.78 MHz, 298K, $CDCl_3$)

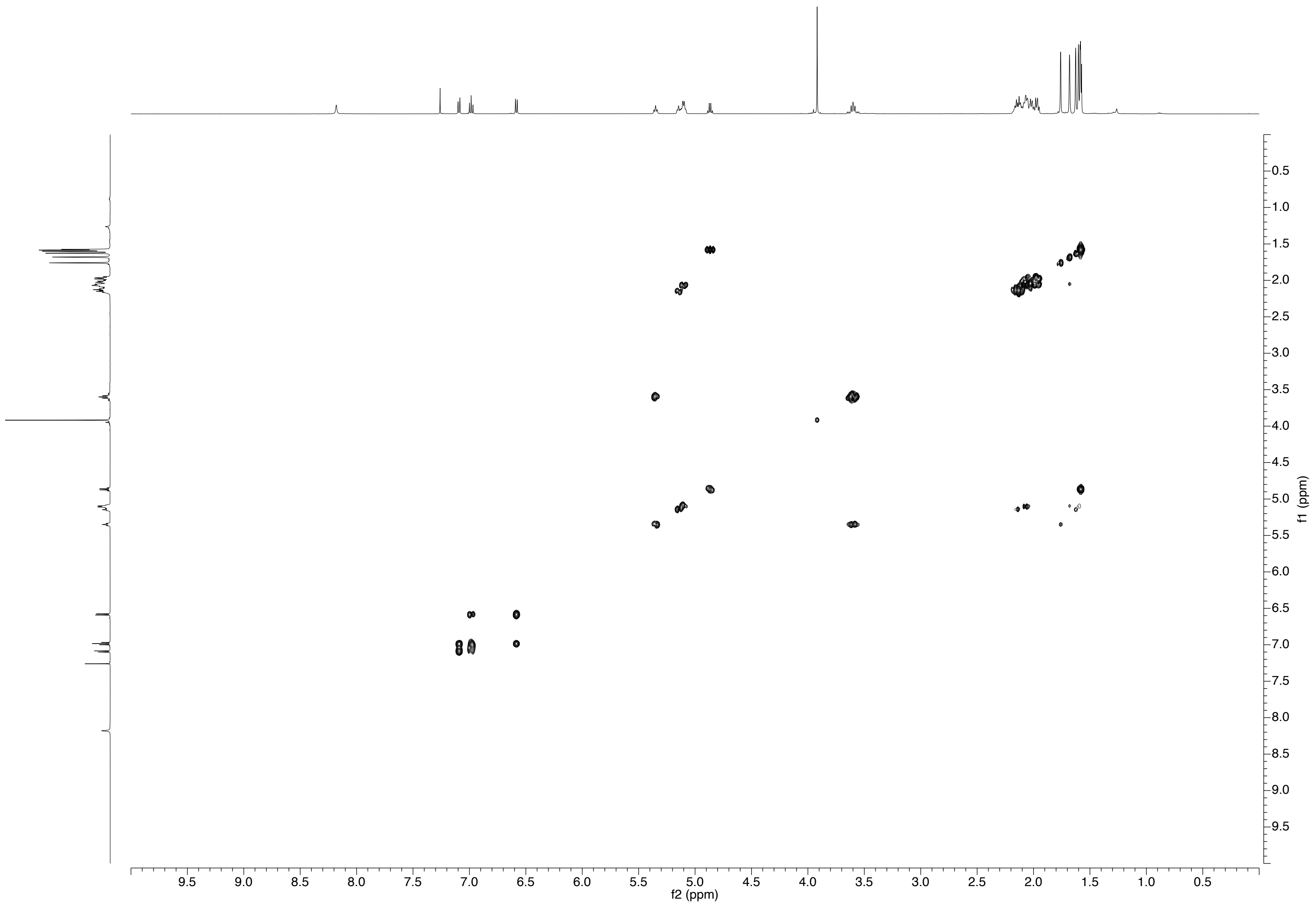

gCOSY spectrum of prezigralone G (**9**) (298K, $CDCl_3$)

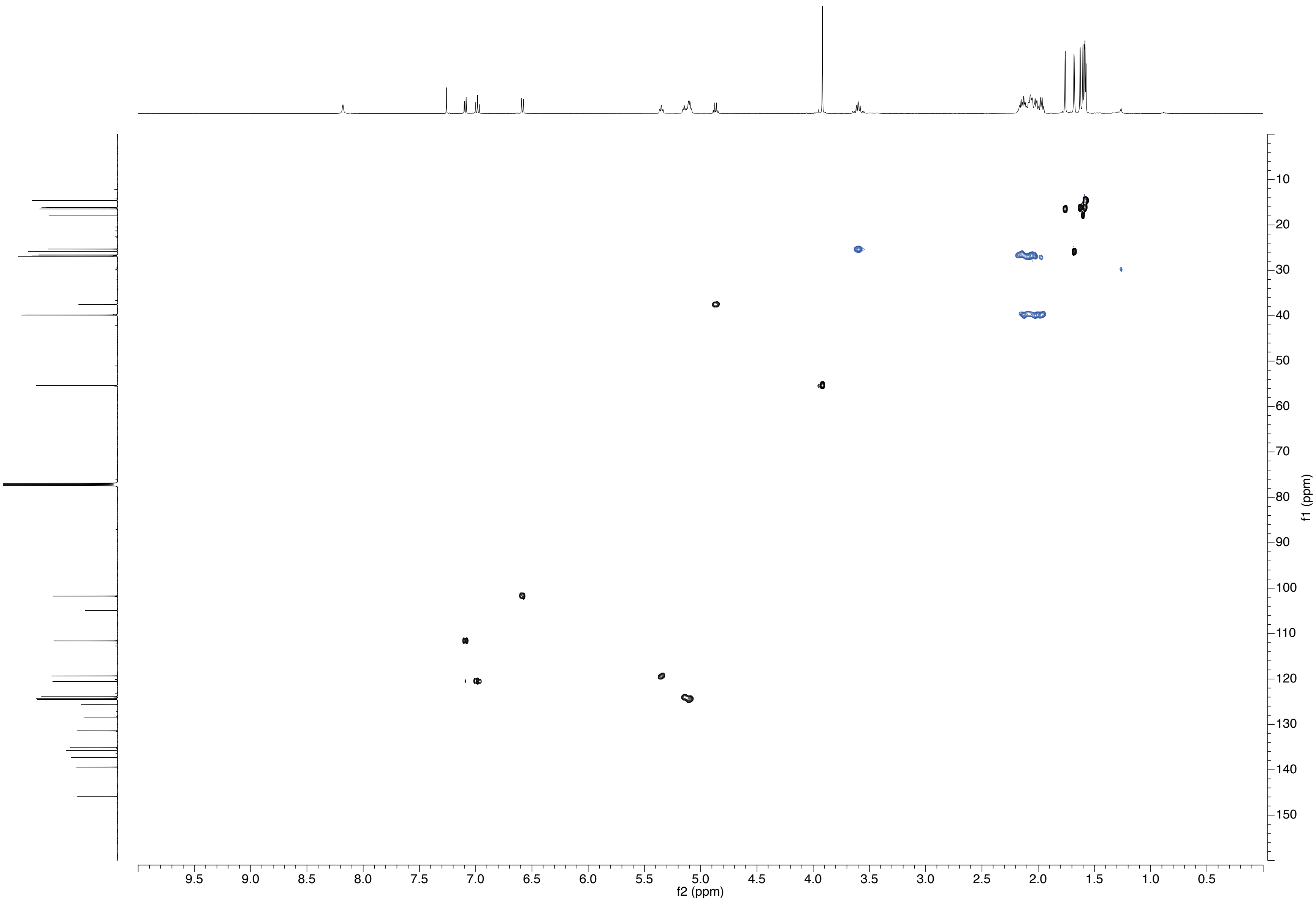

gHSQC spectrum of prezigralone G (**9**) (298K, $CDCl_3$)

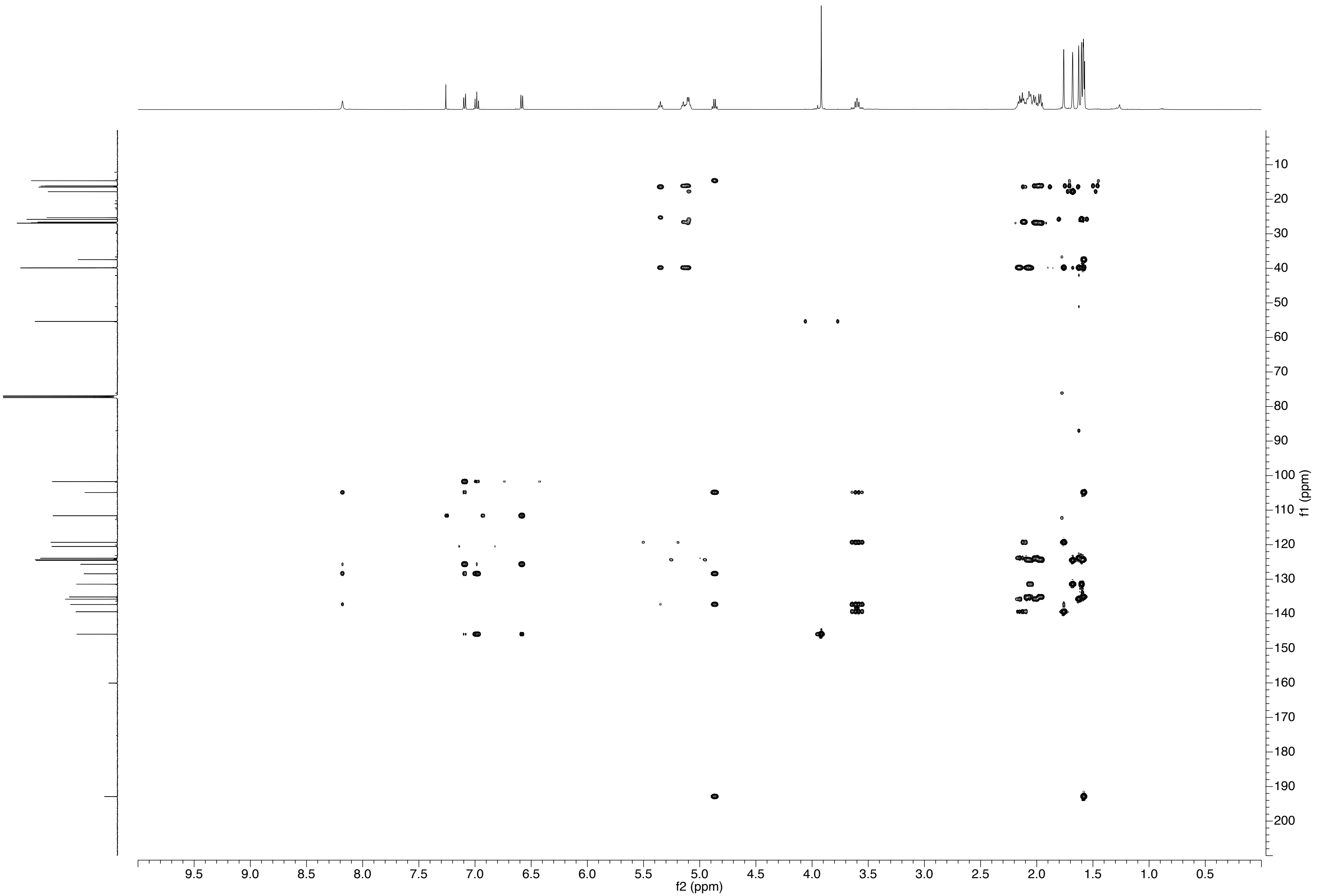

gHMBC spectrum of prezigralone G (**9**) (298K, $CDCl_3$)

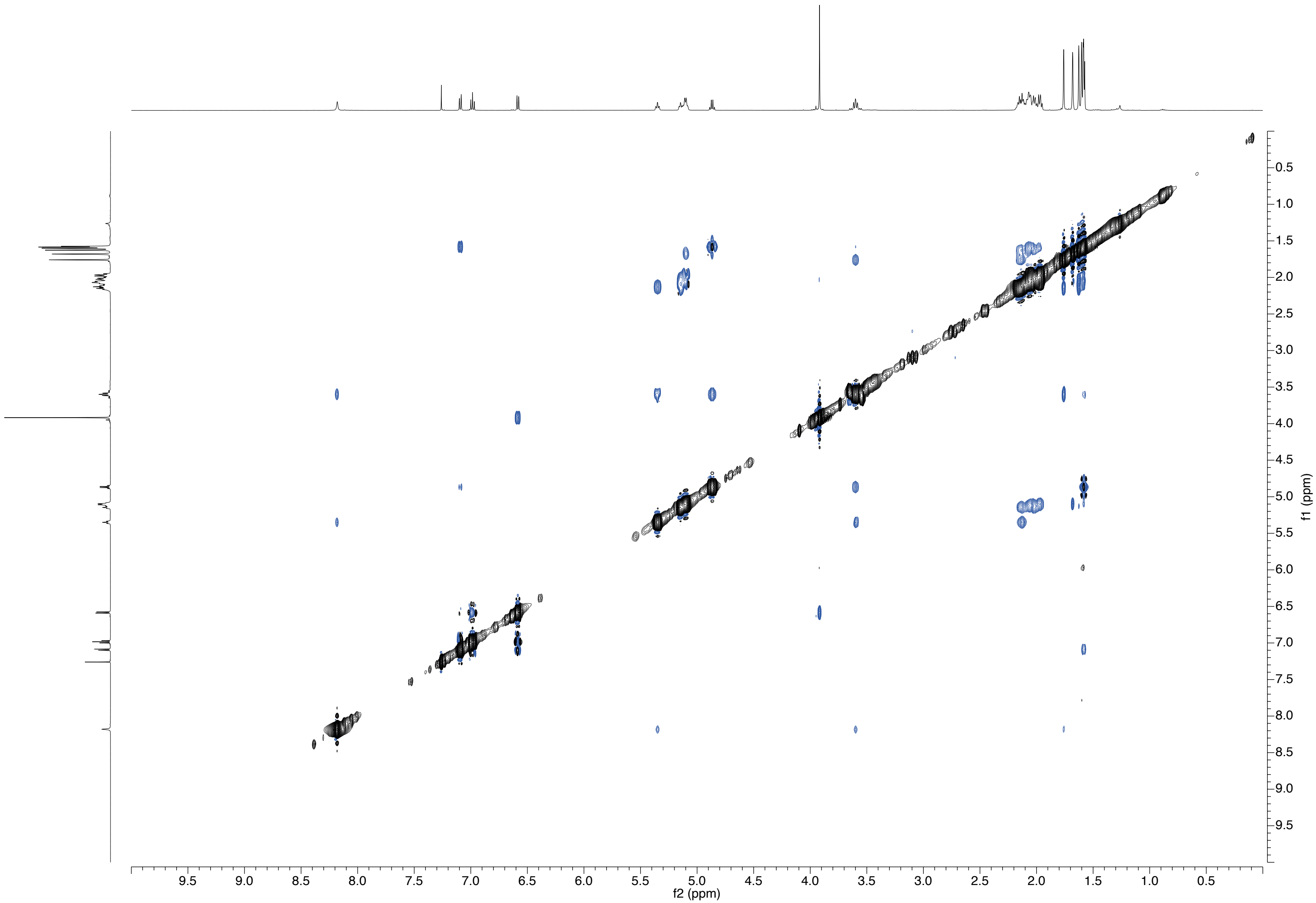

gNOESY spectrum of prezigralone G (**9**) (298K, $CDCl_3$)

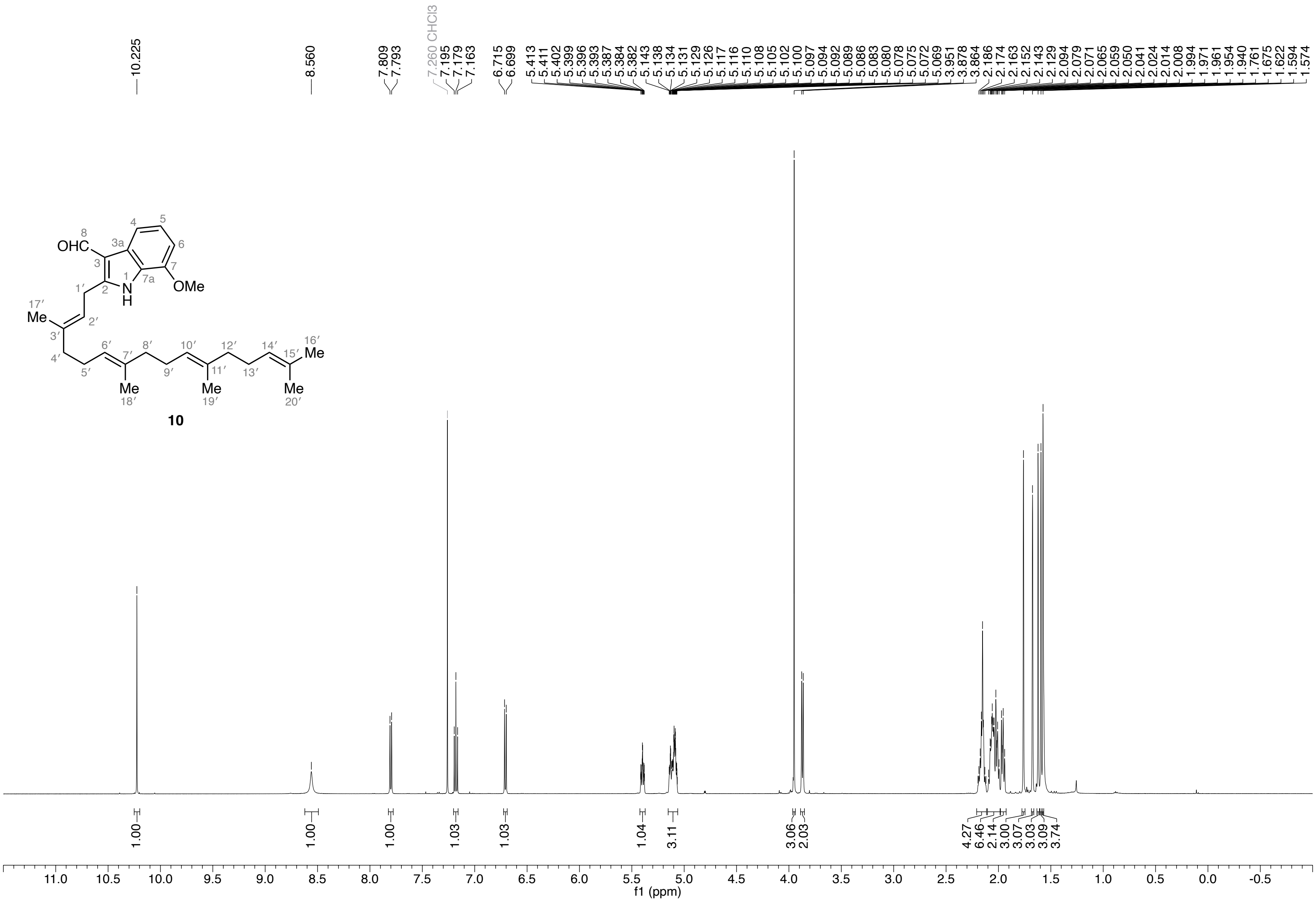

^{1}H NMR spectrum of 2-geranylgeranyl-7-methoxy-1*H*-indole-3-carboxaldehyde (**10**) (500.18 MHz, 298K, $CDCl_3$)

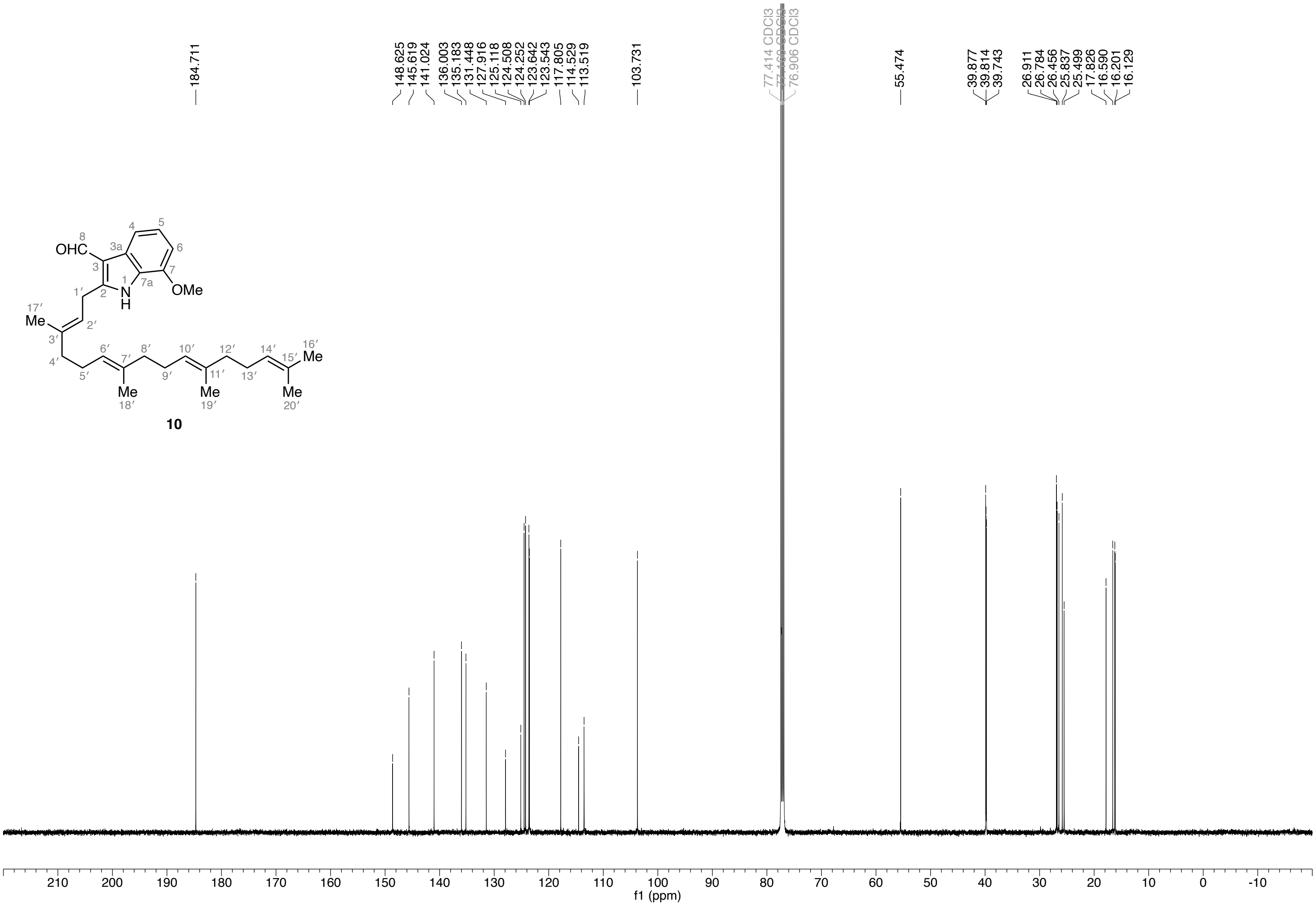

$^{13}C\{^{1}H\}$ NMR spectrum of 2-geranylgeranyl-7-methoxy-1*H*-indole-3-carboxaldehyde (**10**) (125.78 MHz, 298K, $CDCl_3$)

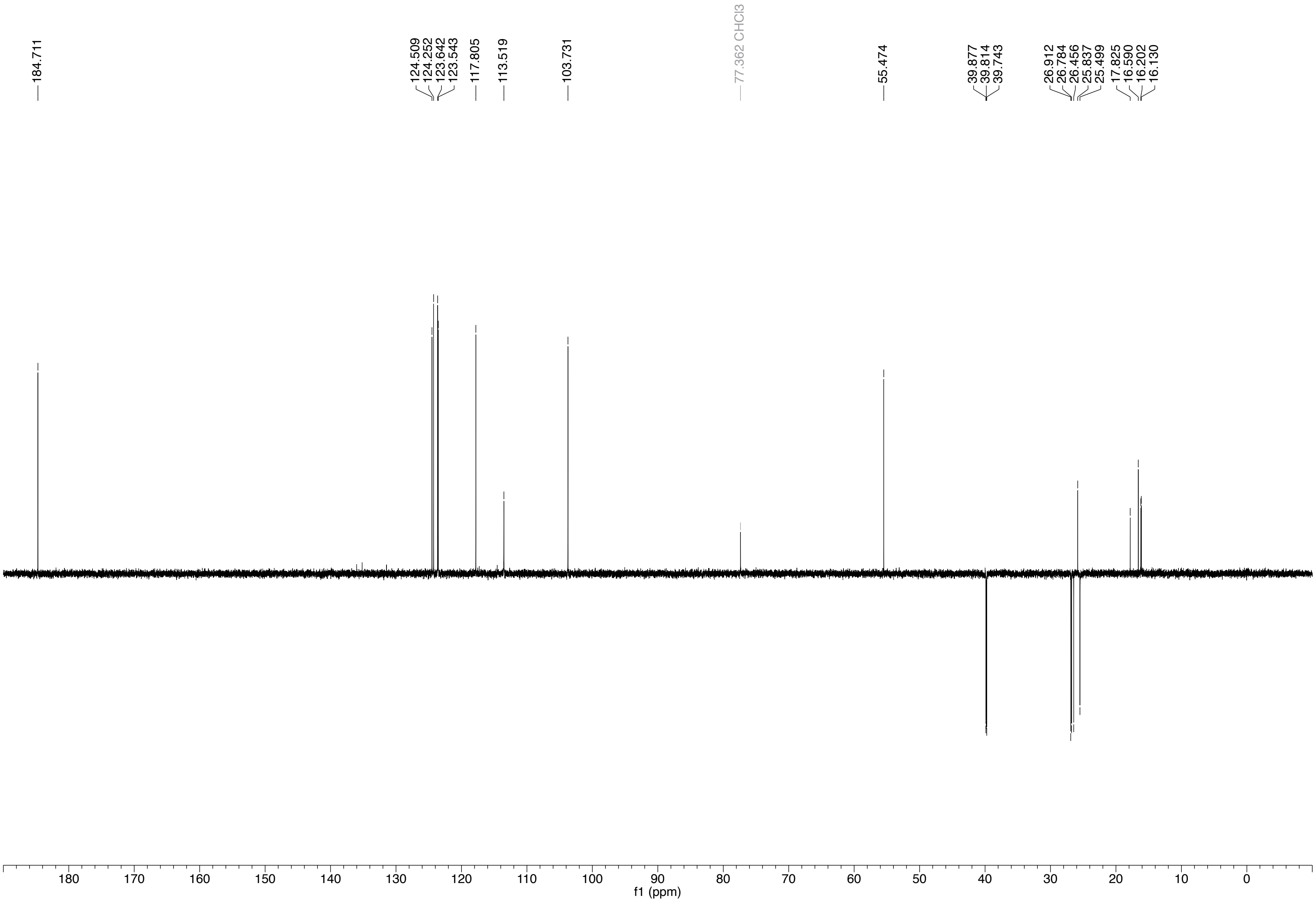

DEPT135 NMR spectrum of 2-geranylgeranyl-7-methoxy-1*H*-indole-3-carboxaldehyde (**10**) (125.78 MHz, 298K, $CDCl_3$)

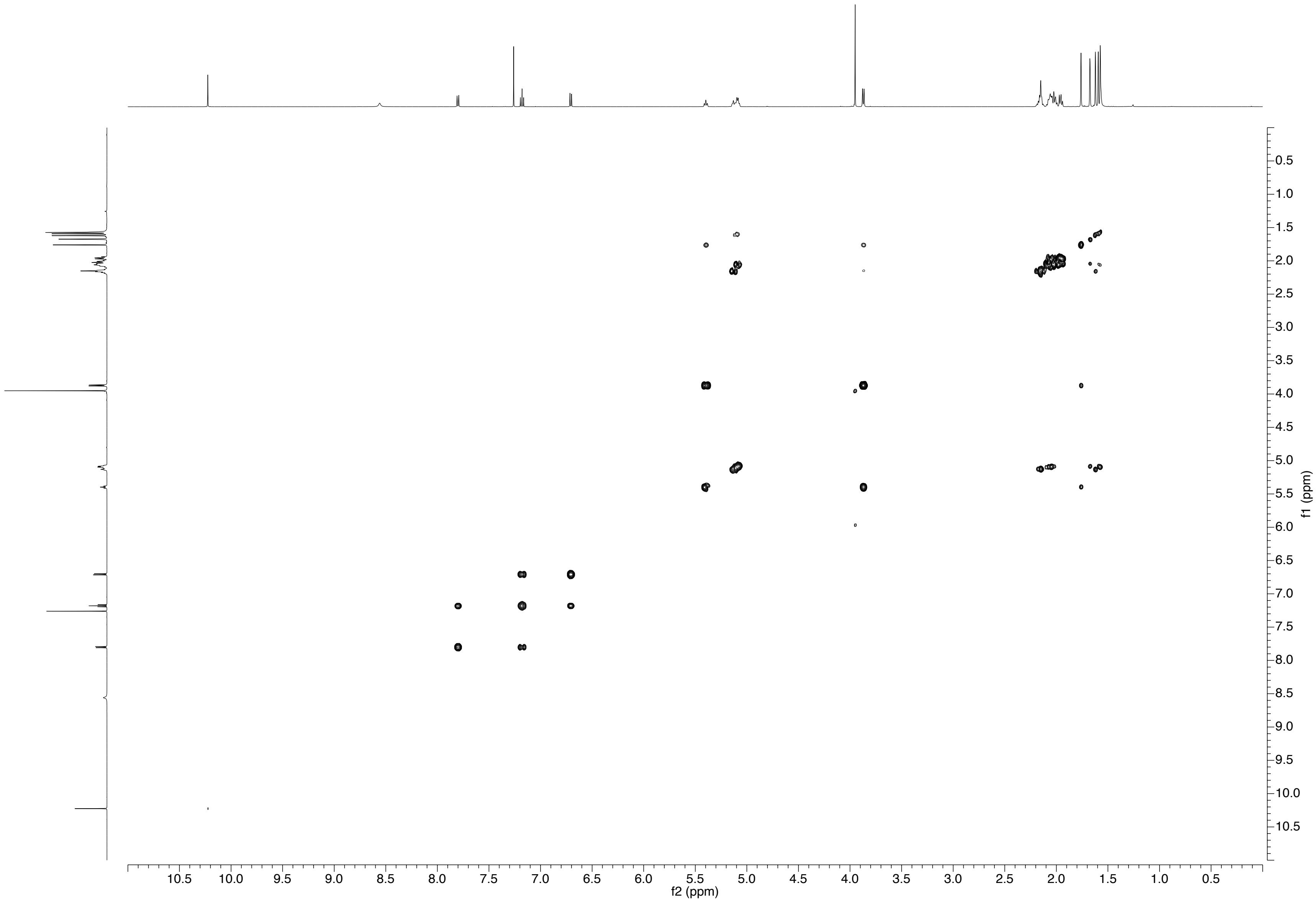

gCOSY spectrum of 2-geranylgeranyl-7-methoxy-1*H*-indole-3-carboxaldehyde (**10**) (298K, $CDCl_3$)

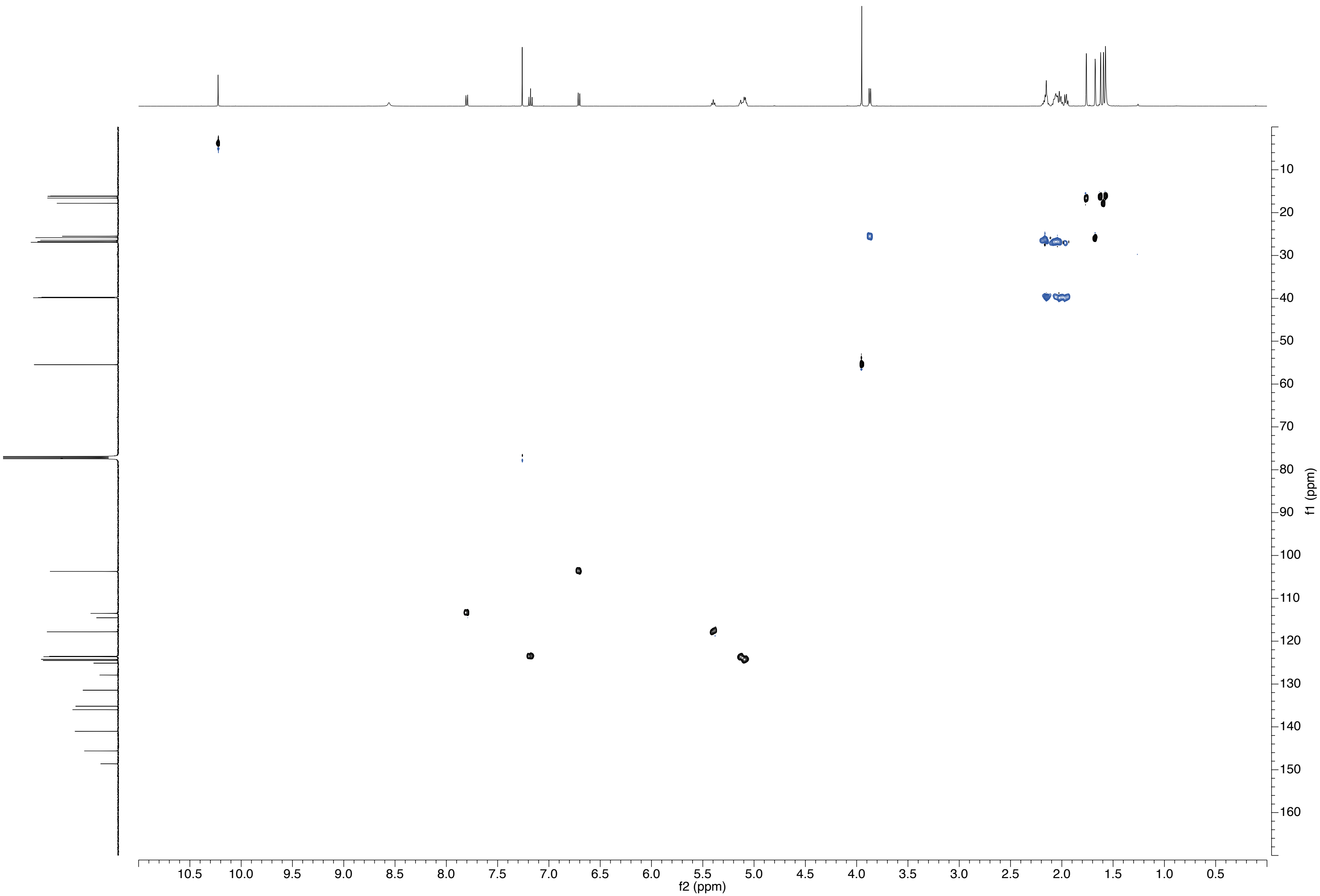

gHSQC spectrum of 2-geranylgeranyl-7-methoxy-1*H*-indole-3-carboxaldehyde (**10**) (298K, $CDCl_3$)

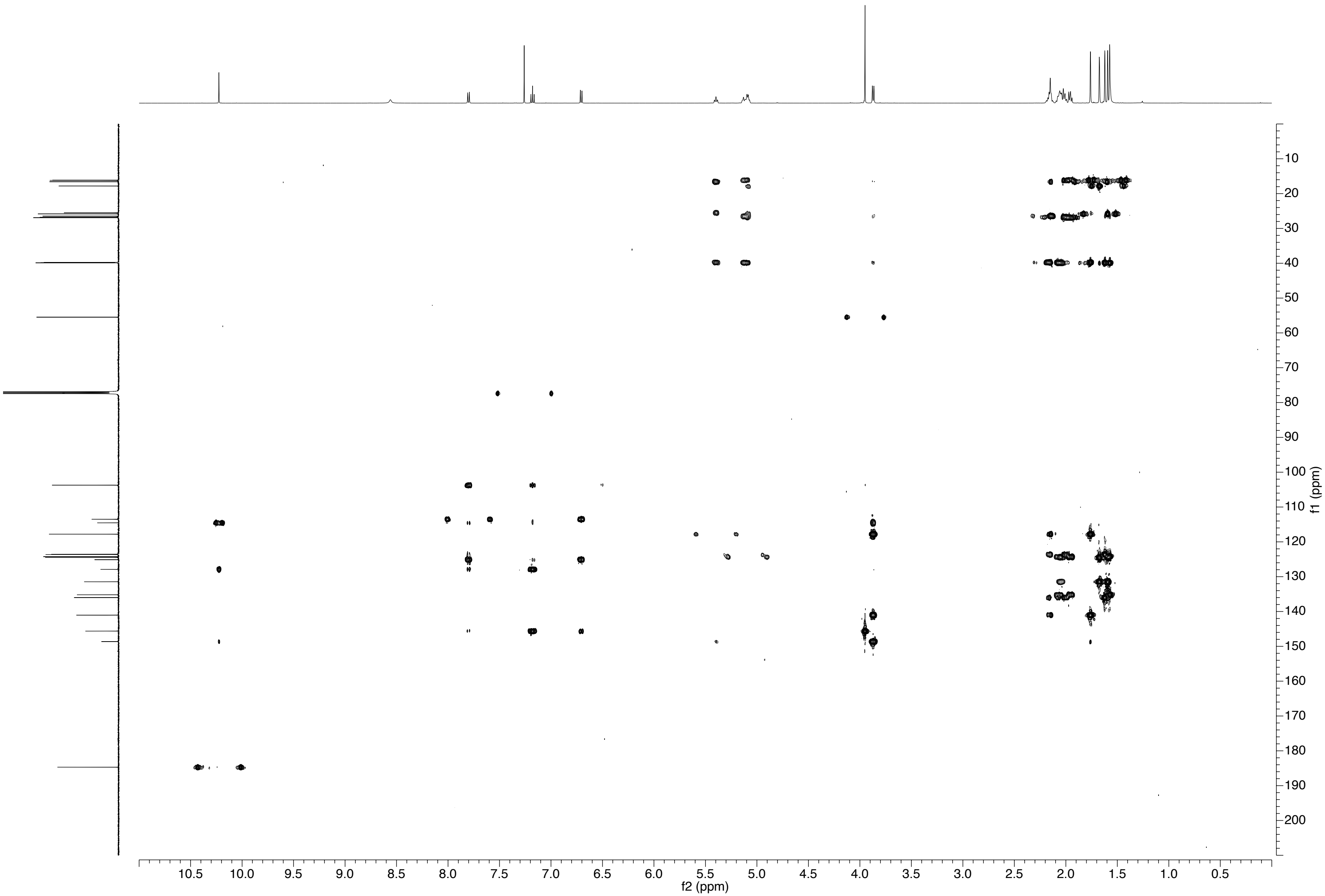

gHMBC spectrum of 2-geranylgeranyl-7-methoxy-1*H*-indole-3-carboxaldehyde (**10**) (298K, $CDCl_3$)

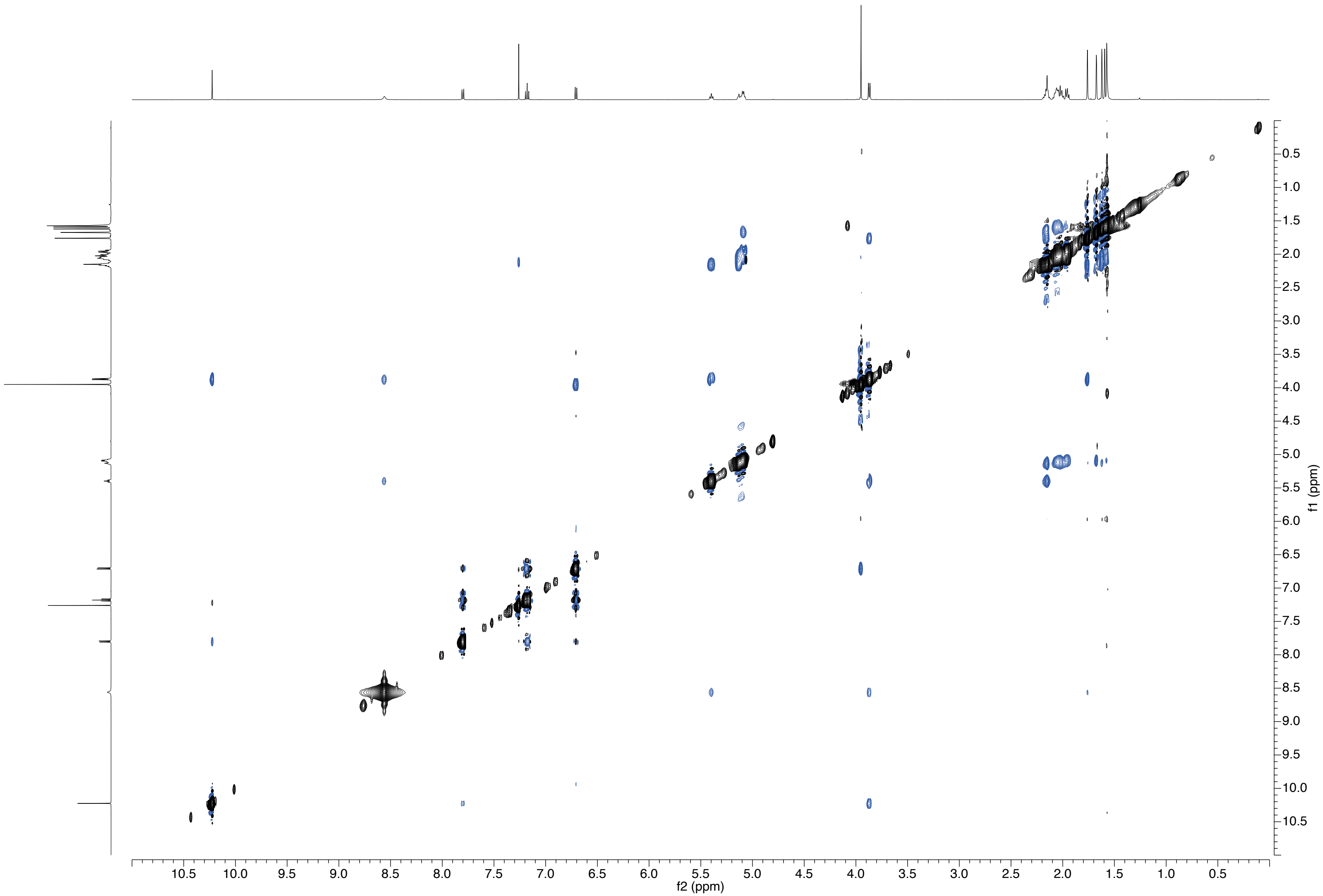

gNOESY spectrum of 2-geranylgeranyl-7-methoxy-1*H*-indole-3-carboxaldehyde (**10**) (298K, $CDCl_3$)

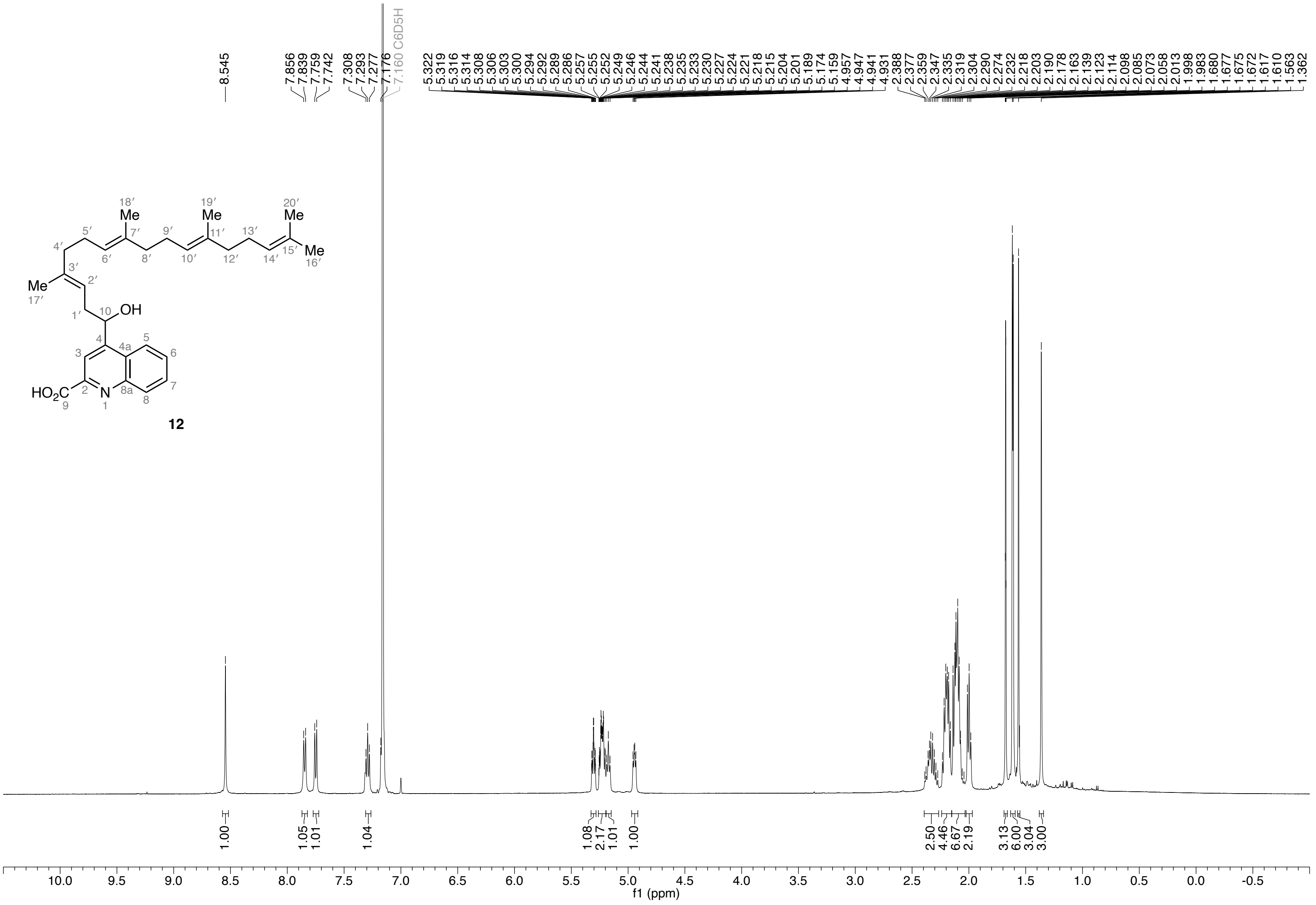

^{1}H NMR spectrum of 4-(geranylgeranyl(hydroxy)methyl)quinaldic acid (**12**) (500.18 MHz, 298K, C_6D_6)

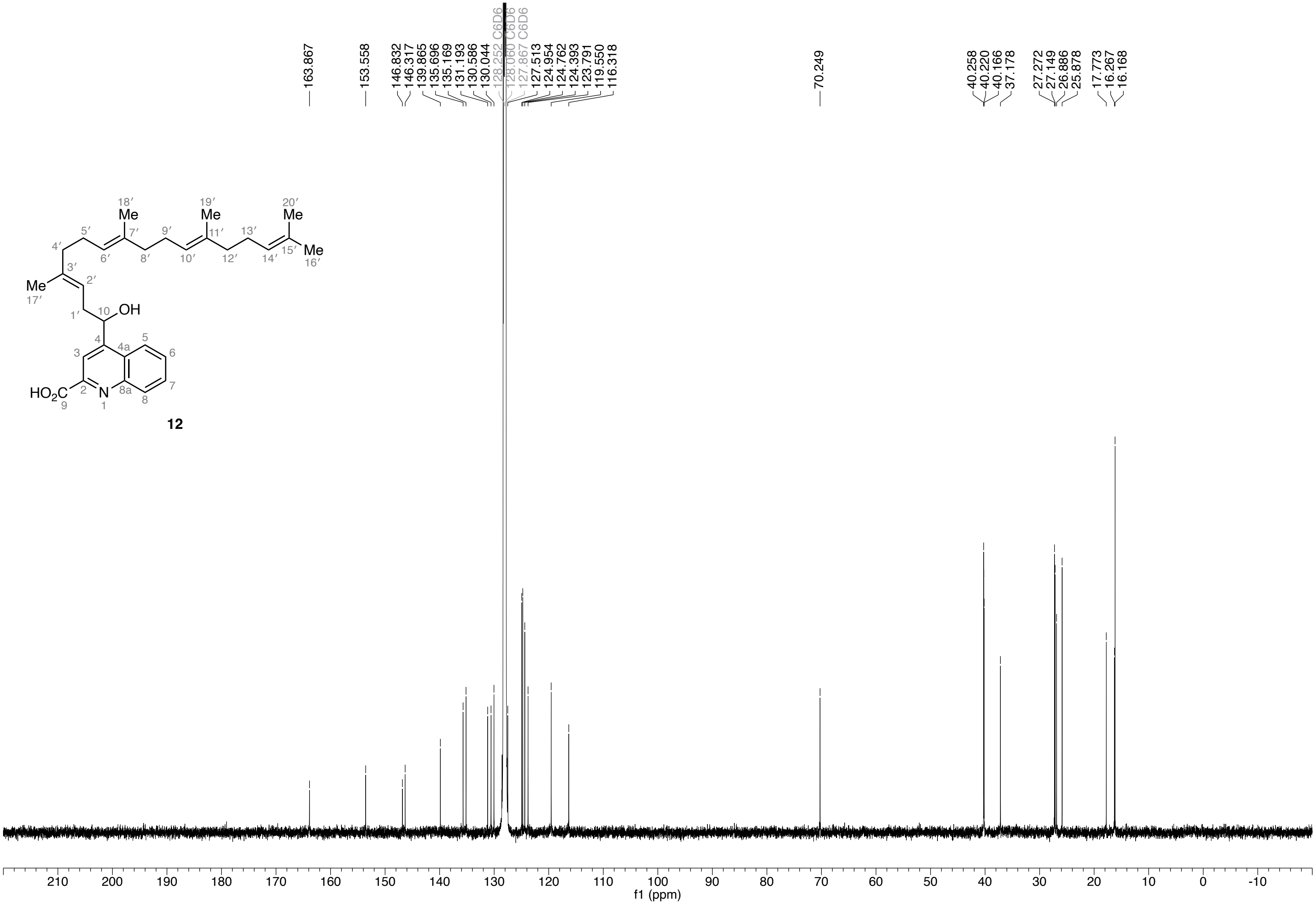

$^{13}C\{^{1}H\}$ NMR spectrum of 4-(geranylgeranyl(hydroxy)methyl)quinaldic acid (**12**) (125.78 MHz, 298K, C_6D_6)

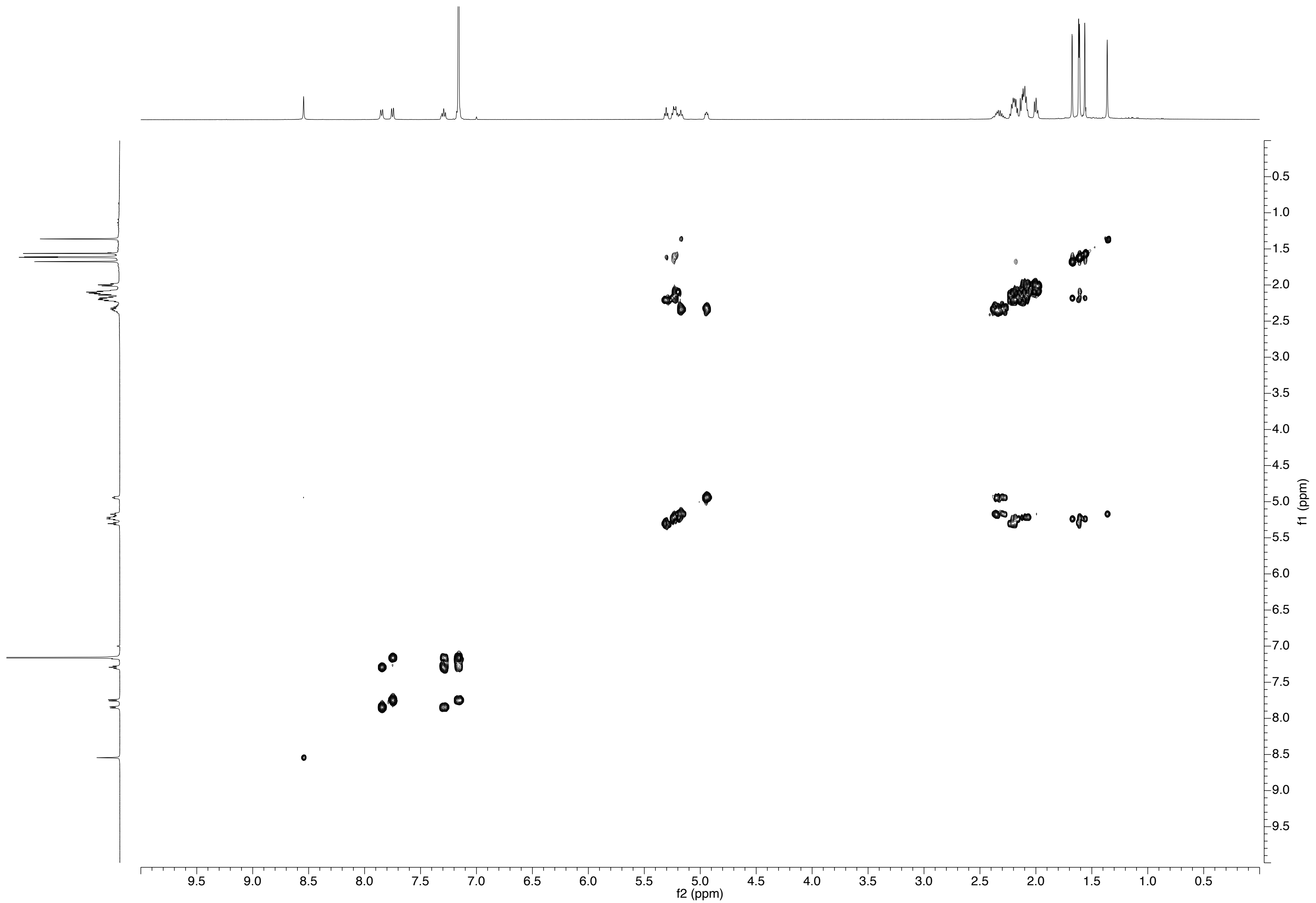

gCOSY spectrum of 4-(geranylgeranyl(hydroxy)methyl)quinaldic acid (**12**) (298K, C_6D_6)

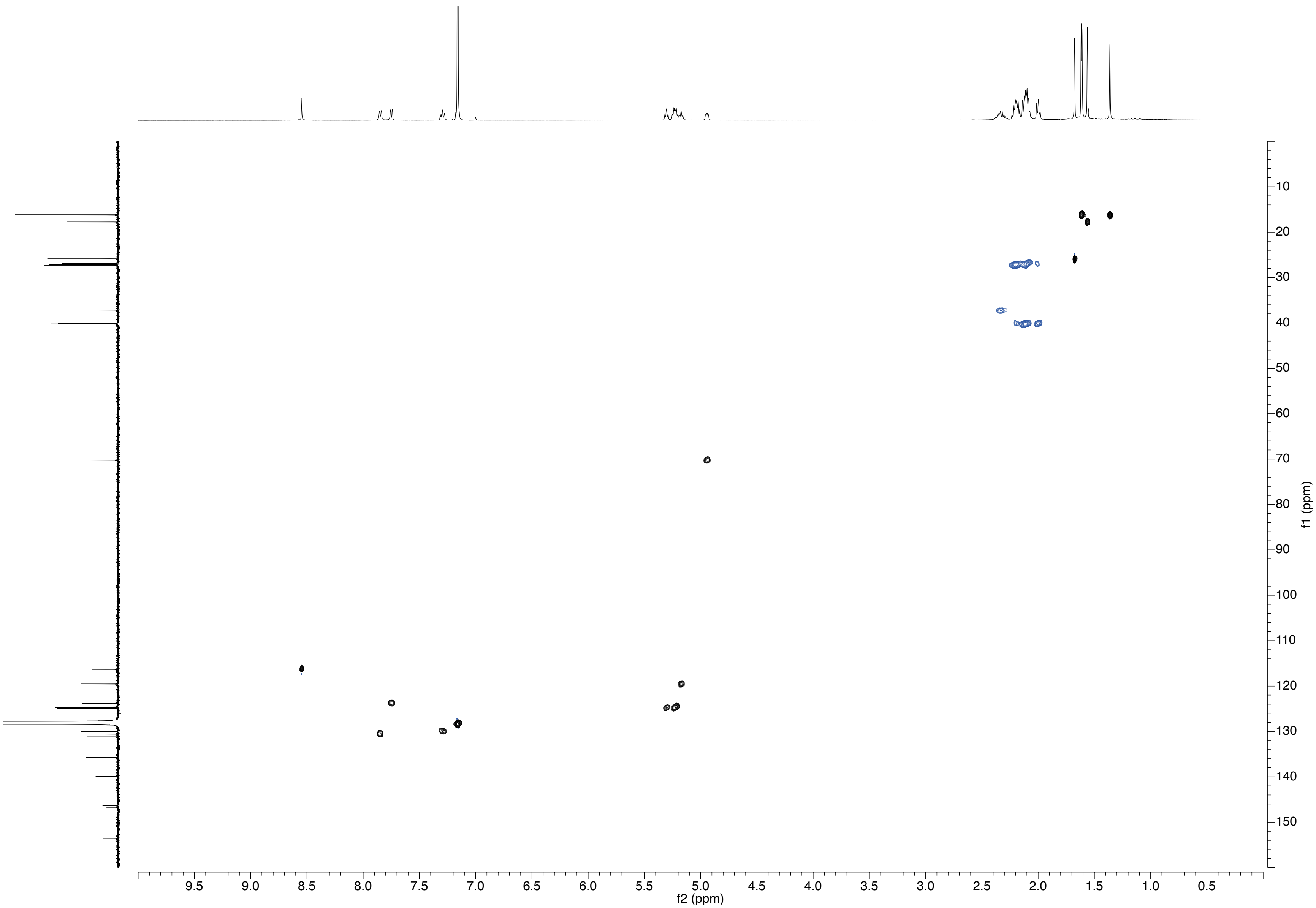

gHSQC spectrum of 4-(geranylgeranyl(hydroxy)methyl)quinaldic acid (**12**) (298K, C_6D_6)

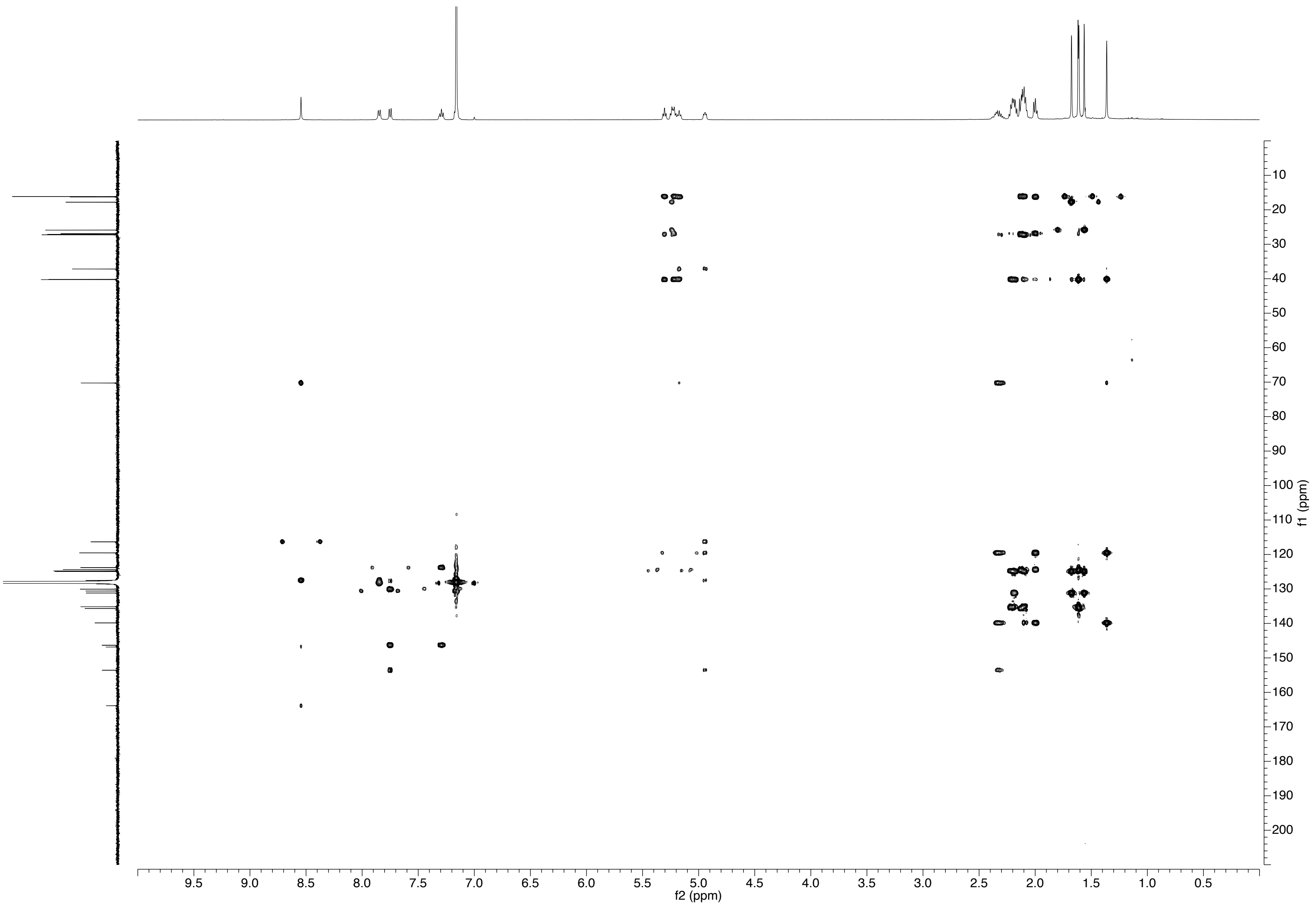

gHMBC spectrum of 4-(geranylgeranyl(hydroxy)methyl)quinaldic acid (**12**) (298K, C_6D_6)

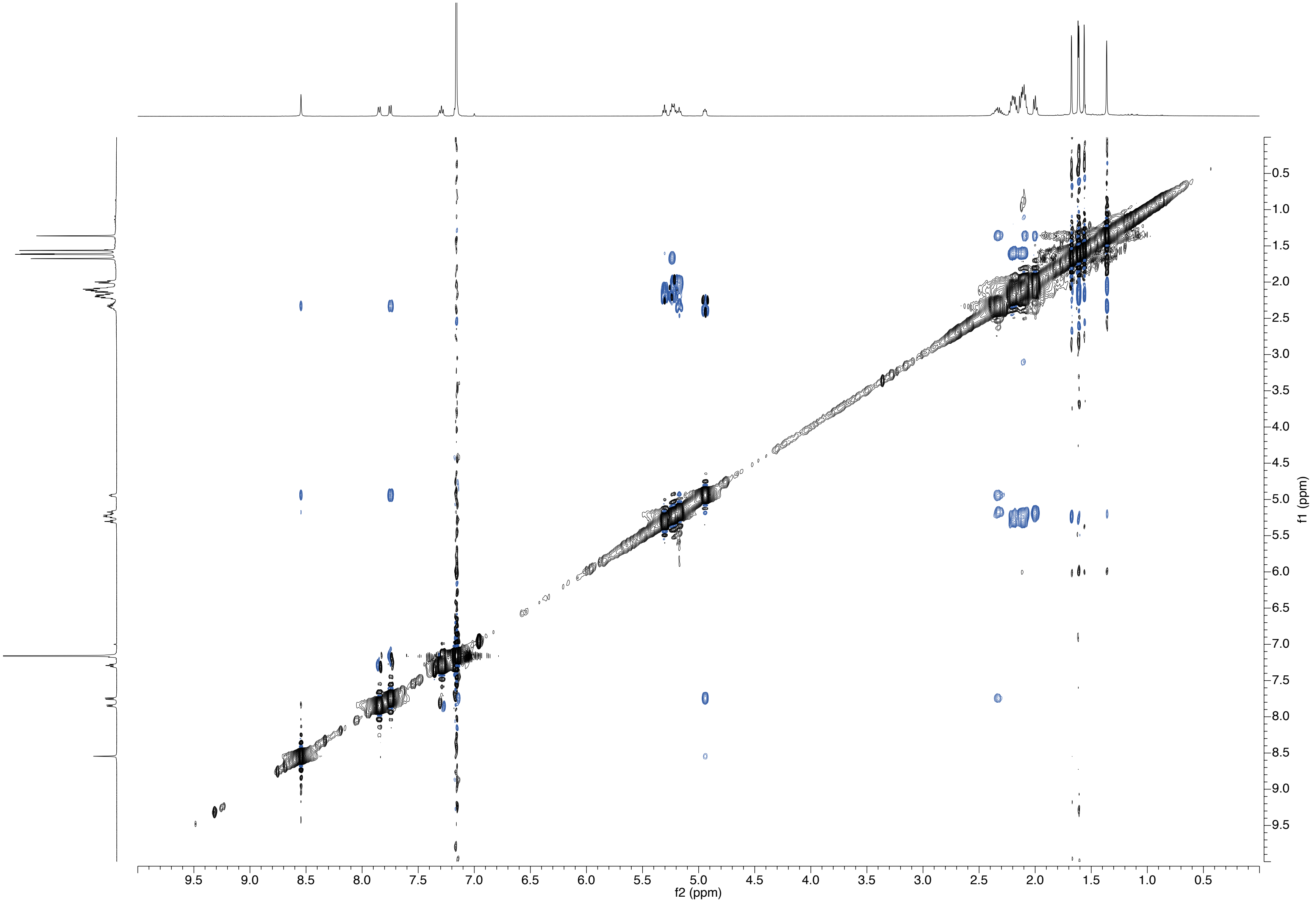

gNOESY spectrum of 4-(geranylgeranyl(hydroxy)methyl)quinaldic acid (**12**) (298K, C_6D_6)

7. Cartesian Coordinates of DFT-Optimized Structures

Gibbs free energies (in Hartree) are provided at the indicated level of theory. For conformers used in ECD calculations, Boltzmann populations (%) at 298.15K are reported.

(5*S*,8*R*,9*R*,10*S*,13*S*,14*R*,15*R*)-**1**

M06-2X/def2-TZVP/SMD(MeOH)

Conformer 1 (Boltzmann population 75.6%)

$\Delta G = -1711.465683$ Hartree

C	7.36083000	-0.41554700	-1.08520500
C	6.63600800	0.93656700	-0.94984300
C	5.12533400	0.64749600	-0.72184300
C	4.77897300	-0.32361900	0.45077300
C	5.58926900	-1.62189400	0.24936400
C	7.08557900	-1.37613200	0.06517100
H	7.02951700	-0.88722800	-2.02791500
H	8.44462400	-0.23619600	-1.18614900
H	5.43359900	-2.29606500	1.10613300
H	5.20044100	-2.14361800	-0.64387100
H	7.52376200	-0.98153000	0.99600100
H	7.59164900	-2.33607800	-0.12252000
H	4.82584600	0.08602300	-1.62828200
C	3.26710900	-0.66060900	0.29325000
H	3.17392300	-1.04964900	-0.73748000
C	2.32755900	0.56531000	0.30683400
C	2.75831200	1.53547100	-0.79199200
H	2.12779100	2.43882600	-0.74739900
H	2.56531800	1.04523200	-1.75981600
C	4.23979800	1.89757100	-0.69723300
H	4.43152600	2.49079700	0.21139200
H	4.48815000	2.55096500	-1.54540600
C	5.09309500	0.23188700	1.85434900
H	4.97609800	1.32061600	1.92703500
H	4.43286800	-0.22611700	2.60576000
H	6.12184400	-0.00244500	2.15663000
C	2.70224300	-1.75784500	1.19819500
H	3.36162400	-2.63616700	1.20421200
H	2.61688200	-1.41474400	2.24212200
C	1.33245700	-2.18111700	0.66788500
H	0.86768500	-2.94185100	1.30921300
H	1.46081000	-2.62707000	-0.33161300
O	1.01536000	0.11853000	-0.10528800
C	7.28619900	1.78528200	0.15149000
H	7.39343500	1.25060500	1.10334300
H	8.29553600	2.09033700	-0.16884800
H	6.71081000	2.70472700	0.34148800
C	6.81719400	1.68804500	-2.27694200
H	7.88108200	1.69019900	-2.56275700
H	6.25018200	1.20681200	-3.09019600
H	6.49570300	2.73775500	-2.20872300
C	2.21408400	1.31860800	1.63753900
H	2.23325900	0.66444100	2.51826300
H	3.02691300	2.04543200	1.74865200
H	1.26613700	1.87745500	1.63388100
C	0.39141400	-0.99289500	0.53445100
C	-0.83361500	-1.25267000	-0.35184700
C	-0.33572400	-0.58388200	1.82333800
C	-1.72784400	-0.05230900	0.01966000
H	-0.58856400	-1.25384000	-1.42742300
H	0.25780700	0.02390900	2.51126100
H	-0.68268600	-1.48675400	2.34865100
O	-1.45847000	0.18684100	1.38853200
O	-1.46164800	1.08492600	-0.73349700
H	-0.49588300	1.21746200	-0.69119700
C	-3.19616200	-0.36612500	-0.15683000
C	-2.63688800	-2.75020300	-0.59819100
H	-2.45066400	-2.97297600	-1.66611300
H	-3.04242600	-3.66025100	-0.13592300
O	-1.41946900	-2.45909200	0.05705300
C	-3.62581100	-1.62807900	-0.45620500
C	-5.03365600	-1.90320600	-0.65529800
O	-5.44763300	-3.02700700	-0.96569300
C	-5.40131800	0.50243700	-0.14326100
H	-3.68285000	1.60151700	0.17816600
N	-4.04905300	0.66864800	-0.00543800
C	-8.15769000	0.18941500	-0.41233100
H	-9.23894000	0.08413000	-0.51297600
C	-7.62453500	1.45907300	-0.09628000
H	-8.29577400	2.30633000	0.04129200
C	-6.25833400	1.62282000	0.03851400
C	-7.33384700	-0.90019800	-0.59289300
H	-7.73360000	-1.88423400	-0.83931100
C	-5.93573000	-0.75545500	-0.45968400
O	-5.62223800	2.77349000	0.33721900
C	-6.40455200	3.93615500	0.53657500
H	-7.10021400	3.80115000	1.37901700
H	-5.70387200	4.74589400	0.76660200
H	-6.97170800	4.18804400	-0.37278900

Conformer 2 (Boltzmann population 24.4%)

$\Delta G = -1711.464617$ Hartree

C	-5.68506800	-0.16483700	2.72922800
C	-5.58245000	1.07298000	1.82257900
C	-4.36220100	0.88086500	0.88418000
C	-4.32756000	-0.44829300	0.07556400
C	-4.48286800	-1.62145300	1.06185500
C	-5.70249500	-1.48458600	1.96863100
H	-4.82308600	-0.16177700	3.40740800
H	-6.57882400	-0.07466900	3.35478500
H	-4.53722900	-2.56242900	0.50734100
H	-3.58416900	-1.67105300	1.68856100
H	-6.62271000	-1.56730300	1.38478800

H	-5.71902300	-2.31590500	2.67789100
H	-3.51071900	0.80132600	1.57591000
C	-2.90257400	-0.54496000	-0.54344900
H	-2.23099800	-0.45432500	0.31683300
C	-2.51393500	0.64108400	-1.43803300
C	-2.67897300	1.94386100	-0.65942000
H	-2.47163500	2.78725000	-1.32451500
H	-1.92017500	1.95411600	0.12969400
C	-4.05788800	2.07935700	-0.01794200
H	-4.82487000	2.19292100	-0.78787300
H	-4.07106900	3.00153800	0.56450400
C	-5.43489100	-0.54963100	-0.99242200
H	-5.75578700	0.42094600	-1.36659900
H	-5.10474800	-1.13563800	-1.85031200
H	-6.31872600	-1.04763700	-0.59661500
C	-2.58346800	-1.89266300	-1.21299300
H	-2.31985400	-2.60960500	-0.43341400
H	-3.45835800	-2.29910700	-1.72255600
C	-1.42849600	-1.80095300	-2.21884600
H	-1.79745200	-1.59523100	-3.22508500
H	-0.88925000	-2.74575500	-2.27223400
O	-1.08597900	0.56987200	-1.70887800
C	-6.91045000	1.31111500	1.09560700
H	-7.28251800	0.42607100	0.58323700
H	-7.66955700	1.61232800	1.82309400
H	-6.82366600	2.11581300	0.36166800
C	-5.33788500	2.28464000	2.73082300
H	-6.06818800	2.28919600	3.54439800
H	-4.33915900	2.25273600	3.17482900
H	-5.44322200	3.22922300	2.19438000
C	-3.24320500	0.72989400	-2.77944900
H	-3.39874900	-0.24126600	-3.24704700
H	-4.21612700	1.20500900	-2.66655500
H	-2.64663500	1.34799100	-3.45323400
C	-0.45086900	-0.70089600	-1.86693100
C	0.40427800	-0.93834100	-0.60914200
C	0.64877700	-0.51238200	-2.90584700
C	1.52469300	0.09286800	-0.81906200
H	-0.12298600	-0.76034900	0.33136900
H	0.31782800	0.12684600	-3.72349000
H	0.98996200	-1.47281100	-3.29624000
O	1.73539400	0.13043400	-2.21892600
O	1.19033700	1.36335700	-0.33818200
H	0.31074300	1.57828900	-0.69620200
C	2.80762100	-0.30886800	-0.13383300
C	1.82958700	-2.52636000	0.39174700
H	1.28572800	-2.55621400	1.34448500
H	2.22850900	-3.52239200	0.20169900
O	0.92974800	-2.24278600	-0.66692900
C	2.94804400	-1.52926000	0.44875000
C	4.16409100	-1.87445300	1.13461400
O	4.30132200	-2.96480400	1.71605900
C	4.99691500	0.35358400	0.46976500
H	3.63670700	1.51634100	-0.54846200
N	3.79294200	0.60854100	-0.12566800
C	7.44836400	-0.14071900	1.66612400
H	8.41176100	-0.31779400	2.12655300
C	7.21941700	1.09440300	1.02986300
H	8.00275500	1.83910000	1.01115300
C	6.00641800	1.34610800	0.43656100
C	6.47578400	-1.10447100	1.70802600
H	6.64698700	-2.05369700	2.19756900
C	5.22641100	-0.86817500	1.10484800
O	5.65570500	2.48440500	-0.20312800
C	6.61947100	3.52799300	-0.26107600
H	7.51289400	3.19762000	-0.79461800
H	6.14698900	4.34252700	-0.80337500
H	6.88823600	3.85945000	0.74391500

(5*S*,8*R*,9*R*,10*S*,13*S*,14*R*,15*S*)-**1**

M06-2X/def2-TZVP/SMD(MeOH)

Conformer 1 (Boltzmann population 64.4%)

$\Delta G = -1711.452891$ Hartree

C	-7.35542200	1.07156800	0.77141600
C	-6.89851700	0.66801900	-0.63975100
C	-5.35679500	0.50030900	-0.61338100
C	-4.78966800	-0.45657400	0.47746000
C	-5.34009300	-0.00123400	1.84379800
C	-6.85995400	0.13599100	1.86535100
H	-6.97888900	2.08150800	0.97432300
H	-8.44828200	1.13248800	0.78896700
H	-5.03149200	-0.70586700	2.61980500
H	-4.89423600	0.96853000	2.09571600
H	-7.33099000	-0.84448500	1.75830000
H	-7.17214800	0.51510800	2.84175900
H	-4.99163900	1.49358900	-0.31304400
C	-3.24965600	-0.23586100	0.47997400
H	-3.12286300	0.84003600	0.66067600
C	-2.55377300	-0.46778900	-0.87510000
C	-3.21197400	0.40653200	-1.93682200
H	-2.76359200	0.19195100	-2.91086600
H	-2.98733900	1.44956700	-1.69450700
C	-4.72752000	0.22316800	-1.98089500
H	-4.97941900	-0.78166900	-2.32943400
H	-5.12973700	0.91258800	-2.72417200
C	-5.15879500	-1.94030200	0.27892100
H	-5.32583100	-2.20690500	-0.76334600
H	-4.36974800	-2.58882200	0.66366600
H	-6.06787500	-2.19709000	0.82115200
C	-2.45712800	-0.93813700	1.57899000
H	-2.91535800	-0.77473500	2.55445900
H	-2.42328900	-2.01908500	1.42233700
C	-1.04319700	-0.36425900	1.61584300
H	-0.43506300	-0.88195800	2.35550300
H	-1.10258200	0.68633800	1.91248400
O	-1.20125400	0.04427400	-0.78016100
C	-7.66102600	-0.57443200	-1.11205500
H	-7.62694100	-1.39454400	-0.39738800
H	-8.71277800	-0.31576900	-1.26441500

H	-7.27334000	-0.94026400	-2.06582300
C	-7.27144800	1.81824000	-1.58367200
H	-8.31517200	2.10173900	-1.42373200
H	-6.65105300	2.69961400	-1.39929000
H	-7.16763700	1.54081000	-2.63395700
C	-2.50219200	-1.91578600	-1.36058900
H	-2.15505000	-2.61495700	-0.59955100
H	-3.47900800	-2.25310000	-1.69651100
H	-1.82541400	-1.96866700	-2.21514700
C	-0.34796200	-0.42789600	0.25688100
C	0.90694100	0.43838900	0.17451100
C	0.29356800	-1.81811600	-0.06348200
H	1.07912300	0.62687400	-0.89274700
H	0.07592100	-2.12950700	-1.08035100
H	-0.02379300	-2.58806200	0.64070200
C	3.32222200	0.16196400	0.31245000
C	2.12241100	2.37623100	0.50332000
H	1.90910900	2.91088800	-0.43015100
H	2.29616900	3.11837100	1.28223900
O	0.95603600	1.64046300	0.89179500
C	3.35882700	1.52674200	0.30815500
C	4.59855000	2.20799300	0.05314300
O	4.66991900	3.44865600	0.01415800
C	5.62999600	-0.03364300	-0.14065400
H	4.32409400	-1.61038600	0.10961700
N	4.40710500	-0.60001100	0.10103300
C	8.11489000	1.08975600	-0.62912700
H	9.09285800	1.51237000	-0.82007100
C	7.97606700	-0.31072200	-0.60693500
H	8.84062000	-0.93577500	-0.78086100
C	6.74621900	-0.87385700	-0.36587500
C	7.03862500	1.91043200	-0.41548200
H	7.14220300	2.98689700	-0.43412500
C	5.77000800	1.35512000	-0.16657300
O	6.47313500	-2.19700500	-0.31705500
C	7.55007800	-3.09827700	-0.53997300
H	8.32443100	-2.96807400	0.21854900
H	7.12629400	-4.09610000	-0.46529300
H	7.97549300	-2.95069400	-1.53460300
C	2.00806300	-0.48282200	0.65785400
O	1.97332900	-0.71167900	2.05179800
H	1.85979600	0.13602900	2.50874200
O	1.74091600	-1.68010700	0.02033600

Conformer 2 (Boltzmann population 35.6%)

$\Delta G = -1711.452333$ Hartree

C	-7.35230200	1.08887000	0.72640400
C	-6.88349500	0.67066500	-0.67656600
C	-5.34263400	0.49839800	-0.63406200
C	-4.78882600	-0.45031600	0.47065100
C	-5.35045300	0.01956100	1.82747000
C	-6.86997700	0.16204700	1.83362300
H	-6.97444300	2.09949100	0.92335100
H	-8.44508200	1.15333500	0.73322200
H	-5.05146700	-0.67866700	2.61295700
H	-4.90363400	0.99014300	2.07441000
H	-7.34337500	-0.81782600	1.73152100
H	-7.18986100	0.55145700	2.80347100
H	-4.97713300	1.49320400	-0.33927000
C	-3.24808600	-0.23481800	0.48604000
H	-3.11928000	0.84198100	0.65978200
C	-2.53997800	-0.47933900	-0.86045600
C	-3.18584700	0.38732700	-1.93590100
H	-2.72852100	0.16328600	-2.90364400
H	-2.96101300	1.43187200	-1.70038600
C	-4.70125900	0.20703800	-1.99300000
H	-4.95208600	-0.80049100	-2.33450000
H	-5.09467700	0.89037000	-2.74653500
C	-5.16129200	-1.93447600	0.28208400
H	-5.31975100	-2.20987300	-0.75924200
H	-4.37806400	-2.58218400	0.67986800
H	-6.07615300	-2.18331400	0.81825300
C	-2.46819800	-0.93158600	1.59765000
H	-2.93517700	-0.75947700	2.56750400
H	-2.43705500	-2.01380300	1.44930400
C	-1.05283000	-0.36208100	1.64366000
H	-0.45191200	-0.87504800	2.39194800
H	-1.11141300	0.69040800	1.93330400
O	-1.18753100	0.03035500	-0.75690800
C	-7.64559600	-0.57377800	-1.14436400
H	-7.62217500	-1.38672600	-0.42112000
H	-8.69471700	-0.31291800	-1.31050300
H	-7.24919300	-0.95068900	-2.09020900
C	-7.24372800	1.81301400	-1.63486700
H	-8.28796000	2.10146000	-1.48756400
H	-6.62212300	2.69408100	-1.45313900
H	-7.13096400	1.52513400	-2.68142100
C	-2.48804700	-1.93162900	-1.33305200
H	-2.14892100	-2.62493900	-0.56310700
H	-3.46304100	-2.26929000	-1.67397700
H	-1.80436000	-1.99382200	-2.18146200
C	-0.34468300	-0.43548800	0.29209200
C	0.90969700	0.43218800	0.21615900
C	0.30134800	-1.82685700	-0.01459300
H	1.09577800	0.59465000	-0.85320100
H	0.08111600	-2.15124700	-1.02673300
H	-0.01356000	-2.58912200	0.69942100
C	3.32009800	0.16339900	0.35936400
C	2.11701500	2.37775700	0.55023100
H	1.92932200	2.93536800	-0.37559800
H	2.28422500	3.10268000	1.34701800
O	0.93586600	1.64982900	0.90051900
C	3.35240100	1.52762300	0.35135000
C	4.58503200	2.21100800	0.06956100
O	4.65289800	3.45180900	0.02706000
C	5.61753400	-0.02783800	-0.14930000
H	4.31827700	-1.60742100	0.11318900
N	4.40135700	-0.59700700	0.11885900
C	8.08823200	1.10125500	-0.69079100
H	9.06080200	1.52635600	-0.90263500
C	7.95259000	-0.29953600	-0.66981100

H	8.81398600	-0.92248100	-0.86555800
C	6.72962000	-0.86556200	-0.40215800
C	7.01565600	1.91937700	-0.45025700
H	7.11699200	2.99605100	-0.46811200
C	5.75393400	1.36111300	-0.17451200
O	6.46011200	-2.18943300	-0.35056400
C	7.53358900	-3.08803500	-0.60006000
H	8.32500600	-2.95736200	0.14054600
H	7.11380800	-4.08691800	-0.51729000
H	7.93542100	-2.93785400	-1.60403300
C	2.00993600	-0.48215100	0.71647600
O	1.91794800	-0.61846400	2.11180600
H	2.67454200	-1.12735300	2.43666900
O	1.74716000	-1.68239300	0.06272700

(5*S*,8*R*,9*R*,10*S*,13*S*,14*R*)-**2**

PBE0-D3(BJ)/def2-TZVP/CPCM(MeOH)//
PBE0/def2-TZVP/CPCM(MeOH)

Conformer 1 (Boltzmann population 98.7%)

Δ*G* = −1635.099993 Hartree

O	3.64402200	2.71746300	1.82799900
O	4.11406000	-1.31234000	-2.50524600
O	2.00356400	-3.59006000	0.33520400
O	1.21558700	-1.03023300	2.60030400
O	-0.35550600	-1.75434100	-0.31130700
N	2.90940900	0.31805700	1.00904800
H	2.56681200	0.70387600	1.87893300
C	4.00186600	3.99742800	2.31737000
H	3.67543900	4.78380500	1.63145600
H	5.08197400	4.06961200	2.47104100
H	3.49033200	4.10993400	3.27037100
C	4.15244800	2.33092300	0.64203900
C	3.74636700	1.04269900	0.21946100
C	4.19891400	0.51924000	-0.99881100
C	5.06444100	1.28833100	-1.79177700
H	5.40684200	0.87109800	-2.73013400
C	5.45568600	2.53428200	-1.37372800
H	6.12419600	3.12997300	-1.98386300
C	5.00103800	3.06374500	-0.15492400
H	5.32296500	4.05006000	0.15152000
C	3.76621400	-0.81223500	-1.43054300
C	2.90310900	-1.51196300	-0.50393300
C	2.50281300	-0.91552600	0.65153100
C	1.55308600	-1.57789500	1.57281000
C	1.05232300	-2.94571300	1.15410800
H	0.99877600	-3.55760300	2.05846600
C	2.43721000	-2.89113200	-0.81475400
H	1.64914300	-2.85539400	-1.57785100
H	3.26452600	-3.47121100	-1.22827800
C	-0.38570600	-2.87654300	0.57059600
C	-0.66753000	-4.19821700	-0.14088900
H	-1.72964000	-4.31490300	-0.35187800
H	-0.36386800	-5.02949500	0.49910200
H	-0.11626200	-4.27251500	-1.07750600
C	-1.38113300	-2.66333700	1.71381600
H	-0.95930600	-1.95781500	2.43392000
H	-1.51768600	-3.61387700	2.23765000
C	-2.71379600	-2.11025500	1.22745600
H	-3.24806000	-2.85401700	0.62889200
H	-3.34156800	-1.90987800	2.09765000
C	-2.45838700	-0.83335700	0.43589300
H	-1.80152500	-0.22041000	1.07056900
C	-1.57943400	-1.15719800	-0.78631500
C	-2.20582100	-2.05245800	-1.85630700
H	-1.40237200	-2.48530800	-2.45506200
H	-2.84045400	-1.47524400	-2.52673500
H	-2.80933300	-2.86470600	-1.45452100
C	-1.13243800	0.14874100	-1.43191100
H	-0.44557400	0.64532400	-0.73832900
H	-0.56676000	-0.07123800	-2.34327800
C	-2.29999400	1.07932000	-1.72974300
H	-2.94166200	0.65145500	-2.50647800
H	-1.90002600	2.00341600	-2.15210300
C	-3.10509600	1.38296200	-0.46931200
H	-2.36895100	1.72552300	0.27555700
C	-3.68412300	0.08152500	0.15945400
C	-4.74727000	-0.62907600	-0.68995400
H	-5.73810300	-0.20801000	-0.52215800
H	-4.80759100	-1.68546000	-0.41927600
H	-4.55435600	-0.57390000	-1.76028900
C	-4.32065600	0.45572300	1.50922800
H	-3.52320900	0.75420700	2.20242900
H	-4.80700600	-0.41990900	1.94911600
C	-5.32747900	1.59151200	1.40443900
H	-6.20178400	1.27407700	0.82688400
H	-5.70558100	1.83197400	2.40313000
C	-4.71021100	2.82983200	0.78088600
H	-3.92841500	3.20646800	1.45358400
H	-5.45588700	3.62842700	0.69536200
C	-4.07377700	2.58816200	-0.59516100
C	-5.15348700	2.42481500	-1.66675700
H	-4.71610300	2.18243100	-2.63867400
H	-5.69931300	3.36647700	-1.77999000
H	-5.88214500	1.65163000	-1.42731300
C	-3.27455600	3.84780500	-0.94367500
H	-2.41478300	3.96921300	-0.27786900
H	-3.91102600	4.73089200	-0.83344300
H	-2.91004700	3.83787800	-1.97313300

Conformer 2 (Boltzmann population 1.3%)

Δ*G* = −1635.095935 Hartree

O	3.99613500	3.24542800	0.64732900
O	5.82521400	-2.20762700	-0.82122600
O	1.69822500	-2.87988000	-0.42654400
O	0.86052900	0.07096400	1.30733800
O	-0.83933000	-1.57871300	-0.57993100
N	3.29284800	0.71281800	0.41270200
H	2.65062400	1.40413300	0.77726700

C	4.31649500	4.61707300	0.79563200
H	4.60976500	5.05581400	-0.16192000
H	5.12017000	4.75453000	1.52403400
H	3.41174200	5.10093100	1.15590600
C	4.96243700	2.41618700	0.20862500
C	4.56724100	1.06256600	0.08966200
C	5.47714400	0.09323500	-0.35259600
C	6.78409500	0.48410700	-0.68094000
H	7.47603400	-0.27511500	-1.02230400
C	7.15837100	1.79827900	-0.56547200
H	8.16697600	2.10285700	-0.81836600
C	6.25051400	2.77195700	-0.11853400
H	6.57107000	3.80180900	-0.03460300
C	5.05975300	-1.30690000	-0.46385500
C	3.67467300	-1.56389200	-0.13462800
C	2.86568100	-0.55882400	0.30029600
C	1.48407200	-0.82313800	0.78341100
C	1.02741900	-2.27289100	0.65342200
H	1.31562100	-2.76919100	1.59820700
C	3.09694500	-2.93041300	-0.27370700
H	3.50082400	-3.41458100	-1.16424900
H	3.36594900	-3.55795700	0.59142900
C	-0.48952300	-2.47006400	0.47406400
C	-0.72879800	-3.93650200	0.10578900
H	-1.78078700	-4.19880400	0.20911700
H	-0.15968200	-4.58279200	0.77829200
H	-0.41195300	-4.14123000	-0.91515800
C	-1.21686100	-2.14459400	1.78422900
H	-0.75822800	-1.27268100	2.25119200
H	-1.08909300	-2.98919100	2.46762300
C	-2.69316900	-1.84410600	1.56175100
H	-3.23297800	-2.74509700	1.25559000
H	-3.12880000	-1.53604700	2.51403400
C	-2.81899600	-0.73690100	0.52200200
H	-2.12674300	0.05216700	0.84858900
C	-2.21404200	-1.21797400	-0.81077000
C	-2.93797300	-2.37242300	-1.50664500
H	-2.24337000	-2.83727400	-2.20869400
H	-3.79207500	-2.01291000	-2.07834800
H	-3.30106400	-3.14081400	-0.82602000
C	-2.13354600	-0.03705600	-1.77094000
H	-1.38364800	0.65902400	-1.38086300
H	-1.77398000	-0.38547700	-2.74484000
C	-3.46278500	0.69123100	-1.91205900
H	-4.20070800	0.05001400	-2.40437500
H	-3.31693900	1.54201700	-2.58086700
C	-3.97962200	1.16983800	-0.55829800
H	-3.14473500	1.72746900	-0.10432900
C	-4.19623300	-0.02311400	0.41821600
C	-5.31455200	-0.99426500	0.01477000
H	-6.28903000	-0.64336800	0.35328600
H	-5.15613700	-1.97026100	0.47885600
H	-5.38703300	-1.14921800	-1.06069500
C	-4.54343100	0.55875000	1.79962600
H	-3.65689900	1.06976400	2.19769000
H	-4.77908000	-0.24993700	2.49762500
C	-5.70079900	1.54582000	1.76286700
H	-6.63106700	1.03346900	1.49670200
H	-5.86348600	1.94899900	2.76747300
C	-5.43461900	2.68278200	0.79374500
H	-4.57870000	3.26297400	1.16303500
H	-6.28677000	3.37146400	0.76685900
C	-5.11412700	2.22511900	-0.63605800
C	-6.38154900	1.74218000	-1.34402700
H	-6.15651800	1.35062600	-2.33940500
H	-7.07017200	2.58299300	-1.47190800
H	-6.91158200	0.96643700	-0.79312600
C	-4.61109800	3.45708400	-1.39526500
H	-3.64528300	3.79509700	-1.00767400
H	-5.32455100	4.27852200	-1.27968000
H	-4.50251000	3.27083200	-2.46590800

(2′*R*,3′*R*)-**4a**

M06-2X/def2-TZVPP//M06-2X/def2-TZVP

Conformer 1 (Boltzmann population 19.3%)

Δ*G* = −1015.754420 Hartree

C	-1.13364200	-1.97807800	0.26072800
C	-0.08643000	-1.10699700	0.40871200
N	-0.27791000	0.24394100	0.33693600
C	-1.51092200	0.80040900	0.12072100
C	-2.63628500	-0.00871000	-0.05412700
C	-2.48009100	-1.47350000	-0.01476700
C	-1.63683700	2.21412400	0.07541800
C	-2.88007500	2.77821200	-0.14175900
C	-4.01310600	1.95289300	-0.31662700
C	-3.89679000	0.58065500	-0.27511700
O	-3.43044400	-2.23092300	-0.20188900
C	1.34626200	-1.50106300	0.68167000
C	-1.05875700	-3.48470500	0.36015400
O	-1.49621600	-4.11898200	-0.80942800
C	2.29676100	-0.83142600	-0.28346200
C	3.63255600	-0.30686400	0.06462200
C	4.65664800	-0.17825900	-1.04697500
C	4.18956900	-0.39055300	1.46471900
O	2.51546600	0.56044300	-0.10129000
O	-0.47650100	2.88634300	0.25825200
C	-0.50786000	4.28914900	0.20274000
C	5.43492000	1.13288900	-0.97848200
H	0.52306100	0.86978600	0.40677000
H	-2.99267800	3.86101200	-0.17916300
H	-4.98358700	2.41978100	-0.48748500
H	-4.74871300	-0.08574000	-0.41018100
H	1.59580700	-1.22040000	1.71509300
H	1.46093100	-2.58661500	0.59656900
H	-0.03587500	-3.83077200	0.55639400
H	-1.67217300	-3.79022400	1.22977100
H	-2.41726300	-3.83723000	-0.91661500
H	2.13620300	-1.11776900	-1.33106200
H	5.34049100	-1.04101800	-1.00247900

H	4.12529600	-0.23816100	-2.00816800
H	4.82890400	-1.27874300	1.56963700
H	4.80142800	0.49770200	1.67497900
H	3.39678500	-0.43055700	2.21948600
H	0.51943200	4.63416100	0.35984800
H	-0.86446100	4.64034300	-0.77927300
H	-1.15712000	4.70445600	0.99079100
H	6.07891000	1.25160500	-1.85998900
H	4.73678800	1.98176800	-0.94809200
H	6.07924300	1.18493400	-0.08955300

Conformer 2 (Boltzmann population 17.9%)

$\Delta G = -1015.754349$ Hartree

C	0.75737500	2.01927300	0.31689100
C	-0.09782400	0.95450900	0.42877400
N	0.35621200	-0.32886900	0.31416800
C	1.67468700	-0.62397600	0.08857900
C	2.61910800	0.39607100	-0.05028700
C	2.17740100	1.79922700	0.03670900
C	2.07579900	-1.98306300	-0.00417400
C	3.40570400	-2.28422800	-0.23109900
C	4.35466700	-1.24683400	-0.36882100
C	3.97106300	0.07357400	-0.28147100
O	2.96043600	2.73487200	-0.11659400
C	-1.57965300	1.05038800	0.70717300
C	0.38683200	3.47758400	0.46401100
O	0.69711300	4.22454900	-0.67938400
C	-2.38764100	0.22539800	-0.26761300
C	-3.57809500	-0.56922200	0.09368100
C	-4.59462000	-0.85076200	-0.99288300
C	-4.09745900	-0.62343200	1.51107600
O	-2.31683000	-1.18658500	-0.12656600
O	1.06981300	-2.87565200	0.14633200
C	1.37493200	-4.24214500	0.03887800
C	-5.73574400	0.16083400	-1.01847300
H	-0.30566000	-1.10235500	0.35828500
H	3.72883800	-3.32186500	-0.30481100
H	5.39810500	-1.50780500	-0.54807500
H	4.67577600	0.89835500	-0.38719200
H	-1.76834300	0.70921300	1.73558700
H	-1.90716500	2.09319200	0.64173300
H	-0.68523500	3.60886500	0.65916500
H	0.92337600	3.86832400	1.35023700
H	1.65598900	4.13108400	-0.78576400
H	-2.31402800	0.56578700	-1.30902400
H	-4.06501600	-0.86172700	-1.95688400
H	-4.98844500	-1.86878600	-0.83833800
H	-4.72353900	0.25165600	1.73652300
H	-4.71141300	-1.52615600	1.64608500
H	-3.27407900	-0.66638700	2.23296500
H	0.43461800	-4.78681000	0.17365400
H	1.79485300	-4.47883800	-0.95232200
H	2.09167700	-4.55257800	0.81666900
H	-6.41773400	-0.03928800	-1.85543500
H	-6.32879400	0.12647600	-0.09332000
H	-5.35072500	1.18506400	-1.13491600

Conformer 3 (Boltzmann population 17.7%)

$\Delta G = -1015.754338$ Hartree

C	0.77990400	2.01626200	-0.02629100
C	-0.09125500	0.98919200	0.22622100
N	0.33758300	-0.30752800	0.25865800
C	1.64424100	-0.65480400	0.03819900
C	2.60788700	0.32465500	-0.21354500
C	2.20177000	1.74090100	-0.24101400
C	2.01373900	-2.02549600	0.06989200
C	3.33187900	-2.37830600	-0.15168400
C	4.30038700	-1.38160600	-0.40488200
C	3.94795100	-0.04999400	-0.43494400
O	3.01529500	2.64299400	-0.43260900
C	-1.56595000	1.14222000	0.51625000
C	0.42561700	3.48416300	-0.11270900
O	1.14956600	4.26168400	0.79954000
C	-2.41186500	0.25223200	-0.36445900
C	-3.60404300	-0.48833400	0.09384500
C	-4.65282300	-0.84611600	-0.93831900
C	-4.08637700	-0.41100900	1.52319400
O	-2.35801000	-1.14319100	-0.10440800
O	0.99222400	-2.87578000	0.32472400
C	1.26665200	-4.25279100	0.34736300
C	-5.78011800	0.17765800	-1.02301000
H	-0.33969300	-1.05672400	0.39383500
H	3.63069700	-3.42558700	-0.13099000
H	5.33435900	-1.68302600	-0.57530300
H	4.66919200	0.74478200	-0.62520600
H	-1.73530100	0.89311300	1.57393200
H	-1.87510600	2.18262400	0.37411500
H	0.61730000	3.81270300	-1.15269600
H	-0.63918800	3.65875700	0.08735300
H	2.07927200	4.12462500	0.56197300
H	-2.36161900	0.49934000	-1.43339100
H	-4.14905300	-0.94942900	-1.91072600
H	-5.05662500	-1.84041800	-0.68609200
H	-4.69038800	0.49252900	1.68844800
H	-4.71233100	-1.28650200	1.75068900
H	-3.24510200	-0.40808100	2.22539700
H	0.31849800	-4.76008100	0.55391400
H	1.65994400	-4.59566500	-0.62353800
H	1.99366000	-4.50100500	1.13784700
H	-6.48697600	-0.08297500	-1.82182400
H	-6.34859000	0.23331400	-0.08361100
H	-5.38420500	1.18177900	-1.23736000

Conformer 4 (Boltzmann population 16.2%)

$\Delta G = -1015.754252$ Hartree

C	1.15950800	1.95825300	-0.07895400
C	0.10175200	1.13321600	0.19977100
N	0.26413200	-0.22145000	0.27397300
C	1.47528500	-0.82828900	0.07078700
C	2.61447000	-0.06805800	-0.20461800

C	2.49828700	1.39926200	-0.27634200
C	1.56451800	-2.24354400	0.14481700
C	2.78585300	-2.85808500	-0.05913400
C	3.93318300	-2.08206200	-0.33652400
C	3.85291700	-0.70847300	-0.40776500
O	3.47490300	2.11541300	-0.48956600
C	-1.31375300	1.58405100	0.47386100
C	1.10447400	3.46379700	-0.21255700
O	1.96623800	4.10985000	0.68231300
C	-2.30903100	0.86506500	-0.40660000
C	-3.64049000	0.39305900	0.02462800
C	-4.70466200	0.20069300	-1.03915000
C	-4.14721100	0.59021500	1.43240700
O	-2.54380800	-0.50476500	-0.11367500
O	0.39410900	-2.86556500	0.41861100
C	0.38944400	-4.26821700	0.48528200
C	-5.50537800	-1.08366900	-0.84218300
H	-0.54886200	-0.81654100	0.42569600
H	2.87026800	-3.94277700	-0.00581800
H	4.88635700	-2.58818400	-0.49240600
H	4.71796100	-0.07936200	-0.61751900
H	-1.53455100	1.38620400	1.53252400
H	-1.40713100	2.66316600	0.31610300
H	1.36064700	3.71482000	-1.26021400
H	0.09508400	3.85270400	-0.02763600
H	2.85084900	3.78432300	0.45646600
H	-2.18180600	1.06826500	-1.47813900
H	-5.37002500	1.07893700	-1.04004300
H	-4.20558500	0.17366000	-2.01903000
H	-4.77788200	1.48885300	1.48970200
H	-4.75595300	-0.27418000	1.73214400
H	-3.32884700	0.68299500	2.15448000
H	-0.64085000	-4.57047400	0.70019000
H	0.70798800	-4.71279100	-0.47167300
H	1.05143600	-4.63101700	1.28849500
H	-6.18031400	-1.25764400	-1.69075700
H	-4.82328800	-1.94266500	-0.76594400
H	-6.12095600	-1.05089500	0.06774500

Conformer 5 (Boltzmann population 9.6%)

$\Delta G = -1015.753756$ Hartree

C	-0.85525800	2.02048300	-0.31510000
C	0.08112000	1.03491700	-0.48663000
N	-0.25989200	-0.28483000	-0.39316200
C	-1.53999300	-0.69594400	-0.13067500
C	-2.56057700	0.23704400	0.06838300
C	-2.24114500	1.67419100	0.00535900
C	-1.82288900	-2.08582200	-0.06186400
C	-3.11410100	-2.50338600	0.20148700
C	-4.14068100	-1.55343100	0.39975500
C	-3.87177600	-0.20366300	0.33603900
O	-3.09402900	2.53605000	0.20999200
C	1.53859600	1.26163700	-0.81304600
C	-0.61304800	3.50821000	-0.43213200
O	-0.94629200	4.19618000	0.74142400
C	2.44295800	0.48848400	0.11956400
C	3.68854700	-0.18977000	-0.29161400
C	4.79232400	-0.44598900	0.72054600
C	4.16753100	-0.17096300	-1.72348000
O	2.49139600	-0.92113500	-0.05559100
O	-0.75214700	-2.88676600	-0.27070600
C	-0.93931200	-4.27638300	-0.19401300
C	4.35228000	-0.46446600	2.17822500
H	0.46203400	-0.99830800	-0.48232100
H	-3.34675600	-3.56605700	0.25746800
H	-5.15162000	-1.90584500	0.60665600
H	-4.63833800	0.55618600	0.48805200
H	1.71653800	0.95799400	-1.85458900
H	1.77979200	2.32679300	-0.73474600
H	0.43717000	3.73469700	-0.65653700
H	-1.20972800	3.87520400	-1.28961200
H	-1.88976800	4.01865900	0.87520500
H	2.35223200	0.79298700	1.16866300
H	5.23629500	-1.42007200	0.45623300
H	5.58294800	0.30431700	0.56003800
H	4.80736200	0.70454500	-1.90479900
H	4.76361100	-1.07364000	-1.92134800
H	3.33367300	-0.15862300	-2.43378900
H	0.03711000	-4.73677400	-0.37751000
H	-1.30146500	-4.57432300	0.80344700
H	-1.65613800	-4.62436800	-0.95566600
H	5.15474800	-0.85419100	2.81799900
H	4.09868100	0.54040800	2.54325500
H	3.47199600	-1.11130300	2.30025600

Conformer 6 (Boltzmann population 5.1%)

$\Delta G = -1015.753160$ Hartree

C	-0.88651800	2.00473100	0.03084600
C	0.06492400	1.07143800	-0.28706500
N	-0.24337800	-0.25842500	-0.34510700
C	-1.50195400	-0.73282900	-0.08577000
C	-2.54106600	0.14451500	0.23352200
C	-2.26707100	1.59136700	0.28998900
C	-1.74355200	-2.13079200	-0.14628700
C	-3.01289700	-2.61197700	0.11477100
C	-4.05839800	-1.71808000	0.43642400
C	-3.83026900	-0.36057200	0.49461100
O	-3.15212400	2.40749600	0.54053600
C	1.50725600	1.36826800	-0.62358200
C	-0.66627400	3.49623800	0.15145200
O	-1.49444100	4.22822700	-0.70831800
C	2.45686900	0.54761400	0.21896300
C	3.70564400	-0.06371500	-0.27877300
C	4.84271400	-0.37520700	0.67972600
C	4.14432600	0.08406600	-1.71599100
O	2.53220900	-0.84115900	-0.07210000
O	-0.65862700	-2.87383300	-0.46662200
C	-0.80582700	-4.26911800	-0.52569300
C	4.44413800	-0.52482700	2.14177200
H	0.49441600	-0.93708100	-0.52727600

H	-3.21400500	-3.68169000	0.07258100
H	-5.05198300	-2.12000400	0.63707700
H	-4.61384500	0.35711400	0.73751300
H	1.66149000	1.15099900	-1.69009100
H	1.72198100	2.43094300	-0.47270100
H	-0.84648200	3.77540900	1.20778300
H	0.36920100	3.77528300	-0.08148300
H	-2.39739900	3.99809900	-0.44139200
H	2.38965900	0.76219200	1.29197600
H	5.30028900	-1.31325600	0.32412500
H	5.61205800	0.40447600	0.56147700
H	4.76095700	0.98575900	-1.84021400
H	4.75286700	-0.78561600	-2.00341800
H	3.29153800	0.13677900	-2.40143000
H	0.17583200	-4.67933700	-0.78471200
H	-1.12643200	-4.67656400	0.44697800
H	-1.53824100	-4.55991800	-1.29642000
H	5.27212600	-0.94723200	2.72585200
H	4.17968600	0.44015100	2.59603900
H	3.58153200	-1.20010500	2.23203400

Conformer 7 (Boltzmann population 4.5%)

$\Delta G = -1015.753042$ Hartree

C	-0.02611800	2.04858900	-0.24014300
C	-0.52232700	0.91161600	-0.81765700
N	0.21506100	-0.23853700	-0.84201700
C	1.46326400	-0.32905100	-0.28372300
C	2.06100100	0.78958900	0.30113000
C	1.32749100	2.07025800	0.31734000
C	2.13918000	-1.57551800	-0.29997600
C	3.40028500	-1.67068200	0.25770300
C	4.00295400	-0.53416800	0.84096800
C	3.34623300	0.67768600	0.86578200
O	1.82082900	3.09199000	0.78762300
C	-1.91861800	0.76078300	-1.36282200
C	-0.78014600	3.35099000	-0.10441600
O	-0.11068700	4.42095500	-0.70892800
C	-2.84821200	0.32888500	-0.24757100
C	-3.05233300	-1.07203200	0.16748300
C	-3.41024400	-1.32109600	1.61734600
C	-2.40501400	-2.23204400	-0.55430700
O	-4.02736900	-0.36302900	-0.58257200
O	1.44022500	-2.58414700	-0.87998300
C	2.04951200	-3.84948500	-0.95614800
C	-2.17642000	-1.48286300	2.50078400
H	-0.15647400	-1.07313400	-1.28130600
H	3.93419600	-2.62012900	0.25376800
H	4.99876700	-0.63069200	1.27434600
H	3.78262600	1.57256500	1.30968700
H	-2.26899300	1.70545600	-1.79617700
H	-1.93220500	0.01490400	-2.16899800
H	-1.77708700	3.28350800	-0.56145500
H	-0.93469800	3.53749400	0.97656000
H	0.74362400	4.47458100	-0.25474900
H	-2.97503600	1.09071300	0.53527000
H	-4.02141500	-0.47309500	1.96012900
H	-4.05060400	-2.21637800	1.67811500
H	-1.42194800	-2.47707400	-0.12552400
H	-3.04594900	-3.12140000	-0.46134800
H	-2.29388700	-2.02239000	-1.62608400
H	1.33832800	-4.51356100	-1.45856100
H	2.98282800	-3.80489900	-1.53996500
H	2.27036600	-4.24453600	0.04848500
H	-2.45730000	-1.58586200	3.55738500
H	-1.59301500	-2.37345200	2.22560500
H	-1.51446200	-0.60776200	2.40918500

Conformer 8 (Boltzmann population 3.4%)

$\Delta G = -1015.752793$ Hartree

C	0.03028100	1.93024500	-0.64306800
C	-0.27202700	0.67732300	-1.10061200
N	0.58495400	-0.36528800	-0.90051400
C	1.77750900	-0.22055200	-0.23651900
C	2.15186700	1.01783900	0.29167800
C	1.23529400	2.16722600	0.14770100
C	2.61967300	-1.35005600	-0.07675500
C	3.82217100	-1.21101200	0.59136100
C	4.19885800	0.04480300	1.11538000
C	3.37620800	1.14237500	0.97568100
O	1.45601600	3.25132400	0.67941700
C	-1.59711600	0.31417200	-1.71498900
C	-0.87604700	3.12746000	-0.77319600
O	-1.42247300	3.50158400	0.46700100
C	-2.62861700	0.21443800	-0.60703800
C	-2.88296500	-1.01778100	0.16237600
C	-3.44159700	-0.88935600	1.56615400
C	-2.15103000	-2.30644200	-0.13894500
O	-3.77517300	-0.56623800	-0.84977100
O	2.12922800	-2.49548600	-0.61292500
C	2.90620300	-3.66190700	-0.49933400
C	-4.61921600	-1.82941700	1.80971900
H	0.35553900	-1.29084300	-1.24445300
H	4.48216100	-2.06775000	0.72125200
H	5.15096000	0.13235200	1.63950700
H	3.63149500	2.12229600	1.37942200
H	-1.91262200	1.07381100	-2.44179200
H	-1.52853200	-0.63683000	-2.25849400
H	-0.28785500	3.95496100	-1.20891000
H	-1.71674600	2.92706600	-1.45043600
H	-0.66319900	3.73867000	1.01908000
H	-2.80545100	1.16636300	-0.08608400
H	-2.63277900	-1.07533200	2.29085800
H	-3.76742800	0.15080100	1.71200700
H	-1.20631700	-2.35845700	0.42345000
H	-2.76727500	-3.16888400	0.15179800
H	-1.94391100	-2.40653100	-1.21160100
H	2.34540400	-4.46503300	-0.98886900
H	3.88032600	-3.53983900	-0.99968400
H	3.07378000	-3.92569500	0.55734000
H	-5.06981500	-1.64734000	2.79458800

H	-5.38987500	-1.66553500	1.04320300
H	-4.32002300	-2.88686700	1.77407500

Conformer 9 (Boltzmann population 2.1%)

$\Delta G = -1015.752311$ Hartree

C	-0.63325800	1.50652300	-0.90221200
C	-0.52629900	0.18319300	-1.23492100
N	0.61360600	-0.51131400	-0.95862900
C	1.68916000	0.05830200	-0.32291000
C	1.64627000	1.39171900	0.09269100
C	0.41918600	2.17774300	-0.14545300
C	2.84073600	-0.73110200	-0.07466600
C	3.92559200	-0.16480000	0.56908500
C	3.87878500	1.18517500	0.97977900
C	2.75615100	1.95274900	0.75253600
O	0.27660400	3.31848200	0.28604500
C	-1.67179100	-0.62846400	-1.78497100
C	-1.85996800	2.34746100	-1.13644800
O	-2.52550800	2.64771100	0.06774400
C	-2.71990200	-0.77537200	-0.69636800
C	-2.57029600	-1.68849300	0.44666700
C	-3.20544200	-1.24613400	1.74896000
C	-1.40459000	-2.64198000	0.57017600
O	-3.46540800	-1.96807900	-0.62400000
O	2.74648000	-2.01250600	-0.50765700
C	3.84656700	-2.86275400	-0.29742800
C	-2.36974000	-0.17464000	2.44699100
H	0.68850200	-1.49238800	-1.20137300
H	4.81871400	-0.75639100	0.76606500
H	4.74511600	1.61167400	1.48617900
H	2.68346800	2.99399600	1.06706800
H	-2.10817400	-0.14060900	-2.66734500
H	-1.32837500	-1.62139300	-2.10379000
H	-1.54617500	3.27560300	-1.64591600
H	-2.58038400	1.83921700	-1.79115000
H	-1.89010600	3.16150700	0.58834500
H	-3.29603700	0.13969700	-0.50075800
H	-4.20495500	-0.85304400	1.51113200
H	-3.35196900	-2.12184800	2.40224800
H	-0.56670700	-2.18079800	1.11327100
H	-1.72356100	-3.53568200	1.12663900
H	-1.05521000	-2.97612100	-0.41501200
H	3.57596000	-3.83930800	-0.71237900
H	4.74443100	-2.48404600	-0.81179200
H	4.06464000	-2.97085700	0.77726000
H	-2.83060400	0.12923900	3.39673000
H	-1.35480300	-0.53584500	2.67209900
H	-2.28332900	0.71698500	1.80678500

Conformer 10 (Boltzmann population 1.5%)

$\Delta G = -1015.751984$ Hartree

C	0.68062400	2.01311300	-0.15611400
C	-0.10187100	1.04103900	-0.71722300
N	0.30430300	-0.26397000	-0.72678700
C	1.49137100	-0.67356600	-0.17850000
C	2.36804500	0.25430200	0.38811900
C	1.99595500	1.68242800	0.39584900
C	1.81843900	-2.05341400	-0.18949800
C	3.01761000	-2.47066300	0.35657400
C	3.90296100	-1.52702400	0.92296400
C	3.58606700	-0.18554800	0.94163900
O	2.74448500	2.54309500	0.85132900
C	-1.48175900	1.25847100	-1.28082500
C	0.30149300	3.47060800	-0.03387300
O	1.22387000	4.31697700	-0.65981300
C	-2.53047500	1.04471700	-0.20849500
C	-3.13050900	-0.26180300	0.12851200
C	-3.67948500	-0.46318900	1.52775500
C	-2.77368800	-1.52452700	-0.62285400
O	-3.83465700	0.71588000	-0.62566700
O	0.87455600	-2.84846500	-0.75456900
C	1.13402800	-4.22867400	-0.82821600
C	-5.05170400	-1.13169900	1.52544500
H	-0.27871400	-0.97596800	-1.15214500
H	3.28547800	-3.52649700	0.35597500
H	4.84413300	-1.87729300	1.34758800
H	4.24647900	0.56745100	1.37188500
H	-1.57571400	2.27324600	-1.68574900
H	-1.65940000	0.56783400	-2.11624400
H	-0.68325800	3.66590200	-0.48012900
H	0.21621500	3.70375900	1.04558100
H	2.06653600	4.14827400	-0.21217300
H	-2.48256400	1.78325400	0.60400600
H	-2.95630900	-1.05210300	2.11456000
H	-3.75570100	0.52266300	2.00963700
H	-1.94730200	-2.05659200	-0.12707300
H	-3.64064000	-2.19929800	-0.65460100
H	-2.50061500	-1.30889900	-1.66377800
H	0.26936800	-4.68765500	-1.31911400
H	2.04033200	-4.43005900	-1.42155200
H	1.25628500	-4.66375000	0.17672300
H	-5.47627700	-1.15892700	2.53788000
H	-5.73978300	-0.56775200	0.87963900
H	-5.00731200	-2.16711600	1.15878500

Conformer 11 (Boltzmann population 1.4%)

$\Delta G = -1015.751960$ Hartree

C	0.50248800	2.06625200	0.36593500
C	-0.12349000	0.86995000	0.58917900
N	0.53652700	-0.31615800	0.46939500
C	1.86391600	-0.37599400	0.12664400
C	2.59391500	0.79459500	-0.09393200
C	1.92542100	2.11394600	0.01178000
C	2.49153000	-1.64227400	0.00216700
C	3.83071100	-1.70720400	-0.33781400
C	4.56320600	-0.52071400	-0.55558600
C	3.95674800	0.71189000	-0.43667500
O	2.53292400	3.15631000	-0.18380200
C	-1.57991900	0.73449900	0.95201000
C	-0.24549200	3.36712900	0.41603700

O	-1.35538000	3.31267200	-0.46730300
C	-2.32753400	0.05977400	-0.17371300
C	-3.37641400	-0.96244400	0.00121900
C	-4.38737300	-1.13053100	-1.11398200
C	-3.82403100	-1.42198800	1.36855800
O	-2.05109200	-1.32071800	-0.36810500
O	1.67745000	-2.69897800	0.23830200
C	2.21085500	-3.98928200	0.09181700
C	-5.65415900	-0.30798600	-0.90216700
H	0.00815500	-1.18552300	0.50375100
H	4.32636400	-2.67199800	-0.43784800
H	5.61831500	-0.59357100	-0.82130100
H	4.49076300	1.64781500	-0.60136000
H	-1.66007700	0.14088000	1.87386000
H	-2.01602700	1.72351200	1.12330600
H	-0.58594000	3.57825600	1.44876900
H	0.46773800	4.15432700	0.12916500
H	-1.72160900	4.20009500	-0.54204200
H	-2.33972300	0.65281000	-1.09717100
H	-3.89660200	-0.84785700	-2.05681200
H	-4.63521800	-2.20178100	-1.19614300
H	-4.58138000	-0.74308600	1.78576100
H	-4.26860000	-2.42573000	1.29518400
H	-2.97941700	-1.47874400	2.06457600
H	1.39901800	-4.69308400	0.30365800
H	2.57738700	-4.15423000	-0.93453100
H	3.03728600	-4.16218300	0.80057800
H	-6.33406800	-0.40696200	-1.75886900
H	-6.20292100	-0.63088500	-0.00570200
H	-5.41261900	0.75905200	-0.78418900

Conformer 12 (Boltzmann population 1.4%)

$\Delta G = -1015.751939$ Hartree

C	0.39537200	1.13032200	-0.88540300
C	0.22274000	-0.22047300	-1.02250600
N	-0.96236400	-0.80566900	-0.68009600
C	-2.02663000	-0.09056000	-0.18887300
C	-1.91898100	1.28569600	0.02686900
C	-0.64232700	1.96034900	-0.27738200
C	-3.23828300	-0.76673700	0.10548800
C	-4.30985100	-0.05089300	0.60685600
C	-4.19385200	1.33950900	0.82306500
C	-3.01722000	2.00048700	0.54194600
O	-0.45075300	3.14654000	-0.02404400
C	1.31333400	-1.18594800	-1.40858400
C	1.65019300	1.88081200	-1.24276400
O	2.31854700	2.37741300	-0.10833000
C	2.05930500	-1.59989400	-0.15421900
C	3.40543400	-1.14114700	0.23651100
C	3.70835300	-1.09963600	1.71959100
C	4.24425700	-0.23182200	-0.62586000
O	3.17514700	-2.44382200	-0.29244100
O	-3.21326400	-2.09886000	-0.14370400
C	-4.37489700	-2.84320400	0.12764800
C	3.33575700	0.23759700	2.35336700
H	-1.09235000	-1.80494900	-0.79350100
H	-5.24748100	-0.55445500	0.83849400
H	-5.05134000	1.88399000	1.21943100
H	-2.89249600	3.07144000	0.70267800
H	0.87178900	-2.08412000	-1.86972600
H	1.99582200	-0.75107600	-2.14549200
H	2.35937000	1.24328400	-1.78332900
H	1.36633300	2.70837300	-1.91711600
H	1.69680900	2.99376800	0.30658400
H	1.38589000	-1.87717100	0.67110200
H	3.15688500	-1.92369300	2.19744500
H	4.78020100	-1.31256300	1.86589400
H	4.04960500	0.82229100	-0.38222200
H	5.30779400	-0.44684300	-0.44325600
H	4.05152000	-0.40735600	-1.69199000
H	-4.15335500	-3.88289600	-0.13509800
H	-4.64249800	-2.78581400	1.19503500
H	-5.22432200	-2.48723700	-0.47731900
H	3.49785900	0.21523700	3.43965400
H	3.92712700	1.06375800	1.93550500
H	2.27972900	0.47879700	2.16153000

(2′*R*,3′*S*)-**5a**

M06-2X/def2-TZVP/SMD(MeOH)

Conformer 1 (Boltzmann population 22.8%)

$\Delta G = -976.528600$ Hartree

C	3.08985100	-0.20711000	0.06546100
C	2.69938400	-1.60467900	-0.44168800
C	1.30315500	-1.98260100	0.04245300
C	0.36638900	-0.82277100	-0.08483800
C	0.81490400	0.44609700	-0.31948100
O	2.14041300	0.73324200	-0.49047000
N	-0.95829200	-1.05798800	0.06263000
C	-1.88926500	-0.06686200	-0.02535800
C	-1.49688400	1.24950700	-0.27660000
C	-0.08197800	1.56436100	-0.43935400
C	-3.26061800	-0.38632600	0.14036000
C	-4.20165900	0.60870400	0.05448000
C	-3.80097500	1.93671300	-0.19765900
C	-2.48024500	2.25577600	-0.36054300
O	-3.50757600	-1.69571300	0.37588200
O	0.32827400	2.71678400	-0.66492700
C	3.04660600	-0.14622500	1.59560700
C	3.47248500	1.19590200	2.17621400
C	4.43612700	0.22213500	-0.48279400
O	2.76874700	-1.67329600	-1.85799100
C	-4.86361700	-2.08546900	0.54994600
H	3.42308900	-2.32924600	-0.06821100
H	0.93389500	-2.82324200	-0.54804500
H	1.33002100	-2.30741200	1.08582000
H	-1.27246400	-2.00467400	0.23848400
H	-5.25131100	0.38277300	0.17890300
H	-4.56004400	2.70561300	-0.26174800

H	-2.16812800	3.27322200	-0.55393600
H	2.03401900	-0.36895200	1.93694300
H	3.68850100	-0.94603000	1.97504900
H	3.28268300	1.21501800	3.25010200
H	4.53458800	1.38928400	2.02451100
H	2.90792100	2.01397300	1.72330500
H	4.62811700	1.26600400	-0.23717600
H	5.22551000	-0.39114600	-0.04490300
H	4.46591600	0.11350500	-1.56573300
H	2.34071800	-0.89281800	-2.23705200
H	-4.84780800	-3.15820700	0.72311500
H	-5.30222200	-1.57859800	1.41180200
H	-5.44578300	-1.86421900	-0.34683600

Conformer 2 (Boltzmann population 10.4%)

$\Delta G = -976.527862$ Hartree

C	3.09033900	-0.20072300	0.04065400
C	2.67918500	-1.59518800	-0.44816200
C	1.29935700	-1.96858300	0.07703500
C	0.36336600	-0.81004800	-0.05923800
C	0.81150000	0.45823000	-0.29789300
O	2.13195800	0.75665300	-0.45840900
N	-0.96255100	-1.04768200	0.08246600
C	-1.89544100	-0.06027300	-0.01374300
C	-1.50446800	1.25582500	-0.26830700
C	-0.09001700	1.57261500	-0.42601900
C	-3.26716400	-0.38196200	0.14645900
C	-4.20981600	0.61050000	0.05209800
C	-3.81059900	1.93860200	-0.20352500
C	-2.48989100	2.25969600	-0.36115300
O	-3.51256600	-1.69114500	0.38604000
O	0.31771100	2.72588600	-0.65426500
C	3.10814400	-0.15269600	1.57295500
C	3.60191800	1.16814700	2.14806100
C	4.41876800	0.21942300	-0.55782100
O	2.66874800	-1.56081500	-1.86794100
C	-4.86860300	-2.08239400	0.55615400
H	3.41855900	-2.31678000	-0.09119800
H	0.91409300	-2.82312400	-0.48474000
H	1.35246400	-2.27371900	1.12524300
H	-1.27507500	-1.99453600	0.25961900
H	-5.25958800	0.38286500	0.17227000
H	-4.57099400	2.70559800	-0.27452800
H	-2.17900200	3.27698500	-0.55735100
H	2.09931400	-0.34101700	1.94605700
H	3.73343900	-0.97725100	1.92615700
H	3.45010700	1.18463600	3.22805300
H	4.66463200	1.32419400	1.96116500
H	3.05245400	2.01016900	1.72162300
H	4.63037300	1.25929600	-0.31120100
H	5.21939600	-0.40434500	-0.15631900
H	4.40174300	0.11941200	-1.64149700
H	2.43075000	-2.43744500	-2.19343300
H	-4.85191400	-3.15439800	0.73382100
H	-5.31160800	-1.57268900	1.41409700
H	-5.44756100	-1.86574800	-0.34383800

Conformer 3 (Boltzmann population 9.3%)

$\Delta G = -976.527753$ Hartree

C	-2.96497500	-0.16934700	0.13477800
C	-2.58402100	-1.48257200	-0.57490600
C	-1.18673700	-1.93334700	-0.18817200
C	-0.24629100	-0.77346700	-0.15997900
C	-0.69189600	0.51606200	-0.20004400
O	-2.01586300	0.82982500	-0.29621600
N	1.07932100	-1.03510400	-0.07590500
C	2.01514700	-0.04584800	-0.04330700
C	1.62712000	1.29462700	-0.09485600
C	0.21130500	1.63586500	-0.17510600
C	3.38748500	-0.39191400	0.04163200
C	4.33432100	0.60062500	0.07278700
C	3.93837700	1.95294300	0.02169100
C	2.61665800	2.29800400	-0.05952500
O	3.62881900	-1.72258300	0.08541200
O	-0.19511600	2.81090200	-0.21986800
C	-4.31897400	0.31913300	-0.37099500
C	-4.75275000	1.67452100	0.17275100
C	-2.91914800	-0.29265500	1.65006200
O	-3.46449200	-2.54180200	-0.24909100
C	4.98498600	-2.14003600	0.17089000
H	-2.61394200	-1.28359500	-1.65152900
H	-1.21405300	-2.40168400	0.80076700
H	-0.82653400	-2.68449000	-0.89374900
H	1.38984100	-1.99859800	-0.04358100
H	5.38476500	0.35378400	0.13686300
H	4.70190500	2.71963700	0.04826200
H	2.30841300	3.33409300	-0.09834000
H	-4.26500700	0.36120500	-1.46268900
H	-5.06775800	-0.43326200	-0.11242000
H	-5.66666100	1.99948600	-0.32639600
H	-4.95952600	1.63555800	1.24270000
H	-3.98934600	2.43453800	-0.00051100
H	-3.13913500	0.67039500	2.10970400
H	-3.65916500	-1.01873700	1.98850000
H	-1.93800200	-0.61399300	2.00155800
H	-4.29152900	-2.42866000	-0.73296700
H	4.96351400	-3.22625400	0.19865900
H	5.54700600	-1.80390700	-0.70282200
H	5.44954500	-1.75609800	1.08122900

Conformer 4 (Boltzmann population 9.1%)

$\Delta G = -976.527735$ Hartree

C	3.08710900	-0.20042800	0.03864000
C	2.67177700	-1.60128200	-0.44647700
C	1.29556800	-1.96734200	0.08287900
C	0.35959900	-0.80855500	-0.05476200
C	0.80719300	0.45975700	-0.29263800
O	2.12768600	0.75763900	-0.45438200
N	-0.96594100	-1.04731500	0.08593100
C	-1.89932900	-0.06036500	-0.01129900

C	-1.50871500	1.25633300	-0.26338300
C	-0.09426700	1.57401200	-0.41941300
C	-3.27119000	-0.38337300	0.14466900
C	-4.21435700	0.60857400	0.04945000
C	-3.81546100	1.93738800	-0.20279400
C	-2.49460100	2.25966200	-0.35686500
O	-3.51618300	-1.69315400	0.38103100
O	0.31327700	2.72777500	-0.64606700
C	3.11769000	-0.15300400	1.57105300
C	3.62342100	1.16296700	2.14721000
C	4.41009700	0.22303600	-0.56894300
O	2.57826700	-1.65337700	-1.86290300
C	-4.87259700	-2.08677800	0.54228200
H	3.40940500	-2.32354200	-0.08859800
H	0.91010100	-2.82283300	-0.47477500
H	1.35251900	-2.26365100	1.13273500
H	-1.27813700	-1.99493800	0.25973100
H	-5.26429000	0.37989700	0.16623400
H	-4.57622400	2.70396900	-0.27436600
H	-2.18399600	3.27745400	-0.55090200
H	2.10983000	-0.33563800	1.94961000
H	3.73934800	-0.98267100	1.91915400
H	3.47336800	1.17930500	3.22748800
H	4.68716700	1.31060600	1.95924800
H	3.08021400	2.01005200	1.72270000
H	4.63458700	1.25512400	-0.30236500
H	5.21338700	-0.41640500	-0.19872600
H	4.37870900	0.16393200	-1.65706600
H	3.46294100	-1.63624100	-2.24628500
H	-4.85539600	-3.15935500	0.71638500
H	-5.32117300	-1.58064800	1.39944300
H	-5.44676900	-1.86789200	-0.36024300

Conformer 5 (Boltzmann population 8.3%)

$\Delta G = -976.527642$ Hartree

C	3.07208300	0.14583400	0.03480100
C	2.74854800	-1.11676200	-0.77615200
C	1.41696700	-1.70587000	-0.32775200
C	0.39095700	-0.62467000	-0.20489000
C	0.73895900	0.69541500	-0.15719400
O	2.03641200	1.11870500	-0.24732500
N	-0.91067500	-0.98810900	-0.12784900
C	-1.91629800	-0.07646000	-0.01127300
C	-1.62921200	1.28949500	0.02665700
C	-0.24425400	1.74025600	-0.04901100
C	-3.25746500	-0.52946600	0.06843800
C	-4.27414600	0.38494300	0.18270500
C	-3.97983600	1.76335600	0.22125900
C	-2.68903700	2.21127100	0.14595000
O	-3.39860500	-1.87430300	0.02103700
O	0.07336700	2.94227900	-0.02238500
C	3.09301200	-0.10946200	1.54498400
C	4.05018100	-1.20828200	1.99099700
C	4.35648900	0.79718700	-0.43866200
O	2.72955400	-0.83310900	-2.16708800
C	-4.71792500	-2.39823200	0.09636300
H	3.54129500	-1.84957000	-0.63187800
H	1.08556800	-2.45143800	-1.05279200
H	1.52593300	-2.21185300	0.63576700
H	-1.14977700	-1.97168300	-0.16349900
H	-5.30229200	0.05695500	0.24358800
H	-4.79703900	2.46719700	0.31212800
H	-2.45931400	3.26780800	0.17579300
H	3.36615400	0.83625000	2.01883500
H	2.08278400	-0.34791000	1.88552400
H	4.05845400	-1.27201500	3.07978200
H	3.75298300	-2.18655500	1.60875700
H	5.07229600	-1.01211900	1.66364600
H	4.54973500	1.69800000	0.14543800
H	5.19590500	0.11275300	-0.31483500
H	4.28618700	1.06978100	-1.49077800
H	2.21335300	-0.02911700	-2.31986100
H	-4.61528300	-3.47871700	0.04126100
H	-5.18980700	-2.11963700	1.04078100
H	-5.32237100	-2.04126500	-0.73993100

Conformer 6 (Boltzmann population 7.3%)

$\Delta G = -976.527525$ Hartree

C	-2.96584700	0.01945600	0.42072200
C	-2.66831300	-1.24708300	-0.39033500
C	-1.29335300	-1.79878700	-0.04340900
C	-0.29472600	-0.68690900	-0.02686000
C	-0.67939200	0.62301200	0.01299600
O	-1.98876500	1.00833200	0.03115900
N	1.01993700	-1.01223100	-0.04453900
C	2.00328800	-0.07006000	-0.03766600
C	1.67838500	1.28778700	-0.01362400
C	0.27899600	1.69727400	0.01348000
C	3.35982700	-0.48223700	-0.06038500
C	4.35380700	0.46343400	-0.05542900
C	4.02164200	1.83361000	-0.02864300
C	2.71599500	2.24227400	-0.00839800
O	3.53794700	-1.82335200	-0.08697300
O	-0.07008500	2.89082600	0.03474400
C	-4.32199700	0.62386600	0.06784400
C	-4.59431500	0.79350500	-1.42028600
C	-2.86983400	-0.21071600	1.92241600
O	-3.69124100	-2.18732700	-0.12671300
C	4.87460100	-2.30637100	-0.11448100
H	-2.67434400	-0.96724500	-1.44829400
H	-1.32260800	-2.28222200	0.93773600
H	-0.99208000	-2.55309200	-0.77377600
H	1.28433400	-1.98947400	-0.07204100
H	5.39301000	0.16669600	-0.07239200
H	4.82186100	2.56239900	-0.02511900
H	2.45749600	3.29232700	0.01152400
H	-5.09375300	-0.00075900	0.52155700
H	-4.36724900	1.59480400	0.56788000
H	-5.51240100	1.36375500	-1.56827300
H	-3.78435000	1.33336200	-1.91448500

H	-4.72113600	-0.16794700	-1.92070400
H	-3.19056100	0.69004000	2.44759600
H	-3.51817100	-1.03594000	2.21909700
H	-1.85089900	-0.44260000	2.23491500
H	-3.57617800	-2.94479300	-0.71408200
H	4.80166700	-3.39045900	-0.13614600
H	5.39360600	-1.95293400	-1.00773300
H	5.41675800	-1.99046000	0.77912600

Conformer 7 (Boltzmann population 6.9%)

$\Delta G = -976.527475$ Hartree

C	-2.95812200	0.00951900	0.42310800
C	-2.66645200	-1.25668300	-0.39966500
C	-1.28893100	-1.80206200	-0.07345400
C	-0.29459400	-0.68706000	-0.04395200
C	-0.68014700	0.62239400	0.00255200
O	-1.99050300	1.00433300	0.02249700
N	1.02002500	-1.01178900	-0.05816900
C	2.00281600	-0.06906600	-0.04316600
C	1.67708700	1.28850500	-0.01491400
C	0.27732400	1.69707800	0.01021000
C	3.35959200	-0.48060000	-0.06107700
C	4.35311900	0.46552100	-0.04869600
C	4.02015400	1.83541500	-0.01870100
C	2.71421900	2.24342400	-0.00204200
O	3.53834800	-1.82151300	-0.09070000
O	-0.07254500	2.89042900	0.03623200
C	-4.32183100	0.61012400	0.09415200
C	-4.60513500	0.82534700	-1.38693500
C	-2.84120100	-0.22896600	1.92170500
O	-3.60501500	-2.28338500	-0.14177300
C	4.87525900	-2.30417300	-0.11036200
H	-2.69055400	-0.97750300	-1.45709100
H	-1.30936600	-2.30242300	0.89978700
H	-0.99088700	-2.54324200	-0.81740500
H	1.28503300	-1.98877700	-0.09051100
H	5.39251600	0.16928500	-0.06210000
H	4.81999700	2.56456900	-0.00949800
H	2.45516400	3.29328100	0.02082800
H	-5.08546300	-0.03584200	0.53418600
H	-4.37812700	1.56421000	0.62412100
H	-5.52927700	1.39152300	-1.51003200
H	-3.80283400	1.38774200	-1.86769400
H	-4.72826000	-0.11771900	-1.92316200
H	-3.15790300	0.66655400	2.45811600
H	-3.48281900	-1.05868400	2.22043400
H	-1.81791900	-0.46028500	2.22007000
H	-4.43486200	-2.07439500	-0.58888000
H	4.80268500	-3.38824300	-0.13389200
H	5.39986400	-1.94945000	-0.99981100
H	5.41162100	-1.98929600	0.78711100

Conformer 8 (Boltzmann population 6.1%)

$\Delta G = -976.527355$ Hartree

C	-2.96332000	0.01714700	0.41673900
C	-2.67306600	-1.25210200	-0.40113200
C	-1.29246800	-1.80030200	-0.07633700
C	-0.29583200	-0.68635500	-0.04775000
C	-0.68091100	0.62314900	-0.00226100
O	-1.99068800	1.00685900	0.01341200
N	1.01885200	-1.01177300	-0.05994000
C	2.00207700	-0.06942900	-0.04272700
C	1.67691800	1.28823500	-0.01235900
C	0.27736800	1.69744400	0.01028000
C	3.35878500	-0.48127600	-0.06014700
C	4.35256100	0.46454700	-0.04392200
C	4.02007400	1.83447200	-0.01072600
C	2.71426300	2.24283000	0.00464800
O	3.53734000	-1.82215400	-0.09334000
O	-0.07197200	2.89073000	0.03867400
C	-4.32399500	0.61890900	0.07942800
C	-4.60939900	0.79821000	-1.40538300
C	-2.84646200	-0.20447300	1.91848800
O	-3.67699400	-2.23421000	-0.24337800
C	4.87424100	-2.30475300	-0.11695100
H	-2.69548700	-0.96980000	-1.45520300
H	-1.30639500	-2.29970900	0.89865300
H	-0.99712400	-2.54256300	-0.82034600
H	1.28352600	-1.98879600	-0.09322800
H	5.39189800	0.16809200	-0.05676800
H	4.82013900	2.56333000	0.00176200
H	2.45540600	3.29268900	0.02953600
H	-5.08971500	-0.01258300	0.53418300
H	-4.37053800	1.58601300	0.58672800
H	-5.52770500	1.37105700	-1.54143200
H	-3.80291400	1.33986500	-1.90328700
H	-4.74238700	-0.15924300	-1.91158800
H	-3.13930100	0.70710400	2.44131600
H	-3.51014500	-1.00840600	2.24162200
H	-1.82854500	-0.45709500	2.21774100
H	-3.61026700	-2.62628200	0.63725800
H	4.80165600	-3.38869600	-0.14550700
H	5.39763500	-1.94580100	-1.00541000
H	5.41181400	-1.99408900	0.78126700

Conformer 9 (Boltzmann population 5.5%)

$\Delta G = -976.527261$ Hartree

C	3.06980100	0.15972300	0.02290300
C	2.74196500	-1.11920200	-0.75345000
C	1.41708300	-1.70263900	-0.28302400
C	0.39088700	-0.62001800	-0.18264000
C	0.73709200	0.70096000	-0.15203000
O	2.02940300	1.12956900	-0.23461900
N	-0.91114100	-0.98526900	-0.10652400
C	-1.91918800	-0.07537700	-0.00642500
C	-1.63464400	1.29144500	0.01553300
C	-0.25047400	1.74349100	-0.05935800
C	-3.25995400	-0.53007800	0.07374700
C	-4.27890700	0.38333300	0.17238900
C	-3.98735800	1.76297800	0.19430000

C	-2.69723700	2.21241400	0.11868600
O	-3.39812200	-1.87597300	0.04347000
O	0.06382400	2.94724700	-0.04357400
C	3.12703700	-0.07766000	1.53627600
C	4.09546200	-1.16646300	1.98217600
C	4.34291600	0.80701100	-0.48730900
O	2.67013100	-0.76926600	-2.12845400
C	-4.71666500	-2.40131600	0.12036900
H	3.54371500	-1.84284500	-0.59328400
H	1.08048900	-2.46795100	-0.98631200
H	1.53450600	-2.18796700	0.68997500
H	-1.14786700	-1.96965500	-0.13034400
H	-5.30662400	0.05409200	0.23371500
H	-4.80636900	2.46624300	0.27264300
H	-2.46992300	3.26975600	0.13586100
H	3.40718400	0.87613000	1.98912700
H	2.12377000	-0.31176800	1.90051500
H	4.13735100	-1.20030200	3.07160300
H	3.78516200	-2.15403400	1.63632000
H	5.10738900	-0.98181800	1.61811400
H	4.55088600	1.71108100	0.08676500
H	5.18552400	0.12352500	-0.37992200
H	4.24423100	1.07298300	-1.53839400
H	2.54323200	-1.57475900	-2.64435100
H	-4.61189700	-3.48229300	0.08082400
H	-5.19330000	-2.11057200	1.05874300
H	-5.31825400	-2.05719200	-0.72337000

Conformer 10 (Boltzmann population 5.4%)

$\Delta G = -976.527242$ Hartree

C	-2.97050300	-0.16784200	0.12881800
C	-2.58899800	-1.48001100	-0.57244100
C	-1.18792200	-1.93216400	-0.18150600
C	-0.24499000	-0.77416600	-0.16001500
C	-0.69173600	0.51476400	-0.19844000
O	-2.01570500	0.82817100	-0.29435100
N	1.08104400	-1.03490300	-0.07789800
C	2.01605600	-0.04495400	-0.04422000
C	1.62684600	1.29523200	-0.09447300
C	0.21065400	1.63546000	-0.17381400
C	3.38861300	-0.38999800	0.04124900
C	4.33463600	0.60323900	0.07383200
C	3.93758600	1.95527800	0.02349300
C	2.61561500	2.29934500	-0.05800900
O	3.63089800	-1.72057700	0.08443800
O	-0.19669400	2.81010600	-0.21763600
C	-4.31913700	0.32400700	-0.38869700
C	-4.75897600	1.67317500	0.16511100
C	-2.93624600	-0.28809600	1.64515200
O	-3.54969400	-2.46122800	-0.23005400
C	4.98713300	-2.13703800	0.17364600
H	-2.61642200	-1.28374200	-1.65001700
H	-1.21545500	-2.39330400	0.81045000
H	-0.82683000	-2.68820600	-0.88255800
H	1.39215000	-1.99814000	-0.04511000
H	5.38523400	0.35719300	0.13855500
H	4.70047300	2.72257100	0.05111300
H	2.30658700	3.33523300	-0.09595000
H	-4.24883900	0.37873600	-1.47866300
H	-5.06530600	-0.43618500	-0.15261500
H	-5.66421200	2.00611200	-0.34464200
H	-4.98384600	1.62104800	1.23095400
H	-3.99233100	2.43502300	0.01469000
H	-3.14210000	0.68084700	2.09890800
H	-3.69125300	-0.99941500	1.98181400
H	-1.96278600	-0.62500400	2.00321000
H	-3.37121300	-3.26102700	-0.74033700
H	4.96637100	-3.22327400	0.20161600
H	5.55131900	-1.80074100	-0.69859400
H	5.44893100	-1.75256500	1.08516500

Conformer 11 (Boltzmann population 4.6%)

$\Delta G = -976.527079$ Hartree

C	3.06647000	0.15823900	0.02386400
C	2.73609000	-1.12860000	-0.74949000
C	1.41358900	-1.70404300	-0.27661300
C	0.38759400	-0.62037500	-0.18044700
C	0.73405600	0.70027900	-0.15000100
O	2.02678800	1.12768800	-0.23298100
N	-0.91404600	-0.98599500	-0.10556500
C	-1.92211400	-0.07596300	-0.00623900
C	-1.63732600	1.29080900	0.01754800
C	-0.25306300	1.74282000	-0.05672200
C	-3.26302500	-0.53059200	0.07118100
C	-4.28194700	0.38286700	0.17010700
C	-3.99015500	1.76238000	0.19489300
C	-2.69987600	2.21175500	0.12115600
O	-3.40138400	-1.87634200	0.03806900
O	0.06147500	2.94659400	-0.04017100
C	3.13194000	-0.07392800	1.53804900
C	4.10439700	-1.16090200	1.98048200
C	4.33688900	0.80658500	-0.49270100
O	2.59322100	-0.86961600	-2.13977700
C	-4.72029000	-2.40161900	0.10874700
H	3.53473000	-1.85432900	-0.58629800
H	1.07785000	-2.47260000	-0.97510300
H	1.53265000	-2.17932800	0.70045800
H	-1.15064900	-1.97038800	-0.13157500
H	-5.30979700	0.05367800	0.22943200
H	-4.80911600	2.46565200	0.27371800
H	-2.47246400	3.26904500	0.14018500
H	3.41303700	0.88075200	1.98860200
H	2.13064300	-0.30904800	1.90713300
H	4.15436700	-1.19159900	3.06963900
H	3.79261800	-2.14980800	1.63984300
H	5.11354200	-0.97635400	1.60850900
H	4.55820000	1.70122900	0.09087500
H	5.17900600	0.11881500	-0.40994500
H	4.22572200	1.10106500	-1.53659400
H	3.46234000	-0.71875200	-2.53033700

H	-4.61560800	-3.48247100	0.06571300
H	-5.20003200	-2.11407500	1.04652700
H	-5.31890600	-2.05439400	-0.73583100

Conformer 12 (Boltzmann population 4.3%)

ΔG = −976.527013 Hartree

C	-2.97031200	-0.16789300	0.12168200
C	-2.58936400	-1.48245900	-0.58537000
C	-1.18608300	-1.93313700	-0.20338900
C	-0.24522500	-0.77324600	-0.16933400
C	-0.69206000	0.51575400	-0.20354200
O	-2.01562200	0.82948200	-0.30222500
N	1.08053900	-1.03442700	-0.08341000
C	2.01551600	-0.04461500	-0.04445400
C	1.62644100	1.29575200	-0.09054100
C	0.21044500	1.63625400	-0.17111700
C	3.38796600	-0.38991200	0.04190800
C	4.33392200	0.60325900	0.07873100
C	3.93697800	1.95545700	0.03232200
C	2.61510100	2.29978300	-0.04942800
O	3.63031900	-1.72057200	0.08127600
O	-0.19691400	2.81100500	-0.21008200
C	-4.31945300	0.32370800	-0.39255100
C	-4.76715100	1.66437400	0.17519700
C	-2.93188100	-0.28661200	1.63843400
O	-3.53038400	-2.51232000	-0.35505900
C	4.98658800	-2.13709000	0.16991500
H	-2.62335200	-1.28328200	-1.65923100
H	-1.20431800	-2.40560500	0.78508400
H	-0.82721800	-2.68115500	-0.91298200
H	1.39176500	-1.99782300	-0.05599600
H	5.38445600	0.35701000	0.14364600
H	4.69985100	2.72264500	0.06304400
H	2.30607400	3.33576900	-0.08451800
H	-4.24728600	0.39076300	-1.48169700
H	-5.06240400	-0.44321100	-0.16735700
H	-5.67180500	1.99995400	-0.33383300
H	-4.99543300	1.59905800	1.23963900
H	-4.00273500	2.43077300	0.03635600
H	-3.12625000	0.68469800	2.09212900
H	-3.69639500	-0.98505900	1.98451500
H	-1.96138800	-0.63404800	1.99490200
H	-3.46008500	-2.80981400	0.56174200
H	4.96600900	-3.22340700	0.19451200
H	5.55113600	-1.79797200	-0.70099200
H	5.44784800	-1.75532400	1.08285400

(*R*)-**9a**

M06-2X/def2-TZVP/SMD(MeOH)

Conformer 1 (Boltzmann population 38.7%)

ΔG = −1053.899571 Hartree

C	-0.27878500	-0.79052800	-0.61210900
C	-0.09603400	0.53457700	-0.92668100
N	-1.27074000	1.20996500	-0.71315500
C	-2.22343200	0.33624800	-0.26307100
C	-1.63953000	-0.93788500	-0.17934800
C	-3.56171600	0.56889700	0.07534000
C	-4.30995400	-0.50740800	0.51238300
C	-3.72706600	-1.78913500	0.60676200
C	-2.41259900	-2.02269600	0.27014000
C	0.73327300	-1.90185200	-0.70641300
C	2.00699400	-1.49474800	-0.02293700
C	0.99556700	-2.37629400	-2.13066500
C	1.92548700	-1.16394200	1.48805600
O	3.09134000	-1.43145800	-0.54372900
O	3.05925300	-0.73566000	2.00984800
O	0.93412100	-1.32884500	2.14506800
C	1.13329400	1.27502200	-1.34907500
C	1.88574500	1.80248000	-0.15087800
C	3.20838100	1.87114000	0.00385300
C	3.80659100	2.42406200	1.26559400
C	4.21577000	1.43766800	-1.01921600
O	-3.99966600	1.84755800	-0.05844900
C	-5.35343500	2.10809500	0.28199000
H	-1.40752100	2.19617200	-0.87908400
H	-5.34680600	-0.37745300	0.78857700
H	-4.34467200	-2.60670500	0.95769000
H	-1.98520200	-3.01477000	0.35208000
H	0.34772100	-2.73395200	-0.10570100
H	1.70359300	-3.20599700	-2.14338000
H	0.05961500	-2.70921100	-2.57899900
H	1.40013400	-1.56651600	-2.73937600
H	3.74056800	-0.67984600	1.31302300
H	1.75918200	0.61734100	-1.95154300
H	0.83018000	2.10480400	-1.99619000
H	1.25802400	2.16912800	0.65858900
H	3.04140000	2.70612400	1.98878900
H	4.46991300	1.68780700	1.72928600
H	4.41999300	3.30281600	1.04518200
H	3.77448300	0.97071700	-1.89724000
H	4.79768300	2.30299200	-1.35145200
H	4.92738900	0.73621600	-0.57436500
H	-5.50962200	3.17005300	0.10918600
H	-6.03123400	1.52983200	-0.34980500
H	-5.54133000	1.87573200	1.33253500

Conformer 2 (Boltzmann population 9.6%)

ΔG = −1053.898258 Hartree

C	-0.16999800	0.06730100	0.25332100
C	-0.19908700	-1.02709300	-0.57808600
N	1.07889900	-1.47423700	-0.77346200
C	1.95224100	-0.67422100	-0.08597300
C	1.20876800	0.31690700	0.57802100
C	3.34771800	-0.74723100	-0.00498300
C	3.99766300	0.20127900	0.76069500
C	3.26015700	1.20200400	1.42808400
C	1.88751500	1.27761900	1.35294900
C	-1.36645600	0.85400200	0.71988100

C	-1.16059800	2.26351000	0.23951000
C	-1.60295400	0.79219700	2.22183000
C	-1.16541000	2.44274100	-1.29277000
O	-0.98429700	3.22762500	0.93686000
O	-0.42439200	3.46598600	-1.67264300
O	-1.80983500	1.73993900	-2.02657700
C	-1.37642300	-1.72121700	-1.19076600
C	-2.25629700	-2.33568700	-0.13475400
C	-3.52361500	-2.03177300	0.14775600
C	-4.24958600	-2.73062900	1.26222500
C	-4.34415900	-0.99277700	-0.55964000
O	3.93492400	-1.75734000	-0.69675000
C	5.35012500	-1.85268300	-0.62772600
H	1.33195600	-2.27298600	-1.33638500
H	5.07431000	0.18622900	0.85456800
H	3.80421300	1.92963900	2.01770600
H	1.34706500	2.05616000	1.87638200
H	-2.24174500	0.47601700	0.18417700
H	-2.46520800	1.39783900	2.50480400
H	-1.79016300	-0.24117700	2.51619200
H	-0.73325800	1.15413200	2.77145800
H	-0.47150700	3.56878400	-2.63984500
H	-1.92941700	-1.01119700	-1.80567900
H	-1.00453400	-2.50176700	-1.85974100
H	-1.77066500	-3.10102000	0.46654200
H	-3.62405200	-3.47958200	1.74777200
H	-4.57571100	-2.00729000	2.01576000
H	-5.15236200	-3.22040400	0.88602800
H	-3.81323500	-0.48056800	-1.35993900
H	-5.24193200	-1.45122100	-0.98426300
H	-4.68911500	-0.24171600	0.15811000
H	5.62480200	-2.71288100	-1.23317400
H	5.82031500	-0.95386900	-1.03273100
H	5.67912400	-2.00863700	0.40192800

Conformer 3 (Boltzmann population 7.8%)

$\Delta G = -1053.898061$ Hartree

C	0.79069200	-0.43353400	-0.65970400
C	-0.48963800	-0.76951800	-0.28651200
N	-1.17277500	0.37091500	0.04214800
C	-0.34912900	1.45445500	-0.09811600
C	0.90663500	0.99753000	-0.53189400
C	-0.61862500	2.80858200	0.13260400
C	0.40685400	3.71160700	-0.06985100
C	1.67490300	3.26169600	-0.49291100
C	1.94288400	1.93139500	-0.72699000
C	1.88582700	-1.37767000	-1.08329200
C	2.95555300	-1.26482800	-0.03175700
C	2.43353100	-1.11352100	-2.47876900
C	2.55843500	-1.77207800	1.37148400
O	4.05419800	-0.80376900	-0.19489400
O	3.28798600	-1.21769500	2.32063000
O	1.70582200	-2.60260200	1.54563700
C	-1.16023900	-2.10282100	-0.16573100
C	-2.53124400	-2.11306600	-0.78778500
C	-3.70099200	-2.02079500	-0.15433200
C	-4.99537400	-2.04181700	-0.91611600
C	-3.86294000	-1.87807800	1.33121500
O	-1.87857900	3.10620800	0.54230700
C	-2.17477500	4.47359500	0.78526400
H	-2.14620300	0.39923700	0.31004400
H	0.24876600	4.76754000	0.09813900
H	2.45718400	3.99725300	-0.63436400
H	2.92828700	1.61659700	-1.04565000
H	1.49979700	-2.39764900	-1.01923100
H	3.23837700	-1.81091400	-2.71503800
H	1.63655500	-1.24079800	-3.21126500
H	2.82281100	-0.09972300	-2.56895600
H	3.03171800	-1.57879800	3.18758900
H	-0.53349300	-2.85057800	-0.65326300
H	-1.20524600	-2.37600200	0.89092400
H	-2.54287400	-2.18314300	-1.87265000
H	-4.83415900	-2.14759700	-1.98883200
H	-5.62526300	-2.86849600	-0.57482700
H	-5.56000200	-1.12240200	-0.73654100
H	-2.91921600	-1.83518800	1.87106700
H	-4.42385000	-0.96579400	1.55468900
H	-4.45166200	-2.71004100	1.72840000
H	-3.21528500	4.50643600	1.09851700
H	-1.53999400	4.87352000	1.57902700
H	-2.04636800	5.06619800	-0.12309700

Conformer 4 (Boltzmann population 6.9%)

$\Delta G = -1053.897942$ Hartree

C	0.06682300	0.28484000	-0.83971000
C	0.14945600	-1.08465200	-0.91454500
N	-1.06199500	-1.62550000	-0.57528300
C	-1.94381400	-0.62062700	-0.28094500
C	-1.27730600	0.60647000	-0.43862500
C	-3.28558200	-0.70162200	0.10965200
C	-3.95922800	0.48121000	0.34494400
C	-3.29893400	1.71802200	0.18776400
C	-1.98015700	1.80339800	-0.19836600
C	1.16989000	1.26095900	-1.14890700
C	1.24765300	2.24722200	-0.01399500
C	1.00376700	1.97199300	-2.48533700
C	1.47971700	1.64818800	1.38283300
O	1.15105700	3.44220200	-0.11650000
O	0.72787000	2.22819300	2.29935600
O	2.29288100	0.78616200	1.58932200
C	1.31416200	-1.95835800	-1.26198200
C	2.12152400	-2.29984700	-0.03742400
C	3.38732000	-1.96994600	0.21794300
C	4.02740500	-2.34077600	1.52524000
C	4.28123700	-1.18459100	-0.69633000
O	-3.80146500	-1.95264400	0.22072300
C	-5.16209700	-2.05981800	0.61331800
H	-1.27444400	-2.61230100	-0.57056300
H	-4.99608800	0.47039300	0.64979600
H	-3.85988200	2.62463000	0.37849100

H	-1.50318600	2.76847200	-0.31660200
H	2.11930500	0.71236900	-1.12409000
H	1.81823700	2.67614100	-2.65933100
H	0.99822000	1.23744800	-3.29052500
H	0.06109400	2.52113700	-2.51354600
H	0.91982100	1.84891300	3.17581600
H	0.93062200	-2.87514800	-1.71922100
H	1.92025900	-1.45512000	-2.01341500
H	1.57610400	-2.85307700	0.72415400
H	3.34459600	-2.89339400	2.17049300
H	4.92136200	-2.94887800	1.35949100
H	4.35346100	-1.43971100	2.05390800
H	3.84001300	-0.96852100	-1.66693000
H	4.55055400	-0.23555900	-0.22180400
H	5.21673100	-1.72677800	-0.86088200
H	-5.38491100	-3.12346500	0.64388800
H	-5.31649000	-1.62403400	1.60284300
H	-5.81516300	-1.56871300	-0.11136900

Conformer 5 (Boltzmann population 6.6%)

$\Delta G = -1053.897897$ Hartree

C	0.61719900	-0.70086200	-0.60987400
C	0.42111400	0.54735900	-0.06381600
N	-0.90699400	0.69542000	0.24310800
C	-1.58320300	-0.44690300	-0.08998800
C	-0.65995400	-1.35899100	-0.62461700
C	-2.94300500	-0.75119300	0.04332000
C	-3.36425800	-2.00149600	-0.36763000
C	-2.44007500	-2.92569500	-0.89952400
C	-1.10217600	-2.62874300	-1.03421000
C	1.91455800	-1.28996400	-1.10241400
C	2.89874900	-1.26555900	0.03460900
C	2.47717000	-0.62979800	-2.35422300
C	2.45541400	-1.90889700	1.36645300
O	3.98397900	-0.74758000	0.01059200
O	1.66480400	-2.95310100	1.19160400
O	2.83279200	-1.49327000	2.42857800
C	1.37338400	1.66897100	0.21766200
C	1.00293800	2.92059700	-0.53471900
C	0.38987200	4.00004000	-0.04844400
C	0.08024700	5.17398600	-0.93307800
C	-0.06123400	4.16671800	1.37360000
O	-3.72547200	0.22437700	0.57241800
C	-5.10868400	-0.06175100	0.71858300
H	-1.31873500	1.53526900	0.62445800
H	-4.40409100	-2.28423600	-0.28308800
H	-2.80783000	-3.89672400	-1.20745400
H	-0.40947000	-3.35437400	-1.44322300
H	1.72674900	-2.35063000	-1.30177800
H	3.39650700	-1.12232400	-2.67343200
H	1.74683100	-0.69834500	-3.16027100
H	2.69516300	0.42406700	-2.17597400
H	1.42057200	-3.33550200	2.05356000
H	1.39618100	1.84795800	1.29434100
H	2.37828800	1.35992800	-0.07091500
H	1.25363400	2.90793300	-1.59280700
H	0.42075000	5.01329500	-1.95584700
H	-0.99624300	5.36731600	-0.94948900
H	0.55590800	6.07960800	-0.54560200
H	0.15766800	3.30926500	2.00665300
H	0.40647800	5.05078800	1.81639500
H	-1.14107200	4.34091300	1.39860000
H	-5.55764800	0.82910200	1.15070000
H	-5.56687600	-0.27054400	-0.25067000
H	-5.26096500	-0.91102200	1.38841800

Conformer 6 (Boltzmann population 4.4%)

$\Delta G = -1053.897516$ Hartree

C	-0.22181300	0.74299000	0.69532000
C	-0.02302100	-0.59812700	0.93154200
N	-1.19279000	-1.27071600	0.68805200
C	-2.15725600	-0.38342800	0.29195300
C	-1.58653400	0.89890800	0.27689400
C	-3.49425100	-0.61086000	-0.05499500
C	-4.25431700	0.48033200	-0.43062700
C	-3.68424600	1.77087600	-0.45705600
C	-2.37093000	1.99878800	-0.11179200
C	0.77987900	1.85938400	0.84145400
C	1.99819400	1.52845800	0.02212400
C	1.14407800	2.19571400	2.28207200
C	1.81278600	1.29661400	-1.49834300
O	3.11471500	1.42024200	0.45404000
O	0.70495300	1.84369300	-1.96735400
O	2.62997200	0.71463000	-2.15809800
C	1.20982900	-1.35525300	1.31418300
C	1.94565400	-1.84854000	0.09215600
C	3.26488600	-1.88159600	-0.09464000
C	3.84395800	-2.37864400	-1.38816100
C	4.28834700	-1.45095200	0.91391200
O	-3.91985500	-1.89880100	0.00945100
C	-5.27268000	-2.15233500	-0.34054700
H	-1.31947400	-2.26578200	0.80127700
H	-5.29080700	0.35548600	-0.71030400
H	-4.31087700	2.60019500	-0.76128800
H	-1.95318500	2.99793200	-0.14182500
H	0.33993700	2.74215700	0.36544100
H	1.83795900	3.03656200	2.32020800
H	0.24121100	2.46402900	2.83052300
H	1.60927100	1.34463800	2.78019000
H	0.64087100	1.69899100	-2.92807700
H	1.84755900	-0.71897900	1.92543900
H	0.90998300	-2.20196000	1.94080300
H	1.30763100	-2.20851700	-0.71221000
H	3.06789300	-2.68080400	-2.09154900
H	4.45224900	-1.59826000	-1.85478300
H	4.50673200	-3.23103400	-1.21162600
H	3.85918400	-1.02261300	1.81741900
H	4.90219100	-2.30920400	1.20436400
H	4.96578000	-0.71644800	0.47024400
H	-5.41906800	-3.22345500	-0.22548500

H	-5.95305400	-1.61543700	0.32412200
H	-5.46611700	-1.86457200	-1.37626400

Conformer 7 (Boltzmann population 3.4%)

$\Delta G = -1053.897280$ Hartree

C	0.86556000	-0.27809900	-0.82118300
C	-0.47337200	-0.59113400	-0.81394700
N	-1.18983900	0.52117700	-0.45795800
C	-0.33288500	1.55985700	-0.21643400
C	0.97894600	1.10416500	-0.42850500
C	-0.61870900	2.87023800	0.18454400
C	0.44506300	3.73000100	0.37725100
C	1.76637200	3.27990200	0.17514100
C	2.05216100	1.99258500	-0.22164500
C	2.01045800	-1.19828800	-1.15694400
C	2.82931400	-1.32560000	0.09927200
C	2.85451300	-0.73585600	-2.33665200
C	2.13540200	-2.05418100	1.27094300
O	3.94363000	-0.90202300	0.25619800
O	2.66134700	-1.72711200	2.43561700
O	1.24689600	-2.84854800	1.10808700
C	-1.19073900	-1.86701800	-1.12917600
C	-2.12089600	-2.28186900	-0.01938400
C	-3.44522800	-2.13054400	0.01755500
C	-4.24120800	-2.59269200	1.20479700
C	-4.26979200	-1.49653000	-1.06512900
O	-1.93254200	3.17174100	0.34979100
C	-2.24701900	4.49612100	0.75443200
H	-2.19406300	0.54951800	-0.35364200
H	0.27551800	4.75139400	0.68749700
H	2.57583300	3.98018300	0.34056200
H	3.07737100	1.67596900	-0.36392900
H	1.60215600	-2.19127100	-1.35765100
H	3.67778200	-1.42867400	-2.51623600
H	2.23414900	-0.69334000	-3.23177200
H	3.27364600	0.25486600	-2.16291200
H	2.22193000	-2.22444500	3.14799000
H	-1.73158100	-1.74459200	-2.06964500
H	-0.44979500	-2.65042100	-1.29119400
H	-1.63370700	-2.73098400	0.84258800
H	-3.60791900	-3.04725500	1.96646600
H	-4.78037400	-1.75372900	1.65411800
H	-4.99604300	-3.32243700	0.89764400
H	-3.68412100	-1.14031400	-1.91012500
H	-5.01525500	-2.20584500	-1.43616300
H	-4.82531000	-0.64786500	-0.65520200
H	-3.33073500	4.54038600	0.82855900
H	-1.80323200	4.72093000	1.72675000
H	-1.89967600	5.22087400	0.01483800

Conformer 8 (Boltzmann population 3.4%)

$\Delta G = -1053.897264$ Hartree

C	-0.18897000	0.07778200	0.22844100
C	-0.21800000	-1.03902400	-0.57233900
N	1.05828100	-1.50157500	-0.74112700
C	1.93064000	-0.68951400	-0.06631200
C	1.18758700	0.32407900	0.56300800
C	3.32477400	-0.76845400	0.03055500
C	3.97280400	0.19638500	0.77732500
C	3.23509800	1.21858100	1.41115700
C	1.86380600	1.30039500	1.32015800
C	-1.38270700	0.88570200	0.66227400
C	-1.13858800	2.30161800	0.21528300
C	-1.67750700	0.80514600	2.15339800
C	-0.90937900	2.53365800	-1.29412900
O	-1.05041900	3.25934600	0.93692900
O	-1.63155600	1.72525300	-2.05107400
O	-0.16622800	3.38499200	-1.70043100
C	-1.39631100	-1.74028500	-1.17508900
C	-2.29322500	-2.30559700	-0.10609100
C	-3.55576100	-1.96966500	0.16094600
C	-4.29548700	-2.61347000	1.29923200
C	-4.35610000	-0.94125000	-0.58394700
O	3.91276900	-1.79955700	-0.62869300
C	5.32686300	-1.89965300	-0.54507000
H	1.31118500	-2.31601800	-1.28116600
H	5.04841600	0.17775800	0.88204600
H	3.77785400	1.95787100	1.98729900
H	1.32272400	2.09475300	1.81897500
H	-2.24733200	0.53476200	0.09031300
H	-2.53951400	1.42083300	2.41424200
H	-1.89128500	-0.22946600	2.42439400
H	-0.82178000	1.14501200	2.73854000
H	-1.46634600	1.91360900	-2.99224800
H	-1.93462400	-1.04541400	-1.81976600
H	-1.02718400	-2.54779500	-1.81278700
H	-1.82309700	-3.05716100	0.52421500
H	-3.68368300	-3.35381800	1.81450100
H	-4.61033000	-1.85639300	2.02394100
H	-5.20600600	-3.10180200	0.94022000
H	-3.81854700	-0.47503700	-1.40755200
H	-5.26698500	-1.39514200	-0.98470600
H	-4.67878000	-0.15362900	0.10449700
H	5.60245400	-2.77665600	-1.12548600
H	5.80477400	-1.01390300	-0.96929800
H	5.64637100	-2.03023000	0.49108300

Conformer 9 (Boltzmann population 3.3%)

$\Delta G = -1053.897238$ Hartree

C	0.03977400	0.43847100	0.46834500
C	0.31190900	-0.54118400	-0.46203400
N	-0.85351200	-1.19125200	-0.77461900
C	-1.88972300	-0.64042100	-0.06959400
C	-1.37303000	0.39676500	0.72239600
C	-3.24994800	-0.97052700	-0.07657400
C	-4.09289800	-0.23412400	0.73352900
C	-3.58106700	0.81280700	1.52931600
C	-2.24397400	1.14136100	1.53697800
C	1.01971100	1.39462700	1.10333000
C	1.57123200	2.24234900	-0.00853100

C	2.12514400	0.73236800	1.91188800
C	0.53316100	3.10182000	-0.75853600
O	2.72290900	2.28362000	-0.35609100
O	0.92134600	3.38185700	-1.98880800
O	-0.48386900	3.48803300	-0.24533400
C	1.59872400	-0.97559900	-1.09181500
C	2.16840100	-2.19796500	-0.41794300
C	3.42166700	-2.38312000	-0.00289200
C	3.83211000	-3.67315800	0.64896100
C	4.53225600	-1.38130900	-0.12977300
O	-3.61402700	-1.99417600	-0.89128100
C	-4.99090200	-2.34079100	-0.92036700
H	-0.93136300	-1.94814400	-1.43810200
H	-5.15148200	-0.45051400	0.76238700
H	-4.27392100	1.37010500	2.14765200
H	-1.87436600	1.95421600	2.15013100
H	0.44094900	2.06848000	1.74187200
H	2.77945000	1.48200400	2.35930700
H	1.68606700	0.13175500	2.70845000
H	2.73132000	0.07898400	1.28426500
H	0.26015500	3.94862400	-2.42402500
H	1.39667700	-1.20315800	-2.14419300
H	2.30545100	-0.14885600	-1.07778100
H	1.45948800	-3.01065700	-0.27531800
H	3.00287900	-4.37742200	0.71427100
H	4.64592300	-4.14349200	0.08948600
H	4.21163500	-3.48665200	1.65788700
H	4.22441100	-0.43387600	-0.56791900
H	4.96808800	-1.18028800	0.85332700
H	5.33511100	-1.79548700	-0.74690500
H	-5.07988200	-3.16632200	-1.62219700
H	-5.33224300	-2.66011600	0.06678100
H	-5.59592700	-1.49932200	-1.26503600

Conformer 10 (Boltzmann population 3.2%)

$\Delta G = -1053.897222$ Hartree

C	0.73910600	-0.51202600	-0.63294500
C	0.28318800	0.65178400	-0.05521800
N	-1.04126700	0.50025800	0.26515700
C	-1.45658900	-0.75535100	-0.08665600
C	-0.36353700	-1.43348800	-0.64851800
C	-2.71555900	-1.35108500	0.05226900
C	-2.85966100	-2.65547800	-0.38033200
C	-1.76287100	-3.34617900	-0.93792900
C	-0.52358000	-2.76245000	-1.07803400
C	2.12916500	-0.79821500	-1.14554300
C	3.07029400	-0.65644000	0.01794300
C	2.56016700	0.04553500	-2.33699300
C	2.79385200	-1.60424300	1.20296100
O	3.98670500	0.11964300	0.09331300
O	3.22468700	-1.10478500	2.34621900
O	2.27801700	-2.68220000	1.06548500
C	0.96709500	1.94960200	0.24890300
C	0.34247000	3.09866100	-0.49914600
C	-0.50104000	4.01030900	-0.01429700
C	-1.05152700	5.09580300	-0.89475500
C	-0.99685500	4.05785000	1.40184400
O	-3.68521700	-0.57935100	0.60779200
C	-4.97258800	-1.16096000	0.75173900
H	-1.62022100	1.22206800	0.66990300
H	-3.81158600	-3.15984100	-0.29204100
H	-1.91346700	-4.36908000	-1.26032900
H	0.30585300	-3.31413900	-1.50355500
H	2.15446400	-1.85795100	-1.41807800
H	3.56173200	-0.23410800	-2.66677700
H	1.86577600	-0.11017000	-3.16244300
H	2.56558500	1.10747600	-2.08904800
H	3.05713300	-1.73788200	3.06668200
H	0.93379100	2.11736600	1.32695700
H	2.01914800	1.86886000	-0.02406400
H	0.60436100	3.15384100	-1.55319900
H	-0.67418900	5.02210700	-1.91455000
H	-2.14401900	5.04871200	-0.92229700
H	-0.78982400	6.08029800	-0.49620800
H	-0.59191100	3.27096100	2.03490100
H	-0.75357400	5.02363400	1.85394500
H	-2.08782500	3.97562900	1.41285700
H	-5.60064100	-0.39720200	1.20359600
H	-5.38149300	-1.44444400	-0.22050900
H	-4.93293600	-2.03616900	1.40392800

Conformer 11 (Boltzmann population 3.0%)

$\Delta G = -1053.897146$ Hartree

C	0.84261400	0.41147600	0.78981400
C	-0.52130600	0.58635300	0.77164700
N	-1.11889700	-0.60048400	0.43887700
C	-0.15951800	-1.55221600	0.22498400
C	1.09758300	-0.96131100	0.43228900
C	-0.30772100	-2.89342800	-0.14696200
C	0.83947700	-3.64490300	-0.31245700
C	2.10680300	-3.05880500	-0.11256800
C	2.25729500	-1.74000500	0.25416700
C	1.89028700	1.44667400	1.10530200
C	2.73057400	1.58812000	-0.13656600
C	2.74040000	1.12065800	2.32583600
C	2.01266900	2.03535900	-1.42972100
O	3.90040700	1.32514900	-0.22301800
O	1.01536600	2.87224000	-1.19915100
O	2.36828800	1.66234700	-2.51431000
C	-1.36545200	1.78765200	1.06522900
C	-2.36715700	2.05781300	-0.02608700
C	-3.66860600	1.76756100	-0.01094000
C	-4.54507200	2.09245700	-1.18674600
C	-4.38636300	1.09696500	1.12431900
O	-1.58275700	-3.33003000	-0.31333400
C	-1.75804300	-4.68794600	-0.69022800
H	-2.11416000	-0.73219300	0.32804200
H	0.77690900	-4.68533200	-0.59853100
H	2.98425600	-3.67736000	-0.25545200
H	3.24331600	-1.31663400	0.39748000

H	1.39119000	2.40868400	1.24722400
H	3.49361100	1.89260600	2.48868200
H	2.10263200	1.06441800	3.20782500
H	3.25019600	0.16429500	2.20879300
H	0.60340700	3.14016100	-2.04030900
H	-1.86329400	1.64111300	2.02575600
H	-0.70995900	2.65167900	1.17879400
H	-1.95738200	2.51833100	-0.92178000
H	-3.98709600	2.57710900	-1.98776700
H	-5.00619100	1.18314100	-1.58288600
H	-5.36293000	2.75214700	-0.88265900
H	-3.74187300	0.85219500	1.96607000
H	-5.19908200	1.73388600	1.48523600
H	-4.85048700	0.17149300	0.77081600
H	-2.83099100	-4.84312300	-0.77167200
H	-1.28400700	-4.88811600	-1.65362000
H	-1.34699300	-5.35757700	0.06844800

Conformer 12 (Boltzmann population 2.4%)

$\Delta G = -1053.896951$ Hartree

C	0.07356100	0.27878300	-0.80627200
C	0.14843400	-1.09079600	-0.89047100
N	-1.06940400	-1.62652300	-0.56782600
C	-1.94852800	-0.61814300	-0.27666100
C	-1.27304700	0.60585400	-0.41903800
C	-3.29504000	-0.69337800	0.09855900
C	-3.96398000	0.49207700	0.33385700
C	-3.29427700	1.72579200	0.19272600
C	-1.97084500	1.80547000	-0.17814000
C	1.18500300	1.24713100	-1.10937400
C	1.24321700	2.26611400	-0.00104700
C	1.05569600	1.92211800	-2.46890900
C	1.31289500	1.73722200	1.44333800
O	1.19050900	3.45873700	-0.14859900
O	2.21439500	0.78321700	1.58847200
O	0.62605000	2.18802000	2.32013400
C	1.31232200	-1.96625900	-1.23751000
C	2.14075600	-2.27897500	-0.01956700
C	3.40687900	-1.93294200	0.21161800
C	4.06926600	-2.27625400	1.51553600
C	4.28124400	-1.15648700	-0.72889000
O	-3.81976100	-1.94187800	0.19564300
C	-5.18576600	-2.04327500	0.57077100
H	-1.28776600	-2.61204400	-0.57203500
H	-5.00430400	0.48574500	0.62683600
H	-3.85151300	2.63457700	0.38385100
H	-1.48633900	2.76852200	-0.28321100
H	2.13298500	0.69775600	-1.04923500
H	1.87853300	2.61634100	-2.64285100
H	1.06453400	1.16526100	-3.25318000
H	0.11739800	2.47558000	-2.53450500
H	2.23720400	0.47930900	2.51395100
H	0.92693800	-2.89382400	-1.67048400
H	1.90429600	-1.47486600	-2.00805800
H	1.61135200	-2.82301200	0.75976000
H	3.39728600	-2.81373000	2.18456900
H	4.95912700	-2.88974000	1.34739100
H	4.40828000	-1.36526200	2.01871100
H	3.82327100	-0.95773100	-1.69551300
H	4.55274500	-0.19856700	-0.27363100
H	5.21735000	-1.69553100	-0.90008200
H	-5.41508600	-3.10576000	0.59237900
H	-5.34985900	-1.61232100	1.56084400
H	-5.82688500	-1.54413000	-0.15908300

Conformer 13 (Boltzmann population 2.1%)

$\Delta G = -1053.896820$ Hartree

C	-0.69695600	-0.56199200	0.56442800
C	-0.39732400	0.60928000	-0.09738100
N	0.92765700	0.59799000	-0.44009000
C	1.50129600	-0.57025200	-0.01437000
C	0.50904500	-1.33789400	0.61535400
C	2.82355900	-1.01270400	-0.13645600
C	3.13458300	-2.25638300	0.37996200
C	2.13947200	-3.03713600	1.00553100
C	0.83875200	-2.60302800	1.13203600
C	-2.03560000	-0.97561100	1.11989100
C	-3.04326300	-0.81312700	0.01673500
C	-2.45324100	-0.24765500	2.39016800
C	-2.74112500	-1.53150900	-1.31643300
O	-4.02706000	-0.12266100	0.05957400
O	-2.12357700	-2.68765600	-1.14607600
O	-3.07096300	-1.06960200	-2.37536800
C	-1.28665800	1.76083300	-0.47531300
C	-0.54570800	3.03543300	-0.80066800
C	0.17745200	3.74109100	0.06849000
C	0.89631700	4.99332000	-0.33873300
C	0.35322300	3.34582300	1.50531600
O	3.68322500	-0.16764300	-0.76140500
C	5.02881700	-0.59945500	-0.90156200
H	1.40652200	1.36503000	-0.88950300
H	4.14131000	-2.64301300	0.30753700
H	2.42132700	-4.00814000	1.39381500
H	0.09003000	-3.21956800	1.61465500
H	-1.98129300	-2.05190600	1.31622400
H	-3.41478600	-0.61616900	2.75032600
H	-1.70487900	-0.41195500	3.16542100
H	-2.54113700	0.82569200	2.21877200
H	-1.96055800	-3.10903600	-2.00889300
H	-1.88633200	1.46731600	-1.34207200
H	-1.99603600	1.92542100	0.33941100
H	-0.61121600	3.38600200	-1.82577900
H	0.75840900	5.21374600	-1.39719800
H	0.53940100	5.84609100	0.24565500
H	1.96765000	4.90112500	-0.13762100
H	-0.29053200	2.51842300	1.80338400
H	1.39174800	3.05275700	1.68928600
H	0.14674400	4.19776100	2.15864100
H	5.55064700	0.20104700	-1.42000100
H	5.48781700	-0.76415200	0.07572700

H	5.08271800	-1.51610300	-1.49292300

Conformer 14 (Boltzmann population 1.8%)

$\Delta G = -1053.896692$ Hartree

C	0.03796400	0.46718600	0.45740600
C	0.30410600	-0.51890900	-0.46753000
N	-0.86356100	-1.16918300	-0.77083900
C	-1.89530300	-0.61353000	-0.06306400
C	-1.37329000	0.42675100	0.72122700
C	-3.25639100	-0.94053500	-0.06293200
C	-4.09431100	-0.19879600	0.74750900
C	-3.57681800	0.85035600	1.53677400
C	-2.23907200	1.17635900	1.53684700
C	1.02482200	1.42046400	1.08413500
C	1.64168400	2.22237600	-0.02930000
C	2.08340300	0.75603000	1.95246200
C	0.68108000	2.98352700	-0.96846800
O	2.81608200	2.27407400	-0.28586700
O	-0.40569600	3.40829100	-0.34891300
O	0.94039700	3.17300200	-2.12606600
C	1.58823000	-0.96012900	-1.09793400
C	2.15145800	-2.18529000	-0.42403800
C	3.39797600	-2.36843600	0.01167900
C	3.80240000	-3.66261900	0.65911700
C	4.50642400	-1.36094900	-0.08584600
O	-3.62608600	-1.96646900	-0.87192200
C	-5.00417100	-2.30879900	-0.89584200
H	-0.94603400	-1.92847000	-1.43104300
H	-5.15317600	-0.41294300	0.78188300
H	-4.26568100	1.41104600	2.15651800
H	-1.86481800	1.98994200	2.14637400
H	0.44714100	2.13251800	1.68227800
H	2.74871900	1.50011200	2.39241000
H	1.60014600	0.20020800	2.75591600
H	2.68449900	0.05971300	1.36674900
H	-0.98050700	3.88764800	-0.97177500
H	1.38501700	-1.18676500	-2.15023100
H	2.30091300	-0.13849800	-1.08506500
H	1.44360000	-3.00198200	-0.30032100
H	2.97580700	-4.37172200	0.70144400
H	4.62946200	-4.12326500	0.11111500
H	4.16127700	-3.48393400	1.67696100
H	4.20129200	-0.40765900	-0.51288300
H	4.92849100	-1.17364500	0.90591600
H	5.31888600	-1.76295400	-0.69846600
H	-5.09771300	-3.13645100	-1.59455400
H	-5.34368800	-2.62355500	0.09338000
H	-5.60742000	-1.46658000	-1.24179800

Conformer 15 (Boltzmann population 1.8%)

$\Delta G = -1053.896666$ Hartree

C	0.78347000	-0.48732100	-0.62304800
C	-0.50943200	-0.76333300	-0.24373600
N	-1.14466200	0.41029600	0.06366700
C	-0.27715900	1.45629700	-0.09652900
C	0.95779400	0.93996900	-0.52221500
C	-0.48924000	2.82441100	0.11003300
C	0.57325700	3.67949400	-0.10923500
C	1.82068300	3.16951400	-0.52531600
C	2.03211200	1.82511600	-0.73562000
C	1.84070400	-1.47793800	-1.03628400
C	2.93636700	-1.37060700	-0.00929300
C	2.36955900	-1.27203900	-2.44910500
C	2.56573800	-1.68044100	1.45884300
O	4.06079100	-1.00257900	-0.22292300
O	1.62166500	-2.59984700	1.56588200
O	3.11760300	-1.14273400	2.37977100
C	-1.23649000	-2.06428400	-0.10027000
C	-2.58649800	-2.04185600	-0.76643900
C	-3.77123500	-1.88505700	-0.17485700
C	-5.03996500	-1.87745300	-0.97884700
C	-3.97572500	-1.69200400	1.29974900
O	-1.73495300	3.18248600	0.51479600
C	-1.97182100	4.56546700	0.73353900
H	-2.11635000	0.48406300	0.32925800
H	0.45967600	4.74394700	0.03981800
H	2.63260100	3.86913700	-0.68108900
H	3.00220200	1.46302600	-1.05133200
H	1.42462300	-2.48390600	-0.93848100
H	3.14790000	-2.00062000	-2.67945500
H	1.55491900	-1.39506100	-3.16261800
H	2.78786400	-0.27351500	-2.57488400
H	1.43256800	-2.77686800	2.50487600
H	-0.62522200	-2.85262300	-0.54105200
H	-1.32711300	-2.29745400	0.96285600
H	-2.56633200	-2.14402400	-1.84864000
H	-4.84811200	-2.02094300	-2.04209000
H	-5.71360700	-2.66814600	-0.63595500
H	-5.57239600	-0.93147700	-0.84442000
H	-3.05027100	-1.68344900	1.87204700
H	-4.49485300	-0.74578600	1.47881400
H	-4.62106800	-2.48063300	1.69727200
H	-3.00966300	4.64858400	1.04646100
H	-1.31943100	4.95165400	1.51977300
H	-1.81886500	5.13579700	-0.18525500

Conformer 16 (Boltzmann population 1.7%)

$\Delta G = -1053.896631$ Hartree

C	0.12005000	-1.05181300	-0.74443500
C	0.27241000	0.29250200	-0.99968900
N	-0.90972400	0.93336200	-0.72929500
C	-1.83403100	0.02335800	-0.29207400
C	-1.22608100	-1.24163300	-0.27954800
C	-3.16516500	0.21699000	0.09497000
C	-3.88080200	-0.89035200	0.50882800
C	-3.27279800	-2.16335600	0.53321100
C	-1.96491600	-2.35795100	0.14867200
C	1.15721100	-2.13827300	-0.88667300
C	2.33431600	-1.74081300	-0.04046900
C	1.56253600	-2.45476000	-2.31975800

C	2.05419300	-1.64063300	1.47587800
O	3.43950600	-1.49457400	-0.44670600
O	2.98370300	-0.94650000	2.10511900
O	1.11357900	-2.17576000	2.00062100
C	1.46151500	1.09452900	-1.43310900
C	2.08292500	1.82423000	-0.26772800
C	1.89491600	3.09787800	0.07838500
C	2.57387000	3.67422400	1.28814100
C	1.01161900	4.06680200	-0.65222400
O	-3.62915700	1.49168800	0.02835500
C	-4.97523800	1.71222600	0.42351000
H	-1.06316500	1.92594800	-0.83370900
H	-4.91078100	-0.79114700	0.82124800
H	-3.86459700	-3.00602100	0.86881500
H	-1.51680500	-3.34379400	0.17920900
H	0.74118300	-3.03472200	-0.41725800
H	2.29621600	-3.26186400	-2.34408100
H	0.68375800	-2.76677900	-2.88395800
H	1.99483700	-1.58368500	-2.81229600
H	2.79113100	-0.92454400	3.05911600
H	2.20093300	0.42643900	-1.87376000
H	1.15201500	1.78696300	-2.21727600
H	2.72877200	1.21442200	0.35999200
H	1.83330900	4.05819200	1.99575600
H	3.19120600	2.93427500	1.79763400
H	3.20607200	4.52089700	1.00521800
H	0.55026200	3.65021700	-1.54568000
H	0.21377300	4.41208000	0.01224100
H	1.58258900	4.95391400	-0.94059300
H	-5.15583200	2.77725900	0.30075600
H	-5.66342300	1.14789300	-0.20964000
H	-5.12359700	1.43277500	1.46890100

8a

ωB97X-D/def2-TZVPP/SMD(H_2O)//ωB97X-D/def2-SVP/SMD(H_2O)

ΔG = −1864.503734 Hartree (with quasi-harmonic corrections)

C	5.60963500	-2.08124500	0.61222900
C	7.12278900	-1.98271400	0.43447300
C	7.49628900	-1.15997600	-0.79198700
C	6.88444800	0.25560100	-0.81068100
C	5.35088200	0.11685700	-0.57601800
C	4.56607100	1.42657200	-0.69153000
C	3.06316800	1.17612200	-0.78553900
C	2.53003600	0.35997700	0.39341900
C	3.37388600	-0.92869400	0.52228700
C	4.90918400	-0.70582000	0.67839200
C	2.70447000	-1.88077800	1.51087800
C	1.32447100	-2.24685200	0.97414400
C	0.45817900	-1.01049800	0.68843400
C	-0.67296000	-1.39909800	-0.29867900
C	-1.58266400	-0.23497900	-0.67838100
C	-0.21695300	-0.51206400	1.98257700
C	5.25399100	-0.05112300	2.03007500
C	7.13534200	0.84170400	-2.21007200
C	7.59633600	1.16422200	0.20274400
C	2.46136000	1.25172200	1.64042800
C	-3.04764700	-0.45901300	-0.50046400
C	-3.54150700	-1.72907000	-0.40157800
C	-4.97207800	-1.96296900	-0.36403000
C	-5.80473600	-0.74923000	-0.39286200
C	-7.21273200	-0.83552200	-0.32519200
C	-7.97541700	0.31070300	-0.35873600
C	-7.37191800	1.58255700	-0.46400800
C	-5.99585100	1.69368900	-0.53248500
C	-5.20095100	0.51527900	-0.49431700
C	-2.56331200	-2.86345800	-0.33848000
C	-6.00025000	4.06552400	-0.68986800
N	-3.83962200	0.62899300	-0.55868700
O	1.18866100	-0.00800600	0.00074700
O	-1.20747000	0.76987700	-1.25247300
O	-1.27246400	0.38461300	1.74492900
O	-5.45595400	-3.10256700	-0.30665500
O	-5.29479000	2.84051800	-0.63643100
O	-1.37587200	-2.45634100	0.30484300
H	5.38736600	-2.66071800	1.52215100
H	5.18815900	-2.65039800	-0.23583300
H	7.58426400	-1.54666500	1.33555500
H	7.54540300	-2.99612900	0.34309300
H	8.59331900	-1.08275200	-0.88186100
H	7.14615100	-1.69455600	-1.69342500
H	5.01488500	-0.50455400	-1.42835600
H	4.79011800	2.08942400	0.16006800
H	4.87791400	1.97655800	-1.59053900
H	2.51043200	2.12434400	-0.85399100
H	2.84745500	0.60848800	-1.70563400
H	3.26662200	-1.41122000	-0.46640600
H	2.62433500	-1.43210500	2.51398000
H	3.28889700	-2.80385600	1.62602600
H	0.79480900	-2.91387800	1.66749800
H	1.45634500	-2.80243700	0.03160800
H	-0.20125500	-1.72621300	-1.24696000
H	0.53200700	-0.08382900	2.66303900
H	-0.64473300	-1.39053100	2.48597900
H	5.16857800	1.04227700	2.01675500
H	4.58818400	-0.42667700	2.82091300
H	6.27846700	-0.28811800	2.34425300
H	8.19962200	0.73904700	-2.47684100
H	6.54470000	0.31626600	-2.97817700
H	6.88940000	1.91282000	-2.26540300
H	7.64874600	0.73071800	1.20904200
H	8.63171600	1.34866800	-0.12701300
H	7.09882600	2.14305600	0.28489300
H	2.44219500	0.68972100	2.58280900
H	3.31263900	1.94118200	1.68649500
H	1.54519000	1.85261200	1.57572400
H	-3.41439900	1.54656400	-0.67308700
H	-7.67273200	-1.82124500	-0.24609600
H	-9.06398200	0.24835400	-0.30515000

H	-7.99907500	2.47384800	-0.49019800
H	-2.98225500	-3.68725000	0.25386100
H	-2.34215100	-3.25634600	-1.34966500
H	-6.58790900	4.22541200	0.22805700
H	-5.24459000	4.85430000	-0.77848300
H	-6.66866600	4.10061600	-1.56491600
H	-0.94176600	1.30792500	1.65777500
O	-0.68807300	3.01114900	1.57113000
H	0.00043400	3.29542100	2.18448900
H	-0.28422800	3.12159900	0.67968800
O	0.29750900	3.09717600	-0.95871100
H	-0.35917700	3.58079100	-1.47628700
H	0.05247600	2.16500100	-1.09959400

TSa

*ω*B97X-D/def2-TZVPP/SMD(H_2O)//*ω*B97X-D/def2-SVP/SMD(H_2O)

ΔG = −1864.480586 Hartree (with quasi-harmonic corrections)

C	4.63054900	-2.19907000	0.70012600
C	6.15127600	-2.12582100	0.58752800
C	6.59041400	-1.40543800	-0.68071300
C	5.99653000	0.00919700	-0.83830600
C	4.45201700	-0.09859400	-0.66723000
C	3.68944700	1.20527700	-0.92276300
C	2.18979400	0.95989500	-1.06888800
C	1.59335600	0.24096800	0.14236300
C	2.41455000	-1.04066200	0.41350600
C	3.94302200	-0.81760300	0.62516700
C	1.68668100	-1.90836100	1.43982600
C	0.32061500	-2.29513700	0.87953800
C	-0.49764900	-1.07300300	0.46685700
C	-1.68248700	-1.41554900	-0.45077600
C	-2.58244300	-0.15022700	-0.45243500
C	-1.24837600	-0.41220200	1.63426000
C	4.23186100	-0.06092000	1.93653700
C	6.32006000	0.48245500	-2.26511100
C	6.67027300	0.98793300	0.13510400
C	1.47499400	1.22766600	1.31216200
C	-4.06450300	-0.50335000	-0.41887000
C	-4.52172900	-1.78943400	-0.50393300
C	-5.93505600	-2.07307600	-0.58497500
C	-6.82030300	-0.89646900	-0.53282100
C	-8.22467200	-1.03695900	-0.57828300
C	-9.03061600	0.07975900	-0.54170700
C	-8.47348600	1.37429200	-0.46176800
C	-7.10108400	1.53668000	-0.41562700
C	-6.26374100	0.38869100	-0.44792900
C	-3.52382800	-2.91015700	-0.52164000
C	-7.19390300	3.91003800	-0.30885400
N	-4.90575800	0.54860800	-0.39759300
O	0.27349100	-0.15351700	-0.28840100
O	-2.32626900	0.79098900	-1.29697800
O	-2.31458600	0.33123300	1.05141400
O	-6.37912500	-3.22996300	-0.68910000
O	-6.44244300	2.71245900	-0.34011200
O	-2.32259000	-2.53544900	0.11360100
H	4.35922000	-2.70328900	1.64105900
H	4.24319300	-2.83024400	-0.11986600
H	6.57432500	-1.62206900	1.47201700
H	6.56685600	-3.14618000	0.59704300
H	7.69122600	-1.34395500	-0.72479500
H	6.27618700	-2.00642400	-1.55307200
H	4.14906000	-0.78190300	-1.48374600
H	3.88111300	1.93195700	-0.11648700
H	4.05077600	1.67903400	-1.84627800
H	1.64872800	1.90390100	-1.23435100
H	2.01114600	0.32475000	-1.95181600
H	2.34845200	-1.59791100	-0.53877500
H	1.57096200	-1.38379600	2.40227000
H	2.25211200	-2.82671000	1.64905500
H	-0.24880400	-2.89453600	1.60192900
H	0.47035700	-2.92193100	-0.01457000
H	-1.35039000	-1.61969500	-1.48350700
H	-0.63481800	0.26302500	2.23760900
H	-1.66761400	-1.18854100	2.28996300
H	4.17521100	1.02927700	1.83003100
H	3.51639300	-0.35598900	2.71795200
H	5.23221500	-0.29164000	2.32469600
H	7.39467400	0.35194600	-2.47139100
H	5.76106600	-0.09739200	-3.01760000
H	6.08819200	1.54758700	-2.41551700
H	6.66546600	0.63559300	1.17397500
H	7.72346900	1.13421900	-0.15495900
H	6.18396500	1.97554200	0.11367700
H	1.38076500	0.74089000	2.29126600
H	2.34408900	1.89377900	1.36043900
H	0.58705900	1.85139500	1.14378300
H	-4.51080300	1.48599000	-0.42377200
H	-8.64781300	-2.04014200	-0.64392600
H	-10.11686100	-0.02427700	-0.57609700
H	-9.13282800	2.24209800	-0.43657300
H	-3.91977700	-3.77574400	0.02708600
H	-3.32041300	-3.24168600	-1.55891200
H	-7.85382100	3.94255800	0.57281300
H	-6.46870400	4.72962900	-0.24867600
H	-7.79787700	4.02462200	-1.22314700
H	-2.15864000	1.55583600	1.15973200
O	-1.96995700	2.70335000	1.25692800
H	-1.33898500	2.84164500	1.97921700
H	-1.46795700	2.91802300	0.39327100
O	-0.80485200	2.85971200	-0.99634300
H	-1.24323200	3.48575200	-1.58690200
H	-1.26640900	1.98676600	-1.17791100

1a

*ω*B97X-D/def2-TZVPP/SMD(H_2O)//*ω*B97X-D/def2-SVP/SMD(H_2O)

ΔG = −1864.510491 Hartree (with quasi-harmonic corrections)

C	5.61270000	-2.06615400	0.82943700
C	7.13413500	-1.99825000	0.72382600
C	7.58182200	-1.30255300	-0.55512400
C	6.99294000	0.11063200	-0.74111300
C	5.44742600	0.01047300	-0.57559100
C	4.69006500	1.31192200	-0.85360700
C	3.18975000	1.07199200	-1.00383200
C	2.58238400	0.37151400	0.21305600
C	3.40106500	-0.90808600	0.50700200
C	4.92881000	-0.68470400	0.72538200
C	2.67080300	-1.76520000	1.54029000
C	1.31171200	-2.16498400	0.97471900
C	0.49739700	-0.94845800	0.54799100
C	-0.69081400	-1.28709200	-0.36570600
C	-1.59326900	-0.03596900	-0.23032300
C	-0.26484800	-0.27007100	1.69555000
C	5.20993000	0.09412700	2.02524800
C	7.32416000	0.55696500	-2.17479900
C	7.66484700	1.10491700	0.21762600
C	2.45259100	1.37305100	1.36858200
C	-3.06744900	-0.38329700	-0.31746900
C	-3.50805500	-1.66711800	-0.46854600
C	-4.92010600	-1.95846600	-0.58672000
C	-5.81697600	-0.79484400	-0.49501000
C	-7.21889900	-0.94502500	-0.57443600
C	-8.03517500	0.16182200	-0.49652500
C	-7.49191600	1.45522500	-0.33926000
C	-6.12245800	1.62663900	-0.25775700
C	-5.27450200	0.48844300	-0.33384400
C	-2.50939700	-2.78661300	-0.50993800
C	-6.23579500	3.99132200	-0.03443800
N	-3.91901600	0.66068300	-0.25239300
O	1.26183600	-0.03189500	-0.22007900
O	-1.37788500	0.94081100	-1.18725500
O	-1.31680100	0.46729500	1.07063600
O	-5.34659400	-3.11298100	-0.75202300
O	-5.47543000	2.80100700	-0.10896500
O	-1.31608700	-2.43238000	0.15250700
H	5.33559300	-2.55225800	1.77811500
H	5.22830100	-2.71164700	0.01924500
H	7.55323700	-1.47909700	1.60124100
H	7.54719700	-3.01920200	0.75474300
H	8.68300800	-1.24521000	-0.59490800
H	7.26996700	-1.91849600	-1.41785300
H	5.14612000	-0.68492900	-1.38243800
H	4.88071700	2.04953800	-0.05712300
H	5.05687100	1.77153100	-1.78214200
H	2.65822700	2.01786900	-1.18152800
H	3.01234500	0.42718500	-1.88007800
H	3.34070800	-1.47735500	-0.43845700
H	2.54507700	-1.22861000	2.49476200
H	3.23905600	-2.67780100	1.76522900
H	0.73688600	-2.75660400	1.69921900
H	1.46877000	-2.80009900	0.08802500
H	-0.38436200	-1.43157100	-1.41513400
H	0.32756000	0.42709200	2.29331400
H	-0.69519000	-1.03423000	2.36036600
H	5.15147600	1.18231200	1.90044000
H	4.49131100	-0.18949700	2.80804700
H	6.20887200	-0.12822100	2.42184900
H	8.39956500	0.42119400	-2.37341700
H	6.76801900	-0.03613800	-2.91902900
H	7.09430500	1.61936600	-2.34609600
H	7.65179600	0.77275100	1.26307100
H	8.72052400	1.24092600	-0.06839400
H	7.18318300	2.09404600	0.17396800
H	2.35914100	0.89766100	2.35346600
H	3.31786300	2.04475400	1.41062100
H	1.56084900	1.98996200	1.19306300
H	-3.54818900	1.60569600	-0.19045900
H	-7.63140900	-1.94699100	-0.69899700
H	-9.11952200	0.05084100	-0.55755200
H	-8.15969200	2.31507500	-0.28293400
H	-2.91388200	-3.66746300	0.00653100
H	-2.29302200	-3.08350500	-1.55449600
H	-6.91395300	3.97594600	0.83366700
H	-5.51737700	4.81081100	0.08134700
H	-6.82098000	4.14699700	-0.95475400
H	-1.07969800	2.22993300	1.27810100
O	-0.82985700	3.17332100	1.35350400
H	-0.19004900	3.18446700	2.07671500
H	0.00217200	3.19373300	-0.17696900
O	0.38620800	3.01425400	-1.06593000
H	-0.20859100	3.47511000	-1.67042500
H	-0.52648100	1.41141400	-1.04482600

8b

*ω*B97X-D/def2-TZVPP/SMD(H_2O)//*ω*B97X-D/def2-SVP/SMD(H_2O)

ΔG = −1864.503616 Hartree (with quasi-harmonic corrections)

C	5.23428500	-0.45309900	2.24720500
C	6.62540200	-1.01032700	2.53800600
C	6.96225500	-2.18804900	1.63278300
C	6.83964600	-1.87917600	0.12675100
C	5.43089900	-1.26291700	-0.12336900
C	5.09970800	-1.01061700	-1.59759400
C	3.61272000	-0.73087700	-1.79840300
C	3.10959400	0.43532600	-0.94504700
C	3.51153600	0.18617200	0.52721700
C	5.03389300	-0.04290700	0.77133700
C	2.80829800	1.20124800	1.42724800
C	1.29825500	1.01856700	1.29554600
C	0.81838300	1.06620800	-0.16107600
C	-0.50414700	0.30928800	-0.35028400
C	-1.72704000	0.82192400	0.39391200
C	0.63627600	2.52861200	-0.62416000

C	5.84925300	1.23836300	0.51028900
C	6.95155600	-3.21744700	-0.62236500
C	8.00466400	-0.99281300	-0.33842400
C	3.58132800	1.76035800	-1.56413600
C	-2.90440600	-0.08056500	0.20754200
C	-2.74015800	-1.36322800	-0.23495100
C	-3.89612400	-2.20786900	-0.47090400
C	-5.20008800	-1.58429300	-0.19358700
C	-6.39926700	-2.30275500	-0.39146200
C	-7.61263400	-1.69334200	-0.16268200
C	-7.68070700	-0.34799400	0.25947600
C	-6.52173200	0.37655100	0.46010900
C	-5.26317700	-0.25108400	0.24813700
C	-1.35996800	-1.88510100	-0.50657900
C	-7.66380400	2.35508300	1.14141600
N	-4.11420900	0.45514100	0.46854300
O	1.67726100	0.34803300	-1.04090300
O	0.23291300	2.64460500	-1.96764700
O	-1.82003700	1.82946100	1.05878000
O	-3.78509800	-3.36974300	-0.88658400
O	-6.45805100	1.66912700	0.85113100
O	-0.36050400	-1.04473900	0.02819200
H	5.04221800	0.40835900	2.90606300
H	4.48251000	-1.22104000	2.50397100
H	7.38401200	-0.21866200	2.42433700
H	6.67843500	-1.32623900	3.59221400
H	7.98009700	-2.55687900	1.84599000
H	6.27332000	-3.01948600	1.86746300
H	4.73752500	-2.06206800	0.20235900
H	5.70404200	-0.17883600	-1.99449400
H	5.36774800	-1.89000700	-2.19997300
H	3.39085400	-0.52900500	-2.85915400
H	3.03299300	-1.62268600	-1.50972300
H	3.04418900	-0.78803300	0.76102600
H	3.09810200	2.23270100	1.17117300
H	3.08551000	1.05164800	2.47970200
H	0.75762700	1.78094400	1.87531600
H	1.02842900	0.04023900	1.72085100
H	-0.74620900	0.37148600	-1.42946500
H	1.59302200	3.05336100	-0.50420800
H	-0.08119900	3.01956000	0.05009900
H	6.10458300	1.38420500	-0.54651000
H	5.28896300	2.12515900	0.84116400
H	6.79299300	1.23797000	1.07049400
H	7.84440900	-3.76663900	-0.28235400
H	6.07331800	-3.85700100	-0.43636400
H	7.04950700	-3.08164700	-1.70991800
H	8.13136800	-0.09056900	0.27256900
H	8.94806300	-1.55995900	-0.28264500
H	7.87498100	-0.67375400	-1.38422800
H	3.58461700	2.59990000	-0.85782800
H	4.59413900	1.67311800	-1.97582000
H	2.90283900	2.00993200	-2.39222100
H	-4.15865900	1.45623700	0.66060500
H	-6.33717100	-3.33772100	-0.73003100
H	-8.54271100	-2.24455000	-0.31315400
H	-8.65441700	0.11420300	0.42242700
H	-1.22969800	-2.87633100	-0.05021300
H	-1.21581400	-2.00017700	-1.59950700
H	-8.21016800	1.85946700	1.95869300
H	-7.37341800	3.36435000	1.45455800
H	-8.30726600	2.41926400	0.25024700
H	-0.74495600	2.59030200	-1.99420400
O	-2.50700200	2.43848500	-2.01653600
H	-2.78824800	1.53809000	-2.21956700
H	-3.12400100	2.73731900	-1.31700300
O	-4.31280700	3.43223500	-0.12910600
H	-5.19953400	3.07621100	0.03756500
H	-3.99695100	3.70740700	0.74121100

TSb

ωB97X-D/def2-TZVPP/SMD(H_2O)//ωB97X-D/def2-SVP/SMD(H_2O)

ΔG = −1864.469059 Hartree (with quasi-harmonic corrections)

C	4.60743700	-0.19741100	2.05896100
C	6.10743500	-0.42875500	2.22280500
C	6.59542400	-1.59745200	1.37626300
C	6.25603800	-1.47347800	-0.12320000
C	4.72928600	-1.19061700	-0.24568500
C	4.19667600	-1.16679600	-1.68182900
C	2.67215900	-1.23858000	-1.71173900
C	2.01606100	-0.11924100	-0.90356400
C	2.62129000	-0.10127000	0.52014600
C	4.17550100	0.00919500	0.58971100
C	1.82044600	0.85850100	1.39838400
C	0.37037500	0.39256100	1.44720700
C	-0.24689600	0.19402300	0.06012900
C	-1.53904200	-0.61388900	0.09878000
C	-2.63786900	0.38373600	0.47690800
C	-0.78352300	1.51176700	-0.59367100
C	4.67425700	1.39390600	0.12802100
C	6.57815800	-2.82756600	-0.77659200
C	7.14692000	-0.41577900	-0.79165400
C	2.10961900	1.19869300	-1.67884600
C	-3.95616000	-0.28130000	0.13948600
C	-4.01396500	-1.64248600	0.32125900
C	-5.26288700	-2.34815900	0.14864300
C	-6.43025900	-1.51240800	-0.18523000
C	-7.71027000	-2.08058400	-0.36557300
C	-8.78407300	-1.27468300	-0.67679600
C	-8.62897800	0.12080700	-0.82210100
C	-7.38448000	0.69907200	-0.65222700
C	-6.27551500	-0.12681600	-0.32886900
C	-2.78501500	-2.47055300	0.64377200
C	-8.13641700	2.91787100	-1.06295600
N	-5.04481800	0.45100700	-0.16116700
O	0.63150500	-0.51519400	-0.79892400
O	-2.23310300	1.41412200	-0.63723700
O	-2.62293900	0.92306700	1.65155000

O	-5.35012400	-3.58209000	0.27430800
O	-7.09241600	2.01206400	-0.76230900
O	-1.62122500	-1.71656100	0.95531600
H	4.30450800	0.67080600	2.66526700
H	4.06650100	-1.07093300	2.46510100
H	6.66415300	0.48540200	1.95940500
H	6.33267600	-0.62057400	3.28413000
H	7.68512200	-1.72609800	1.49245900
H	6.12944900	-2.52473200	1.75565700
H	4.26363100	-2.07696300	0.22654400
H	4.54780000	-0.26696100	-2.21250400
H	4.59200200	-2.02294700	-2.24616500
H	2.29734500	-1.21080500	-2.74776900
H	2.34679100	-2.19648700	-1.27431000
H	2.39974600	-1.11246400	0.90782500
H	1.88048800	1.89341000	1.02451600
H	2.21463900	0.87366800	2.42386900
H	-0.26203800	1.08090900	2.01943400
H	0.33424000	-0.57992200	1.96227000
H	-1.70607900	-0.94136200	-0.94407600
H	-0.45219800	1.60089500	-1.63133500
H	-0.50235900	2.41123000	-0.02962200
H	4.84769300	1.45446200	-0.95370900
H	3.94871000	2.17714800	0.39181200
H	5.61970600	1.66531800	0.61563800
H	7.59993500	-3.14208100	-0.50937800
H	5.88492200	-3.61358900	-0.43527800
H	6.52891600	-2.78434800	-1.87505100
H	7.13614500	0.54924700	-0.27016900
H	8.19162700	-0.76663900	-0.80759100
H	6.84483800	-0.23651000	-1.83523500
H	1.80247600	2.07347700	-1.08978800
H	3.13131900	1.38236000	-2.02809500
H	1.46685900	1.13238800	-2.56863000
H	-4.97275100	1.46203100	-0.23807400
H	-7.82495200	-3.15941900	-0.25282600
H	-9.77543900	-1.71030000	-0.81588700
H	-9.49466500	0.73590000	-1.06891900
H	-2.99074300	-3.11795100	1.50819400
H	-2.57133800	-3.13522600	-0.21650200
H	-8.91493200	2.89842700	-0.28375400
H	-7.68145800	3.91435800	-1.09661400
H	-8.59013400	2.69067700	-2.04078100
H	-2.74924700	2.39665400	-0.53030100
O	-3.39469000	3.52482900	-0.35338200
H	-4.11911700	3.57212200	-0.99358400
H	-3.83325000	3.39233300	0.54484400
O	-4.33930400	2.87780000	1.97980700
H	-3.92667000	3.45829600	2.63177100
H	-3.72948700	2.08344100	1.93930600

1b

ωB97X-D/def2-TZVPP/SMD(H_2O)//ωB97X-D/def2-SVP/SMD(H_2O)

ΔG = −1864.502317 Hartree (with quasi-harmonic corrections)

C	5.65137200	-0.05833800	1.97986000
C	7.15730500	-0.27444300	2.10524300
C	7.63167300	-1.45046100	1.26117000
C	7.25075500	-1.34976100	-0.23007000
C	5.71921200	-1.07924800	-0.31555600
C	5.14926100	-1.07473600	-1.73769200
C	3.62474800	-1.15447000	-1.72858100
C	2.98293200	-0.03104700	-0.91448900
C	3.62394300	0.00148300	0.49315900
C	5.17824600	0.12606800	0.52051800
C	2.83788600	0.95902200	1.38652400
C	1.39132300	0.48556200	1.47479600
C	0.74544200	0.28402700	0.10130700
C	-0.55275000	-0.51979900	0.15538600
C	-1.61883800	0.52898500	0.43436200
C	0.18414800	1.60007800	-0.53476900
C	5.65314400	1.50909200	0.02940600
C	7.56509500	-2.71071500	-0.87292500
C	8.11510600	-0.29529700	-0.93725500
C	3.04632100	1.28018700	-1.70361000
C	-2.95230500	-0.11686800	0.14488900
C	-3.05191500	-1.46069600	0.40731600
C	-4.31073000	-2.14535500	0.22000400
C	-5.43829200	-1.30322300	-0.21588900
C	-6.72644500	-1.84920500	-0.40764000
C	-7.76461100	-1.03897300	-0.81318900
C	-7.56468100	0.33903800	-1.04398700
C	-6.31158300	0.89521000	-0.86491400
C	-5.23729500	0.06487100	-0.44764100
C	-1.86025600	-2.30383600	0.81618700
C	-6.98939900	3.10467600	-1.43056900
N	-3.99780300	0.62121900	-0.27169700
O	1.60426900	-0.43258300	-0.77059400
O	-1.25733500	1.55525900	-0.45274900
O	-1.57785000	0.93843000	1.76091300
O	-4.43454000	-3.36662400	0.41497800
O	-5.98209700	2.18964500	-1.04627600
O	-0.66087000	-1.57895500	1.05493900
H	5.35735800	0.81510600	2.58304800
H	5.12958700	-0.93113600	2.41180100
H	7.69832900	0.64078400	1.81417000
H	7.41354800	-0.44982900	3.16235700
H	8.72515200	-1.56898100	1.34916400
H	7.18388500	-2.37609800	1.66567500
H	5.27254200	-1.96345900	0.17847900
H	5.48177300	-0.17916700	-2.28722800
H	5.53463800	-1.93527800	-2.30225800
H	3.22426300	-1.13851500	-2.75521000
H	3.31554500	-2.10985700	-1.27419500
H	3.42180700	-1.00909400	0.89285600
H	2.88119100	1.99254700	1.00649800
H	3.25760000	0.98193500	2.40164600
H	0.77972200	1.18096900	2.06137900

H	1.37024800	-0.48384400	1.99656700
H	-0.71167500	-0.87742900	-0.87864400
H	0.43941500	1.66019900	-1.59776000
H	0.54782200	2.50087800	-0.01921800
H	5.79655500	1.55878200	-1.05724100
H	4.92904100	2.28985300	0.30435900
H	6.60954000	1.79275900	0.48774500
H	8.59661900	-3.01344900	-0.63074400
H	6.88830300	-3.49688000	-0.50034700
H	7.48375900	-2.68399200	-1.97000300
H	8.10816300	0.67806500	-0.43139700
H	9.16227700	-0.63717100	-0.97361600
H	7.78549200	-0.13593500	-1.97573100
H	2.75184800	2.15824700	-1.11313900
H	4.05550800	1.46622800	-2.08628400
H	2.37607600	1.20207900	-2.57184400
H	-3.88837700	1.62894400	-0.39627800
H	-6.87623600	-2.91430000	-0.22667500
H	-8.76222900	-1.45720200	-0.96063100
H	-8.40277800	0.95903000	-1.36300600
H	-2.09096500	-2.85470300	1.73939100
H	-1.68253300	-3.05720900	0.02444100
H	-7.79276000	3.14905400	-0.67806400
H	-6.50409400	4.08475100	-1.50250200
H	-7.41778100	2.83657600	-2.40942600
H	-2.32666900	3.04269900	-0.46620200
O	-3.09469000	3.60909500	-0.25474600
H	-3.59528600	3.68375600	-1.07680800
H	-3.58350400	3.05630600	1.34739600
O	-3.64286500	2.60748200	2.21824100
H	-3.26684400	3.24974800	2.83367400
H	-2.34968600	1.53171200	1.94805500
